# Extending protein language models to a viral genomic scale using biologically induced sparse attention

**DOI:** 10.1093/gigascience/giag081

**Published:** 2026-07-21

**Authors:** Thibaut Dejean, Barbra D Ferrell, William Harrigan, Zachary D Schreiber, Rajan Sawhney, K Eric Wommack, Shawn W Polson, Mahdi Belcaid

**Affiliations:** Department of Information and Computer Sciences, University of Hawai`i, Honolulu, HI 96822, USA; Department of Computer and Information Sciences, Delaware Biotechnology Institute, University of Delaware, Newark, DE 19716, USA; Hawai`i Institute of Marine Biology, University of Hawai`i, Kāne`ohe, HI 96744, USA; Department of Computer and Information Sciences, Delaware Biotechnology Institute, University of Delaware, Newark, DE 19716, USA; Department of Information and Computer Sciences, University of Hawai`i, Honolulu, HI 96822, USA; Department of Plant and Soil Sciences, Delaware Biotechnology Institute, University of Delaware, Newark, DE 19716, USA; Department of Computer and Information Sciences, Delaware Biotechnology Institute, University of Delaware, Newark, DE 19716, USA; Department of Information and Computer Sciences, University of Hawai`i, Honolulu, HI 96822, USA

**Keywords:** Protein language models, Long-context transformers, Viral genomics

## Abstract

**Background:**

The transformer architecture in deep learning has revolutionized protein sequence analysis. Recent advancements in protein language models have paved the way for significant progress across various domains, including protein function and structure prediction, multiple sequence alignments, and mutation effect prediction. A protein language model is commonly trained on individual proteins, ignoring the interdependencies between sequences within a genome. However, biological understanding reveals that protein–protein interactions span entire genomic regions, underscoring the limitations of focusing solely on individual proteins.

**Findings:**

To address these limitations, we propose a novel approach that extends the context size of transformer models across the entire viral genome. By training on large genomic fragments, our method captures *putative* long-range dependencies consistent with inter-protein relationships and encodes protein sequences with integrated information from distant proteins within the same genome, offering benefits across downstream tasks. Viruses, with their densely packed genomes, minimal intergenic regions, and protein annotation challenges, are ideal candidates for genome-wide learning. We introduce a long-context protein language model, trained on entire viral genomes, leveraging a biologically informed sparse attention mechanism in which inter-protein links are *inferred computationally* and used as sparsity priors. Our semi-supervised approach supports long sequences of up to 61,000 amino acids (aa).

**Conclusion:**

Our evaluations show improved prediction of masked aa and improved downstream discrimination relative to single-protein models and long-context baselines, with additional validation that our inferred links correlate with independently curated interaction resources.

## Introduction

Deep learning has transformed the analysis of genomic data, driven by transformer models [1, 2]. This shift produced powerful protein language models (pLMs) such as ESM-2, ProtTrans, and ProGen [3–5], which capture complex relationships between amino acids (aa) within proteins and yield high-dimensional embeddings that are effective across tasks, including protein function annotation [6, 7], multiple sequence alignment [8], and structure prediction [9].

However, by focusing on individual proteins, these models miss crucial interactions between distant proteins that shape genome function. While proximity-based interactions such as those within operons are well studied [10], growing evidence highlights long-range protein interactions across large genomic distances [11]; in evolutionary terms, when one protein changes, its partners often must adapt to preserve their collective function [12]. Yet transformer-based models, constrained by limited context windows (e.g., ESM-2 is limited to 1,024 aa, or tokens), cannot process multiple long proteins simultaneously, preventing them from modeling the web of protein interactions across entire genomes.

Recent large language models (LLMs) show that extended context improves the capture of long-range relationships in text [13], suggesting that analogous approaches could yield more accurate protein embeddings and reveal functional relationships between distant proteins that current methods miss. However, extending context is computationally demanding, primarily because attention scales quadratically with sequence length [14]. Existing solutions—from sparse attention patterns to architectural modifications—are optimized for text and do not account for the principles of protein biology, limiting their effectiveness on multiple protein sequences.

To address these limitations, we introduce a pLM that processes entire viral genomes, achieving a context window exceeding 60,000 aa on a single commodity GPU—covering 98% of all sequenced viral genomes in the NCBI Virus database [15] (Supplementary Fig. S1). Our semi-supervised model employs sparse attention guided by computationally inferred protein–protein interactions (PPIs), and our evaluations show that the resulting viral genome embeddings capture biologically meaningful signals missed by current single-protein models.

## Related work

### Long context LLMs

Recent advances in LLMs have sparked intense research into efficiently handling long contexts. Approaches to increasing context length fall broadly into 4 popular categories [13]: (1) context length extrapolation, (2) hardware and algorithmic strategies, (3) prompt compression, and (4) attention matrix approximation. Context length extrapolation methods enable models to process sequences longer than those on which they were initially trained by manipulating positional embeddings. Hardware and algorithmic strategies focus on optimizing computation and memory usage through system optimizations. Prompt compression methods reduce the sequence length by condensing the input information into more compact representations before processing. Lastly, attention matrix approximation employs computational and statistical strategies to estimate attention mechanisms without incurring the quadratic scaling costs of standard attention. We briefly review these approaches below since our approach builds on advances from several of them. From context length extrapolation methods (the “Context length extrapolation” section), we adopt ALiBi positional encoding (PE) for stable inference on extended sequences. From attention matrix approximation strategies (the “Attention matrix approximation” section), we build on sparse attention patterns similar to Longformer, but replace fixed patterns with biologically informed sparsity derived from inferred PPIs. We leverage ESM-2 both as a foundation for PPI inference and as initialization for one of our models. This section provides background on each of these areas to contextualize our contributions.

#### Context length extrapolation

A fundamental limitation in extending transformer models to longer sequences lies in PEs. While PEs are essential for encoding the sequential order of tokens, their effectiveness diminishes significantly when processing sequences that exceed the training data length. This constraint represents a critical bottleneck when adapting models trained on shorter sequences to handle longer inputs.

Approaches such as rotational position embeddings (RoPEs) [16] introduced trainable embeddings that apply position-based rotations to query and key representations. However, traditional PEs suffer substantial performance degradation when processing sequences that are longer than their training context. Two families of methods address this without full retraining: extrapolation and interpolation. Extrapolation methods, exemplified by Attention with Linear Biases (ALiBi) [17], introduce unlearned static biases into attention calculations, systematically adjusting attention scores between key and query pairs based on distance. In contrast, interpolation methods generalize learned PEs to longer sequences, for example, by scaling RoPE position indices to the original context window [18].

#### Hardware and algorithmic strategies

Training transformer models directly on longer sequences would eliminate the need for positional embedding extrapolation techniques. However, this approach is fundamentally constrained by the quadratic memory complexity inherent to the attention mechanism. Specifically, standard attention mechanisms require memory and computational complexity of $O(n^2 d + n d^2)$ per layer, where *n* represents the sequence length and *d* denotes the hidden dimension size.

Distributed computing architectures address these constraints by leveraging model parallelism, distributing the memory and computation demands of attention across multiple GPUs.

Recent algorithmic innovations have substantially improved the efficiency of attention computation without altering its theoretical complexity. For example, FlashAttention [19] optimizes memory access patterns through fused kernel operations implemented in specialized computing frameworks such as Triton [20]. These practical optimizations reduce memory requirements significantly while preserving computational performance.

#### Prompt compression

Prompt compression methods aim to reduce input dimensionality by exploiting redundancy within input sequences, ideally preserving semantic content. However, this approach introduces a fundamental limitation in protein sequence analysis: compressed inputs cannot generate detailed per-residue embeddings for every amino acid, as compression inherently reduces sequence representation granularity. Consequently, prompt compression is less suitable for tasks requiring residue-level detail, such as protein structure prediction or mutation effect prediction.

#### Attention matrix approximation

An alternative approach to managing extended contexts involves approximating rather than explicitly computing the full attention matrix. This strategy includes two primary methodologies: context-agnostic and context-aware sparse matrices, each offering distinct trade-offs between computational efficiency and representational capacity.

##### Context-agnostic sparse matrices

**Figure 1:**
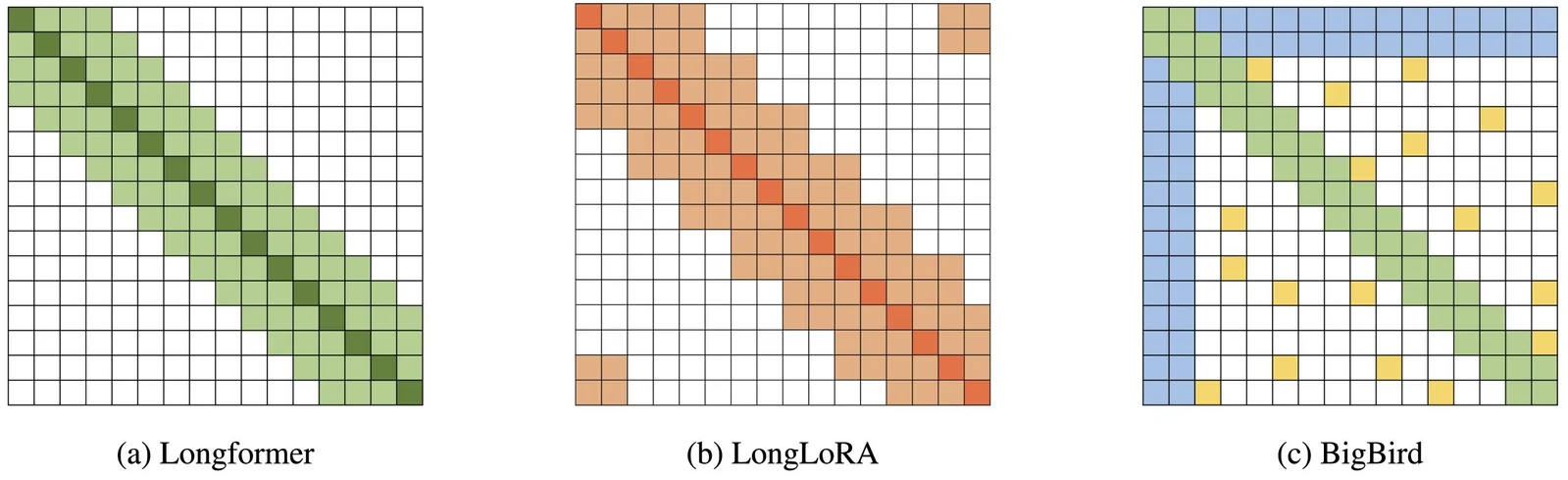
Sparse attention patterns implemented in context-agnostic transformer architectures. (a) Longformer’s dilated sliding-window attention pattern, showing a diagonal band of attention with varying intensities of green. (b) LongLoRA’s shifted attention pattern during fine-tuning, displayed as orange blocks. (c) BigBird’s hybrid approach combining global attention (blue columns), sliding-window attention (green diagonal), and random attention connections (scattered yellow squares). These predetermined sparse attention patterns reduce computational complexity while enabling longer context windows, though they operate independently of sequence content and semantic meaning.

Context-agnostic approaches address the quadratic complexity of traditional self-attention through systematic approximation techniques [14]. Among the most successful implementations are architectures leveraging predefined sparse attention patterns, exemplified by Longformer, LongLoRA, and BigBird [21–23], each employing distinct sparsification strategies (Fig. 1). Longformer implements dilated sliding-window attention, strategically positioning sparse attention windows around tokens (Fig. 1a). LongLoRA extends context length by introducing shifted attention patterns during fine-tuning (Fig. 1b). BigBird employs a hybrid approach combining global attention for select tokens, sliding-window attention, and random attention connections (Fig. 1c). While BigBird enables whole-sequence consideration, the stochastic nature of random attention introduces variability in performance, as randomly selected attention positions lack inherent semantic or functional relevance. Although these approaches successfully extend context windows, their predetermined patterns remain independent of sequence content and semantic meaning, presenting inherent limitations. Most notably for biological applications, these methods cannot incorporate domain-specific knowledge—such as experimentally validated PPI or genomic relationships—into attention calculations.

##### Context-aware sparse matrices

In contrast to context-agnostic methods, context-aware compression approaches dynamically adjust their attention patterns based on input content. For example, the Routing Transformer [24] employs *K*-means clustering on key and query vectors to construct sparse, content-dependent attention matrices.

However, the exclusive reliance on clustering for routing attention presents significant limitations when analyzing biological sequences. Crucially, clustering-based methods may fail to capture interactions between functionally related but structurally and sequentially distinct elements, such as complementary protein domains that co-evolved despite limited sequence similarity.

### Protein and genome language modeling

#### Protein language models

Current popular pLMs, including ProtBert [25], ProtTrans [4], and ProGen2 [5], utilize transformer-based architectures with context windows ranging from 512 aa (ProtTrans-XL) to 2,048 aa (ProGen2). These context limitations have largely confined pLMs to analyzing individual proteins in isolation, neglecting their broader genomic context. Among these, the ESM-2 model [3], trained on the UniRef50 database [26] via masked language modeling, has emerged as one of the most widely adopted pLMs. ESM-2 demonstrates impressive performance in protein contact prediction—attributed to its effective attention mechanism—and generates biologically meaningful embeddings useful across diverse downstream applications [6–9, 27]. However, ESM-2, like its predecessors, focuses exclusively on single-protein sequences with a restricted context length of only 1,024 aa, thereby limiting its ability to capture inter-protein relationships within genomic contexts.

To integrate genomic context into protein representations, the Protein Set Transformer (PST) [28] introduces a graph-based framework wherein proteins, initially embedded via ESM-2, form nodes connected by edges representing genomic proximity. Despite these advantages, PST relies on precomputed single-protein ESM-2 embeddings and is constrained to modeling interactions between consecutive proteins.

#### Genomic language models

Recognizing the inherent limitations of analyzing proteins in isolation has spurred the development of genomic language models (gLMs) [29], which augment ESM-2 embeddings by incorporating context from larger genomic regions. For instance, recent gLMs utilize 30-gene fragments in a masked prediction task, enabling the prediction of protein embeddings by considering the contextual influence of 29 neighboring proteins. This approach generates enriched representations that significantly improve performance across tasks such as protein function prediction and taxonomic classification. However, existing gLMs primarily utilize conventional attention mechanisms, highlighting potential opportunities for incorporating biologically informed sparse attention patterns to further enhance their capabilities.

While existing methods have significantly advanced general language modeling and biological sequence analysis, important limitations remain. Current context-extension techniques in language models typically lack biological specificity. Although extrapolation methods such as ALiBi demonstrate effectiveness in general language tasks, they inherently assume consistent structural patterns between short and extended sequences (for instance, local grammatical structures remain stable regardless of sequence length). This assumption does not hold for multi-protein sequences, where relationships between distant proteins can fundamentally differ from the local sequence patterns learned by single-protein models. Additionally, while content-dependent sparse attention mechanisms show promise for managing extended contexts, their potential for explicitly incorporating biological priors into language model architectures remains largely unexplored.

To address these limitations, we introduce a novel genome-scale pLM architecture specifically designed for viral sequences. Our approach synthesizes recent advances in context-length extension methods with biologically informed sparse attention mechanisms, enabling the effective processing of significantly longer amino acid sequences on commodity hardware while explicitly modeling inter-protein interactions. The following sections detail our methodology and validate its effectiveness across multiple downstream biological tasks.

## Methods

### Datasets and preprocessing

#### Sources and retrieval

We used the NCBI Virus database (retrieved 2024-06), selecting only genomes annotated as “complete” (14,436 genomes) (Supplementary Materials S1, Supplementary Fig. S1). The list of loci used is included in the GitHub repository (https://github.com/Hawaii-Bioinformatics/ViralEmbed). For downstream classification tasks, the final dataset comprised 100,000 proteins from 5,000 genomes after de-duplication.

#### Protein non-redundancy

To prevent highly abundant sequences from biasing training, we applied CD-HIT clustering at 90% sequence identity, reducing the dataset by approximately 50%. Some viral proteins occur dozens or hundreds of times in the database; without deduplication, such sequences would dominate gradient updates and bias learned representations. This threshold collapses near-identical sequences while preserving biologically distinct variants.

#### Splits

To prevent data leakage, we partitioned the dataset at the genome level: all proteins from a given genome were assigned to the same split (train, validation, or test). Splits were stratified by taxonomic species to maintain balanced representation across partitions. The final proportions were 80% train, 10% validation, and 10% test. This design ensures that no protein from a genome in the test set could have been seen during training, either directly or through related proteins from the same genome.

#### Fragments

For long-context baselines, we constructed fragments by concatenating proteins in genomic order with separators, truncating or padding to target lengths as specified.

#### Metadata

For downstream labels, we used (i) NCBI taxonomy (species/family), and (ii) Pfam domain family assignments from HMMER3 against Pfam 35.0 to create task-aligned labels used in “Evaluating genome embeddings quality” and “Evaluation of embedding quality for classification” sections.

### Training and evaluation pipeline overview

**Figure 2:**
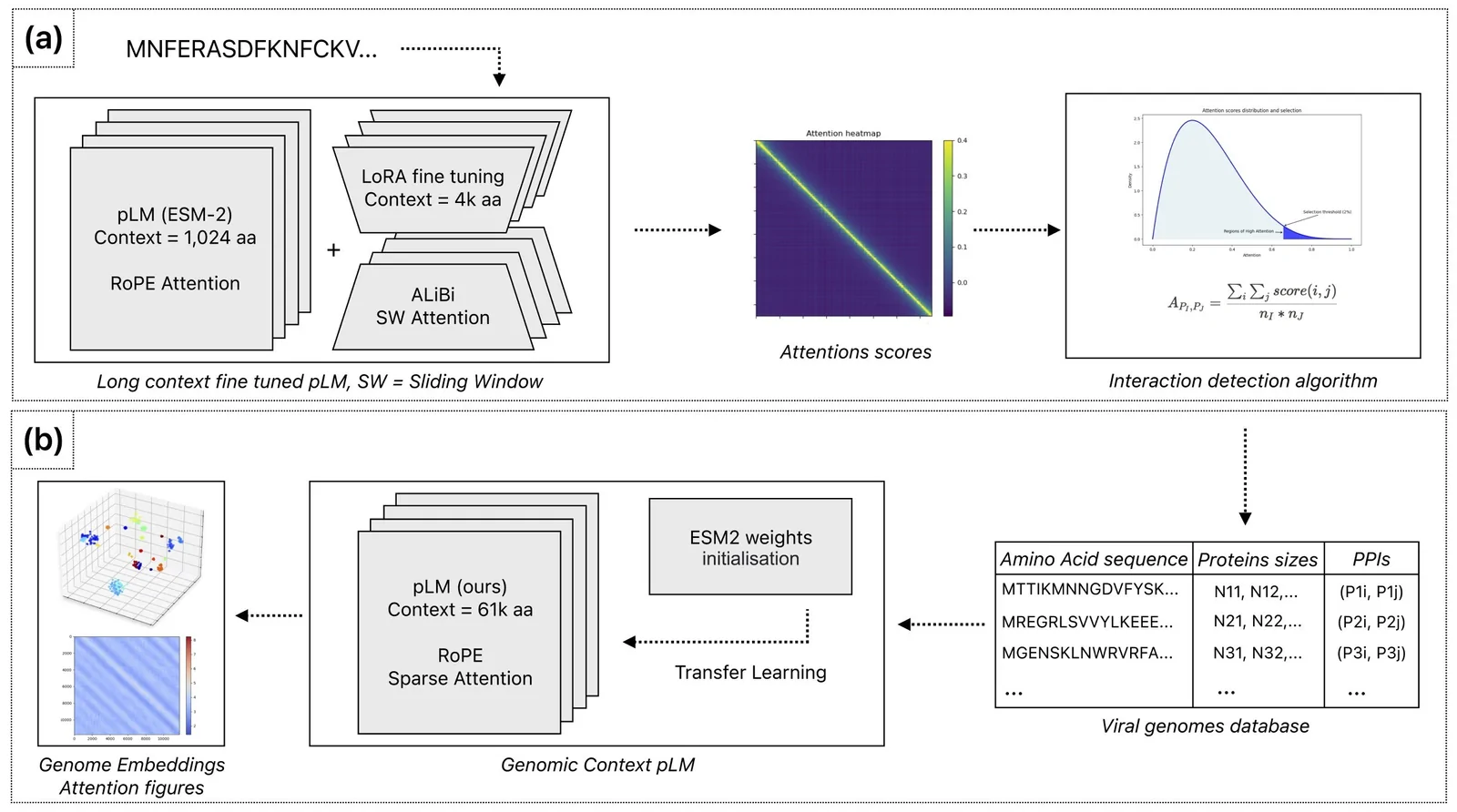
Two-stage approach for training a pLM on complete viral genomes. (a) Identifying putative PPIs on multi-protein fragments using an extended-context ESM-2 model combining LoRA fine-tuning and ALiBi positional extrapolation. The attention scores produced by this model are subsequently used to inform the protein interaction detection algorithm. (b) Training of two long-context pLMs from scratch or initialized from ESM-2 weights using sparse attention patterns guided by inferred protein interactions, enabling efficient whole-genome embedding generation.

Our approach involves three distinct stages, each employing different models (Fig. 2).

#### Stage 1—PPI inference model (ESM-2-PPI)

Training pLMs on entire viral genomes is computationally demanding due to the quadratic scaling of attention mechanisms with sequence length. While existing sparse attention approaches can capture co-evolutionary signals, they typically use static or random masking patterns that neglect biological context. To overcome this, we develop a biologically informed sparse attention method that restricts attention computations exclusively to protein pairs identified as likely interaction partners. Ideally, experimental PPI databases would guide this identification; however, the limited coverage of viral genomes in these databases necessitated a computational strategy. Specifically, we fine-tune ESM-2 (650M parameters) with ALiBi PE (selected after comparing against RoPE; see Supplementary Materials), LoRA adaptation, and Longformer-style sliding window attention (selected after comparing against Shifted Sparse Attention and BigBird; see Supplementary Materials). This extends the effective context window to approximately 13,000 aa, enabling analysis of multi-protein genomic fragments. This model is used exclusively for extracting attention scores to infer PPIs and is not employed for any downstream embedding or classification tasks. The “Developing the PPI inference model (Stage 1)” sections in Methods and Results describe the optimization of this model and the results.

#### Stage 2—Genome-scale language models (LV-3C and LV-5B)

Using the PPIs identified in Stage 1 as biologically informed sparsity priors, we train two novel long-context pLMs. Both models use identical sparse attention patterns; they differ in scale and initialization:

- **LV-3C** (317M parameters; 16 layers): trained from scratch on sequences up to 61,000 aa. Its lighter architecture prioritizes processing longer genomic contexts.
- **LV-5B** (756M parameters; 33 layers): initialized from ESM-2 (650M) weights and trained on sequences up to 48,000 aa. Its larger architecture prioritizes richer pretrained representations.

This complementary design allows us to evaluate two strategies for incorporating genomic context: maximizing sequence length versus leveraging pretrained knowledge. These models represent the primary contribution of this work and are described in “Developing the PPI inference model (Stage 1)” and “Sparse attention computation (Stage 2)” sections.

#### Stage 3—Baseline models for evaluation

To benchmark LV-3C and LV-5B, we compare against several ESM-2 variants:

- **ESM-2-pt**: The original pretrained ESM-2 model with a 1,024 aa context window.
- **ESM-2-RoPE**: ESM-2 fine-tuned with RoPE PE for extended context inference.
- **ESM-2-ALiBi**: ESM-2 fine-tuned with ALiBi PE for extended context inference.

These baselines isolate the contribution of our biologically informed sparse attention mechanism from general context extension strategies.

Throughout the manuscript, we use these standardized names to refer to each model variant. The following sections describe each stage in detail: the “Developing the PPI inference model (Stage 1)” section covers Stage 1 (PPI inference), “Sparse attention computation (Stage 2)”–“Model training (Stage 2)” sections cover Stage 2 (model training), and “Evaluation metrics (Stage 3)”–“Validation against experimentally curated interactions” sections cover Stage 3 (evaluation).

### Developing the PPI inference model (Stage 1)

To extend ESM-2’s context for PPI inference, we evaluated multiple approaches for achieving a context window of 13,000 aa (Supplementary Materials S2). Specifically, we compared two PE strategies (RoPE and ALiBi) (Supplementary Fig. S2) and three sparse attention mechanisms (Longformer, Shifted Sparse Attention, and BigBird) (Supplementary Table S1). Performance was benchmarked against the baseline ESM-2 650M model using both perplexity, which quantifies how well the model predicts amino acid sequences (lower is better), and silhouette scores, which measure how well protein embeddings cluster by structural similarity (higher is better).

PPIs can be inferred from attention scores between proteins in language models, as demonstrated by previous work. For instance, [30] showed that specific attention heads in ESM-1b capture distinct protein relationships, and subsequent work extended these findings to larger models, including ESM-2 [3]. Our approach builds upon this concept by analyzing attention patterns simultaneously across multiple proteins within viral genomes to rank putative interaction links. Standard pLMs, such as ESM-2, have a context length limitation of 1,024 aa, restricting analysis to only 3–5 average-length proteins at once. To overcome this, we first extended ESM-2’s context window to 4,000 aa using attention matrix approximation, and subsequently increased it further to 13,000 aa through context length extrapolation (ALiBi). For the initial extension, ESM-2 was fine-tuned on concatenated protein fragments from the NCBI Viral database [15] using an NVIDIA A6000 GPU with 48 GB RAM. Protein fragments were constructed by sequentially concatenating complete proteins in their genomic order until reaching the 4,000 aa threshold, padding as necessary. To enable efficient training on standard GPU hardware, we used LoRA for parameter-efficient fine-tuning, while Longformer was selected as the sparse-attention pattern based on the comparisons in Supplementary Table S1.

#### Interaction threshold selection

A key parameter in our sparse attention mechanism is *k*, the number of top-ranked non-adjacent protein pairs included in the attention mask for each genome. To select an appropriate value, we varied $k \in \lbrace 10, 20, 35, 50, 100\rbrace$ and evaluated downstream performance on taxonomy and Pfam classification tasks (Supplementary Materials S2). We also tested an alternative policy that includes genomically adjacent pairs in the ranking. Supplementary Table S2 shows that *k=50* maximizes *F*1 score with modest computational cost, while including adjacency during selection provides no benefit since consecutive protein attention is already explicitly included in the sparse pattern (see the “Sparse attention computation (Stage 2)” section).

For each protein fragment analyzed by the fine-tuned model, attention matrices from all encoder layers were extracted. For each layer *ℓ* and attention head *h*, we denote the token-level attention matrix as $M^{(\ell ,h)} \in \mathbb {R}^{L\times L}$ and use *p,q,r,s* for token positions. Each matrix was normalized and symmetrized using average product correction (APC), yielding $N^{(\ell ,h)}$ computed as [3]


\begin{eqnarray*}
\mathrm{ APC}^{(\ell ,h)}_{p,q} &=& \frac{\sum _{s=1}^{L} M^{(\ell ,h)}_{p,s}\,\,\sum _{r=1}^{L} M^{(\ell ,h)}_{r,q}}{\sum _{r=1}^{L}\sum _{s=1}^{L} M^{(\ell ,h)}_{r,s}}, \\\widetilde{M}^{(\ell ,h)}_{p,q} &=& M^{(\ell ,h)}_{p,q} - APC^{(\ell ,h)}_{p,q}, \\N^{(\ell ,h)}_{p,q} &=& \frac{1}{2}\left(\widetilde{M}^{(\ell ,h)}_{p,q} + \widetilde{M}^{(\ell ,h)}_{q,p}\right).
\end{eqnarray*}

Here, *ℓ* indexes layers, *h* indexes attention heads, and $p,q,r,s \in \lbrace 1,\dots ,L\rbrace$ index aa positions within the sequence.

From each normalized attention matrix $N^{(\ell ,h)}$, we define a set $S^{(\ell ,h)}$ containing the top $t_{\mathrm{aa}}\%$ highest attention values between aa pairs. We used $t_{\mathrm{aa}}=2$, corresponding to the top 2% of aa attention values; this aa-level threshold is separate from the final protein-pair threshold *k=50*:


\begin{eqnarray*}
S^{(\ell ,h)} = \lbrace (p,q) \mid N^{(\ell ,h)}_{p,q} \,\gt\, \mathrm{Quantile}_{1 - \frac{t_{\mathrm{aa}}}{100}}(N^{(\ell ,h)})\rbrace .
\end{eqnarray*}

Attention values within the set *S* were subsequently converted to scores via min–max normalization. These normalized scores were then aggregated to compute an attention-based interaction score $A_{P_I,P_J}$ between protein pairs $P_I$ and $P_J$, having lengths $n_I$ and $n_J$, respectively:


\begin{eqnarray*}
A_{P_I,P_J} = \frac{\sum _{p \in P_I}\sum _{q \in P_J} \mathrm{score}(p,q)}{n_I \times n_J} \quad \mathrm{with} \quad (p,q) \in S^{(\ell ,h)}.
\end{eqnarray*}

Interaction scores were computed for all protein pairs within the genome. Preliminary analyses indicated that genomically adjacent proteins consistently yielded the highest attention scores (Supplementary Fig. S3), likely reflecting genuine biological interactions. Therefore, for constructing the sparse attention matrix used in subsequent model training, only the top 50 ranked scores from non-consecutive protein pairs were included as interacting proteins, with the remaining pairs masked. This threshold of 50 interactions represents a balance between computational efficiency and interaction coverage appropriate for the viral genome sizes analyzed (Supplementary Fig. S4). The threshold parameter can be adjusted based on genome sequence length and available computational resources.

To calibrate the number of protein pairs selected for sparse attention on biologically relevant data, we investigated interaction patterns among Family A DNA polymerase (PolA), ribonucleotide reductase (RNR), and helicase (HEL) (Supplementary Figs. S3 and S4, Supplementary Table S2). These proteins constitute a well-characterized interaction network [31] and display strong evolutionary coupling due to their coordinated roles in DNA replication [32, 33].

### Sparse attention computation (Stage 2)

**Figure 3:**
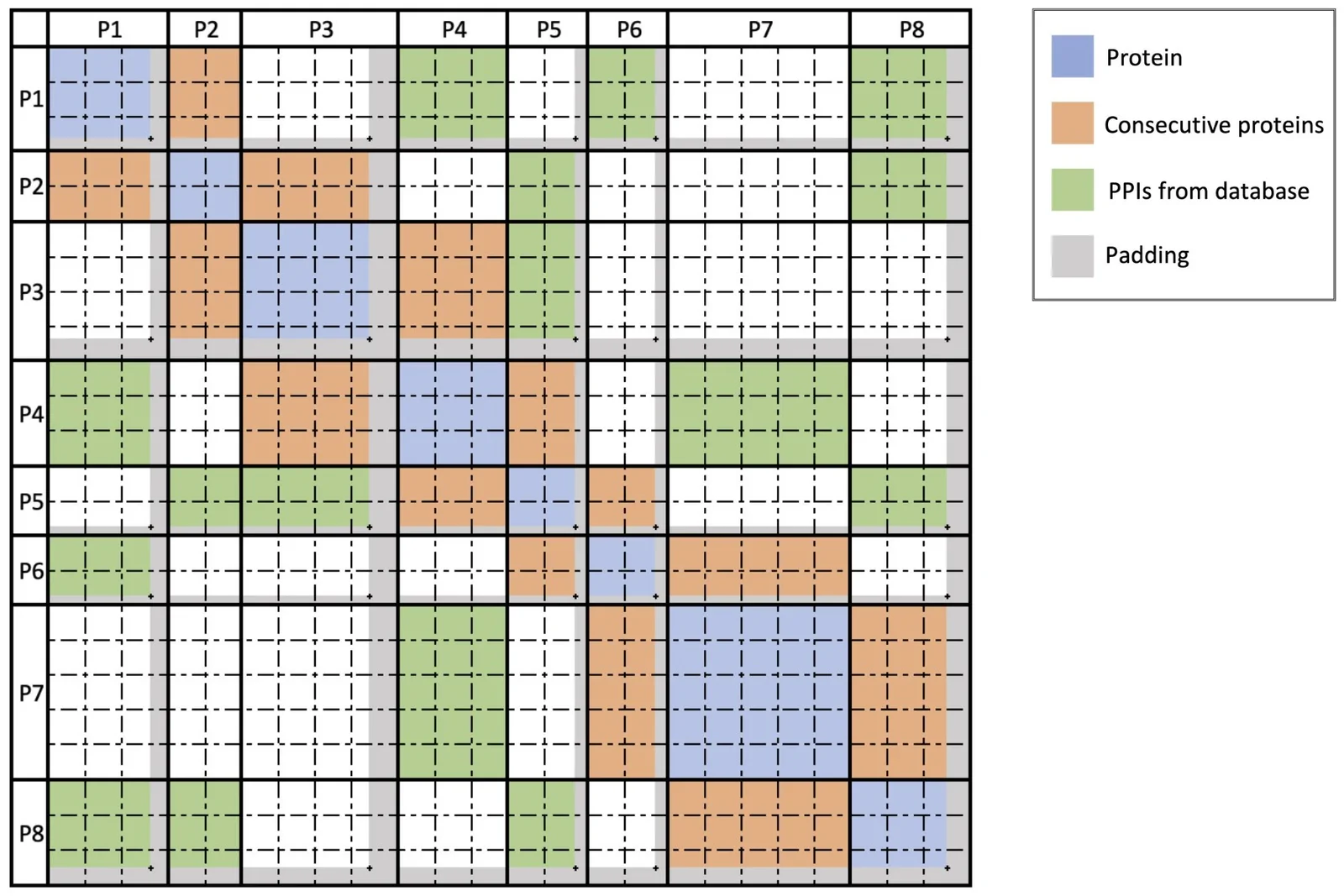
Sparse attention computation architecture. Example attention matrix demonstrating 4 distinct protein interaction patterns considered during sparse attention computation: intra-protein (self) attention (blue), interactions between consecutive proteins (orange), putative PPIs identified by our method (the “Developing the PPI inference model (Stage 1)” section) and stored in a database (green), and protein pairs not identified as interacting (white), for which attention calculations are omitted. Gray regions indicate padding introduced during fragment construction.

Once the PPIs have been identified and selected using the mechanism described above, it is possible to proceed with training a long-context pLM. Model training is based on a sparse attention mechanism, described below, and represented in Fig. 3.

To maintain biological fidelity when computing cross-protein attention, we developed a two-stage fragmentation approach. First, we segment the viral genome precisely at protein boundaries, preserving each protein as a distinct unit. Second, we subdivide each protein into uniform blocks of fixed length *B*, resulting in a block-level representation of the viral genome. We then map the previously identified PPIs onto these blocks, creating fragment-level interaction pairs that guide our sparse attention model. This transformation preserves biologically meaningful relationships while enabling efficient computation across the entire viral genome.

Specifically, given two interacting proteins $P_1$ and $P_2$, we transfer these interactions to interactions between their constituent blocks:


\begin{eqnarray*}
(P_1, P_2) \Rightarrow (B_{1_i}, B_{2_j}), \quad i \in [1,n_1], \,\, j \in [1,n_2],
\end{eqnarray*}

where

-
$P_1, P_2$ are two interacting proteins from genome *G*.
-
$B_{1,i}$ is the *i*th block of protein $P_1$, with $n_1$ total blocks.
-
$B_{2,j}$ is the *j*th block of protein $P_2$, with $n_2$ total blocks.

For each pair of blocks $(B_{1,i}, B_{2,j})$, we compute the block-level attention scores using the scaled dot-product attention formula:


\begin{eqnarray*}
Att_G(B_{1,i},B_{2,j}) = \frac{Q_{B_{1,i}} K_{B_{2,j}}^T}{\sqrt{d_k}},
\end{eqnarray*}

where

-
$B_{1,i}$ , $B_{2,j}$ are the blocks from genome *G* being considered.
-
$Q_{B_{1,i}}$ is the query vector subset from genome *G* corresponding to block $B_{1,i}$.
-
$K_{B_{2,j}}$ is the key vector subset from genome *G* corresponding to block $B_{2,j}$.
-
$d_k$ is the dimensionality of the key vectors.

Efficient computation of sparse attention requires specialized data structures optimized for block operations. We utilize the Block Sparse Row (BSR) tensor format [34], which stores non-zero elements in fixed-size blocks, significantly reducing both memory consumption and computational overhead. Empirical testing identified a block size *B = 32* as optimal, balancing computational efficiency and minimal padding overhead in our viral protein datasets.

In our implementation, each query–key product $Q_{F_i}(K_{F_j})^T$ between fragment pairs $B_{1,i}$ and $B_{2,j}$ produces a *32 × 32* tensor, directly aligning with BSR’s block structure. We subsequently apply softmax normalization followed by multiplication with the corresponding value vectors using optimized Triton [20] kernels tailored for block-sparse operations. Finally, we remove padding tokens to restore original sequence dimensions using precomputed position indices (Algorithm 1).

**Algorithm 1:** Algorithm for computing biologically informed sparse attention, from input tensors through block-wise operations to final output

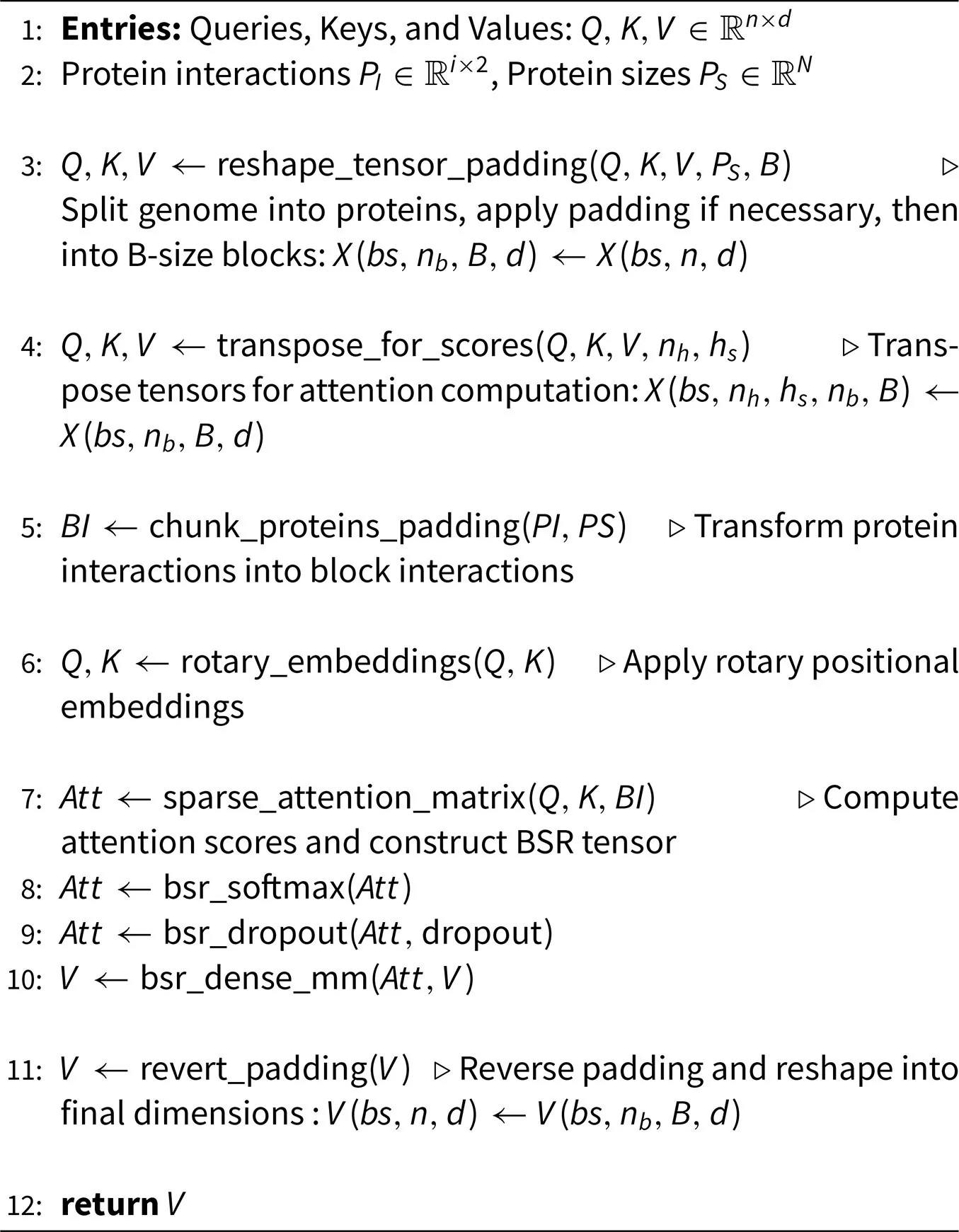

### Model training (Stage 2)

The training was conducted using the NCBI Virus database (retrieved on 06/2024), comprising 14,436 complete viral genomes totaling 683 million aa (Supplementary Fig. S1). The database represents a comprehensive reference set of viral diversity, ensuring robust generalization across viral families. All genomes were segmented at protein boundaries and padded appropriately to meet sequence length constraints. All training was conducted using NVIDIA A6000 GPUs (48 GB RAM) with block-sparse computations optimized through the Triton library [20].

The long-context pLMs represent two main variants, designed to produce complementary types of embeddings. These two models differ in scale, with LV-3C being the lighter version (317M parameters) and LV-5B the larger one (756M parameters).

The two models also differ in their initialization and training strategies:

- LV-3C: Randomly initialized and trained from scratch on genomic segments (up to 61,000 aa) with an initial learning rate of $3\times 10^{-4}$, using biologically informed sparse attention for 12 epochs.
- LV-5B: Trained leveraging transfer learning from the ESM-2 model (650M parameters) on genomic segments (up to 48,000 aa), using the same biologically informed sparse attention with an initial learning rate of $3\times 10^{-5}$, also for 12 epochs.

This two-model design aims to capture complementary aspects of viral genomic information. LV-3C prioritizes the modeling of long-range genomic context through a lighter architecture that enables the processing of extended sequences, whereas LV-5B prioritizes richer pretrained representations through a larger model operating on shorter segments.

### Evaluation metrics (Stage 3)

We employ two primary metrics throughout our evaluations:

**Perplexity** measures how well the model predicts amino acid sequences, quantifying the model’s uncertainty when predicting masked tokens. It is defined as the exponentiated average cross-entropy loss:


\begin{eqnarray*}
\mathrm{Perplexity} = \exp \left(-\frac{1}{N}\sum _{i=1}^{N}\log P(x_i | \mathrm{context})\right),
\end{eqnarray*}

where *N* is the number of masked tokens, and $P(x_i | \mathrm{context})$ is the predicted probability of the true amino acid $x_i$. Lower perplexity indicates better predictive performance. This metric is particularly important for assessing whether models maintain consistent prediction quality as sequence length increases.

**Silhouette score** evaluates embedding quality by measuring clustering coherence. For a given embedding *i* with cluster label *a*, the silhouette score is


\begin{eqnarray*}
s(i) = \frac{b(i) - a(i)}{\max (a(i), b(i))},
\end{eqnarray*}

where *a(i)* is the mean distance to other points in the same cluster, and *b(i)* is the mean distance to points in the nearest neighboring cluster. Scores range from −1 to 1, where higher values indicate better-defined clusters. We use this metric to assess whether embeddings group biologically related sequences (e.g., by sequence similarity or taxonomic classification).

### Evaluating global performance and protein embeddings quality

We evaluated our biologically informed sparse attention models (LV-3C and LV-5B) against ESM-2 pre-trained (ESM-2-pt), RoPE fine-tuned (ESM-2-RoPE), and ALiBi fine-tuned (ESM-2-ALiBi) variants using perplexity and clustering silhouette scores. Perplexity assessed token prediction accuracy over extended contexts, while the silhouette scores evaluated the quality and information content of protein sequence embeddings. We assessed model performance using viral genome fragments ranging from 500 to 20,000 aa. Although our models support sequences up to 61,000 aa, we limited evaluations to 20,000 aa to accommodate the computational constraints (time and memory) associated with full attention when running on a single GPU (NVIDIA A6000 with 48 GB of RAM).

#### Sequence-based clustering reference

To evaluate whether model embeddings preserve sequence relationships, we clustered proteins using MMseqs2 at 80% sequence identity. These clusters serve as reference labels for computing silhouette scores: high silhouette scores indicate that proteins similar in sequence are also proximal in embedding space. This provides a baseline assessment of embedding quality independent of functional or taxonomic annotations.

### Evaluating genome embeddings quality

We assessed the quality of genome-scale embeddings produced by our models using taxonomic clustering and species classification. To further evaluate embedding utility, we trained a single-layer classifier to identify species from genome embeddings (details provided in Supplementary Material S3).

### Evaluation of embedding quality for classification

We evaluated the models’ contextual embeddings by assessing their performance on species clustering and classification tasks (see also Supplementary Material S3; Pfam/domain-family validation is described separately in Supplementary Material S4), as well as by analyzing the similarity distributions obtained from comparing pairs of embeddings.

- **Evaluation of taxonomic information preservation:** To evaluate how effectively different models preserve taxonomic information, we analyzed clustering patterns of protein embeddings derived from 100 distinct viral species.
- **Analysis of species clustering efficiency:** Next, we trained a single-layer taxonomic classifier designed to predict the species identity of individual proteins directly from their embeddings. To systematically assess classification performance, we varied the number of species classes from 5 up to 100 species. We measured classifier performance using *F*1-score averaged over a 5-fold cross-validation scheme.
- **Analysis of embedding variance and distribution:** To further characterize the differences among embedding spaces produced by various models, we randomly selected 5,000 proteins from over 1,000 viral genomes and computed pairwise Euclidean distances between their embeddings. We then compared the resulting distributions from embeddings generated by ESM-2 variants and our 2 proposed models, LV-5B and LV-3C.

### Evaluating across-model attention

The following two sections evaluate the attention patterns of the trained LV-3C and LV-5B models, as distinct from the Stage 1 PPI inference used to build the sparse-attention priors: they assess whether the trained models—having been guided by these priors during training—retain and recapitulate biologically meaningful PPI signal in their final attention. To this end, we analyzed attention patterns across the entire network architecture. Attention scores from all heads in each layer were extracted to examine each model’s ability to detect and represent interactions spanning various distances within the genome sequence.

We categorized attention interactions into three distinct proximity classes:

- **Short-range interactions**: Attention between aa positioned within 100 residues of each other, typically representing intra-protein or intra-domain relationships.
- **Medium-range interactions**: Attention between aa separated by 100–500 residues, generally capturing interactions between domains or adjacent proteins.
- **Long-range interactions**: Attention between aa separated by more than 500 residues, predominantly representing inter-protein interactions between non-adjacent proteins.

For each category, we calculated the mean attention score across multiple viral genomes, enabling comprehensive analysis of the model’s attention distribution patterns.

### Validation against experimentally curated interactions

To validate the biological relevance of model-derived attention patterns, we benchmarked attention-derived protein-pair scores, interpreted as putative interaction links, against experimentally validated interactions documented in the STRING database [35]. Attention scores were extracted from all heads and layers, and protein pairs were ranked by their aggregate long-range attention score following [30]. Validation encompassed 146 annotated interactions across 5 distinct viral genomes. STRING interactions with confidence *\ge* 0.500 served as positive examples; negative examples consisted of all other protein pairs from the same genomes not reported as interacting in STRING. We note that this negative set likely contains some true interactions not yet experimentally characterized, making our reported metrics conservative estimates.

## Results

### Developing the PPI inference model (Stage 1)

Before training our genome-scale models LV-3C and LV-5B, we first developed ESM-2-PPI, a separate auxiliary model used exclusively to derive the PPI priors that guide the sparse attention mechanism. ESM-2-PPI extends pretrained ESM-2 (650M) to a 13,000 aa context window via LoRA fine-tuning, sparse attention, and ALiBi positional extrapolation. Because the choice of PE and sparse-attention pattern directly affects the reliability of the attention scores subsequently used as priors, we evaluated two PE strategies (RoPE, ALiBi) (Supplementary Fig. S2) and three sparse attention mechanisms (Longformer, Shifted Sparse Attention, BigBird) (Supplementary Table S1) on held-out viral genome fragments ranging from 500 to 13,000 aa, requiring both stable perplexity across extended sequence lengths and embeddings that preserve biological relationships. ALiBi with Longformer attention achieved the best balance of perplexity stability (1.99 across all tested lengths), clustering quality (silhouette score: 0.88), and computational efficiency.

To validate that ESM-2-PPI captures biologically meaningful interactions, we analyzed attention patterns among PolA, RNR, and HEL—proteins with well-characterized co-evolutionary relationships [31–33]. These known interacting pairs consistently ranked among the top 50 non-adjacent protein pairs across 83 viral genomes (Supplementary Figs S3 and S4), supporting our selection of *k=50* as the interaction threshold.

This validation serves only to calibrate the PPI selection threshold for ESM-2-PPI, and the PolA/RNR/HEL interactions were not used during training of LV-3C or LV-5B, which learn from PPIs inferred independently across all genomes in the training set.

### Evaluating global performance and embeddings quality

This section evaluates our two genome-scale pLMs LV-3C (317M parameters, trained from scratch on sequences up to 61,000 aa) and LV-5B (756M parameters, initialized from ESM-2 weights and trained on sequences up to 48,000 aa) against three ESM-2 baselines (ESM-2-pt, ESM-2-RoPE, ESM-2-ALiBi). Both LV models share the same biologically informed sparse attention mechanism and differ only in scale and initialization, allowing us to disentangle the contribution of context length from that of pretrained knowledge.

**Figure 4:**
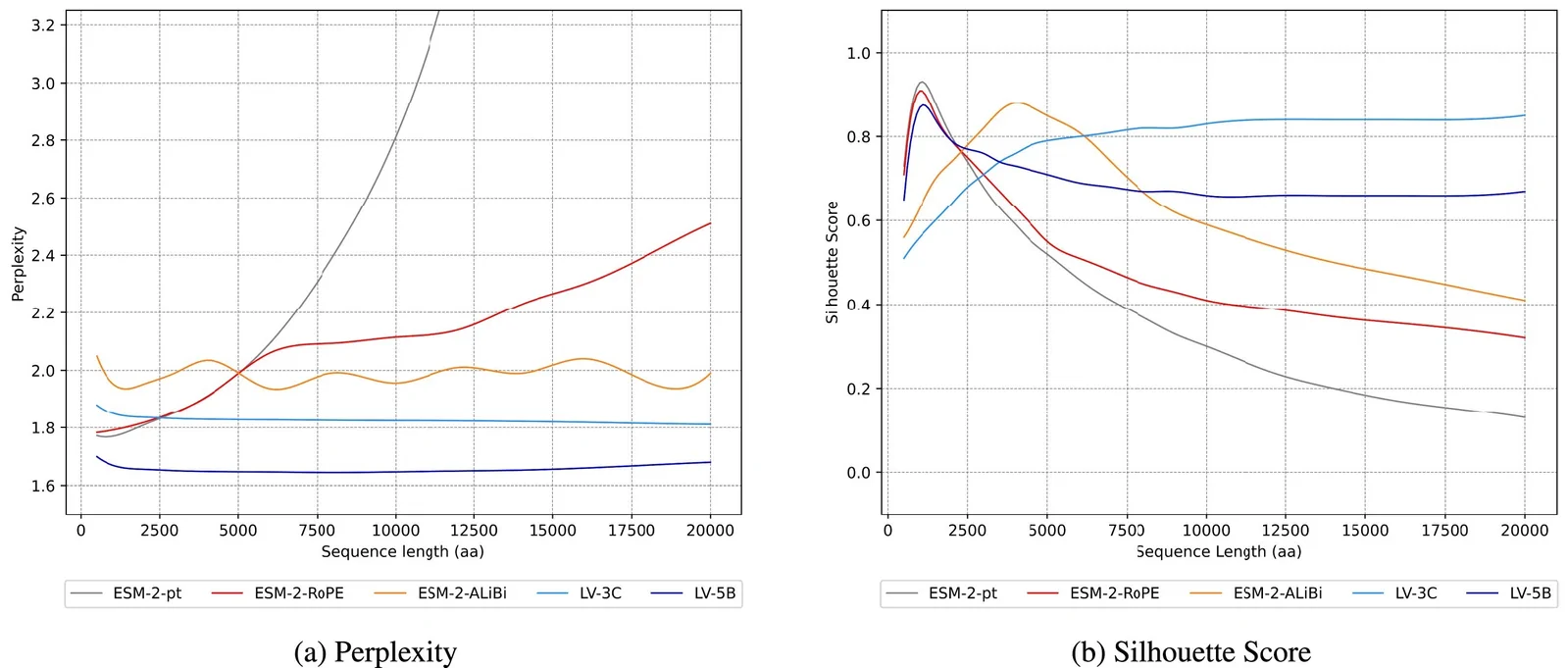
Global performances of our models compared to ESM-2 variant baselines. (a) Perplexity evaluation of ESM-2-pt, ESM-2-RoPE, ESM-2-ALiBi, and our biologically informed sparse attention models LV-3C and LV-5B. (b) Clustering ability evaluation using silhouette score on fragment embeddings, using sequence similarity as reference with MMSeqs2 80% amino acid identity clustering. These smoothed curves are obtained from perplexity and silhouette score measurements for sequences ranging from 500 to 20,000 aa.

The LV-3C and LV-5B models demonstrated significantly lower perplexity (uncertainty when predicting masked tokens) compared to ESM-2 variants on sequences longer than 3,000 aa (Fig. 4a), maintaining this advantage consistently across all tested lengths. In contrast, both the pre-trained ESM-2-pt (gray) and its RoPE-fine-tuned variant ESM-2-RoPE (red) experienced significant perplexity inflation when evaluated beyond their original training context windows. Fine-tuned version of ESM-2-ALiBi (orange) maintains near-constant perplexity with sequence length, but higher than our models.

For clustering quality analysis, we clustered protein sequences and fragments using MMseqs2 [36] at an 80% similarity threshold and computed silhouette scores to quantify embedding coherence. Our results revealed clear differences in clustering performance among the tested models (Fig. 4b).

Both LV models provided superior embedding coherence and better discrimination of protein sequences compared to baseline approaches. LV-3C (light blue) exhibited robust clustering quality, particularly for longer sequences (silhouette score: 0.83), while LV-5B (dark blue) achieved performance comparable to ESM-2-pt and ESM-2-RoPE for shorter sequences and maintained high effectiveness at extended lengths (silhouette score: 0.67). ESM-2-pt (gray) excelled only within its original training range (1,024 aa), with embedding quality markedly declining for longer, multi-protein sequences. While ESM-2-ALiBi (orange) extended effective clustering performance up to approximately 4,000 aa, embedding quality still deteriorated as sequence lengths increased, despite the PE extrapolation strategy.

### Evaluating genome embeddings quality

**Figure 5:**
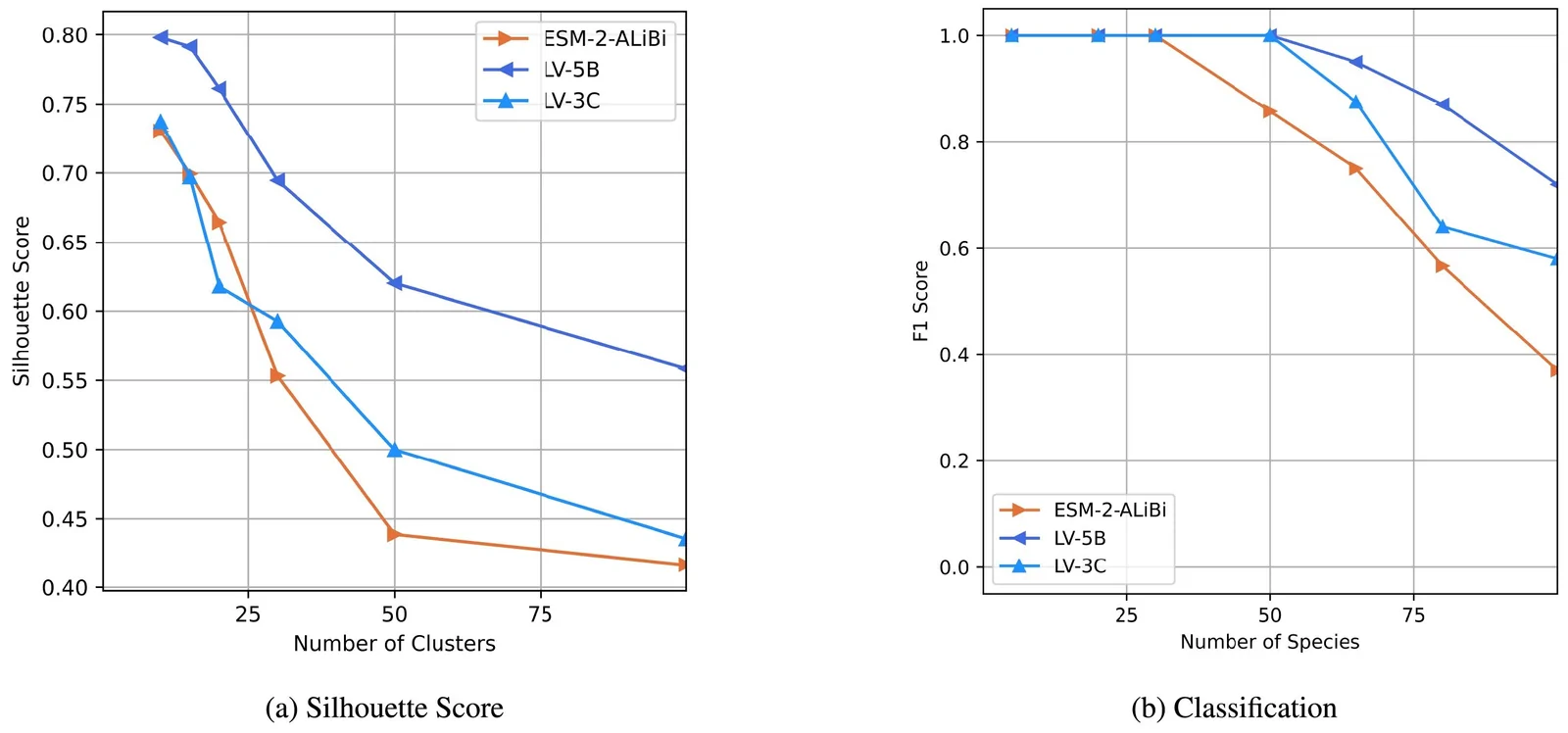
Evaluation of full genome embeddings. (a) Silhouette scores comparing genome embeddings generated by LV-3C, LV-5B, and ESM-2-ALiBi, using taxonomic species as cluster labels. (b) *F*1-scores for a single-layer classifier predicting species identity from genome embeddings (LV-3C, LV-5B, ESM-2-ALiBi). The *x*-axis varies the number of viral species used as classification targets, from 5 to 100; larger values denote finer-grained taxonomic resolution.

The quality of the genomic embeddings was assessed by downstream performance in a clustering and a classification task. We employ two complementary evaluations: silhouette scores assess embedding coherence, whether proteins from the same taxonomic species cluster together in latent space, while classification accuracy assesses whether embeddings encode sufficient discriminative information for species prediction. These metrics capture related but distinct properties: coherent embeddings facilitate classification, but similar classification performance can arise from different embedding geometries. Silhouette scores (Fig. 5a) indicated strong taxonomic discrimination across all models, with the LV-5B variant consistently outperforming others.

The performance of the species classification of genomes evaluated by *F*1-scores (Fig. 5b) revealed a notable trend: as the number of species increased—and consequently, task complexity—embeddings generated using biologically informed sparse attention demonstrated superior classification accuracy relative to genome embeddings from fine-tuned ESM-2-ALiBi. This performance improvement at higher taxonomic resolution suggests our embeddings effectively capture nuanced genomic features, enhancing discriminative power at larger phylogenetic scales. These properties hold promising implications for phylogenetic and metagenomic applications.

### Evaluation of protein embedding quality for classification

#### Analysis of embedding variance and distribution

**Figure 6:**
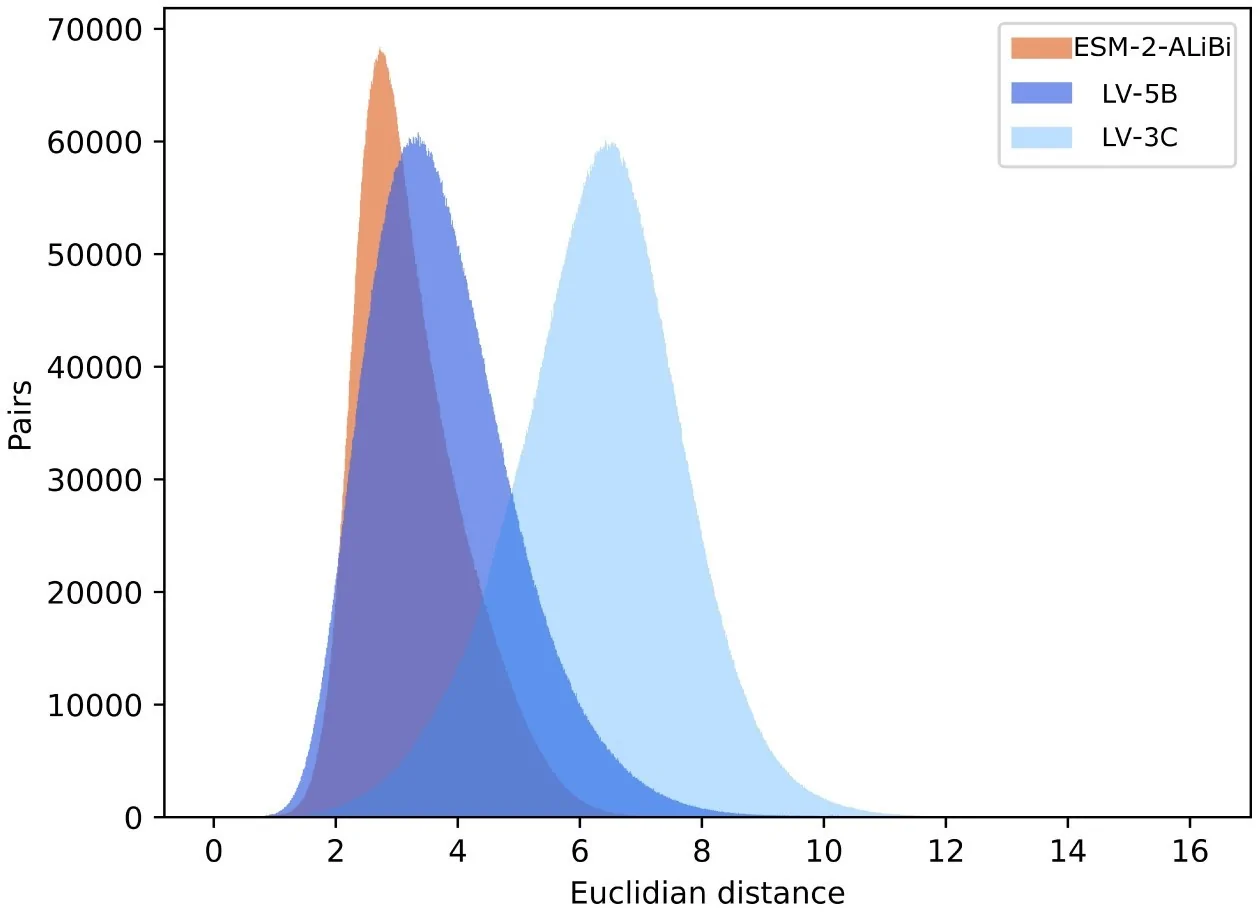
Protein embeddings pairwise Euclidean distance. Each curve summarizes *≈*12.5 million pair comparisons: lower distances (left tail) indicate within-species similarity, while greater distances reflect increasing taxonomic separation. ESM-2-ALiBi yields the most compact manifold, LV-5B broadens the range, and LV-3C further widens and right-shifts the distribution, demonstrating its larger dynamic range for distinguishing distantly related proteins without sacrificing tight clustering of closely related sequences.

The quality of protein embeddings generated by ESM-2-ALiBi and proposed models LV-3C and LV-5B was assessed based on embedding variance and distribution of the pairwise Euclidean distances between 5,000 randomly selected proteins from over 1,000 viral genomes. The analysis (Fig. 6) revealed distinct variability patterns among these embedding spaces. Embeddings produced by ESM-2-ALiBi demonstrated the narrowest distribution, with the lowest interquartile range (1.16) and a relatively limited embedding space extent (range: 9.43). In contrast, embeddings from model LV-5B showed greater variability, with a broader interquartile range (1.58) and a larger embedding space (range: 15.69). Model LV-3C exhibited the most dispersed embeddings, spanning the widest embedding space (range: 16.18) and displaying significantly higher average pairwise Euclidean distances. These results indicate that incorporating genomic context into LV-3C substantially diversifies embedding representations, likely capturing biologically meaningful, genome-specific information.

**Figure 7:**
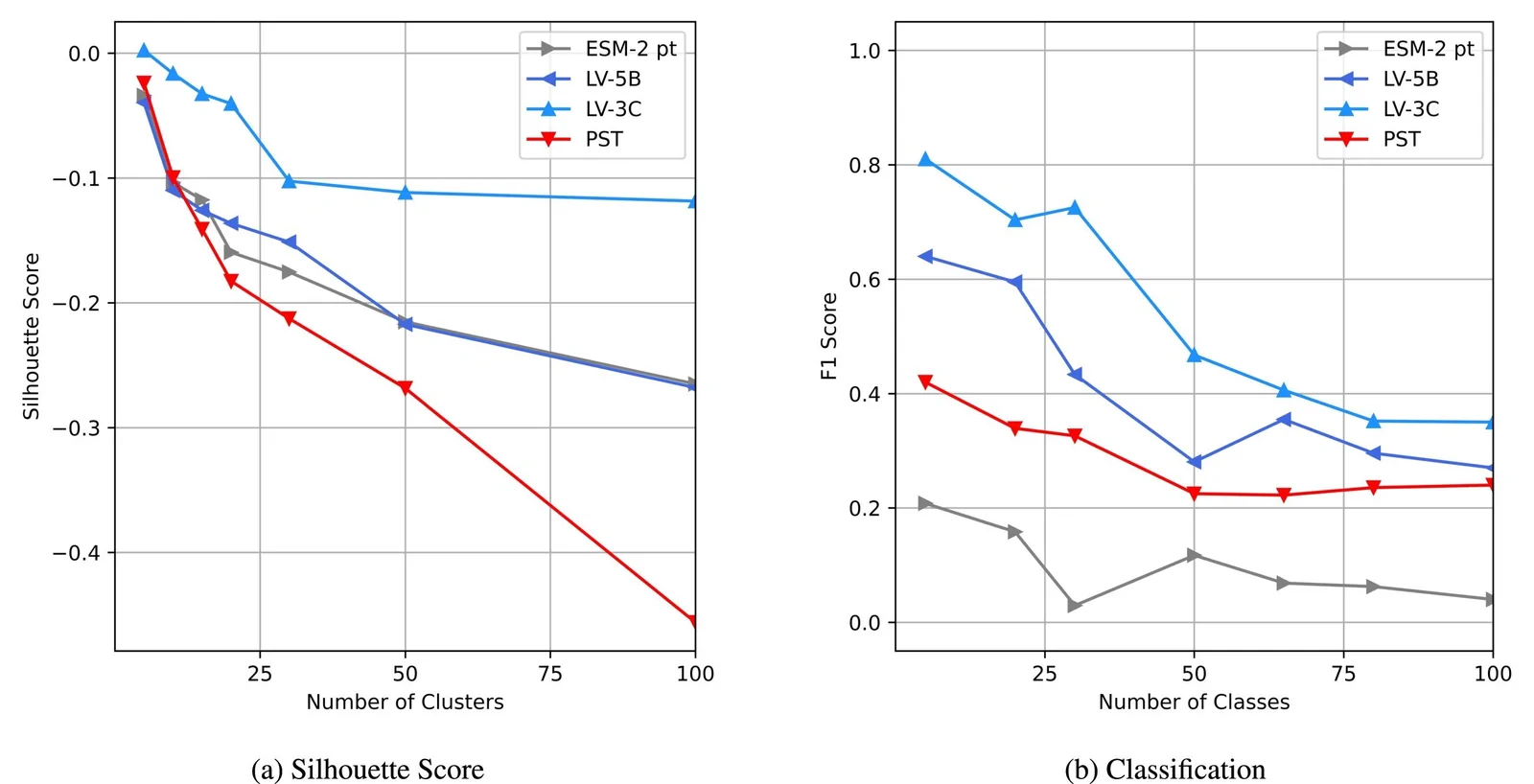
Evaluation of contextualized protein embeddings. (a) Silhouette scores comparing protein embeddings generated by LV-3C, LV-5B, PST, and ESM-2-pt, using taxonomic species as cluster labels. (b) *F*1-scores for a single-layer classifier predicting species identity from protein embeddings (LV-3C, LV-5B, PST, ESM-2-pt). The *x*-axis varies the number of viral species used as classification targets, from 5 (a coarse 5-way task) to 100 (a fine-grained 100-way task); larger values denote harder, more taxonomically resolved classification.

Further analysis of the Euclidean distance across protein embeddings shows that context provides a stronger correlation with taxonomic distance (Figs 6 and 7). Specifically, embeddings from proteins within the same species exhibit high similarity, characterized by low Euclidean distance. As the taxonomic distance between proteins increases, embedding similarity correspondingly decreases, highlighting the models’ ability to capture biologically meaningful divergence across broader taxonomic categories.

#### Evaluation of taxonomic information preservation

Given that protein embeddings inherently cluster primarily by protein type rather than species, silhouette scores based on taxonomy labels across all models were generally low (slightly negative). Despite this challenge, embeddings generated by the LV-3C model exhibited better species-specific clustering relative to those produced by ESM-2-pt and LV-5B (Fig. 7a, Pfam-specific clustering in Supplementary Material S4 and Supplementary Fig. S5). For comparison, we included embeddings from the PST, a recent genome-level protein model, as an additional baseline. We note that PST’s public release (v2.1.0) does not support genome-level embeddings, and its graph neural network architecture does not produce attention scores comparable to our transformer-based approach; PST is therefore included only in protein embedding evaluations where outputs are directly comparable. PST achieved the lowest silhouette scores, indicating limited capability in capturing viral species-level taxonomic relationships.

#### Analysis of species clustering efficiency

As illustrated in Fig. 7b, classification performance was the lowest when utilizing context-independent embeddings generated by the pre-trained ESM-2-pt. PST embeddings improved classification outcomes slightly, affirming that PST does capture genome-level context to some degree. However, LV-3C and LV-5B embeddings enriched with extensive genomic context further enhanced classifier accuracy across all evaluated class sizes. Among the tested models, LV-3C consistently achieved superior performance, reflected in the highest *F*1-scores for every class configuration.

### Evaluating across-model attention

Our proposed models were evaluated based on mean attention scores in short-range (within 100 aa, representing intra-protein or intra-domain relationships), mid-range (within 100–500 aa, representing inter-domain or adjacent protein relationships), and long-range ( *>* 500 aa apart, representing inter-protein interactions between non-adjacent proteins) interactions. The distribution of normalized attention scores across the three ranges provides insight into the model’s capacity to capture biologically relevant interactions at different genomic scales. High variance in the attention score distribution indicates preferential focus on specific interaction ranges, while more uniform distributions suggest balanced attention across all distance categories. This analysis is particularly valuable for assessing whether the model effectively captures the long-range dependencies that conventional pLMs typically miss due to context limitations.

**Figure 8:**
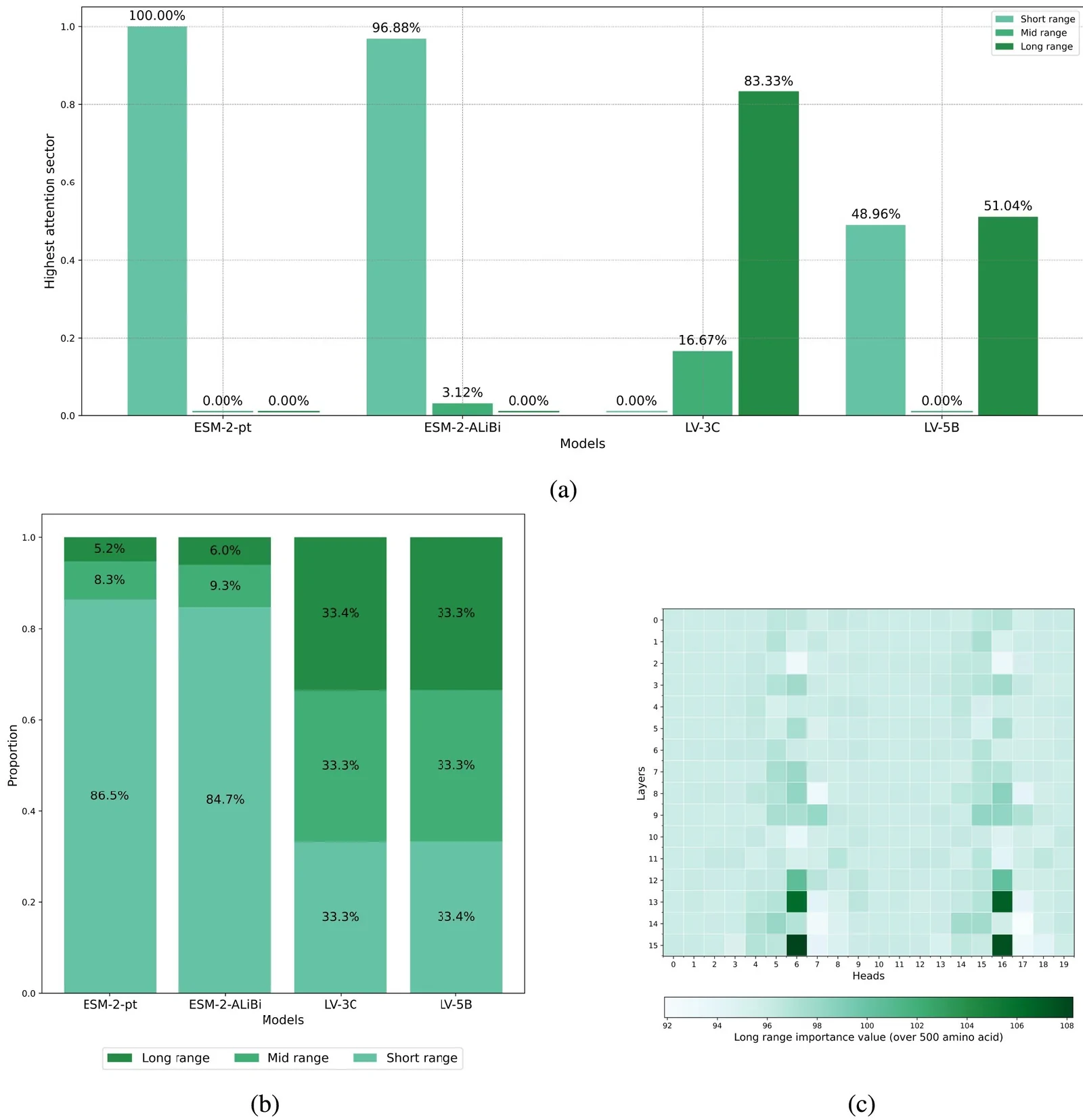
Attention analysis of our models. (a) Prevalence of dominant short-range (*łe*100 aa), medium-range (100–500 aa), and long-range (*>*500 aa) sectors across viral genomes. The chart represents 1,000 randomly selected viral genomes (*łe*13,000 aa in length), showing the proportion of genomes in which each interaction range category exhibited the highest mean attention score. (b) Distribution of normalized attention scores across interaction distance categories in viral proteomes. Stacked bar charts represent the distribution of attention scores for each interaction range category across 1,000 viral genomes. (c) Fine-grained analysis of long-range attention patterns in the LV-3C model. Darker (green) blocks indicate attention heads with a predominant long-range attention. The visualization demonstrates how attention mechanisms capture biologically significant inter-protein interactions spanning substantial genomic distances within viral proteomes. Distribution variance indicates the relative focus of attention heads on specific interaction distances.

The attention profiles (short-, medium-, and long-distance) differ substantially among the four models evaluated (Fig. 8a). The pre-trained ESM-2-pt model exhibits a highly localized attention mechanism, concentrating almost exclusively on short-range interactions (100%), typically within individual proteins or immediate neighboring residues. This pattern aligns with ESM-2’s training paradigm, which focused on single-protein contexts and thus developed representations primarily capturing local residue relationships.

In contrast, the ESM-2-ALiBi fine-tuned model, which employs a sliding-window (LongFormer) attention approach, demonstrates a modest expansion in attention scope. While it identifies a small fraction of medium-distance interactions (3.12%), it still predominantly focuses on short-distance interactions (96.88%). These findings suggest that although the sliding-window methodology enables detection of certain medium-range relationships, the model largely remains constrained to interactions between proximal aa.

Our models, LV-3C and LV-5B, exhibit markedly different attention distributions. Both models display balanced average attention scores across all distance categories (short, medium, and long), indicating a comprehensive approach to protein sequence modeling that equally integrates local and global contextual information. However, important distinctions between the two models emerge:

- Model LV-3C most frequently attributes maximal attention to long-distance interactions. This behavior highlights its enhanced capability to capture meaningful relationships between aa widely separated in sequence space, potentially spanning different proteins within the viral genome.
- Model LV-5B, while maintaining generally balanced average scores, frequently assigns the highest attention importance to both short- and long-distance interactions. This indicates a flexible modeling strategy capable of emphasizing both local structural information and global protein–protein relationships as needed. Notably, LV-5B’s attention distribution retains traces of its ESM-2-pt initialization, suggesting it effectively combines characteristics of the pre-trained model with adaptations necessary for the expanded genomic context.

To further investigate inter-protein interactions, we calculated attention scores and identified attention heads specifically enriched for long-range information, following the methodology described in the “Validation against experimentally curated interactions” section.

**Figure 9:**
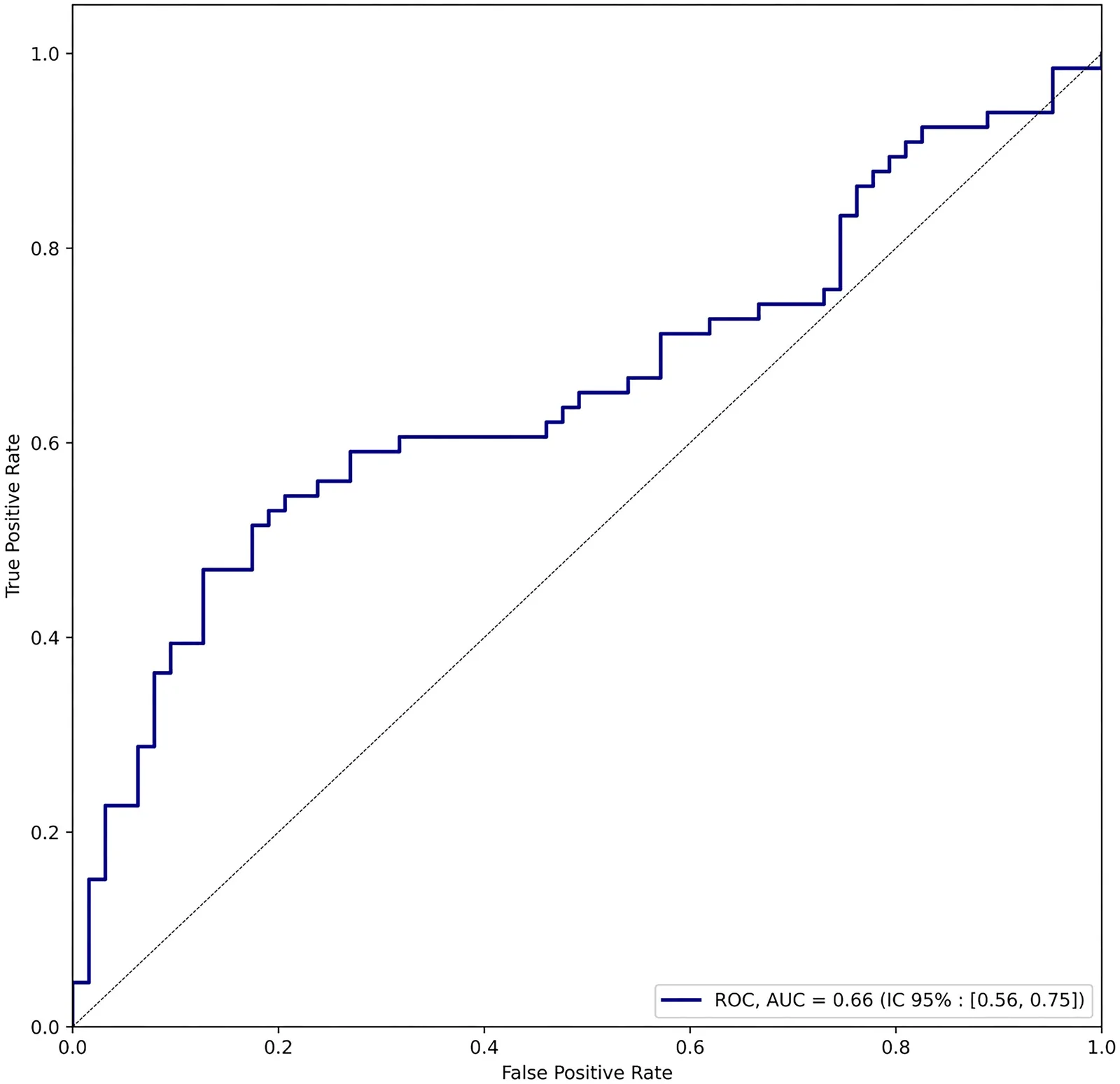
Receiver operating characteristic (ROC) curve of the binary classification task. ROC curve for distinguishing STRING-supported protein pairs from non-reported protein pairs using attention-derived pair scores. The curve shows LV-3C’s ability to distinguish experimentally validated interactions (STRING confidence *\ge* 0.500) from non-interacting protein pairs across five viral genomes (146 positive interactions). The AUC of 0.66, with a 95% bootstrapped confidence interval of [0.56, 0.75], indicates statistically significant predictive performance.

In a binary classification framework, using a STRING interaction confidence threshold of 0.500, our LV-3C model achieved an average *F*1 score of 0.74 and accuracy of 0.88. The classifier demonstrated an Area Under the Curve (AUC) of 0.66 (Fig. 9, with a bootstrapped 95% confidence interval of [0.56, 0.75]), indicating statistically significant predictive performance. The difference between *F*1 (0.74) and AUC (0.66) reflects that *F*1 is measured at an optimized decision threshold, while AUC captures ranking performance across all thresholds—a more challenging metric given the inherent noise in attention scores and the known incompleteness of viral protein interaction databases. These results show significant concordance between our attention-based interaction scores and experimentally validated interactions in the STRING database, indicating that the method enriches for putative protein interactions within viral genomes. Furthermore, attention-derived scores showed significant positive correlation with STRING confidence scores (Spearman *ρ = 0.33*, *p < 0.001*), providing additional evidence for the biological relevance of the learned attention patterns.

## Discussion

We note that attention-derived inter-protein links should be interpreted as computational priors rather than direct evidence of physical PPIs. For this reason, our evaluation strategy focused on language-model metrics, taxonomy/Pfam-aligned downstream tasks, and concordance with independent interaction resources (STRING), rather than claiming direct prediction of physical interactions.

Our findings demonstrate that extending transformer-based language models to encompass entire viral proteomes yields substantial improvements in both local amino acid prediction and broader protein function inference. By designing a biologically informed and content-aware sparse attention mechanism guided by inter-protein co-evolutionary signals, we have addressed the long-standing challenge of modeling densely packed viral genomes while maintaining computational tractability.

A central insight from our study is that higher-order genomic context—the collective presence and arrangement of multiple proteins—significantly enhances protein representation accuracy beyond what can be achieved by analyzing protein sequences in isolation [3], or using context length extension [16, 17] or context-agnostic sparse attention [21–23]. As the results show, conventional pLMs trained on individual proteins (e.g., ESM-2) focus on local sequence features and exhibit marked performance degradation when context extends beyond their trained window. In contrast, our biologically informed sparse attention approach captures both local relationships and long-range dependencies across entire viral genomes. This more comprehensive perspective reveals functional couplings between non-consecutive proteins that would otherwise remain undetected [31–33]. To confirm that this advantage arises from the inference step rather than from simply adding long-range links, we trained otherwise identical models given inferred, random, distance-matched, or no long-range links. Only the inferred links lowered held-out perplexity, and the improvement concentrated on the protein pairs the inference model scored most highly (Supplementary Material S6).

The attention patterns learned by our models show statistically significant concordance with experimentally validated PPIs, benchmarked against the STRING database [35]. The balanced distribution of attention across short, medium, and long-range interactions in our LV-3C and LV-5B models, in contrast to the predominantly local focus of the ESM-2 baselines, further reinforces the biological relevance of our approach.

The significant divergence in embedding spaces between context-independent (other models) and the context-aware LV-3C and LV-5B models, as evidenced by the distinct distributions of pairwise Euclidean distances between protein embeddings, underscores the fundamental difference in information captured by these approaches. The wider embedding space of our LV-3C model, with its greater interquartile range and broader distribution of distances, suggests a richer representation that incorporates both sequence-specific and context-dependent information.

To assess generalization across viral diversity, we evaluated model performance specifically on RNA viruses, which represent the most abundant and challenging subgroup in our dataset (Supplementary Material S5). Despite their higher mutation rates and structural complexity, our models consistently outperformed baselines (Supplementary Fig. S6), suggesting robust performance across viral groups.

To assess sensitivity to our 90% deduplication threshold, we additionally clustered at 65% identity, yielding 246,737 sequences—indicating that 71% of sequences retained at 90% clustering have no other sequence sharing >65% identity. This analysis suggests that the majority of our evaluation data consists of substantially divergent sequences, not near-duplicates. The impact of sequence similarity also varies by task: perplexity is most sensitive to train/test similarity, while taxonomy and species classification are less affected since these tasks require learning species-level patterns, making within-species similarity a desired signal rather than a confounder. Critically, all models were evaluated under identical conditions, ensuring valid relative comparisons.

To validate that our embeddings capture functional information beyond taxonomy, we evaluated performance using Pfam protein domain annotations (Supplementary Fig. S5). While silhouette scores are low, reflecting that genomic context, not domain identity, is the dominant organizing principle—classification performance remains strong, with LV-3C and LV-5B outperforming baselines. This confirms that domain information is encoded within our embeddings alongside the contextual signals that distinguish proteins with identical domains in different genomic environments.

Despite these advances, computational overhead remains a practical consideration. Our selection of a maximum of 50 PPIs for the sparse attention mechanism strikes an effective balance for viral datasets while maintaining computational efficiency. This parameter can be readily increased if GPU resources are available. We intentionally constrained our implementation to run on a single GPU, as we believe this represents a widely accessible computational environment for most research groups. While we utilized an NVIDIA A6000 for this study, the rapid advancement of more affordable GPUs (sub-$5,000) with comparable memory capacities makes our approach increasingly accessible. For processing significantly larger genomes—particularly from eukaryotic organisms—additional optimizations may be beneficial. Additionally, our semi-supervised approach to inferring putative interactions could benefit from larger ground-truth databases or complementary evidence from proteomic or structural studies to further refine the biological relevance of the learned attention patterns.

Although our training data include human pathogen sequences, our models pose substantially lower biosecurity risk compared to generative genomic models such as Evo [37]. Both LV-3C and LV-5B are designed to produce embeddings for downstream analysis rather than to generate novel sequences. The masked language modeling objective predicts individual positions given surrounding context, rather than generating complete sequences autoregressively. These distinctions substantially limit the utility of our models for generating functional novel proteins or genomes.

Looking forward, our framework offers considerable promise for extension to larger viral genomes, bacteriophages, and potentially even eukaryotic genomes with appropriate scaling. The methodology could be particularly valuable for analyzing metagenomic data, where contextual information might help resolve ambiguities in protein function assignment. Furthermore, the correlation between embedding similarity and taxonomic distance suggests potential applications in phylogenetic analysis and evolutionary studies.

## Conclusion

Our study proposes a novel framework for viral protein language modeling by extending transformer architectures to process entire viral genomes. By integrating a biologically informed sparse attention mechanism, our approach efficiently captures putative long-range inter-protein interactions that are missed by conventional single-protein models. This design not only reduces perplexity across extended sequences (up to 61,000 aa) but also generates context-aware embeddings that accurately recapitulate known co-evolutionary relationships—for instance, among PolA, RNR, and helicase.

Our results demonstrate that large-scale learning improves embedding quality. The enhanced clustering of protein and genome embeddings, along with robust taxonomic classification, underscores the broader applicability of our method to phylogenetic analysis and metagenomic studies. Our work provides a solid foundation for working with viral genomes and paves the way for more refined analyses of PPIs, ultimately deepening our understanding of viral biology and evolution. Looking ahead, our framework offers considerable promise for extension to larger genomes, and potentially even eukaryotic genomes. By refining the biological specificity of sparse attention patterns and incorporating additional layers of experimental validation, our approach can continue to evolve toward more precise and comprehensive genomic analyses. Ultimately, this work not only deepens our understanding of viral genome organization and evolution but also provides a scalable framework for protein modeling at genomic scales, bridging the gap between computational biology and experimental validation.

## Availability of source code and requirements

- Project name: ViralEmbed.
- Project home page: https://github.com/Hawaii-Bioinformatics/ViralEmbed.
- Operating system(s): Linux and macOS.
- Programming language: Python (*\ge* 3.9).
- Other requirements: PyTorch (*\ge* 1.8), fair-esm, transformers, Biopython; a CUDA-capable GPU with *\ge* 16 GB VRAM is recommended for processing long genomes.
- License: MIT.
- RRID: SCR_027596.

## Additional files

**Supplementary Figure S1**: Viral genome length distributions in the NCBI Virus Database. This histogram shows that a context length of 61k amino acids allows us to process over 98% of the 14,436 complete genomes of the database.

**Supplementary Figure S2**: Perplexity evaluation of positional encoding methods for fine-tuning. Performance metrics for ESM-2-pt and fine-tuned variants using Sliding Window (SW) attention mechanism with RoPE and ALiBi positional encoding. Perplexity values represent mean across sequences ranging from 500 to 13,000 amino acids.

**Supplementary Figure S3**: Distribution of interaction pair rankings of Family A DNA polymerase (PolA), ribonucleotide reductase (RNR), and helicase (HEL) across 83 viral genomes. Violin plots represent the ranking positions of PolA-HEL, PolA-RNR, and RNR-HEL pairs (total *n* = 249). Left panel: rankings among all protein pairs within fragments. Right panel: rankings after excluding genomically adjacent protein pairs.

**Supplementary Figure S4**: Selection of interaction threshold. PolA-HEL, PolA-RNR, and RNR-HEL pairs shown as a function of total genomic pairs and relative genomic rank. Dashed curves denote selection thresholds; pairs below each curve are retained for attention calculations.

**Supplementary Figure S5**: Evaluation of embeddings using Pfam domain annotations. (a) Silhouette scores using Pfam families as cluster labels. Negative scores across all models reflect that genomic context—not domain identity alone—is the primary organizing principle of the embedding space. (b) *F*1-scores for Pfam family classification. Despite low clustering coherence, LV-3C and LV-5B achieve strong classification performance, indicating that domain information is encoded but not dominant.

**Supplementary Figure S6**: Model performance on RNA viruses compared to all viruses. (a) Silhouette scores and (b) *F*1-scores for taxonomic species classification, comparing LV-3C, LV-5B, PST, and ESM-2-pt. Solid lines: RNA viruses only; lighter lines: all viruses. Despite the greater complexity of RNA viruses, our models maintain consistent improvements over baselines.

**Supplementary Table S1**: Performance metrics of ESM-2-pt and fine-tuned versions using different sparse attention patterns. Longformer refers to the Sliding Window Attention pattern from [21], $S^2$-Attn to the Shifted Sparse Attention from [22], and BigBird to the eponymous attention pattern from [23]. “+full” means normalization and embedding layers are trainable during low-rank fine-tuning. Metrics are calculated on genome fragments ranging between 10,000 and 13,000 amino acids.

**Supplementary Table S2**: Percentage of known interacting pairs (PolA-RNR-HEL) captured at different selection thresholds.

**Supplementary Table S3**: Held-out perplexity for the interaction-inference controls. Lower is better. Only the inferred links lower perplexity relative to having no long-range links.

**Supplementary Table S4**: Enrichment of improved positions on high-scoring inferred pairs. The most improved positions fall disproportionately on the proteins the inference model ranks most highly.

## List of abbreviations

aa: amino acid(s); ALiBi: Attention with Linear Biases; APC: Average Product Correction; AUC: Area Under the Curve; BSR: Block Sparse Row; CD-HIT: Cluster Database at High Identity with Tolerance; CRediT: Contributor Roles Taxonomy; DOME: Data, Optimization, Model, Evaluation; ESM: Evolutionary Scale Modeling; gLM: genomic language model; GPU: Graphics Processing Unit; HEL: helicase; LLM: large language model; LoRA: Low-Rank Adaptation; MMseqs2: Many-against-Many sequence searching 2; NCBI: National Center for Biotechnology Information; PE: positional encoding; Pfam: Protein family database; pLM: protein language model; PolA: Family A DNA polymerase; PPI: protein–protein interaction; PST: Protein Set Transformer; RNR: ribonucleotide reductase; RoPE: Rotary Position Embedding; ROC: Receiver Operating Characteristic; RRID: Research Resource Identifier; STRING: Search Tool for the Retrieval of Interacting Genes/Proteins; VRAM: Video Random Access Memory.

## Supplementary Material

giag081_Supplemental_File

giag081_Authors_Response_To_Reviewer_Comments_Original_Submission

giag081_Authors_Response_To_Reviewer_Comments_revision_1

giag081_GIGA-D-25-00436_original_submission

giag081_GIGA-D-25-00436_revision_1

giag081_GIGA-D-25-00436_revision_2

giag081_Reviewer_1_Report_Original_Submission Reviewer 1 -- 12/11/2025

giag081_Reviewer_1_Report_Revision_1 Reviewer 1 -- 5/5/2026

giag081_Reviewer_2_Report_Original_Submission Reviewer 2 -- 1/4/2026

giag081_Reviewer_2_Report_Revision_1 Reviewer 2 -- 3/30/2026

## Data Availability

Machine learning annotations have been deposited in the DOME registry [38]. All additional supporting data [39] are available in the *GigaScience* repository, GigaDB [40].
