# Supplementary material for: Extending protein language models to a viral genomic scale using biologically induced sparse attention
Source: Gigascience. 2026 Jul 21;15:giag081. doi: 10.1093/gigascience/giag081 (PMC13495794; doi:10.1093/gigascience/giag081)
Supplement: Supplementary file 1 [file giag081_authors_response_to_reviewer_comments_original_submission.pdf]

Author's Response To Reviewer Comments

Responses to reviewers:

Reviewer #1
>>>>>>>>>>>>>>>>>>
The paper by Dejean, et. al. describes a novel approach, and set of models, to extend protein language models to a much larger context window (60,000 amino acids) using a combination of approaches to extending the context window including biologically-informed attention mechanisms. The approach is interesting, and potentially very useful. The authors apply it to viral sequences, and show in a variety of ways, improvements over existing models.

The manuscript could be improved in several ways detailed below:
<<<<<<<<<<<<<<<<<<
We thank the reviewer for their very thorough and constructive evaluation of our manuscript. We are extremely pleased that the reviewer finds our approach interesting and potentially very useful.
We greatly appreciate the depth and rigor of the comments provided. Each point raised was insightful and helped us identify areas where the manuscript lacked clarity. We have addressed every comment raised by implementing the suggested changes and ensuring that previously confusing sections are now clearly explained. We believe the revised manuscript is substantially improved as a result of this careful review.
Below we provide point-by-point responses to each comment.

>>>>>>>>>>>>>>>>>>
1. The authors of the evo paper (which is referenced in the current paper), a DNA language model with a large context, thought it necessary to specifically exclude eukaryotic and thus potential human pathogen viral sequences from their training because of the potential safety issues around their generative model. It is A) not clear if any similar filtering was done in the current study (it is not mentioned, so I assume not), B) not clear if this is *as* necessary as it may be with the evo model - that is, do you consider your models to be usefully generative? At the very least some discussion of this important consideration should be mentioned in the paper.
<<<<<<<<<<<<<<<<<<

We thank the reviewer for raising this important consideration. Our training dataset was not specifically filtered to exclude human pathogens. However, our models pose substantially lower biosecurity risk compared to Evo for several reasons. First, both models are designed to produce embeddings for downstream analysis, unlike Evo, which can generate complete novel sequences token-by-token. Second, masked language modeling predicts individual positions given surrounding context, rather than generating complete sequences autoregressively. These distinctions substantially limit our models' utility for generating functional novel proteins or genomes.
For these reasons, we do not consider our models to be usefully generative in the sense that would require the same filtering applied to Evo.

As suggested, we have added a paragraph to the Discussion section addressing these biosecurity considerations.
>>>>>>>>>>>>>>>>>>
2. The different models that are developed and used in the paper are overall confusing. This is a significant issue with the paper since it makes it very difficult to figure out what results map to which models, how the pieces fit together, and the significance of the advances. My feeling is that reducing the number of different models and forms of different models being referred to in the main text (see point 3, below).
<<<<<<<<<<<<<<<<<<

We thank the reviewer for this feedback. After revisiting the text after some time, we can see exactly what's confusing the reviewer and we agree that the relationship between the different models was not sufficiently clear in the original manuscript. Our answers to each of the points raised by the reviewer are provided below.

>>>>>>>>>>>>>>>>>>
a. If I understand (and I may not), ESM is used in some form (fine tuned, I think) to serve as the first stage that predicts protein interactions, which are then fed into a second stage which (may be) the two models labelled LV-3C and LV-5B. ESM is used in two forms (maybe more?) - which seems to be the original form and a fine-tuned form which might have an extended context window and also might be fine-tuned on the same input sequence?
<<<<<<<<<<<<<<<<<<

That is correct. A modified version of ESM that was finetuned to handle long fragments is used to predict protein interactions. Specifically, our pipeline consists of three distinct stages, each using different models: Stage 1 (PPI Inference): ESM-2 is fine-tuned with ALiBi positional encoding, LoRA, and Longformer-style attention to extend its context window to ~13,000 aa. This model's sole purpose is to extract attention scores for inferring putative protein-protein interactions. It is not used for any downstream tasks.

Stage 2 The novel Genome-Scale Model we propose. Using the PPIs identified in Stage 1 as sparsity priors, we train two novel long-context protein language models:

LV-3C: Trained from scratch
LV-5B: Initialized from ESM-2 weights

These two models represent the primary contributions of this work.

Stage 3 Baseline comparisons: For evaluation, we compare LV-3C and LV-5B against several ESM-2 variants (pretrained, fine-tuned with RoPE, fine-tuned with ALiBi). These baselines were trained solely to provide fair comparisons and demonstrate the advantages of our biologically-informed sparse attention approach.

We have added a new "Training and Evaluation Pipeline Overview" subsection (Section 3.2) that explicitly describes these three stages, defines standardized model names (ESM-2-PPI, ESM-2-pt, ESM-2-RoPE, ESM-2-ALiBi, LV-3C, LV-5B), and clarifies the purpose of each model in the pipeline.

>>>>>>>>>>>>>>>>>>
b. The first part of the results section, which talks about predicting protein interactions seems to describe this fine-tuning. But it's not clear. There are two positional encoding and 3 sparse encoding strategies tested (which is fine) but it makes it really hard to figure out what is then used, and why. The motivation for this section, and the choices for the models needs to be made clear. Also, there seem to be variants of the fine-tuning that are used throughout? This makes things more confusing.
<<<<<<<<<<<<<<<<<<

Again, We agree that the motivation for the fine-tuning ablation study and the rationale for our final model choices were not sufficiently clear.

To clarify: the fine-tuning experiments in Section 4.1 pertain exclusively to the PPI Inference Model (Stage 1, now called ESM-2-PPI) as described in our response to comment 2.a. This model's sole purpose is extracting attention scores to identify putative protein-protein interactions—it is not used for any downstream tasks.

The PPI inference model must satisfy two requirements: (1) stable perplexity across extended sequences (10,000+ aa), and (2) high-quality embeddings that preserve biological relationships. We evaluated two positional encoding strategies (RoPE, ALiBi) and three sparse attention mechanisms (Longformer, Shifted Sparse Attention, BigBird) against these criteria. ALiBi + Longformer achieved the best balance of perplexity stability, embedding quality, and computational efficiency, and was therefore selected. The other

variants do not appear elsewhere in the manuscript.

We have (1) renamed Section 4.1 from "Selecting an Optimal Fine-tuning Strategy" to "Optimizing the PPI Inference Model (Stage 1)", (2) added an opening paragraph that explicitly states the motivation, evaluation criteria, and final selection before presenting detailed results, and (3) clarified that the fine-tuning variants in Table 1 are specific to Stage 1 only.

>>>>>>>>>>>>>>>>>>
c. The difference between the two main models, LV-3C and LV-5B are not clear. 3C is trained on genomic segments using 'sparse attention informed by protein-protein interactions' (are these from the first stage?) and 5B is trained on shorter segments - but using transfer learning from ESM-2 and using 'biologically-informed sparse attention'. How is 'biologically-informed' different than 'protein-protein interactions'? Much more clarity around the differences and motivation for each of these models is needed to be able to interpret the following results.
<<<<<<<<<<<<<<<<<<

We apologize for the confusion. To clarify: "sparse attention informed by protein-protein interactions" and "biologically-informed sparse attention" refer to the same mechanism. Both LV-3C and LV-5B use identical sparse attention patterns derived from the PPIs inferred in Stage 1.
The differences between LV-3C and LV-5B lie in two aspects: model scale and initialization strategy.
First, the models differ in architecture size. LV-3C is lighter (317M parameters, 16 layers) while LV-5B is larger (756M parameters, 33 layers). These architectural choices directly impact the sequence lengths that can be processed under the same GPU memory constraints: LV-3C handles longer genomic segments (up to 61,000 aa) whereas LV-5B processes shorter segments (up to 48,000 aa) but with greater representational capacity.
Second, the models differ in initialization. LV-5B is initialized from pretrained ESM-2 (650M) weights, providing a strong starting point from large-scale protein sequence data. LV-3C is trained from scratch with random initialization.
In summary, LV-3C prioritizes longer genomic context through a lighter architecture trained from scratch, whereas LV-5B prioritizes richer pretrained representations through a larger model operating on shorter segments. This design allows us to evaluate complementary strategies for incorporating genomic context into protein representations.
We have (1) standardized terminology throughout to use "biologically-informed sparse attention" consistently, and (2) expanded the Training and Evaluation Pipeline Overview section (Section 3.2) to explicitly describe the architectural differences, initialization strategies, and complementary design rationale for LV-3C and LV-5B.

>>>>>>>>>>>>>>>>>>
d.
i . ESM 'flavors' are used to compare - as baselines - for the other models, but since it's hard to follow what these ESM flavors are exactly it makes interpretation difficult. Also, the same ESM model(s) is used for the 'first stage' to predict interactions as to compare against the final 'second stage' models? This needs to be made clear.
Related, I liked the use of the PolA, RNR, and HEL to optimize the number of interactions needed. But then this same complex is used as an example in the final model. Which is confusing, and also, it's not entirely clear that it's not circular (I feel it's not, but this really needs some explanation and clarity) - that is, 'we used PolA, RNR, and HEL to optimize our stage 1, and we find that our stage 2 is really good at predicting these interactions'
<<<<<<<<<<<<<<<<<<

We thank the reviewer for raising this concern. Regarding point 1 (ESM model variants): the new Section 3.2 (Training and Evaluation Pipeline Overview) now clearly distinguishes ESM-2-PPI (used only for Stage 1 PPI inference) from ESM-2-pt, ESM-2-RoPE, and ESM-2-ALiBi (used only as evaluation baselines in Stage 3). These are separate models serving different purposes.

Regarding point 2 (the PolA/RNR/HEL analysis): we appreciate the opportunity to clarify that this is not circular. The PolA/RNR/HEL protein complex was used in Section 4.1 solely to validate that ESM-2-PPI's attention scores correlate with known biological interactions and to select an appropriate threshold (top 50 pairs) for the sparse attention mask. Importantly, this validation informed only the selection threshold—not the training of LV-3C or LV-5B.

In Stage 2, LV-3C and LV-5B are trained on the full NCBI Virus database (14,436 genomes), with PPIs inferred independently for each genome. The models never see PolA/RNR/HEL labels during training. The STRING database validation in Section 4.5 uses an entirely independent set of 146 annotated interactions across five different viral genomes, none of which overlap with the PolA/RNR/HEL validation set. Thus, the STRING results represent independent validation of the learned attention patterns.

We have (1) added the following clarifying text to Section 4.1: "This validation serves only to calibrate the PPI selection threshold for ESM-2-PPI. The PolA/RNR/HEL interactions are not used during training of LV-3C or LV-5B, which learn from PPIs inferred independently across all genomes in the training set," and (2) ensured Section 4.5 clearly states that STRING validation is independent from the threshold calibration.

>>>>>>>>>>>>>>>>>>
e. PST is introduced as another model not trained here, but included as a comparator for only one part of the results - Fig 9, evaluation of protein embeddings. It's not clear why it needs to be included in these results, but not for others?
<<<<<<<<<<<<<<<<<<

We thank the reviewer for this concern. PST was included as a comparator because it addresses a closely related objective: generating contextualized protein embeddings that incorporate genomic context. We considered it essential to benchmark against this relevant prior work.
However, PST (v2.1.0) only supports protein-level embeddings—genome-level embedding generation is not available in the public release. Additionally, PST uses a graph neural network architecture for context aggregation, which differs fundamentally from our attention-based framework and does not produce comparable attention scores.
For these reasons, a direct comparison with PST was only feasible for protein embeddings, where both methods produce comparable outputs.
We have added the following clarification to Section 4.4.2: "We note that PST's public release (v2.1.0) does not support genome-level embeddings, and its graph neural network architecture does not produce attention scores comparable to our transformer-based approach; PST is therefore included only in protein embedding evaluations where outputs are directly comparable."

>>>>>>>>>>>>>>>>>>
f. Methods 3.4 selecting an optimal fine tuning strategy: for what model or portion of the model?
<<<<<<<<<<<<<<<<<<

The selection of the optimal fine-tuning strategy described in Section 3.3 (previously 3.4) refers to the strategy applied for extending the context length of the ESM-2 model to create ESM-2-PPI. Specifically, this step identifies the most effective approach to fine-tune ESM-2 to handle long protein sequences (up to 13,000 amino acids) for viral protein interaction inference.
The resulting model (ESM-2-PPI) is then used for protein–protein interaction identification (Figure 2a). The interactions predicted with this model subsequently serve to construct the sparse attention patterns for training LV-3C and LV-5B.
We have (1) renamed this section to "Optimizing the PPI Inference Model (Stage 1)" (now Section 3.3) to clarify its purpose, and (2) added introductory text explicitly stating that this section describes the optimization of the Stage 1 model (ESM-2-PPI).

>>>>>>>>>>>>>>>>>>
3. The paper would *greatly* benefit from some revision and streamlining to make the entire process, the

models used, and the methods more clear. I would suggest trying to move some of the results to a supplemental section: the discussion of interaction inference (section 4.1) or 'stage 1' and the different things tried there to be confusing - whereas I might be very interested in those results and what was being done if they weren't confusing (moving those to the supplement is one way of accomplishing this, then describing 'we optimized stage 1 (see supplement) and decided on using XXX for the final model because it showed XXX.'
<<<<<<<<<<<<<<<<<<

We agree that streamlining the main text improves readability. We have moved the detailed “ablation” studies for the PPI Inference Model (Stage 1) to a new Supplementary Section ('Optimization of the PPI Inference Model'), including the comparison of positional encoding strategies (Supplementary Table 1, Supplementary Figure 1) and the threshold selection analysis (Supplementary Figures 2-3, Supplementary Table 2). The main text now contains a condensed Section 4.1 that summarizes the optimization criteria, final model choice, and key validation results, with references to the Supplementary Material for complete details.
>>>>>>>>>>>>>>>>>>
4. Each in the results must have a clear motivation sentence or section right at the start: what is the hypothesis that's being examined here? What are you doing to test this hypothesis (give enough of an idea of the approach/methods to give the reader a reminder)? Then at the end, What do the results tell you and how does it suggest the next section?
<<<<<<<<<<<<<<<<<<

We thank the reviewer for this suggestion. We have revised every single Results subsection to include an opening sentence stating the motivation or hypothesis being tested, a brief reminder of the approach, and where appropriate, a concluding sentence linking to the next section.

>>>>>>>>>>>>>>>>>>
5. The Related Work section is well-written and was very useful to give me good background information. However, it reads more like a mini-review paper than a focused examination of the approaches which are used in the current paper - and, importantly, it's not clear that all the methods described are important to understand for the paper. That is, which parts are *needed* for the reader to understand the approaches tested and/or used in the final work. A short section at the start of this that briefly teases that the final model includes elements from these different approaches would be helpful so the reader knows why they should be paying attention.
<<<<<<<<<<<<<<<<<<

We have added an introductory paragraph that previews how our final model draws on context length extrapolation (ALiBi), sparse attention mechanisms (Longformer-style), and protein language modeling (ESM-2). Specifically, we have added the following text at the start of section of 2, before 2.1

"Our approach draws on advances from several areas reviewed below. From context length extrapolation methods (Section 2.1.1), we adopt ALiBi positional encoding for stable inference on extended sequences. From attention matrix approximation strategies (Section 2.1.4), we build on sparse attention patterns similar to Longformer, but replace fixed patterns with biologically-informed sparse attention derived from predicted protein-protein interactions. From protein language modeling (Section 2.2.1), we leverage ESM-2 both as a foundation for PPI inference and as initialization for one of our models. This section provides background on each of these areas to contextualize our contributions."

>>>>>>>>>>>>>>>>>>
6. Perplexity and silhouette score are used extensively in the results section, but never described in the methods section.
<<<<<<<<<<<<<<<<<<

We thank the reviewer for this observation. We have added a new subsection (“ Evaluation Metrics”) to the Methods section clearly describing both evaluation metrics immediately before we discuss how these metrics are used in the “Evaluating Global Performance and Embeddings Quality” section.

>>>>>>>>>>>>>>>>>>
7. The final paragraph of the introduction seems out of place. This should be moved to the discussion maybe? It's an odd way to end the introduction, and feels like it's more of a caveat that can be discussed after, than the most important thing we should be taking from the paper.
<<<<<<<<<<<<<<<<<<

We agree - we have moved this text to the Discussion instead. The Introduction now concludes with a summary of our contributions.

>>>>>>>>>>>>>>>>>>
8. Methods: the description of Splits is not clear. It seems that genomes, and collections of proteins from the same genome were kept together in a single split so as not to contaminate the evaluation, which makes sense, but just needs to be stated in a more careful and clear way.
<<<<<<<<<<<<<<<<<<

We have revised the Splits paragraph in Section 3.1 (Datasets and Preprocessing) to more precisely describe our strategy for preventing data leakage. The text says now the following:

To prevent data leakage, we partitioned the dataset at the genome level: all proteins from a given genome were assigned to the same split (train, validation, or test). Splits were stratified by taxonomic species to maintain balanced representation across partitions. The final proportions were 80% train, 10% validation, and 10% test. This design ensures that no protein from a genome in the test set could have been seen during training, either directly or through related proteins from the same genome.

>>>>>>>>>>>>>>>>>>
9. Methods: protein non-redundancy - the use of an identity filter to separate similar proteins is A) welcome, it's an important thing to do, but… B) 90% seems overly generous - that is, it makes the task pretty easy since 89% sequence identity is still *very* similar. I would welcome a mention of this in the discussion, and some more supplemental results that show a few of the downstream task comparisons using the more stringent 50% filter.
<<<<<<<<<<<<<<<<<<

We thank the reviewer for this important point. Our 90% identity threshold reduces the dataset from 683,242 to 348,751 sequences (49% reduction), removing highly abundant sequences, which occurred dozens or hundreds of times and which would otherwise dominate training.
To assess sensitivity to this threshold, we additionally clustered at 65% identity, yielding 246,737 sequences. This indicates that 71% of sequences retained at 90% clustering have no other sequence sharing >65% identity, i.e., the majority of our evaluation data consists of substantially divergent sequences, not near-duplicates at 89% similarity. Furthermore, 62.5% of sequences at the 65% threshold are singletons with no similar sequences in the database.
We also note that the impact of sequence similarity varies by evaluation task. Perplexity is most sensitive to train/test similarity since predicting masked residues is easier when similar sequences were seen during training. In contrast, taxonomy and species classification are less affected since our genome-level splitting ensures that test proteins come from entirely different genomes than training proteins, and these tasks require learning species-level patterns and generalizing to other proteins of the same species, making within-species similarity a desired signal rather than a confounder.
Critically, all models (ours and baselines) were evaluated under identical deduplication and splitting conditions. The consistent performance advantage of LV-3C and LV-5B across all tasks reflects genuine improvements in capturing genomic context, not artifacts of the evaluation setup.
We have clarified the rationale for the 90% threshold in the Methods section and added a discussion of threshold sensitivity, including the 65% clustering analysis, to the Discussion section.

>>>>>>>>>>>>>>>>>>
10. Methods: metadata - I can't tell if the first paragraph only is the 'metadata' part (that seems

reasonable) but the rest of the section is not about metadata and probably should be titled something on its own.
<<<<<<<<<<<<<<<<<<

The reviewer is correct. The paragraph following "Metadata" was incorrectly left without a section heading. We have separated the "Metadata" paragraph from the subsequent methodological content and incorporated the pipeline overview into the new Section 3.2 (Training and Evaluation Pipeline Overview).

>>>>>>>>>>>>>>>>>>
11. Methods: the section titled 'Ablation' doesn't immediately strike me as being about ablation at all? For this (and some of the other methods sections too) a short lead in of what the method describes would be good 'We needed an approach to limit the number of interactions used for the context so we …'. Also, leading with 'We varied k…' - what is k (it's there in the equation, but the reader won't know that)?
<<<<<<<<<<<<<<<<<<

Agreed. "Ablation" is not the best title for this section. We have renamed it to "Interaction Threshold Selection" and added introductory context that defines the parameter
k (the number of top-ranked protein pairs included in the sparse attention mask) before describing how its value was selected.

>>>>>>>>>>>>>>>>>>
12. Figure listing is out of order it seems? Maybe because methods refer to results section - not sure.
<<<<<<<<<<<<<<<<<<

Thank you for raising this issue, we have noticed that this happens indeed because the Methods references Results figures. Fixing this requires restructuring the manuscript. We have reviewed figure references throughout the manuscript and made sure that all figures are correctly labeled and referenced.

>>>>>>>>>>>>>>>>>>
13. Table 1 - what is S2? Also really not clear what the other labels refer to exactly either
<<<<<<<<<<<<<<<<<<

In Table 1, S2-Attn refers to the shifted sparse attention mechanism presented in the LongLoRA paper. In this table, labels (Longformer, S2-Attn and BigBird) refers to the attention mechanism used during fine-tuning to extend the context of ESM-2.

We have updated the Table 1 caption to explicitly define each attention mechanism: "Longformer refers to the Sliding Window Attention pattern from [25], S^2-Attn to the Shifted Sparse Attention from LongLoRA [26], and BigBird to the eponymous attention pattern [27]." We have also ensured that the label "ESM-2-pt" is consistent with the standardized naming convention defined in Section 3.2.

>>>>>>>>>>>>>>>>>>
14. Figure 5 is fairly easy to digest visually (removing the adjacent proteins improves the ranking of known interactions) but would very much benefit from a quantitative measure of how different these distributions are (likely a p-value from some test)
<<<<<<<<<<<<<<<<<<

To quantitatively assess the difference between the two distributions shown in Figure 5, we performed a non-parametric Wilcoxon–Mann–Whitney test, as the score distributions did not meet the normality assumption. The test revealed a highly significant difference between the two groups ($p = 3.31\times10^{-22}$), indicating that the ranking of known interactions is substantially improved when adjacent proteins are removed.

We have added this statistical comparison to the figure caption.

>>>>>>>>>>>>>>>>>>
15. Section 4.2. The motivation for using MMseqs2 is not clear. I gather it's to provide a baseline 'trusted' clustering to compare to? Also this is not described in the methods.
<<<<<<<<<<<<<<<<<<

The reviewer is correct. MMseqs2 clustering serves as a reference standard based on sequence similarity: we evaluate whether proteins that cluster together by sequence identity also cluster together in embedding space (as measured by silhouette score). This assesses whether the learned embeddings preserve fundamental sequence relationships.
We have added a description of this evaluation approach to the Methods section and clarified the motivation in Section 4.2.

We have added the following text to Section 4.2: "We used MMseqs2 sequence-based clustering as a reference to assess whether embeddings preserve fundamental sequence relationships—proteins that share high sequence identity should be proximal in embedding space."

>>>>>>>>>>>>>>>>>>
16. I would suggest moving the discussion of analysis of variance and distribution of the embeddings to the first downstream analysis reported. It is useful, but really doesn't say anything about model quality or performance by itself. If you put it first (in section 4.4) it will lead in to the other measures, which are stronger (in my opinion).
<<<<<<<<<<<<<<<<<<

We appreciate this suggestion. We agree, this suggested reorganization improves the narrative. We have reorganized Section 4.4 to present this descriptive analysis first.

Section 4.4 now presents subsections in this order: (1) Analysis of Embedding Variance and Distribution, (2) Evaluation of Taxonomic Information Preservation, (3) Analysis of Species Clustering Efficiency.

>>>>>>>>>>>>>>>>>>
17. Comparison of the embeddings to STRING is a nice addition, but not much time is spent on describing how this is done. 146 interactions (I'm assuming these are TPs) are considered. What are used as negatives? What does it mean to have an F1 score of 0.74 (which seems - pretty good) and an AUC of 0.66 (which seems marginal)? The final sentence in this section is not supported (no correlation between STRING confidence scores and attention scores was shown).
<<<<<<<<<<<<<<<<<<

We thank the reviewer for this observation. To clarify, the 146 interactions correspond to experimentally supported STRING interactions (confidence >= 0.500) and serve as positive examples. Negative examples consist of all other protein pairs from the same viral genomes that have no reported interaction in STRING. We acknowledge that this negative set likely contains some true interactions that have not yet been experimentally characterized; this is a common limitation when evaluating incomplete interaction databases. This label noise would be expected to reduce apparent model performance, making our reported metrics conservative estimates. Despite this challenge, the significant predictive performance suggests our attention-based scores capture genuine biological signals.

Regarding the apparent discrepancy between F1 (0.74) and AUC (0.66): the F1 score reflects performance at an optimized decision threshold, where the model achieves good precision–recall balance. Here, the AUC measures ranking performance across all thresholds and is expectedly lower given both the inherent noise in attention scores and the known incompleteness of viral protein interaction databases, where unlabeled positives in the negative set may also degrade ranking metrics.

We agree that the correlation claim was not adequately supported in the original text. We have now computed the Spearman rank correlation between STRING confidence scores and attention-derived interaction scores ($\rho = 0.33$, $p < 0.001$), confirming a statistically significant positive association. This has been added to the revised manuscript.

We have updated the manuscript, which now contains: (1) explicit description of positive and negative examples, (2) acknowledgment that the negative set may contain uncharacterized true interactions, (3) explanation of the F1 vs AUC discrepancy, (4) the Spearman correlation statistic, and (5) a dedicated Methods subsection ("Validation Against Experimentally Curated Interactions") describing the STRING benchmarking procedure in full.

>>>>>>>>>>>>>>>>>>
18. Figure 12 needs a figure legend.
<<<<<<<<<<<<<<<<<<

ROC curve for binary classification of protein–protein interactions based on attention scores. The curve shows the model's ability to distinguish experimentally validated interactions (STRING confidence ≥ 0.500) from non-interacting protein pairs across five viral genomes (146 positive interactions). The AUC of 0.66, with a 95% bootstrapped confidence interval of [0.56, 0.75], indicates statistically significant predictive performance.
We have added this caption to Figure 12.

>>>>>>>>>>>>>>>>>>
19. Section 4.3 the authors state that 'The aim of clustering-based evaluation is to identify consistency between embeddings in latent space' - but it's not clear that this is how clustering is being used or evaluated following this? - the performance of the different models on a classification task don't directly evaluate how consistent the embeddings are (you could have very different embeddings that lead to similar performance, e.g.)
<<<<<<<<<<<<<<<<<<

The reviewer raises a valid point. We have revised the text to better describe what each evaluation measures. The silhouette score evaluation assesses embedding coherence. i.e. , whether proteins from the same taxonomic group cluster together in latent space. The classification evaluation assesses whether embeddings contain sufficient discriminative information for species prediction, which, as the reviewer points out, is different. We have clarified this distinction in the revised manuscript.

We have replaced the original text with: "We employ two complementary evaluations: silhouette scores assess embedding coherence, whether proteins from the same taxonomic species cluster together in latent space, while classification accuracy assesses whether embeddings encode sufficient discriminative information for species prediction. These metrics capture related but distinct properties: coherent embeddings facilitate classification, but similar classification performance can arise from different embedding geometries."

Reviewer #2:
>>>>>>>>>>>>>>>>>>
The authors trained a new long-context protein language model for viral protein analysis. In their work, they leverage a biologically informed sparse attention mechanism which also incorporated the inter-protein relationship as sparsity priors. They then evaluated and showed that their model has an improved perplexity and embedding quality. Overall, I think the biological question is important and the method part is relatively convincing. Although I also have few concerns that may further help improve the manuscript.
<<<<<<<<<<<<<<<<<<

We thank the reviewer for their thoughtful evaluation and for recognizing the importance of the biological problem our research solves and the convincing nature of our methodology. We address each concern

below.

>>>>>>>>>>>>>>>>>>
Major comments:
1. Viral species, genome sizes and embedded proteins varied dramatically, excepting the deduplication, the authors should discuss clearer how they get a clean and high-quality data for the training. What is the model performance for different viral group?
<<<<<<<<<<<<<<<<<<

We thank the reviewer for this important point. Our training data was obtained from the NCBI Virus database (retrieved June 2024), which provides curated, complete viral genomes with standardized annotations. We applied the following quality control steps:

Only complete genomes were included (14,436 genomes total)
Proteins were deduplicated using CD-HIT at 90% identity
Genome-level splits ensured no proteins from the same genome appeared in both training and test sets
Taxonomic stratification maintained balanced representation across viral families

We also added the following sentence to the Datasets and Preprocessing section:

- We used the NCBI Virus database (retrieved 2024-06), selecting only genomes annotated as ”complete'' (14,436 genomes).

Regarding performance across viral groups: we have added an analysis examining model performance on RNA viruses, which represent approximately 70% of our training data and constitute the most challenging viral subgroup due to their high mutation rates, rapid evolution, and diverse genome organizations. As shown in Supplementary Figure S6 in the supplementary material, our models consistently outperform baselines on RNA viruses, though with slightly lower absolute performance compared to all viruses combined, as expected given the greater complexity of this subgroup. Since RNA viruses represent the most demanding test case, we expect performance on structurally simpler dsDNA and ssDNA viruses to be comparable or better. This analysis is presented in the Supplementary Material (Section "Model Performance Across Viral Subgroups").

>>>>>>>>>>>>>>>>>>
2. From the main text, I don't know how the authors selected the 83 viral genomes from the NCBI database step by step. Also, what viruses were chosen? They may picked the ones with PolA, RNR, and HEL proteins, but whether it is enough for the downstream evaluation should be proved. Whether it could be used for unknown pairs as a viral protein language model.
<<<<<<<<<<<<<<<<<<

We apologize for the lack of clarity. The 83 viral genomes used for the PolA/RNR/HEL validation were selected as follows:

We performed blast and Soft alignment (protocols described in 1 and 2 – see below) searches against the NCBI Virus database using reference sequences for PolA, RNR, and HEL

We retained only genomes where all three proteins were detected with high confidence (e-value < 1e-3, coverage > 80% or soft alignment score >45% over the shorter of the query or target sequence [1, 2])

This yielded 83 genomes, primarily from the previous ICTV Caudovirales order (tailed bacteriophages), where these replication proteins are well-conserved.

We emphasize that this 83-genome subset was used exclusively to validate the PPI inference model (Stage 1 of building the model) and to select the interaction threshold parameter. It was not used for training LV-

3C or LV-5B, which were trained on the full NCBI Virus database (14,436 genomes).

Regarding generalization to unknown pairs: this is precisely the goal of our approach. The STRING database validation (Section 4.5) demonstrates that our model generalizes beyond the PolA/RNR/HEL complex. The 146 STRING interactions used for evaluation span five different viral genomes and include diverse protein pairs not seen during threshold selection. The significant AUC (0.66, 95% CI [0.56, 0.75]) and correlation with STRING confidence scores (Spearman $\rho = 0.33$, $p < 0.001$) confirm that our attention-based interaction scores generalize to novel protein pairs.
We have clarified the distinction between the Stage 1 PPI inference validation set (83 genomes) and the Stage 3 independent evaluation set (STRING interactions) in the revised manuscript.
We have also added a description of the genome selection criteria to the Supplementary Material under "Optimization of the PPI Inference Model (Stage 1)".

Harrigan, W.L., et al. Improvements in viral gene annotation using large language models and soft alignments. BMC Bioinformatics 25, 165 (2024). https://doi.org/10.1186/s12859-024-05779-6
Sawhney, Rajan, et al. "Fine-tuning protein language models unlocks the potential of underrepresented viral proteomes." PeerJ 13 (2025): e19919.

>>>>>>>>>>>>>>>>>>>
3. Additional biological validations may be needed, such as known viral protein motifs or domains.
<<<<<<<<<<<<<<<<<<<

We thank the reviewer for this suggestion. We have added an analysis evaluating our embeddings against Pfam protein family annotations (Supplementary Figure S5).
Interestingly, silhouette scores using Pfam families as cluster labels are negative across all models, indicating that proteins from the same domain family do not cluster tightly in embedding space. This result is expected and validates our approach: our embeddings are designed to encode genomic context, not solely domain identity. Proteins with identical Pfam domains but different genomic environments (e.g., a helicase in a bacteriophage vs. an RNA virus) should, and do as shown in the results, receive distinct embeddings.
Importantly, classification performance remains strong, with LV-3C and LV-5B outperforming baselines across all numbers of Pfam families. This demonstrates that protein family and domain information is encoded within our embeddings; it is simply not the dominant signal. Our models thus capture functional domain information at least as well as single-protein baselines, while additionally encoding the genomic context that is the focus of this work.

We have added an analysis to the Supplementary Material (Section "Validation Using Protein Domain Annotations") and referenced it in the Discussion.

Editorial Manager®

Cookie Preference Center

We use cookies which are necessary to make our site work. We may also use additional cookies to analyze, improve and personalize our content and your digital experience. For more information, see our Cookie Policy.

You may choose not to allow some types of cookies. However, blocking some types may impact your experience of our site and the services we are able to offer. See the different category headings below to find out more or change your settings.

Allow all

Manage Consent Preferences

Strictly Necessary Cookies

Always active

These cookies are necessary for the website to function and cannot be switched off in our systems. They are usually only set in response to actions made by you which amount to a request for services, such as setting your privacy preferences, logging in or filling in forms. You can set your browser to block or alert you about these cookies, but some parts of the site will not then work.

Functional Cookies

These cookies enable the website to provide enhanced functionality and personalisation. They may be set by us or by third party providers whose services we have added to our pages. If you do not allow these cookies then some or all of these services may not function properly.

Performance Cookies

These cookies allow us to count visits and traffic sources so we can measure and improve the performance of our site. They help us to know which pages are the most and least popular and see how visitors move around the site.

Targeting Cookies

These cookies may be set through our site by our advertising partners. They may be used by those companies to build a profile of your interests and show you relevant adverts on other sites. If you do not allow these cookies, you will experience less targeted advertising.

Cookie List

Clear

Apply Cancel

Consent Leg.Interest

Confirm my choices

Power
