# Supplementary material for: Extending protein language models to a viral genomic scale using biologically induced sparse attention
Source: Gigascience. 2026 Jul 21;15:giag081. doi: 10.1093/gigascience/giag081 (PMC13495794; doi:10.1093/gigascience/giag081)
Supplement: Supplementary file 2 [file giag081_authors_response_to_reviewer_comments_revision_1.pdf]

Author's Response To Reviewer Comments

Responses to reviewers:

Reviewer #1: The authors do a reasonable job of addressing the concerns I previously raised and the manuscript is improved with respect to clarity and content. I believe that this is a valuable study with interesting results. However, one point was not addressed (shortening and clarifying the introduction) and for several others the authors response did not match the updated reorganized text. Though I was able to mostly follow the changes, the lack of clarity in the response and the authors lack of care with their responses made this challenging:

We thank the reviewer for the positive feedback and the careful reading. We apologize that our previous edits were harder to follow than they should have been. This revision implements all of the reviewer's requested changes, with removed text shown in strikethrough and new text in blue so it can be located directly in the manuscript. Our point-by-point responses are provided below.

1. My original point 5 is addressed in the authors response, but not included in the paper. The introduction is still too long and has not been revised/clarified.

Thank you for the suggestion. We have tightened and cleaned the Introduction, removing redundant or irrelevant passages and streamlining the motivation. The section is now substantially more focused.

2. My original point 13 has been addressed but in the restructuring of the paper Table 1 has been moved to the supplement, which is fine, but just makes tracking the updates to the paper difficult.

Original Table 1 is now Supplementary Table S1. We apologize for not flagging this relocation clearly in the previous response.

3. Likewise - my original point 14 was about Figure 5, which was addressed but Figure 5 was moved to Supplementary Figure S3 (not indicated in the revision comments).

Again, we apologize for failing to flag this move. Original Figure 5 is now Supplementary Figure S3.

4. My point 15 was addressed? I think - but the response from the authors to my point includes text that is not in the paper so it's hard to evaluate.
Apologies for the confusion, during the reorganization, the material was moved into the Methods and edited for clarity. The text is in blue in Methods, Section 3.7, under "Sequence-based clustering reference".

5. My original point 18: Figure 12 has been moved to supplement, but this wasn't indicated in the response.

Again, we apologize for failing to flag this move.

Since the paper was substantially reorganized I reread it to find a few issues not addressed or not present in my first review:
1. In the abstract the word 'perplexity' is used to describe the improvement in performance of the method. Though perplexity is a (somewhat) common term for AI/ML this should be explained for readers not

familiar - or another word/phrase used in the abstract at least.

The abstract no longer uses 'perplexity.' The term is now briefly clarified the first time it is used (Section 3.3) and formally defined in Evaluation Metrics (Section 3.6).

2. In the introduction the authors state: "Our semi-supervised protein language model employs sparse attention mechanisms guided by known protein-protein interactions." which is misleading given that a major point of the paper is to use PLMs to infer protein interactions? It seems that the model *can* use known protein-protein interactions, but that's not the main thrust of the paper.

We agree with the reviewer and have corrected the abstract and Introduction which now state that interactions are inferred computationally and used as sparsity priors, not "known."

3. As noted previously and above section 2 is great, but doesn't need to be here. This should be condensed down to *only* what is necessary to understand the motivation for choices in the paper. As it stands this significantly detracts from the paper since it is a *lot* of unnecessary content that readers must get through before they get to the content of the paper.

We have substantially condensed Section 2, retaining only the background needed to motivate our design choices.

4. Inferring PPIs to address the attention issue is a strong idea. However, the text states that this is a 'biologically informed sparse attention method', which is not really correct. The *motivation* for this is biological information - but the way it's being applied is through further inference. It is important to note that prediction of protein-protein interactions is not a solved problem, and - importantly - this paper does not really address the issue. The use of a single protein complex to evaluate/parameterize the approach is OK, but is insufficient to establish that it works to accurately predict protein interactions. The approach itself (using ESM to filter for interactions) is fine - I would simply suggest revising the language about what this process is accomplishing (maybe 'enriching for putative protein-protein interactions' or something similar). It would be important to see an addition to the analysis and evaluation that would be a model which included randomized protein interactions (instead of inferred) to show that the PPI inference process improves overall model performance. The baselines provided by ESM-pt, ESM-ROPE, and ESM-ALIBI are good, but don't *seem* to address the same question [it is unclear to me, which needs to be clarified as well].

We thank the reviewer for this important request. We agree that our method should not be described as directly predicting physical PPIs. We have revised the manuscript to describe the interaction-inference step as producing putative interaction links or sparsity priors, rather than solved or experimentally validated PPIs. We also added a new experiment in Supplementary Section S6 to test whether the inferred links improve the model beyond random or distance-matched links. To test this fairly requires several independently trained models. A random choice requires multiple replicates; and because the inferred links tend to fall at particular genomic distances, we also need a control that draws random links at distances similar to those of the inferred pairs (otherwise a difference could come from distance rather than from which pairs are chosen). Together with a model given no long-range links at all (i.e., model relying on direct neighbors only), eight models are needed:

- inferred (1 model): the 50 top-ranked pairs from the interaction-inference model (the ESM-2-PPI model described in the manuscript).
- random (3 models): 50 pairs drawn uniformly at random with three independent draws whose results we average
- distance-matched (3 models): 50 random pairs drawn to match the range (maximum genomic distance) of the inferred pairs.
- no-link (1 model): only neighboring proteins are connected.

As such, rather than the large models from the paper, we ran this controlled comparison with compact ~35M-parameter models instead. Each model was trained from scratch and all eight models share the same architecture, initial weights, training data and order, masking, and optimizer. Since the only

difference is which 50 links each one is given (if any), differences in their results can therefore come only from which pairs are connected.

On 600 held-out genomes, held-out perplexity was:

Model; Held-out perplexity
no long-range links; 16.120
50 random links (average of 3); 16.124
50 distance-matched links (average of 3); 16.119
50 inferred links; 16.064

Adding 50 random links leaves perplexity essentially unchanged from having no long-range links (16.124 vs. 16.120); adding the same number of inferred links lowers it (lower is better). The benefit therefore comes from which pairs are connected, not from simply adding connections. Matching the random links to the inferred links' genomic distances does not close the gap either as the distance-matched control performs like the other controls (no-link, random, and distance-matched all land at ≈16.12). The advantage therefore reflects which proteins are paired, not how far apart they sit.

The genome-wide change is small (about 0.4%), but that number is an average over every position in the genome. The 50 links leave most positions untouched, because they matter only for the minority of positions that actually depend on an interaction partner. Averaging those few changed positions together with the many unchanged ones is what makes the effect look small. It is nonetheless real. Even diluted this way, the 50 inferred links outperform the 50 random links, the 50 distance-matched links, and no links at all.

The advantage is also consistent across genomes. The inferred model has lower perplexity than the random average on 554 of 600 held-out genomes (92%). It stays lower when compared against each of the three random sets on its own, and even against the best of the three chosen separately for each genome; that is, for each genome we take lowest perplexity achieved by any of the three random models, and inferred still wins on 78% of genomes.

We also examined where in each genome the improvement occurs. Since most positions do not change, the improvement is concentrated on a small fraction of positions, and those positions sit disproportionately on the proteins involved in the interaction-inference model's highest-scoring pairs. We rank positions by how much the inferred links improve them, i.e., the drop in per-position loss relative to the control, and asked where the most-improved ones fall. In the table below, the baseline rate is the fraction of the genome taken up by the proteins in the kept pairs (what we would expect by chance), and the top-100 enrichment is how much more often the 100 most-improved positions land on those pairs than that baseline. The enrichment changes smoothly with the confidence of the pairs considered. The enrichment is ~11X for the very highest-scoring pairs, 5.2X at the top 10%, and 1.4X when all 50 are pooled. These improvements follow the model's score rather than appearing at an arbitrary cut-off.

Edges kept (by confidence); baseline rate; top-100 enrichment; top-1000 enrichment
top 2%; 4.1%; 11.1X; 7.4X
top 5%; 9.0%; 7.2X; 5.6X
top 10%; 13.8%; 5.2X; 4.2X
top 20%; 25.6%; 3.1X; 2.7X
top 50%; 50.4%; 1.7X; 1.6X
all (loose); 71.6%; 1.4X; 1.3X

With every other setting held identical, 50 randomly chosen links give no measurable improvement over having no long-range links, whereas 50 links chosen by the interaction-inference model lower held-out perplexity. The advantage is consistent across genomes and concentrates on the pairs the model scores highest.

5. 3.10 and 3.11 seem to be referring to a downstream task rather than the upstream task of predicting

PPIs to make sparse attention matrices. However, the parallel nature of these activities is confusing and should be carefully described and delineated.

We have added clarifying text at the start of the across-model attention analysis explicitly distinguishing the Stage 1 PPI inference (used to build the sparse-attention priors) from the downstream evaluation of attention in the trained LV-3C/LV-5B models.

6. It is really unclear from section 4.1 what is being done and why? There are insufficient results presented in 4.1 to demonstrate that this is working (see my point 4 above). The wording in places is confusing: "we optimized a PPI inference model (ESM-2-PPI) to" implies that an existing model is being optimized, when really this is a new model based on ESM. Explaining what this is and why it's done briefly again here would be important. 3.2 stage 1 describes the ESM-2-PPI training process, and does not mention anything about about using ROPE and ALIBI models in the PPI prediction, and the reasons for using them are not clearly laid out in 4.1. "We evaluated two positional encoding.." - evaluated on what? Again - this is in the Methods somewhere, but needs to be also described here.

Section 4.1 (now labeled as "Developing the PPI Inference Model") opens by explaining that ESM-2-PPI is a new auxiliary model built solely to derive the interaction priors, why the positional-encoding (RoPE vs ALiBi) and sparse-attention comparisons were run, and on what data they were evaluated.

7. Section 4.2 highlights my prior comment (#4) about needing to motivate each results section. The section starts out, "The LV-3C and LV-5B models demonstrated significantly lower perplexity…" - where we don't know what LV-3C and LV-5B models are and how they differ (again, yes - the Methods section talks about this, but this needs to be clearly motivated and explained, briefly, in this section as well).
Thank you for raising this. We have added text to Section 4.2, which now opens with a brief description of LV-3C and LV-5B (size, initialization, training length) and how they differ, before presenting results.

8. Figure 7: it's not clear what the different number of clusters means - I assume from broader (smaller number of clusters) to more specific (larger number of clusters) for taxonomy. This should be made clear in the text.

We clarified the different number of clusters meaning in the caption. The x-axis is now shown as the number of viral species used as classification targets (5 to 100); larger values denote finer-grained taxonomic resolution.

Reviewer #2: The authors well addressed all my comments.

We thank the reviewer for the positive review.

Editorial Manager®

Cookie Preference Center

We use cookies which are necessary to make our site work. We may also use additional cookies to analyze, improve and personalize our content and your digital experience. For more information, see our Cookie Policy.

You may choose not to allow some types of cookies. However, blocking some types may impact your experience of our site and the services we are able to offer. See the different category headings below to find out more or change your settings.

Allow all

Manage Consent Preferences

Strictly Necessary Cookies

Always active

These cookies are necessary for the website to function and cannot be switched off in our systems. They are usually only set in response to actions made by you which amount to a request for services, such as setting your privacy preferences, logging in or filling in forms. You can set your browser to block or alert you about these cookies, but some parts of the site will not then work.

Functional Cookies

These cookies enable the website to provide enhanced functionality and personalisation. They may be set by us or by third party providers whose services we have added to our pages. If you do not allow these cookies then some or all of these services may not function properly.

Performance Cookies

These cookies allow us to count visits and traffic sources so we can measure and improve the performance of our site. They help us to know which pages are the most and least popular and see how visitors move around the site.

Targeting Cookies

These cookies may be set through our site by our advertising partners. They may be used by those companies to build a profile of your interests and show you relevant adverts on other sites. If you do not allow these cookies, you will experience less targeted advertising.

Cookie List

Clear

Apply Cancel
Consent Leg.Interest

Confirm my choices

Power
