# Supplementary material for: Extending protein language models to a viral genomic scale using biologically induced sparse attention
Source: Gigascience. 2026 Jul 21;15:giag081. doi: 10.1093/gigascience/giag081 (PMC13495794; doi:10.1093/gigascience/giag081)
Supplement: Supplementary file 5 [file giag081_giga-d-25-00436_revision_2.pdf]

GigaScience

Extending Protein Language Models to a Viral Genomic Scale Using Biologically Induced Sparse Attention

--Manuscript Draft--

**Manuscript Number:**	GIGA-D-25-00436R2
**Full Title:**	Extending Protein Language Models to a Viral Genomic Scale Using Biologically Induced Sparse Attention
**Article Type:**	Research
**Funding Information:**	National Science Foundation (1736030) — Dr K. Eric Wommack<br>National Science Foundation (2025567) — Dr K. Eric Wommack
**Abstract:**	The transformer architecture in deep learning has revolutionized protein sequence analysis. Recent advancements in protein language models have paved the way for significant progress across various domains, including protein function and structure prediction, multiple sequence alignments and mutation effect prediction. A protein language model is commonly trained on individual proteins, ignoring the interdependencies between sequences within a genome. However, biological understanding reveals that protein–protein interactions span entire genomic regions, underscoring the limitations of focusing solely on individual proteins. To address these limitations, we propose a novel approach that extends the context size of transformer models across the entire viral genome. By training on large genomic fragments, our method captures \emph{putative} long-range dependencies consistent with inter-protein relationships and encodes protein sequences with integrated information from distant proteins within the same genome, offering benefits across downstream tasks. Viruses, with their densely packed genomes, minimal intergenic regions, and protein annotation challenges, are ideal candidates for genome-wide learning. We introduce a long-context protein language model, trained on entire viral genomes, leveraging a biologically informed sparse attention mechanism in which inter-protein links are \emph{inferred computationally} and used as sparsity priors. Our semi-supervised approach supports long sequences of up to 61,000 amino acids (aa). Our evaluations show improved perplexity and downstream discrimination relative to single-protein models and long-context baselines, with additional validation that our inferred links correlate with independently curated interaction resources.
**Corresponding Author:**	Mahdi Belcaid, Ph.D.<br>University of Hawai'i at Manoa<br>Honolulu, HI UNITED STATES
**Corresponding Author Secondary Information:**	
**Corresponding Author's Institution:**	University of Hawai'i at Manoa
**Corresponding Author's Secondary Institution:**	
**First Author:**	Thibaut Dejean
**First Author Secondary Information:**	
**Order of Authors:**	Thibaut Dejean<br>Barbra D. Ferrell<br>William Harrigan<br>Zachary D. Schreiber<br>Rajan Sawhney<br>K. Eric Wommack<br>Shawn W. Polson

Powered by Editorial Manager® and ProduXion Manager® from Aries Systems Corporation

	Mahdi Belcaid, Ph.D.
**Order of Authors Secondary Information:**	
**Response to Reviewers:**	Responses to reviewers:<br><br>Reviewer #1: The authors do a reasonable job of addressing the concerns I previously raised and the manuscript is improved with respect to clarity and content. I believe that this is a valuable study with interesting results. However, one point was not addressed (shortening and clarifying the introduction) and for several others the authors response did not match the updated reorganized text. Though I was able to mostly follow the changes, the lack of clarity in the response and the authors lack of care with their responses made this challenging:<br><br>We thank the reviewer for the positive feedback and the careful reading. We apologize that our previous edits were harder to follow than they should have been. This revision implements all of the reviewer's requested changes, with removed text shown in strikethrough and new text in blue so it can be located directly in the manuscript. Our point-by-point responses are provided below.<br><br>1. My original point 5 is addressed in the authors response, but not included in the paper. The introduction is still too long and has not been revised/clarified.<br><br>Thank you for the suggestion. We have tightened and cleaned the Introduction, removing redundant or irrelevant passages and streamlining the motivation. The section is now substantially more focused.<br><br>2. My original point 13 has been addressed but in the restructuring of the paper Table 1 has been moved to the supplement, which is fine, but just makes tracking the updates to the paper difficult.<br><br>Original Table 1 is now Supplementary Table S1. We apologize for not flagging this relocation clearly in the previous response.<br><br>3. Likewise - my original point 14 was about Figure 5, which was addressed but Figure 5 was moved to Supplementary Figure S3 (not indicated in the revision comments).<br><br>Again, we apologize for failing to flag this move. Original Figure 5 is now Supplementary Figure S3.<br><br>4. My point 15 was addressed? I think - but the response from the authors to my point includes text that is not in the paper so it's hard to evaluate.<br>Apologies for the confusion, during the reorganization, the material was moved into the Methods and edited for clarity. The text is in blue in Methods, Section 3.7, under "Sequence-based clustering reference”.<br><br>5. My original point 18: Figure 12 has been moved to supplement, but this wasn't indicated in the response.<br><br>Again, we apologize for failing to flag this move.<br><br>Since the paper was substantially reorganized I reread it to find a few issues not addressed or not present in my first review:<br>1. In the abstract the word 'perplexity' is used to describe the improvement in performance of the method. Though perplexity is a (somewhat) common term for AI/ML this should be explained for readers not familiar - or another word/phrase used in the

abstract at least.

The abstract no longer uses 'perplexity.' The term is now briefly clarified the first time it is used (Section 3.3) and formally defined in Evaluation Metrics (Section 3.6).

2. In the introduction the authors state: "Our semi-supervised protein language model employs sparse attention mechanisms guided by known protein-protein interactions." which is misleading given that a major point of the paper is to use PLMs to infer protein interactions? It seems that the model *can* use known protein-protein interactions, but that's not the main thrust of the paper.

We agree with the reviewer and have corrected the abstract and Introduction which now state that interactions are inferred computationally and used as sparsity priors, not "known."

3. As noted previously and above section 2 is great, but doesn't need to be here. This should be condensed down to *only* what is necessary to understand the motivation for choices in the paper. As it stands this significantly detracts from the paper since it is a *lot* of unnecessary content that readers must get through before they get to the content of the paper.

We have substantially condensed Section 2, retaining only the background needed to motivate our design choices.

4. Inferring PPIs to address the attention issue is a strong idea. However, the text states that this is a 'biologically informed sparse attention method', which is not really correct. The *motivation* for this is biological information - but the way it's being applied is through further inference. It is important to note that prediction of protein-protein interactions is not a solved problem, and - importantly - this paper does not really address the issue. The use of a single protein complex to evaluate/parameterize the approach is OK, but is insufficient to establish that it works to accurately predict protein interactions. The approach itself (using ESM to filter for interactions) is fine - I would simply suggest revising the language about what this process is accomplishing (maybe 'enriching for putative protein-protein interactions' or something similar). It would be important to see an addition to the analysis and evaluation that would be a model which included randomized protein interactions (instead of inferred) to show that the PPI inference process improves overall model performance. The baselines provided by ESM-pt, ESM-ROPE, and ESM-ALIBI are good, but don't *seem* to address the same question [it is unclear to me, which needs to be clarified as well].

We thank the reviewer for this important request. We agree that our method should not be described as directly predicting physical PPIs. We have revised the manuscript to describe the interaction-inference step as producing putative interaction links or sparsity priors, rather than solved or experimentally validated PPIs. We also added a new experiment in Supplementary Section S6 to test whether the inferred links improve the model beyond random or distance-matched links. To test this fairly requires several independently trained models. A random choice requires multiple replicates; and because the inferred links tend to fall at particular genomic distances, we also need a control that draws random links at distances similar to those of the inferred pairs (otherwise a difference could come from distance rather than from which pairs are chosen). Together with a model given no long-range links at all (i.e., model relying on direct neighbors only), eight models are needed:

- inferred (1 model): the 50 top-ranked pairs from the interaction-inference model (the ESM-2-PPI model described in the manuscript).
- random (3 models): 50 pairs drawn uniformly at random with three independent draws whose results we average
- distance-matched (3 models): 50 random pairs drawn to match the range (maximum genomic distance) of the inferred pairs.
- no-link (1 model): only neighboring proteins are connected.

As such, rather than the large models from the paper, we ran this controlled comparison with compact ~35M-parameter models instead. Each model was trained from scratch and all eight models share the same architecture, initial weights, training

Powered by Editorial Manager® and ProduXion Manager® from Aries Systems Corporation

data and order, masking, and optimizer. Since the only difference is which 50 links each one is given (if any), differences in their results can therefore come only from which pairs are connected.

On 600 held-out genomes, held-out perplexity was:

Model	Held-out perplexity
no long-range links	16.120
50 random links (average of 3)	16.124
50 distance-matched links (average of 3)	16.119
50 inferred links	16.064

Adding 50 random links leaves perplexity essentially unchanged from having no long-range links (16.124 vs. 16.120); adding the same number of inferred links lowers it (lower is better). The benefit therefore comes from which pairs are connected, not from simply adding connections. Matching the random links to the inferred links' genomic distances does not close the gap either as the distance-matched control performs like the other controls (no-link, random, and distance-matched all land at ≈16.12). The advantage therefore reflects which proteins are paired, not how far apart they sit.

The genome-wide change is small (about 0.4%), but that number is an average over every position in the genome. The 50 links leave most positions untouched, because they matter only for the minority of positions that actually depend on an interaction partner. Averaging those few changed positions together with the many unchanged ones is what makes the effect look small. It is nonetheless real. Even diluted this way, the 50 inferred links outperform the 50 random links, the 50 distance-matched links, and no links at all.

The advantage is also consistent across genomes. The inferred model has lower perplexity than the random average on 554 of 600 held-out genomes (92%). It stays lower when compared against each of the three random sets on its own, and even against the best of the three chosen separately for each genome; that is, for each genome we take lowest perplexity achieved by any of the three random models, and inferred still wins on 78% of genomes.

We also examined where in each genome the improvement occurs. Since most positions do not change, the improvement is concentrated on a small fraction of positions, and those positions sit disproportionately on the proteins involved in the interaction-inference model's highest-scoring pairs. We rank positions by how much the inferred links improve them, i.e., the drop in per-position loss relative to the control, and asked where the most-improved ones fall. In the table below, the baseline rate is the fraction of the genome taken up by the proteins in the kept pairs (what we would expect by chance), and the top-100 enrichment is how much more often the 100 most-improved positions land on those pairs than that baseline. The enrichment changes smoothly with the confidence of the pairs considered. The enrichment is ~11X for the very highest-scoring pairs, 5.2X at the top 10%, and 1.4X when all 50 are pooled. These improvements follow the model's score rather than appearing at an arbitrary cut-off.

Edges kept (by confidence)	baseline rate	top-100 enrichment	top-1000 enrichment
top 2%	4.1%	11.1X	7.4X
top 5%	9.0%	7.2X	5.6X
top 10%	13.8%	5.2X	4.2X
top 20%	25.6%	3.1X	2.7X
top 50%	50.4%	1.7X	1.6X
all (loose)	71.6%	1.4X	1.3X

With every other setting held identical, 50 randomly chosen links give no measurable improvement over having no long-range links, whereas 50 links chosen by the interaction-inference model lower held-out perplexity. The advantage is consistent across genomes and concentrates on the pairs the model scores highest.

5. 3.10 and 3.11 seem to be referring to a downstream task rather than the

	upstream task of predicting PPIs to make sparse attention matrices. However, the parallel nature of these activities is confusing and should be carefully described and delineated.<br><br>We have added clarifying text at the start of the across-model attention analysis explicitly distinguishing the Stage 1 PPI inference (used to build the sparse-attention priors) from the downstream evaluation of attention in the trained LV-3C/LV-5B models.<br><br>6. It is really unclear from section 4.1 what is being done and why? There are insufficient results presented in 4.1 to demonstrate that this is working (see my point 4 above). The wording in places is confusing: "we optimized a PPI inference model (ESM-2-PPI) to" implies that an existing model is being optimized, when really this is a new model based on ESM. Explaining what this is and why it's done briefly again here would be important. 3.2 stage 1 describes the ESM-2-PPI training process, and does not mention anything about about using ROPE and ALIBI models in the PPI prediction, and the reasons for using them are not clearly laid out in 4.1. "We evaluated two positional encoding.." - evaluated on what? Again - this is in the Methods somewhere, but needs to be also described here.<br><br>Section 4.1 (now labeled as "Developing the PPI Inference Model") opens by explaining that ESM-2-PPI is a new auxiliary model built solely to derive the interaction priors, why the positional-encoding (RoPE vs ALiBi) and sparse-attention comparisons were run, and on what data they were evaluated.<br><br>7. Section 4.2 highlights my prior comment (#4) about needing to motivate each results section. The section starts out, "The LV-3C and LV-5B models demonstrated significantly lower perplexity…" - where we don't know what LV-3C and LV-5B models are and how they differ (again, yes - the Methods section talks about this, but this needs to be clearly motivated and explained, briefly, in this section as well).<br>Thank you for raising this. We have added text to Section 4.2, which now opens with a brief description of LV-3C and LV-5B (size, initialization, training length) and how they differ, before presenting results.<br><br>8. Figure 7: it's not clear what the different number of clusters means - I assume from broader (smaller number of clusters) to more specific (larger number of clusters) for taxonomy. This should be made clear in the text.<br><br>We clarified the different number of clusters meaning in the caption. The x-axis is now shown as the number of viral species used as classification targets (5 to 100); larger values denote finer-grained taxonomic resolution.<br><br>Reviewer #2: The authors well addressed all my comments.<br><br>We thank the reviewer for the positive review.
**Additional Information:**	
**Question**	**Response**
Are you submitting this manuscript to a special series or article collection?	No
**Experimental design and statistics**<br><br>Full details of the experimental design and statistical methods used should be given in the Methods section, as detailed in our Minimum Standards Reporting Checklist. Information essential to interpreting the data presented should be made available	Yes

in the figure legends.<br><br>Have you included all the information requested in your manuscript?	
**Resources**<br><br>A description of all resources used, including antibodies, cell lines, animals and software tools, with enough information to allow them to be uniquely identified, should be included in the Methods section. Authors are strongly encouraged to cite Research Resource Identifiers (RRIDs) for antibodies, model organisms and tools, where possible.<br><br>Have you included the information requested as detailed in our Minimum Standards Reporting Checklist?	Yes
**Availability of data and materials**<br><br>All datasets and code on which the conclusions of the paper rely must be either included in your submission or deposited in publicly available repositories (where available and ethically appropriate), referencing such data using a unique identifier in the references and in the "Availability of Data and Materials" section of your manuscript.<br><br>Have you have met the above requirement as detailed in our Minimum Standards Reporting Checklist?	Yes
GigaScience has policies and guidelines in place for the use of generative AI-writing tools such as ChatGPT. If you have used such writing tools to assist with writing the manuscript this must be declared and cited in the text. Authors should not list AI-writing tools and other AI-assisted technologies as an author or co-author and should acknowledge that they are fully responsible for text generated or refined by AI-writing	Yes

tools.<p><br><br>A summary of use (particularly in the introduction or among methods) needs to be included at the end of the paper, and the outputs should also be included as a supplementary file hosted in GigaDB or other open repositories. Please read our guidelines for more information. <p><br><br>By submitting to GigaScience, you are aware of the journal's AI-writing tools policy, and if you have declared use of such tools below, you have acknowledged this where appropriate in your manuscript and have made a summary of use and outputs available. <p><br><b>AI-assisted writing tools have been used in the preparation of this manuscript?	

Powered by Editorial Manager® and ProduXion Manager® from Aries Systems Corporation

Manuscript Final Click here to access/download;Manuscript;final_pdf_5_8_26.pdf

Extending Protein Language Models to a Viral Genomic Scale Using Biologically Induced Sparse Attention

Thibaut Dejean
Department of Information and Computer Sciences
University of Hawaii
Honolulu, HI
Barbra D. Ferrell
Department of Computer & Information Sciences
Delaware Biotechnology Institute
University of Delaware, Newark, DE

Zachary D. Schreiber
Department of Computer & Information Sciences
Delaware Biotechnology Institute
University of Delaware, Newark, DE

William Harrigan
Hawai'i Institute of Marine Biology
University of Hawai'i
Kaneohe, HI

Rajan Sawhney
Department of Information and Computer Sciences
University of Hawaii
Honolulu, HI

K. Eric Wommack
Department of Plant & Soil Sciences
Delaware Biotechnology Institute
University of Delaware, Newark, DE

Shawn W. Polson
Department of Computer & Information Sciences
Delaware Biotechnology Institute
University of Delaware, Newark, DE

Mahdi Belcaid*
Department of Information and Computer Sciences
University of Hawaii
Honolulu, HI
mahdi@hawaii.edu
*Corresponding author

Abstract

The transformer architecture in deep learning has revolutionized protein sequence analysis. Recent advancements in protein language models have paved the way for significant progress across various domains, including protein function and structure prediction, multiple sequence alignments and mutation effect prediction. A protein language model is commonly trained on individual proteins, ignoring the interdependencies between sequences within a genome. However, biological understanding reveals that protein–protein interactions span entire genomic regions, underscoring the limitations of focusing solely on individual proteins. To address these limitations, we propose a novel approach that extends the context size of transformer models across the entire viral genome. By training on large genomic fragments, our method captures *putative* long-range dependencies consistent with inter-protein relationships and encodes protein sequences with integrated information from distant proteins within the same genome, offering benefits across downstream tasks. Viruses, with their densely packed genomes, minimal intergenic regions, and protein annotation challenges, are ideal candidates for genome-wide learning. We introduce a long-context protein language model, trained on entire viral genomes, leveraging a biologically informed sparse attention mechanism in which inter-protein links are *inferred computationally* and used as sparsity priors. Our semi-supervised

approach supports long sequences of up to 61,000 amino acids (aa). Our evaluations show improved prediction of masked amino acids and improved downstream discrimination relative to single-protein models and long-context baselines, with additional validation that our inferred links correlate with independently curated interaction resources.

1 Introduction

Deep learning has transformed the analysis of genomic data, driven by transformer models [1, 2]. This shift produced powerful protein language models such as ESM-2, ProtTrans, and ProGen [3, 4, 5], which capture complex relationships between amino acids (aa) within proteins and yield high-dimensional embeddings that are effective across tasks including protein function annotation [6], multiple sequence alignment [7], and structure prediction [8].

However, by focusing on individual proteins, these models miss crucial interactions between distant proteins that shape genome function. While proximity-based interactions such as those within operons are well studied [9], growing evidence highlights long-range protein interactions across large genomic distances [10]; in evolutionary terms, when one protein changes, its partners often must adapt to preserve their collective function [11]. Yet transformer-based models, constrained by limited context windows (e.g., ESM-2 is limited to 1,024 aa, or tokens), cannot process multiple long proteins simultaneously, preventing them from modeling the web of protein interactions across entire genomes.

Recent large language models (LLMs) show that extended context improves the capture of long-range relationships in text [12], suggesting that analogous approaches could yield more accurate protein embeddings and reveal functional relationships between distant proteins that current methods miss. However, extending context is computationally demanding, primarily because attention scales quadratically with sequence length [13]. Existing solutions—from sparse attention patterns to architectural modifications—are optimized for text and do not account for the principles of protein biology, limiting their effectiveness on multiple protein sequences.

To address these limitations, we introduce a protein language model that processes entire viral genomes, achieving a context window exceeding 60,000 aa on a single commodity GPU —covering 98% of all sequenced viral genomes in the NCBI Virus database [14] (Supplementary Figure S1). Our semi-supervised model employs sparse attention guided by computationally inferred protein–protein interactions, and our evaluations show that the resulting viral genome embeddings capture biologically meaningful signals missed by current single-protein models.

2 Related Work

2.1 Long Context LLMs

Recent advances in large language models have sparked intense research into efficiently handling long contexts. Approaches to increasing context length fall broadly into four popular categories [12] : 1) context length extrapolation, 2) hardware and algorithmic strategies, 3) prompt compression, and 4) attention matrix approximation. Context length extrapolation methods enable models to process sequences longer than those on which they were initially trained by manipulating positional embeddings. Hardware and algorithmic strategies focus on optimizing computation and memory usage through system optimizations. Prompt compression methods reduce the sequence length by condensing the input information into more compact representations before processing. Lastly, attention matrix approximation employs computational and statistical strategies to estimate attention mechanisms without incurring the quadratic scaling costs of standard attention. We briefly review these approaches below since our approach builds on advances from several of them. From context length extrapolation methods (Section 2.1.1), we adopt ALiBi positional encoding for stable inference on extended sequences. From attention matrix approximation strategies (Section 2.1.4), we build on sparse attention patterns similar to Longformer, but replace fixed patterns with biologically informed sparsity derived from inferred protein-protein interactions (PPI). We leverage ESM-2 both as a foundation for PPI inference and as initialization for one of our models. This section provides background on each of these areas to contextualize our contributions.

2.1.1 Context Length Extrapolation

A fundamental limitation in extending transformer models to longer sequences lies in Positional Encodings (PEs). While PEs are essential for encoding the sequential order of tokens, their effectiveness diminishes significantly when processing sequences that exceed the training data length. This constraint represents a critical bottleneck when adapting models trained on shorter sequences to handle longer inputs.

Approaches such as rotational position embeddings (RoPE) [15] introduced trainable embeddings that apply position-based rotations to query and key representations. However, traditional PEs suffer substantial performance degradation when processing sequences that are longer than their training context. Two families of methods address this without full retraining: extrapolation and interpolation. Extrapolation methods, exemplified by Attention with Linear Biases (ALiBi) [16], introduce unlearned static biases into attention calculations, systematically adjusting attention scores between key–query pairs based on distance. In contrast, interpolation methods generalize learned PEs to longer sequences, for example by scaling RoPE position indices to the original context window [17].

2.1.2 Hardware and Algorithmic strategies

Training transformer models directly on longer sequences would eliminate the need for positional embedding extrapolation techniques. However, this approach is fundamentally constrained by the quadratic memory complexity inherent to the attention mechanism. Specifically, standard attention mechanisms require memory and computational complexity of $O(n^2d + nd^2)$ per layer, where n represents the sequence length and d denotes the hidden dimension size.

Distributed computing architectures address these constraints by leveraging model parallelism, distributing the memory and computation demands of attention across multiple GPUs.

Recent algorithmic innovations have substantially improved the efficiency of attention computation without altering its theoretical complexity. For example, FlashAttention [18] optimizes memory access patterns through fused kernel operations implemented in specialized computing frameworks such as Triton [19]. These practical optimizations reduce memory requirements significantly while preserving computational performance.

2.1.3 Prompt Compression

Prompt compression methods aim to reduce input dimensionality by exploiting redundancy within input sequences, ideally preserving semantic content. However, this approach introduces a fundamental limitation in protein sequence analysis: compressed inputs cannot generate detailed per-residue embeddings for every amino acid, as compression inherently reduces sequence representation granularity. Consequently, prompt compression is less suitable for tasks requiring residue-level detail, such as protein structure prediction or mutation effect prediction.

2.1.4 Attention Matrix Approximation

An alternative approach to managing extended contexts involves approximating rather than explicitly computing the full attention matrix. This strategy includes two primary methodologies: context-agnostic and context-aware sparse matrices, each offering distinct trade-offs between computational efficiency and representational capacity.

Context-Agnostic Sparse Matrices. Context-agnostic approaches address the quadratic complexity of traditional self-attention through systematic approximation techniques [13]. Among the most successful implementations are architectures leveraging predefined sparse attention patterns, exemplified by Longformer, LongLoRA, and BigBird [20, 21, 22], each employing distinct sparsification strategies (Figure 1). Longformer implements dilated sliding-window attention, strategically positioning sparse attention windows around tokens (Figure 1a). LongLoRA extends context length by introducing shifted attention patterns during fine-tuning (Figure 1b). BigBird employs a hybrid approach combining global attention for select tokens, sliding-window attention, and random attention connections (Figure 1c). While BigBird enables whole-sequence consideration, the stochastic nature of random attention introduces variability in performance, as randomly selected attention positions lack inherent semantic or functional relevance. Although these approaches successfully extend context windows, their predetermined patterns remain independent of sequence content and semantic meaning, presenting inherent limitations. Most notably for biological applications, these methods cannot incorporate domain-specific knowledge—such as experimentally validated protein–protein interactions or genomic relationships—into attention calculations.

Context-Aware Sparse Matrices. In contrast to context-agnostic methods, context-aware compression approaches dynamically adjust their attention patterns based on input content. For example, the Routing Transformer [23] employs K-means clustering on key and query vectors to construct sparse, content-dependent attention matrices.

However, the exclusive reliance on clustering for routing attention presents significant limitations when analyzing biological sequences. Crucially, clustering-based methods may fail to capture interactions between functionally related but structurally and sequentially distinct elements, such as complementary protein domains that co-evolved despite limited sequence similarity.

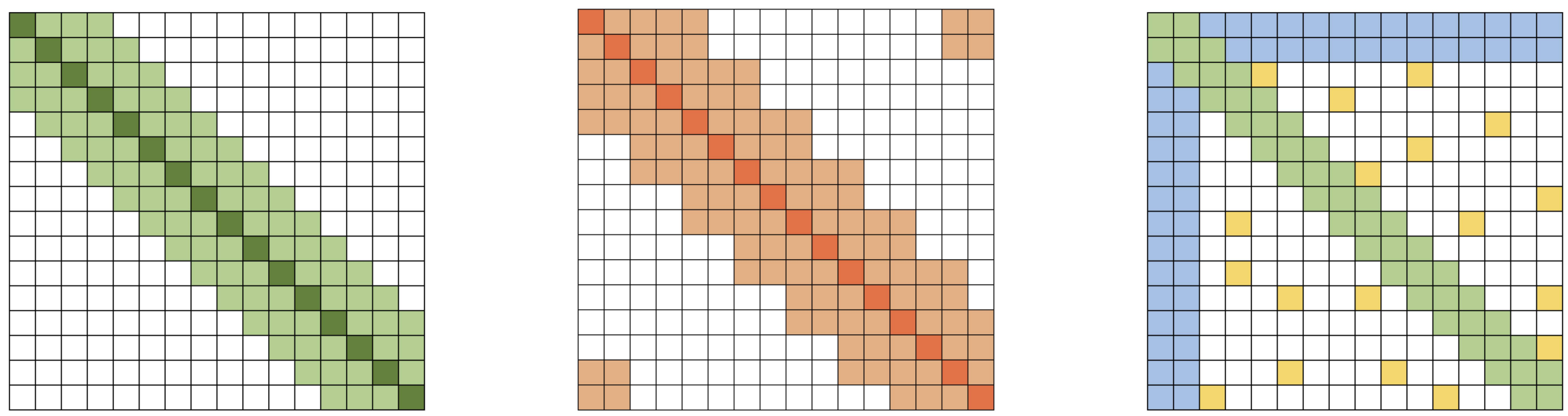

(a) Longformer (b) LongLoRA (c) BigBird

Figure 1: **Sparse attention patterns implemented in context-agnostic transformer architectures.** **(a)** Longformer's dilated sliding-window attention pattern, showing a diagonal band of attention with varying intensities of green. **(b)** LongLoRA's shifted attention pattern during fine-tuning, displayed as orange blocks. **(c)** BigBird's hybrid approach combining global attention (blue columns), sliding-window attention (green diagonal), and random attention connections (scattered yellow squares). These predetermined sparse attention patterns reduce computational complexity while enabling longer context windows, though they operate independently of sequence content and semantic meaning.

2.2 Protein & Genome Language Modeling

2.2.1 Protein Language Models

Current popular protein language models (pLMs), including ProtBert [24], ProtTrans [4], and ProGen2 [5], utilize transformer-based architectures with context windows ranging from 512 aa (ProtTrans-XL) to 2,048 aa (ProGen2). These context limitations have largely confined pLMs to analyzing individual proteins in isolation, neglecting their broader genomic context. Among these, the ESM-2 model [3], trained on the UniRef50 database [25] via masked language modeling, has emerged as one of the most widely adopted pLMs. ESM-2 demonstrates impressive performance in protein contact prediction—attributed to its effective attention mechanism—and generates biologically meaningful embeddings useful across diverse downstream applications [6, 7, 8]. However, ESM-2, like its predecessors, focuses exclusively on single-protein sequences with a restricted context length of only 1,024 aa, thereby limiting its ability to capture inter-protein relationships within genomic contexts.

To integrate genomic context into protein representations, the Protein Set Transformer (PST) [26] introduces a graph-based framework wherein proteins, initially embedded via ESM-2, form nodes connected by edges representing genomic proximity. Despite these advantages, PST relies on precomputed single-protein ESM-2 embeddings and is constrained to modeling interactions between consecutive proteins.

2.2.2 Genomic Language Models

Recognizing the inherent limitations of analyzing proteins in isolation has spurred the development of genomic language models (gLMs) [27], which augment ESM-2 embeddings by incorporating context from larger genomic regions. For instance, recent gLMs utilize 30-gene fragments in a masked prediction task, enabling the prediction of protein embeddings by considering the contextual influence of 29 neighboring proteins. This approach generates enriched representations that significantly improve performance across tasks such as protein function prediction and taxonomic classification. However, existing gLMs primarily utilize conventional attention mechanisms, highlighting potential opportunities for incorporating biologically informed sparse attention patterns to further enhance their capabilities.

While existing methods have significantly advanced general language modeling and biological sequence analysis, important limitations remain. Current context-extension techniques in language models typically lack biological specificity. Although extrapolation methods such as ALiBi demonstrate effectiveness in general language tasks, they inherently assume consistent structural patterns between short and extended sequences (for instance, local grammatical structures remain stable regardless of sequence length). This assumption does not hold for multi-protein sequences, where relationships between distant proteins can fundamentally differ from the local sequence patterns learned by single-protein models. Additionally, while content-dependent sparse attention mechanisms show promise for managing extended contexts, their potential for explicitly incorporating biological priors into language model architectures remains largely unexplored.

To address these limitations, we introduce a novel genome-scale protein language model architecture specifically designed for viral sequences. Our approach synthesizes recent advances in context-length extension methods with biologically informed sparse attention mechanisms, enabling the effective processing of significantly longer amino acid sequences on commodity hardware while explicitly modeling inter-protein interactions. The following sections detail our methodology and validate its effectiveness across multiple downstream biological tasks.

3 Methods

3.1 Datasets and Preprocessing

Sources and retrieval. We used the NCBI Virus database (retrieved 2024-06), selecting only genomes annotated as "complete" (14,436 genomes) (Supplementary Materials S1, Supplementary Figure S1). The list of loci used is included in the Github page (`https://github.com/Hawaii-Bioinformatics/ViralEmbed`). For downstream classification tasks, the final dataset comprised 100,000 proteins from 5,000 genomes after de-duplication.

Protein non-redundancy. To prevent highly abundant sequences from biasing training, we applied CD-HIT clustering at 90% sequence identity, reducing the dataset by approximately 50%. Some viral proteins occur dozens or hundreds of times in the database; without deduplication, such sequences would dominate gradient updates and bias learned representations. This threshold collapses near-identical sequences while preserving biologically distinct variants.

Splits. To prevent data leakage, we partitioned the dataset at the genome level: all proteins from a given genome were assigned to the same split (train, validation, or test). Splits were stratified by taxonomic species to maintain balanced representation across partitions. The final proportions were 80% train, 10% validation, and 10% test. This design ensures that no protein from a genome in the test set could have been seen during training, either directly or through related proteins from the same genome.

Fragments. For long-context baselines, we constructed fragments by concatenating proteins in genomic order with separators, truncating or padding to target lengths as specified.

Metadata. For downstream labels we used (i) NCBI taxonomy (species/family), and (ii) Pfam domain family assignments from HMMER3 against Pfam 35.0 to create task-aligned labels used in Sections 3.8 and 3.9.

3.2 Training and Evaluation Pipeline Overview

Our approach involves three distinct stages, each employing different models (Figure 2):

Stage 1 – PPI Inference Model (ESM-2-PPI): Training protein language models on entire viral genomes is computationally demanding due to the quadratic scaling of attention mechanisms with sequence length. While existing sparse attention approaches can capture co-evolutionary signals, they typically use static or random masking patterns that neglect biological context. To overcome this, we develop a biologically informed sparse attention method that restricts attention computations exclusively to protein pairs identified as likely interaction partners. Ideally, experimental PPI databases would guide this identification; however, the limited coverage of viral genomes in these databases necessitated a computational strategy. Specifically, we fine-tune ESM-2 (650M parameters) with ALiBi positional encoding (selected after comparing against RoPE; see Supplementary Materials), LoRA adaptation, and Longformer-style sliding window attention (selected after comparing against Shifted Sparse Attention and BigBird; see Supplementary Materials). This extends the effective context window to approximately 13,000 aa, enabling analysis of multi-protein genomic fragments. This model is used exclusively for extracting attention scores to infer PPIs and is not employed for any downstream embedding or classification tasks. Section 3.3 describes the optimization of this model, and Section 4.1 presents the results.

Stage 2 – Genome-Scale Language Models (LV-3C and LV-5B): Using the PPIs identified in Stage 1 as biologically informed sparsity priors, we train two novel long-context protein language models. Both models use identical sparse attention patterns; they differ in scale and initialization:

- **LV-3C** (317M parameters; 16 layers): Trained from scratch on sequences up to 61,000 aa. Its lighter architecture prioritizes processing longer genomic context.
- **LV-5B** (756M parameters; 33 layers): Initialized from ESM-2 (650M) weights and trained on sequences up to 48,000 aa. Its larger architecture prioritizes richer pretrained representations.

This complementary design allows us to evaluate two strategies for incorporating genomic context: maximizing sequence length versus leveraging pretrained knowledge. These models represent the primary contribution of this work and are described in Sections 3.3–3.4.

Stage 3 – Baseline Models for Evaluation: To benchmark LV-3C and LV-5B, we compare against several ESM-2 variants:

- **ESM-2-pt**: The original pretrained ESM-2 model with a 1,024 aa context window.
- **ESM-2-RoPE**: ESM-2 fine-tuned with RoPE positional encoding for extended context inference.
- **ESM-2-ALiBi**: ESM-2 fine-tuned with ALiBi positional encoding for extended context inference.

These baselines isolate the contribution of our biologically informed sparse attention mechanism from general context extension strategies.

Throughout the manuscript, we use these standardized names to refer to each model variant. The following sections describe each stage in detail: Section 3.3 covers Stage 1 (PPI inference), Sections 3.4–3.5 cover Stage 2 (model training), and Sections 3.6–3.11 cover Stage 3 (evaluation).

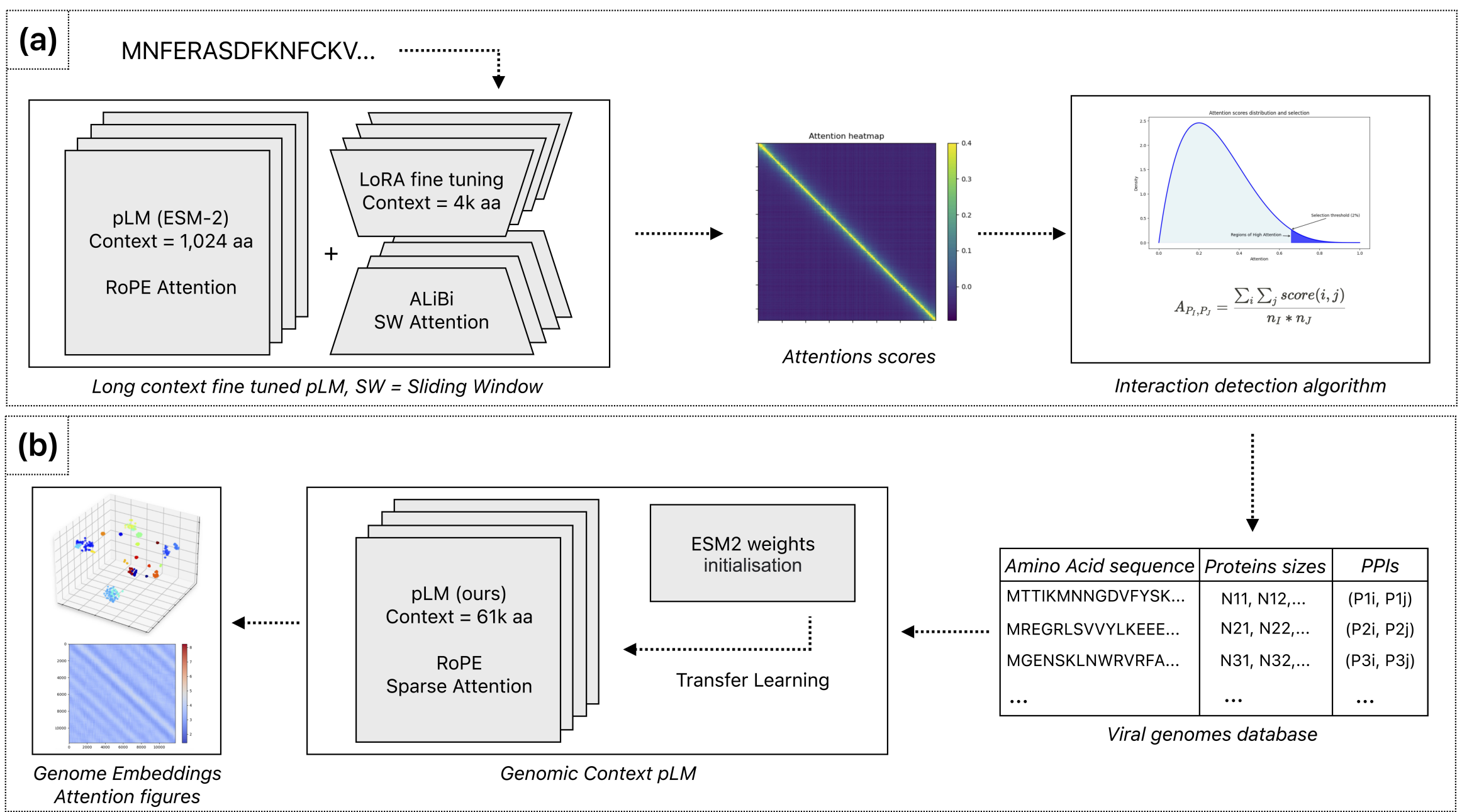

Figure 2: **Two-stage approach for training a protein language model on complete viral genomes.** **(a)** Identifying putative protein-protein interactions on multi-protein fragments using an extended-context ESM-2 model combining LoRA fine-tuning and ALiBi positional extrapolation. The attention scores produced by this model are subsequently used to inform the protein interaction detection algorithm. **(b)** Training of two long-context protein language models from scratch or initialized from ESM-2 weights using sparse attention patterns guided by inferred protein interactions, enabling efficient whole-genome embedding generation.

3.3 Developing the PPI Inference Model (Stage 1)

To extend ESM-2's context for PPI inference, we evaluated multiple approaches for achieving a context window of 13,000 aa (Supplementary Materials S2). Specifically, we compared two positional encoding strategies (RoPE and ALiBi) (Supplementary Figure S2) and three sparse attention mechanisms (Longformer, Shifted Sparse Attention, and BigBird) (Supplementary Table S1). Performance was benchmarked against the baseline ESM-2 650M model using both perplexity, which quantifies how well the model predicts amino acid sequences (lower is better), and silhouette scores, which measure how well protein embeddings cluster by structural similarity (higher is better).

PPIs can be inferred from attention scores between proteins in language models, as demonstrated by previous work. For instance, [28] showed that specific attention heads in ESM-1b capture distinct protein relationships, and subsequent work extended these findings to larger models including ESM-2 [3]. Our approach builds upon this concept by

analyzing attention patterns simultaneously across multiple proteins within viral genomes to rank putative interaction links. Standard protein language models, such as ESM-2, have a context length limitation of 1,024 aa, restricting analysis to only 3–5 average-length proteins at once. To overcome this, we first extended ESM-2's context window to 4,000 aa using attention matrix approximation, and subsequently increased it further to 13,000 aa through context length extrapolation (ALiBi). For the initial extension, ESM-2 was fine-tuned on concatenated protein fragments from the NCBI Viral database [14] using an NVIDIA A6000 GPU with 48 GB RAM. Protein fragments were constructed by sequentially concatenating complete proteins in their genomic order until reaching the 4,000 aa threshold, padding as necessary. To enable efficient training on standard GPU hardware, we used LoRA for parameter-efficient fine-tuning, while Longformer was selected as the sparse-attention pattern based on the comparisons in Supplementary Table S1.

Interaction Threshold Selection. A key parameter in our sparse attention mechanism is k, the number of top-ranked non-adjacent protein pairs included in the attention mask for each genome. To select an appropriate value, we varied $k \in \{10, 20, 35, 50, 100\}$ and evaluated downstream performance on taxonomy and Pfam classification tasks (Supplementary Materials S2). We also tested an alternative policy that includes genomically adjacent pairs in the ranking. Supplementary Table S2 shows that $k = 50$ maximizes F1 score with modest computational cost, while including adjacency during selection provides no benefit since consecutive protein attention is already explicitly included in the sparse pattern (see Section 3.4).

For each protein fragment analyzed by the fine-tuned model, attention matrices from all encoder layers were extracted. For each layer ℓ and attention head h, we denote the token-level attention matrix as $M^{(\ell,h)} \in \mathbb{R}^{L\times L}$ and use p, q, r, s for token positions. Each matrix was normalized and symmetrized using Average Product Correction (APC), yielding $N^{(\ell,h)}$ computed as [3]:

$$\begin{aligned} APC^{(\ell,h)}_{p,q} &= \frac{\sum_{s=1}^{L} M^{(\ell,h)}_{p,s} \sum_{r=1}^{L} M^{(\ell,h)}_{r,q}}{\sum_{r=1}^{L}\sum_{s=1}^{L} M^{(\ell,h)}_{r,s}}, \\ \widetilde{M}^{(\ell,h)}_{p,q} &= M^{(\ell,h)}_{p,q} - APC^{(\ell,h)}_{p,q}, \\ N^{(\ell,h)}_{p,q} &= \frac{1}{2}\left(\widetilde{M}^{(\ell,h)}_{p,q} + \widetilde{M}^{(\ell,h)}_{q,p}\right). \end{aligned}$$

Here, ℓ indexes layers, h indexes attention heads, and $p, q, r, s \in \{1, \ldots, L\}$ index amino-acid positions within the sequence.

From each normalized attention matrix $N^{(\ell,h)}$, we define a set $S^{(\ell,h)}$ containing the top $t_{\text{aa}}\%$ highest attention values between amino-acid pairs. We used $t_{\text{aa}} = 2$, corresponding to the top 2% of amino-acid attention values; this amino-acid-level threshold is separate from the final protein-pair threshold $k = 50$:

$$S^{(\ell,h)} = \{(p,q) \mid N^{(\ell,h)}_{p,q} > \text{Quantile}_{1-\frac{t_{\text{aa}}}{100}}(N^{(\ell,h)})\}.$$

Attention values within the set S were subsequently converted to scores via min-max normalization. These normalized scores were then aggregated to compute an attention-based interaction score A_{P_I,P_J} between protein pairs P_I and P_J, having lengths n_I and n_J, respectively:

$$A_{P_I,P_J} = \frac{\sum_{p\in P_I}\sum_{q\in P_J} \text{score}(p,q)}{n_I \times n_J} \quad \text{with} \quad (p,q) \in S^{(\ell,h)}.$$

Interaction scores were computed for all protein pairs within the genome. Preliminary analyses indicated that genomically adjacent proteins consistently yielded the highest attention scores (Supplementary Figure S3), likely reflecting genuine biological interactions. Therefore, for constructing the sparse attention matrix used in subsequent model training, only the top 50 ranked scores from non-consecutive protein pairs were included as interacting proteins, with the remaining pairs masked. This threshold of 50 interactions represents a balance between computational efficiency and interaction coverage appropriate for the viral genome sizes analyzed (Supplementary Figure S4). The threshold parameter can be adjusted based on genome sequence length and available computational resources.

To calibrate the number of protein pairs selected for sparse attention on biologically relevant data, we investigated interaction patterns among Family A DNA polymerase (PolA), ribonucleotide reductase (RNR), and helicase (HEL) (Supplementary Figures S3, S4, Supplementary Table S2). These proteins constitute a well-characterized interaction network [29] and display strong evolutionary coupling due to their coordinated roles in DNA replication [30, 31].

3.4 Sparse Attention Computation (Stage 2)

Once the PPIs have been identified and selected using the mechanism described above, it is possible to proceed with training a long-context pLM. Model training is based on a sparse attention mechanism, described below, and represented in Figure 3.

To maintain biological fidelity when computing cross-protein attention, we developed a two-stage fragmentation approach. First, we segment the viral genome precisely at protein boundaries, preserving each protein as a distinct unit. Second, we subdivide each protein into uniform blocks of fixed length B, resulting in a block-level representation of the viral genome. We then map the previously identified PPIs onto these blocks, creating fragment-level interaction pairs that guide our sparse attention model. This transformation preserves biologically meaningful relationships while enabling efficient computation across the entire viral genome.

Specifically, given two interacting proteins P_1 and P_2, we transfer these interactions to interactions between their constituent blocks:

$$(P_1, P_2) \Rightarrow (B_{1_i}, B_{2_j}), \quad i \in [1, n_1],\ j \in [1, n_2]$$

where:

- P_1, P_2 are two interacting proteins from genome G.
- $B_{1,i}$ is the i-th block of protein P_1, with n_1 total blocks.
- $B_{2,j}$ is the j-th block of protein P_2, with n_2 total blocks.

For each pair of blocks $(B_{1,i}, B_{2,j})$, we compute the block-level attention scores using the scaled dot-product attention formula:

$$Att_G(B_{1,i}, B_{2,j}) = \frac{Q_{B_{1,i}} K^T_{B_{2,j}}}{\sqrt{d_k}}$$

where:

- $B_{1,i}$, $B_{2,j}$ are the blocks from genome G being considered
- $Q_{B_{1,i}}$ is the query vector subset from genome G corresponding to block $B_{1,i}$.
- $K_{B_{2,j}}$ is the key vector subset from genome G corresponding to block $B_{2,j}$.
- d_k is the dimensionality of the key vectors.

Efficient computation of sparse attention requires specialized data structures optimized for block operations. We utilize the Block Sparse Row (BSR) tensor format [32], which stores non-zero elements in fixed-size blocks, significantly reducing both memory consumption and computational overhead. Empirical testing identified a block size $B = 32$ as optimal, balancing computational efficiency and minimal padding overhead in our viral protein datasets.

In our implementation, each query–key product $Q_{F_i}(K_{F_j})^T$ between fragment pairs $B_{1,i}$ and $B_{2,j}$ produces a 32×32 tensor, directly aligning with BSR's block structure. We subsequently apply softmax normalization followed by multiplication with the corresponding value vectors using optimized Triton [19] kernels tailored for block-sparse operations. Finally, we remove padding tokens to restore original sequence dimensions using precomputed position indices (Algorithm 1).

3.5 Model Training (Stage 2)

The training was conducted using the NCBI Virus database (retrieved on 06/2024), comprising 14,436 complete viral genomes totaling 683 million aa (Supplementary Figure S1). The database represents a comprehensive reference set of viral diversity, ensuring robust generalization across viral families. All genomes were segmented at protein boundaries and padded appropriately to meet sequence length constraints. All training was conducted using NVIDIA A6000 GPUs (48 GB RAM) with block-sparse computations optimized through the Triton library [19].

The long-context protein language models represent two main variants, designed to produce complementary types of embeddings. These two models differ in scale, with LV-3C being the lighter version (317M parameters) and LV-5B the larger one (756M parameters).

The two models also differ in their initialization and training strategies:

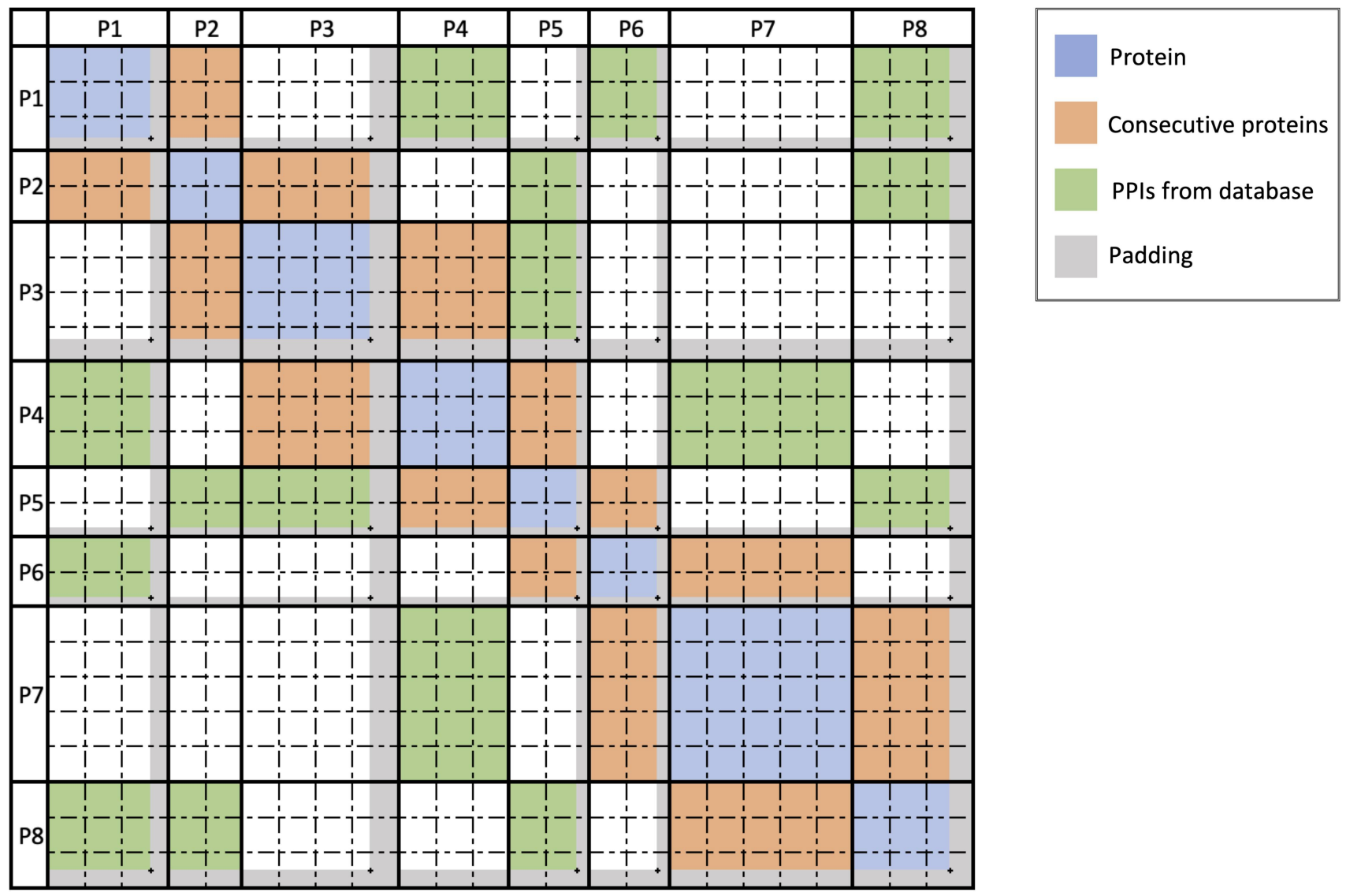

Figure 3: **Sparse attention computation architecture.** Example attention matrix demonstrating four distinct protein interaction patterns considered during sparse attention computation: intra-protein (self) attention (blue), interactions between consecutive proteins (orange), putative protein–protein interactions identified by our method (Section 3.3) and stored in a database (green), and protein pairs not identified as interacting (white), for which attention calculations are omitted. Gray regions indicate padding introduced during fragment construction.

- LV-3C: Randomly initialized and trained from scratch on genomic segments (up to 61,000 amino acids) with an initial learning rate of 3×10^{-4}, using biologically informed sparse attention for 12 epochs.
- LV-5B: Trained leveraging transfer learning from the ESM-2 model (650M parameters) on genomic segments (up to 48,000 amino acids), using the same biologically informed sparse attention with an initial learning rate of 3×10^{-5}, also for 12 epochs.

This two-model design aims to capture complementary aspects of viral genomic information. LV-3C prioritizes the modeling of long-range genomic context through a lighter architecture that enables the processing of extended sequences, whereas LV-5B prioritizes richer pretrained representations through a larger model operating on shorter segments.

3.6 Evaluation Metrics (Stage 3)

We employ two primary metrics throughout our evaluations:

Perplexity measures how well the model predicts amino acid sequences, quantifying the model's uncertainty when predicting masked tokens. It is defined as the exponentiated average cross-entropy loss:

$$\text{Perplexity} = \exp\left(-\frac{1}{N}\sum_{i=1}^{N}\log P(x_i|\text{context})\right)$$

where N is the number of masked tokens and $P(x_i|\text{context})$ is the predicted probability of the true amino acid x_i. Lower perplexity indicates better predictive performance. This metric is particularly important for assessing whether models maintain consistent prediction quality as sequence length increases.

Silhouette score evaluates embedding quality by measuring clustering coherence. For a given embedding i with cluster label a, the silhouette score is:

$$s(i) = \frac{b(i) - a(i)}{\max(a(i), b(i))}$$

where $a(i)$ is the mean distance to other points in the same cluster and $b(i)$ is the mean distance to points in the nearest neighboring cluster. Scores range from -1 to 1, where higher values indicate better-defined clusters. We use this

Algorithm 1 Algorithm for computing biologically informed sparse attention, from input tensors through block-wise operations to final output.

Entries: Queries, Keys, and Values: $Q, K, V \in \mathbb{R}^{n \times d}$
Protein interactions $P_I \in \mathbb{R}^{i \times 2}$, Protein sizes $P_S \in \mathbb{R}^N$

$Q, K, V \leftarrow$ reshape_tensor_padding(Q, K, V, P_S, B) ▷ Split genome into proteins, apply padding if necessary, then into B-size blocks: $X(bs, n_b, B, d) \leftarrow X(bs, n, d)$

$Q, K, V \leftarrow$ transpose_for_scores(Q, K, V, n_h, h_s) ▷ Transpose tensors for attention computation: $X(bs, n_h, h_s, n_b, B) \leftarrow X(bs, n_b, B, d)$

$BI \leftarrow$ chunk_proteins_padding(PI, PS) ▷ Transform protein interactions into block interactions

$Q, K \leftarrow$ rotary_embeddings(Q, K) ▷ Apply rotary positional embeddings

$Att \leftarrow$ sparse_attention_matrix(Q, K, BI) ▷ Compute attention scores and construct BSR tensor
$Att \leftarrow$ bsr_softmax(Att)
$Att \leftarrow$ bsr_dropout$(Att,$ dropout$)$
$V \leftarrow$ bsr_dense_mm(Att, V)

$V \leftarrow$ revert_padding(V) ▷ Reverse padding and reshape into final dimensions : $V(bs, n, d) \leftarrow V(bs, n_b, B, d)$

return V

metric to assess whether embeddings group biologically related sequences (e.g., by sequence similarity or taxonomic classification).

3.7 Evaluating Global Performance & Protein Embeddings Quality

We evaluated our biologically informed sparse attention models (LV-3C and LV-5B) against ESM-2 pre-trained (ESM-2-pt) and ALiBi fine-tuned variant (ESM-2-ALiBi) using perplexity and clustering silhouette scores. Perplexity assessed token prediction accuracy over extended contexts, while the silhouette scores evaluated the quality and information content of protein sequence embeddings. We assessed model performance using viral genome fragments ranging from 500 to 20,000 aa. Although our models support sequences up to 61,000 aa, we limited evaluations to 20,000 aa to accommodate the computational constraints (time and memory) associated with full attention when running on a single GPU (NVIDIA A6000 with 48 GB of RAM).

Sequence-based clustering reference. To evaluate whether model embeddings preserve sequence relationships, we clustered proteins using MMseqs2 at 80% sequence identity. These clusters serve as reference labels for computing silhouette scores: high silhouette scores indicate that proteins similar in sequence are also proximal in embedding space. This provides a baseline assessment of embedding quality independent of functional or taxonomic annotations.

3.8 Evaluating Genome Embeddings Quality

We assessed the quality of genome-scale embeddings produced by our models using taxonomic clustering and species classification. To further evaluate embedding utility, we trained a single-layer classifier to identify species from genome embeddings (details provided in Supplementary Material S3).

3.9 Evaluation of Embedding Quality for Classification

We evaluated the models' contextual embeddings by assessing their performance on species clustering and classification tasks (see also Supplementary Material S3; Pfam/domain-family validation is described separately in Supplementary Material S4), as well as by analyzing the similarity distributions obtained from comparing pairs of embeddings.

- **Evaluation of taxonomic information preservation:** To evaluate how effectively different models preserve taxonomic information, we analyzed clustering patterns of protein embeddings derived from 100 distinct viral species.

- **Analysis of species clustering efficiency:** Next, we trained a single-layer taxonomic classifier designed to predict the species identity of individual proteins directly from their embeddings. To systematically assess classification performance, we varied the number of species classes from 5 up to 100 species. We measured classifier performance using F1-score averaged over a 5-fold cross-validation scheme.

- **Analysis of embedding variance and distribution:** To further characterize the differences among embedding spaces produced by various models, we randomly selected 5,000 proteins from over 1,000 viral genomes and computed pairwise Euclidean distances between their embeddings. We then compared the resulting distributions from embeddings generated by ESM-2 variants and our two proposed models, LV-5B and LV-3C.

3.10 Evaluating Across-Model Attention

The following two sections evaluate the attention patterns of the trained LV-3C and LV-5B models, as distinct from the Stage 1 PPI inference used to build the sparse-attention priors: they assess whether the trained models—having been guided by these priors during training—retain and recapitulate biologically meaningful PPI signal in their final attention. To this end, we analyzed attention patterns across the entire network architecture. Attention scores from all heads in each layer were extracted to examine each model's ability to detect and represent interactions spanning various distances within the genome sequence.

We categorized attention interactions into three distinct proximity classes:

- **Short-range interactions**: Attention between amino acids positioned within 100 residues of each other, typically representing intra-protein or intra-domain relationships.

- **Medium-range interactions**: Attention between amino acids separated by 100–500 residues, generally capturing interactions between domains or adjacent proteins.

- **Long-range interactions**: Attention between amino acids separated by more than 500 residues, predominantly representing inter-protein interactions between non-adjacent proteins.

For each category, we calculated the mean attention score across multiple viral genomes, enabling comprehensive analysis of the model's attention distribution patterns.

3.11 Validation Against Experimentally Curated Interactions

To validate the biological relevance of model-derived attention patterns, we benchmarked attention-derived protein-pair scores, interpreted as putative interaction links, against experimentally validated interactions documented in the STRING database [33]. Attention scores were extracted from all heads and layers, and protein pairs were ranked by their aggregate long-range attention score following [28]. Validation encompassed 146 annotated interactions across five distinct viral genomes. STRING interactions with confidence ≥ 0.500 served as positive examples; negative examples consisted of all other protein pairs from the same genomes not reported as interacting in STRING. We note that this negative set likely contains some true interactions not yet experimentally characterized, making our reported metrics conservative estimates.

Data and Code Availability

All source code, long-context inference scripts, and pair-ranking utilities are available at `https://github.com/Hawaii-Bioinformatics/ViralEmbed`. Example inputs are provided in `large_context/scripts/data/`, including a test viral proteome FASTA (`LR699048.fa`) and a matching protein-labels text file (`LR699048.txt`) used for pair-ranking demonstrations.

Attention tensors are generated locally using the provided inference script (see `large_context/scripts/large_prot_encoding/inference.py` instructions in the scripts README).

A worked "realistic use case" notebook is included in `large_context/scripts/data/realistic_use_case/use_case.ipynb`. To reproduce the results, download the NCBI Virus snapshot used in this study (14,436 complete genomes; retrieved 2024–06) and regenerate the attention tensors and downstream analyses using the provided scripts.

4 Results

4.1 Developing the PPI Inference Model (Stage 1)

Before training our genome-scale models LV-3C and LV-5B, we first developed ESM-2-PPI, a separate auxiliary model used exclusively to derive the protein–protein interaction priors that guide the sparse attention mechanism. ESM-2-PPI extends pretrained ESM-2 (650M) to a 13,000 aa context window via LoRA fine-tuning, sparse attention, and ALiBi positional extrapolation. Because the choice of positional encoding and sparse-attention pattern directly affects the reliability of the attention scores subsequently used as priors, we evaluated two positional encoding strategies (RoPE, ALiBi) (Supplementary Figure S2) and three sparse attention mechanisms (Longformer, Shifted Sparse Attention, BigBird) (Supplementary Table S1) on held-out viral genome fragments ranging from 500 to 13,000 aa, requiring both stable perplexity across extended sequence lengths and embeddings that preserve biological relationships. ALiBi with Longformer attention achieved the best balance of perplexity stability (1.99 across all tested lengths), clustering quality (silhouette score: 0.88), and computational efficiency.

To validate that ESM-2-PPI captures biologically meaningful interactions, we analyzed attention patterns among Family A DNA polymerase (PolA), ribonucleotide reductase (RNR), and helicase (HEL)—proteins with well-characterized co-evolutionary relationships [29, 30, 31]. These known interacting pairs consistently ranked among the top 50 non-adjacent protein pairs across 83 viral genomes (Supplementary Figures S3, S4), supporting our selection of $k = 50$ as the interaction threshold.

This validation serves only to calibrate the PPI selection threshold for ESM-2-PPI and the PolA/RNR/HEL interactions were not used during training of LV-3C or LV-5B, which learn from PPIs inferred independently across all genomes in the training set.

4.2 Evaluating Global Performance & Embeddings Quality

This section evaluates our two genome-scale protein language models LV-3C (317M parameters, trained from scratch on sequences up to 61,000 aa) and LV-5B (756M parameters, initialized from ESM-2 weights and trained on sequences up to 48,000 aa) against three ESM-2 baselines (ESM-2-pt, ESM-2-RoPE, ESM-2-ALiBi). Both LV models share the same biologically informed sparse attention mechanism and differ only in scale and initialization, allowing us to disentangle the contribution of context length from that of pretrained knowledge.

The LV-3C and LV-5B models demonstrated significantly lower perplexity (uncertainty when predicting masked tokens) compared to ESM-2 variants on sequences longer than 3,000 aa (Figure 4a), maintaining this advantage consistently across all tested lengths. In contrast, both the pre-trained ESM-2-pt (gray) and its RoPE-fine-tuned variant ESM-2-RoPE (red) experienced significant perplexity inflation when evaluated beyond their original training context windows. Fine-tuned version of ESM-2-ALiBi (orange) maintains near-constant perplexity with sequence length, but higher than our models.

For clustering quality analysis, we clustered protein sequences and fragments using MMseqs2 [34] at an 80% similarity threshold and computed silhouette scores to quantify embedding coherence. Our results revealed clear differences in clustering performance among the tested models (Figure 4b).

Both LV models provided superior embedding coherence and better discrimination of protein sequences compared to baseline approaches. LV-3C (light blue) exhibited robust clustering quality, particularly for longer sequences (silhouette score: 0.83), while LV-5B (dark blue) achieved performance comparable to ESM-2-pt and ESM-2-RoPE for shorter sequences and maintained high effectiveness at extended lengths (silhouette score: 0.67). ESM-2-pt (gray) excelled only within its original training range (1,024 aa), with embedding quality markedly declining for longer, multi-protein sequences. While ESM-2-ALiBi (orange) extended effective clustering performance up to approximately 4,000 aa, embedding quality still deteriorated as sequence lengths increased, despite the positional encoding extrapolation strategy.

4.3 Evaluating Genome Embeddings Quality

The quality of the genomic embeddings was assessed by downstream performance in a clustering and a classification task. We employ two complementary evaluations: silhouette scores assess embedding coherence, whether proteins from the same taxonomic species cluster together in latent space, while classification accuracy assesses whether embeddings encode sufficient discriminative information for species prediction. These metrics capture related but distinct properties: coherent embeddings facilitate classification, but similar classification performance can arise from different embedding geometries. Silhouette scores (Figure 5a) indicated strong taxonomic discrimination across all models, with the LV-5B variant consistently outperforming others.

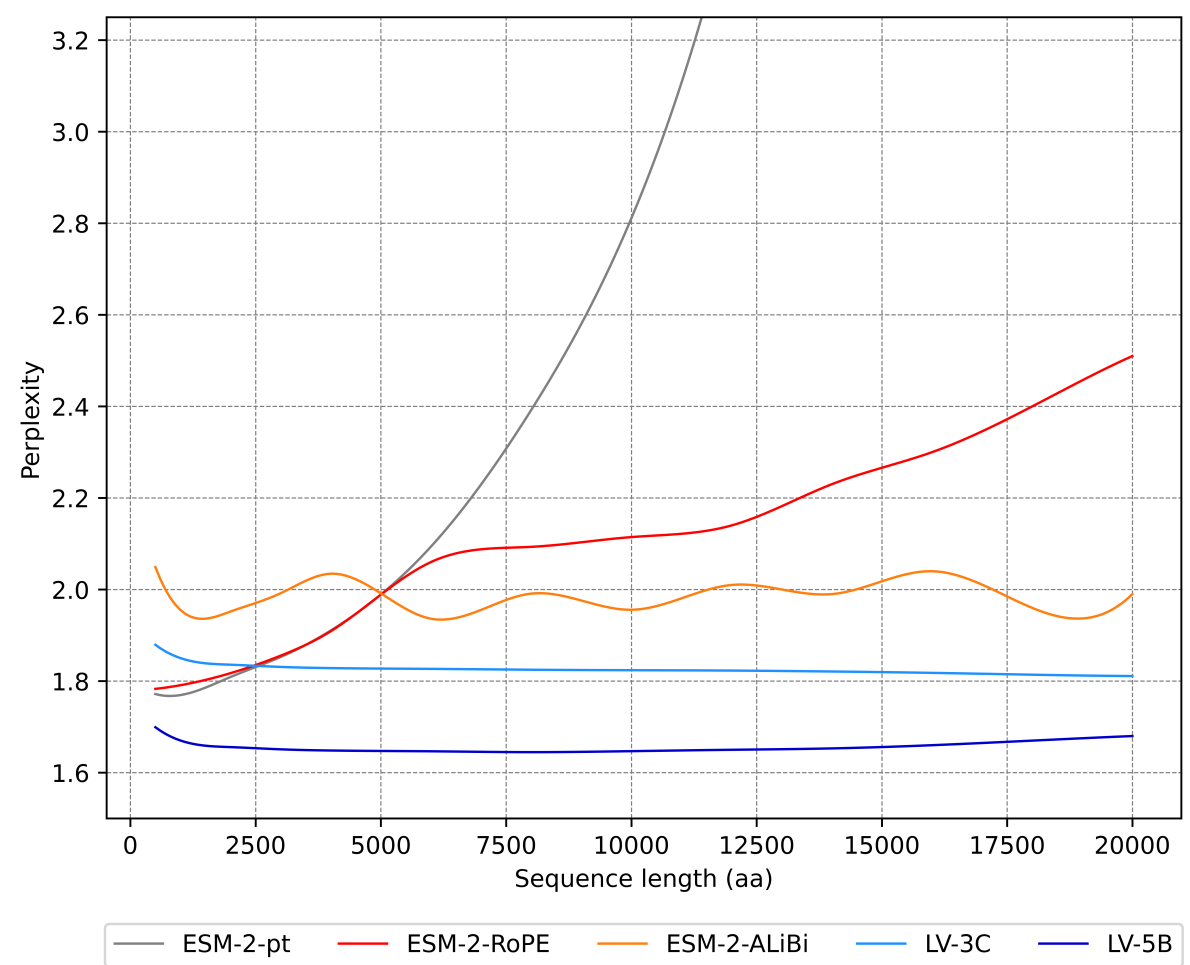

(a) Perplexity

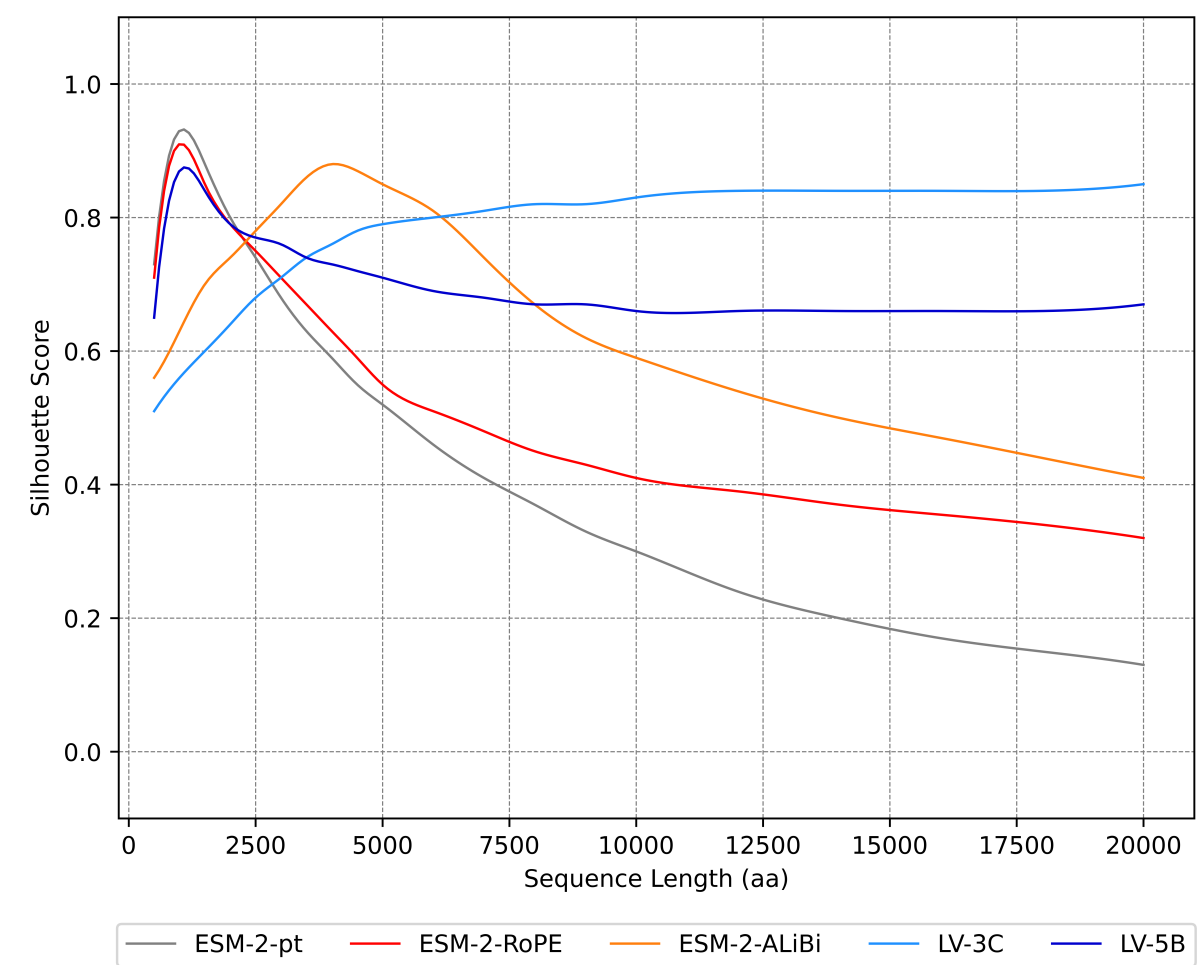

(b) Silhouette Score

Figure 4: **Global performances of our models compared to ESM-2 variant baselines. (a)** Perplexity evaluation of ESM-2-pt, ESM-2-RoPE, ESM-2-ALiBi, and our biologically informed sparse attention models LV-3C & LV-5B. **(b)** Clustering ability evaluation using silhouette score on fragment embeddings, using sequence similarity as reference with MMSeqs2 80% amino acid identity clustering. These smoothed curves are obtained from perplexity and silhouette score measurements for sequences ranging from 500 to 20,000 amino acids.

The performance of the species classification of genomes evaluated by F1-scores (Figure 5b) revealed a notable trend: as the number of species increased—and consequently, task complexity—embeddings generated using biologically informed sparse attention demonstrated superior classification accuracy relative to genome embeddings from fine-tuned ESM-2-ALiBi. This performance improvement at higher taxonomic resolution suggests our embeddings effectively capture nuanced genomic features, enhancing discriminative power at larger phylogenetic scales. These properties hold promising implications for phylogenetic and metagenomic applications.

4.4 Evaluation of Protein Embedding Quality for Classification

4.4.1 Analysis of Embedding Variance and Distribution

The quality of protein embeddings generated by ESM-2-ALiBi and proposed models LV-3C and LV-5B were assessed based on embedding variance and distribution of the pairwise Euclidean distances between 5,000 randomly selected protein from over 1,000 viral genomes. The analysis (Figure 6) revealed distinct variability patterns among these embedding spaces. Embeddings produced by ESM-2-ALiBi demonstrated the narrowest distribution, with the lowest interquartile range (1.16) and a relatively limited embedding space extent (range: 9.43). In contrast, embeddings from model LV-5B showed greater variability, with a broader interquartile range (1.58) and a larger embedding space (range: 15.69). Model LV-3C exhibited the most dispersed embeddings, spanning the widest embedding space (range: 16.18) and displaying significantly higher average pairwise Euclidean distances. These results indicate that incorporating genomic context into LV-3C substantially diversifies embedding representations, likely capturing biologically meaningful, genome-specific information.

Further analysis of the Euclidean distance across protein embeddings shows that context provides a stronger correlation with taxonomic distance (Figures 6, 7). Specifically, embeddings from proteins within the same species exhibit high similarity, characterized by low Euclidean distance. As the taxonomic distance between proteins increases, embedding similarity correspondingly decreases, highlighting the models' ability to capture biologically meaningful divergence across broader taxonomic categories.

4.4.2 Evaluation of Taxonomic Information Preservation

Given that protein embeddings inherently cluster primarily by protein type rather than species, silhouette scores based on taxonomy labels across all models were generally low (slightly negative). Despite this challenge, embeddings generated by the LV-3C model exhibited better species-specific clustering relative to those produced by ESM-2-pt and LV-5B (Figure 7a, Pfam-specific clustering in Supplementary Material S4 and Supplementary Figure S5). For

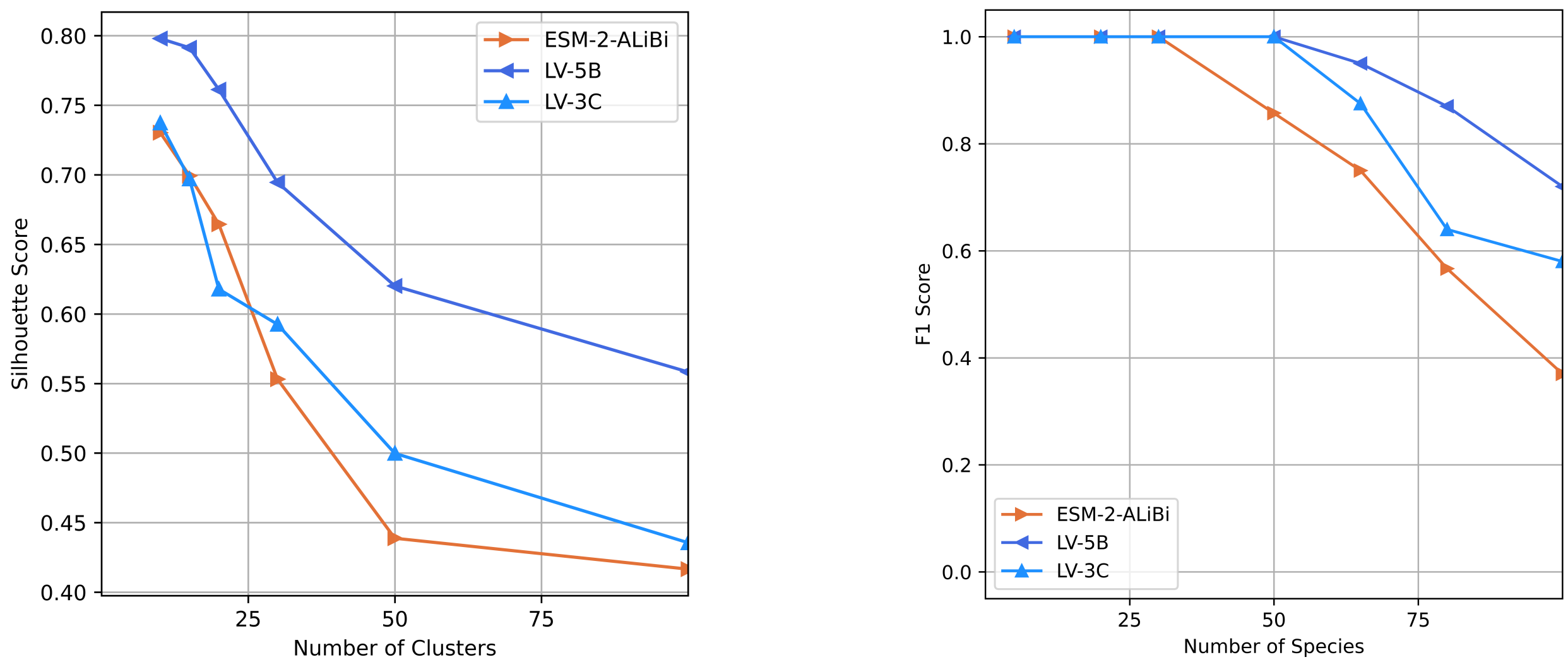

(a) Silhouette Score

(b) Classification

Figure 5: **Evaluation of full genome embeddings. (a)** Silhouette scores comparing genome embeddings generated by LV-3C, LV-5B, and ESM-2-ALiBi, using taxonomic species as cluster labels. **(b)** F1-scores for a single-layer classifier predicting species identity from genome embeddings (LV-3C, LV-5B, ESM-2-ALiBi). The x-axis varies the number of viral species used as classification targets, from 5 to 100; larger values denote finer-grained taxonomic resolution.

comparison, we included embeddings from the Protein Set Transformer (PST), a recent genome-level protein model, as an additional baseline. We note that PST's public release (v2.1.0) does not support genome-level embeddings, and its graph neural network architecture does not produce attention scores comparable to our transformer-based approach; PST is therefore included only in protein embedding evaluations where outputs are directly comparable. PST achieved the lowest silhouette scores, indicating limited capability in capturing viral species-level taxonomic relationships.

4.4.3 Analysis of Species Clustering Efficiency

As illustrated in Figure 7b, classification performance was the lowest when utilizing context-independent embeddings generated by the pre-trained ESM-2-pt. PST embeddings improved classification outcomes slightly, affirming that PST does capture genome-level context to some degree. However, LV-3C and LV-5B embeddings enriched with extensive genomic context further enhanced classifier accuracy across all evaluated class sizes. Among the tested models, LV-3C consistently achieved superior performance, reflected in the highest F1-scores for every class configuration.

4.5 Evaluating Across-Model Attention

Our proposed models were evaluated based on mean attention scores in short-range (within 100 aa, representing intra-protein or intra-domain relationships), mid-range (within 100-500 aa, representing inter-domain or adjacent protein relationships), and long-range ($>$ 500 aa apart, representing inter-protein interactions between non-adjacent proteins) interactions. The distribution of normalized attention scores across the three ranges provides insight into the model's capacity to capture biologically relevant interactions at different genomic scales. High variance in the attention score distribution indicates preferential focus on specific interaction ranges, while more uniform distributions suggest balanced attention across all distance categories. This analysis is particularly valuable for assessing whether the model effectively captures the long-range dependencies that conventional protein language models typically miss due to context limitations.

The attention profiles (short-, medium-, and long-distance) differ substantially among the four models evaluated (Figure 8a). The pre-trained ESM-2-pt model exhibits a highly localized attention mechanism, concentrating almost exclusively on short-range interactions (100%), typically within individual proteins or immediate neighboring residues. This pattern aligns with ESM-2's training paradigm, which focused on single-protein contexts and thus developed representations primarily capturing local residue relationships.

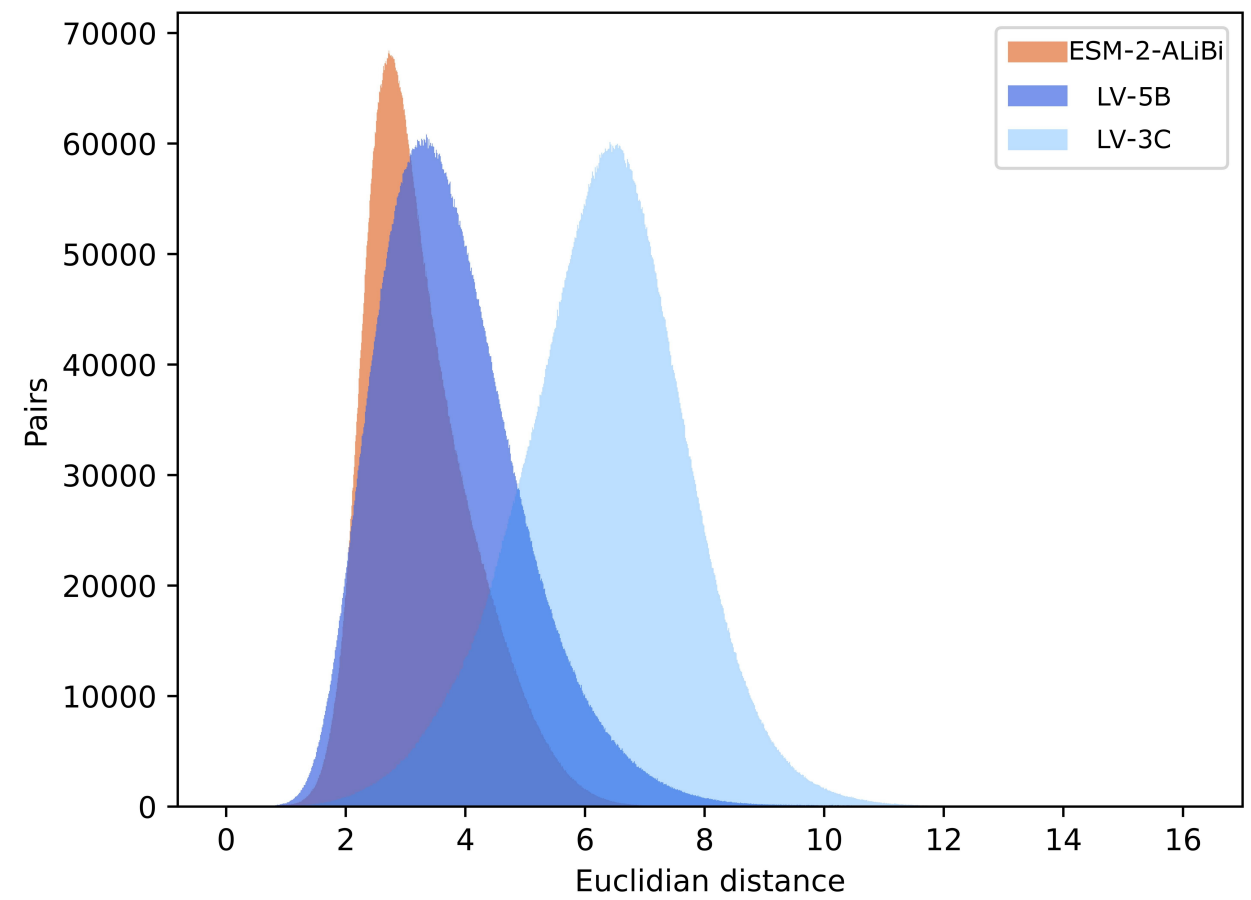

Figure 6: **Protein embeddings pairwise Euclidean distance.** Each curve summarizes ≈12.5 million pair comparisons: lower distances (left tail) indicate within-species similarity, while greater distances reflect increasing taxonomic separation. ESM-2-ALiBi yields the most compact manifold, LV-5B broadens the range, and LV-3C further widens and right-shifts the distribution, demonstrating its larger dynamic range for distinguishing distantly related proteins without sacrificing tight clustering of closely related sequences.

In contrast, the ESM-2-ALiBi fine-tuned model, which employs a sliding-window (LongFormer) attention approach, demonstrates a modest expansion in attention scope. While it identifies a small fraction of medium-distance interactions (3.12%), it still predominantly focuses on short-distance interactions (96.88%). These findings suggest that although the sliding-window methodology enables detection of certain medium-range relationships, the model largely remains constrained to interactions between proximal amino acids.

Our models, LV-3C and LV-5B, exhibit markedly different attention distributions. Both models display balanced average attention scores across all distance categories (short, medium, and long), indicating a comprehensive approach to protein sequence modeling that equally integrates local and global contextual information. However, important distinctions between the two models emerge:

- Model LV-3C most frequently attributes maximal attention to long-distance interactions. This behavior highlights its enhanced capability to capture meaningful relationships between amino acids widely separated in sequence space, potentially spanning different proteins within the viral genome.
- Model LV-5B, while maintaining generally balanced average scores, frequently assigns highest attention importance to both short- and long-distance interactions. This indicates a flexible modeling strategy capable of emphasizing both local structural information and global protein–protein relationships as needed. Notably, LV-5B's attention distribution retains traces of its ESM-2-pt initialization, suggesting it effectively combines characteristics of the pre-trained model with adaptations necessary for the expanded genomic context.

To further investigate inter-protein interactions, we calculated attention scores and identified attention heads specifically enriched for long-range information, following the methodology described in Section 3.11.

In a binary classification framework, using a STRING interaction confidence threshold of 0.500, our LV-3C model achieved an average F1 score of 0.74 and accuracy of 0.88. The classifier demonstrated an Area Under the Curve (AUC) of 0.66 (Figure 9, with a bootstrapped 95% confidence interval of [0.56, 0.75]), indicating statistically significant predictive performance. The difference between F1 (0.74) and AUC (0.66) reflects that F1 is measured at an optimized decision threshold, while AUC captures ranking performance across all thresholds—a more challenging metric given the inherent noise in attention scores and the known incompleteness of viral protein interaction databases. These results show significant concordance between our attention-based interaction scores and experimentally validated interactions in the STRING database, indicating that the method enriches for putative protein interactions within viral genomes. Furthermore, attention-derived scores showed significant positive correlation with STRING confidence scores (Spearman $\rho = 0.33$, $p < 0.001$), providing additional evidence for the biological relevance of the learned attention patterns.

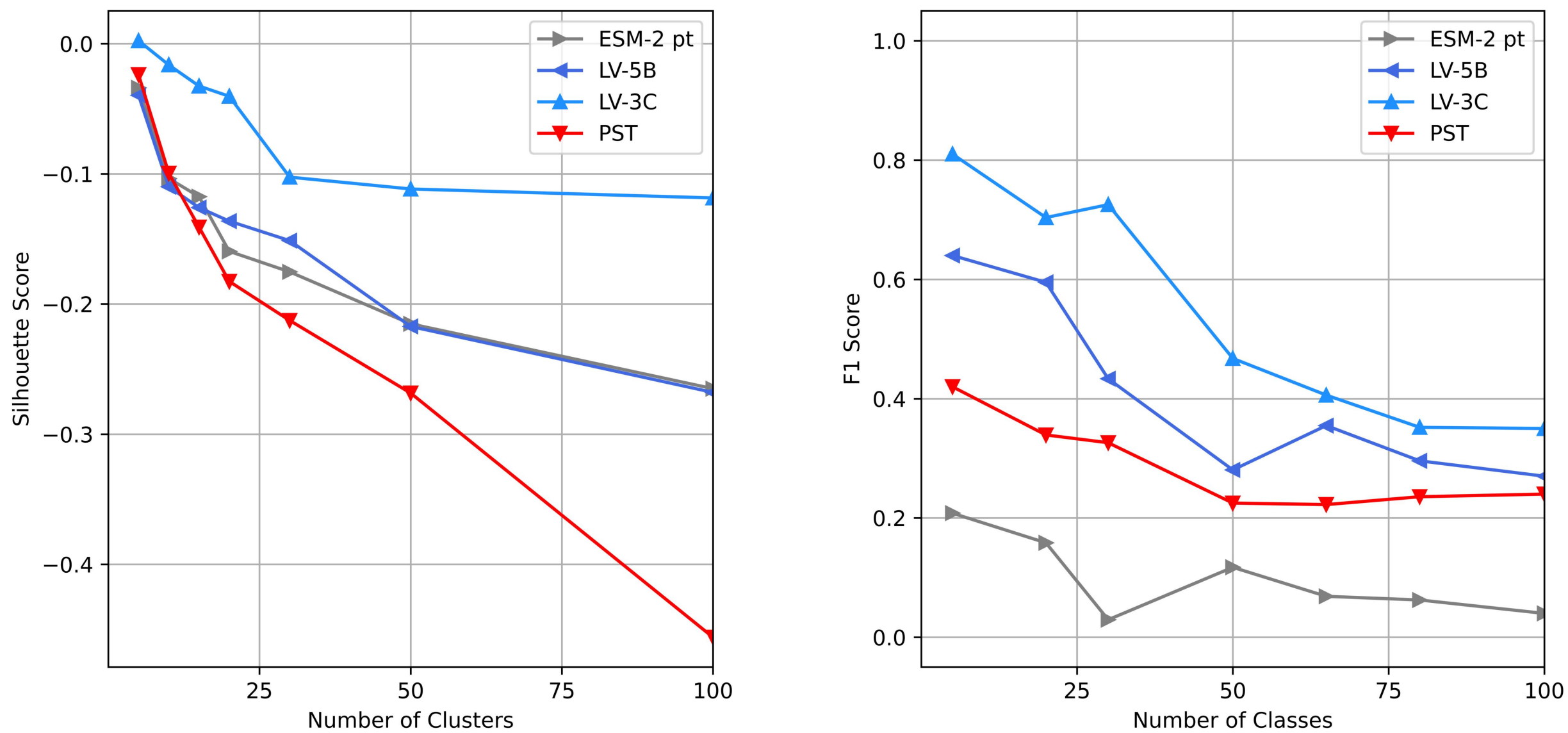

(a) Silhouette Score

(b) Classification

Figure 7: **Evaluation of contextualized protein embeddings. (a)** Silhouette scores comparing protein embeddings generated by LV-3C, LV-5B, Protein Set Transformer (PST), and ESM-2-pt, using taxonomic species as cluster labels. **(b)** F1-scores for a single-layer classifier predicting species identity from protein embeddings (LV-3C, LV-5B, PST, ESM-2-pt). The x-axis varies the number of viral species used as classification targets, from 5 (a coarse 5-way task) to 100 (a fine-grained 100-way task); larger values denote harder, more taxonomically resolved classification.

5 Discussion

We note that attention-derived inter-protein links should be interpreted as computational priors rather than direct evidence of physical PPIs. For this reason, our evaluation strategy focused on language-model metrics, taxonomy/Pfam-aligned downstream tasks, and concordance with independent interaction resources (STRING), rather than claiming direct prediction of physical interactions.

Our findings demonstrate that extending transformer-based language models to encompass entire viral proteomes yields substantial improvements in both local amino acid prediction and broader protein function inference. By designing a biologically informed and content-aware sparse attention mechanism guided by inter-protein co-evolutionary signals, we have addressed the long-standing challenge of modeling densely packed viral genomes while maintaining computational tractability.

A central insight from our study is that higher-order genomic context—the collective presence and arrangement of multiple proteins—significantly enhances protein representation accuracy beyond what can be achieved by analyzing protein sequences in isolation [3], or using context length extension [15, 16] or context-agnostic sparse attention [20, 21, 22]. As the results show, conventional protein language models trained on individual proteins (e.g., ESM-2) focus on local sequence features and exhibit marked performance degradation when context extends beyond their trained window. In contrast, our biologically informed sparse attention approach captures both local relationships and long-range dependencies across entire viral genomes. This more comprehensive perspective reveals functional couplings between non-consecutive proteins that would otherwise remain undetected [29, 30, 31]. To confirm that this advantage arises from the inference step rather than from simply adding long-range links, we trained otherwise identical models given inferred, random, distance-matched, or no long-range links. Only the inferred links lowered held-out perplexity, and the improvement concentrated on the protein pairs the inference model scored most highly (Supplementary Section S6).

The attention patterns learned by our models show statistically significant concordance with experimentally validated protein–protein interactions, benchmarked against the STRING database [33]. The balanced distribution of attention across short, medium, and long-range interactions in our LV-3C and LV-5B models, in contrast to the predominantly local focus of the ESM-2 baselines, further reinforces the biological relevance of our approach.

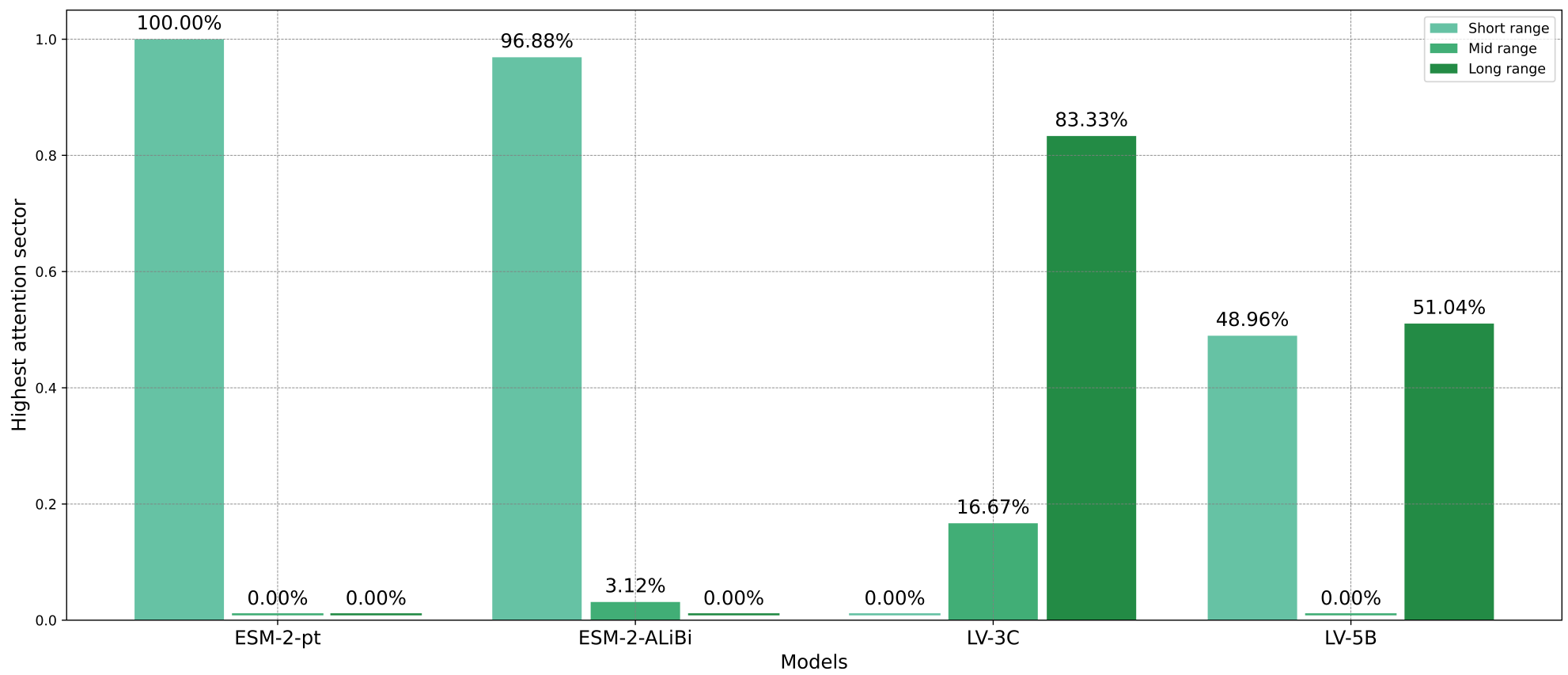

(a)

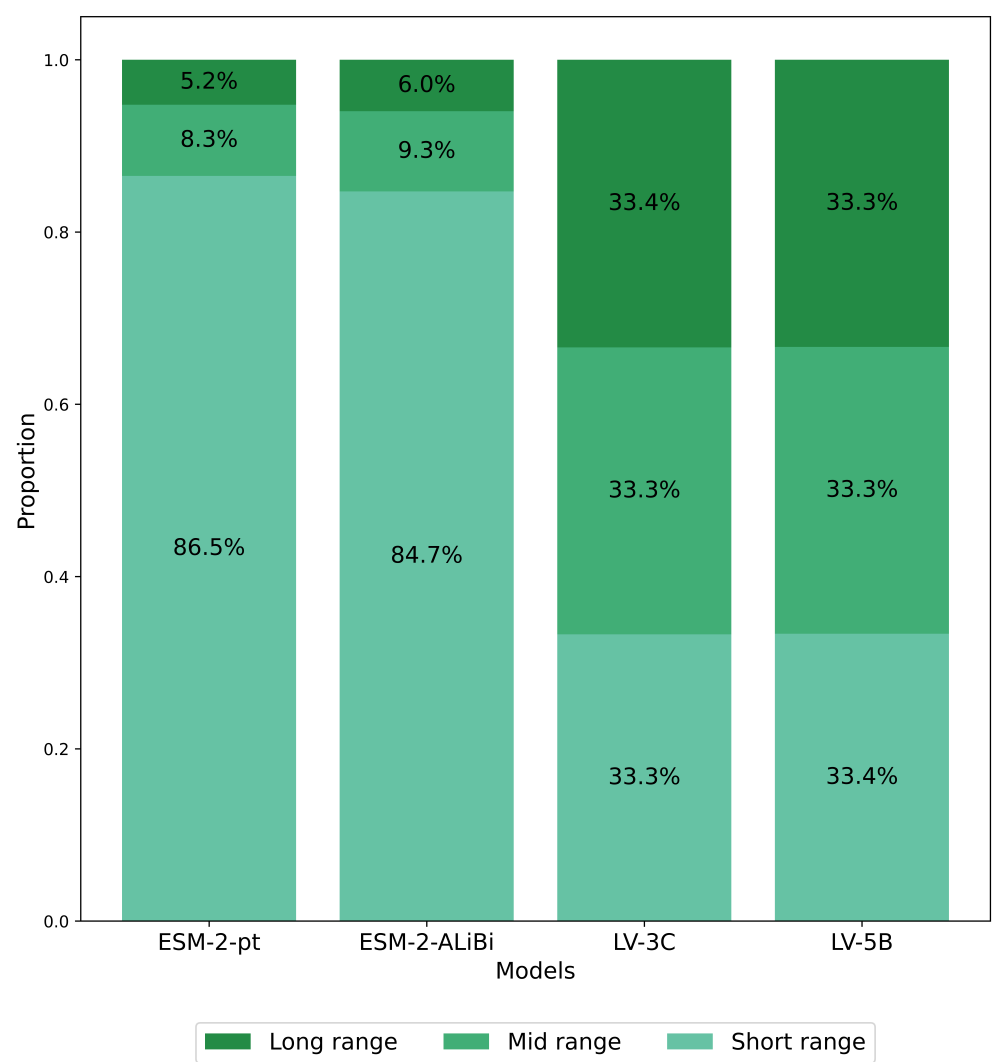

(b)

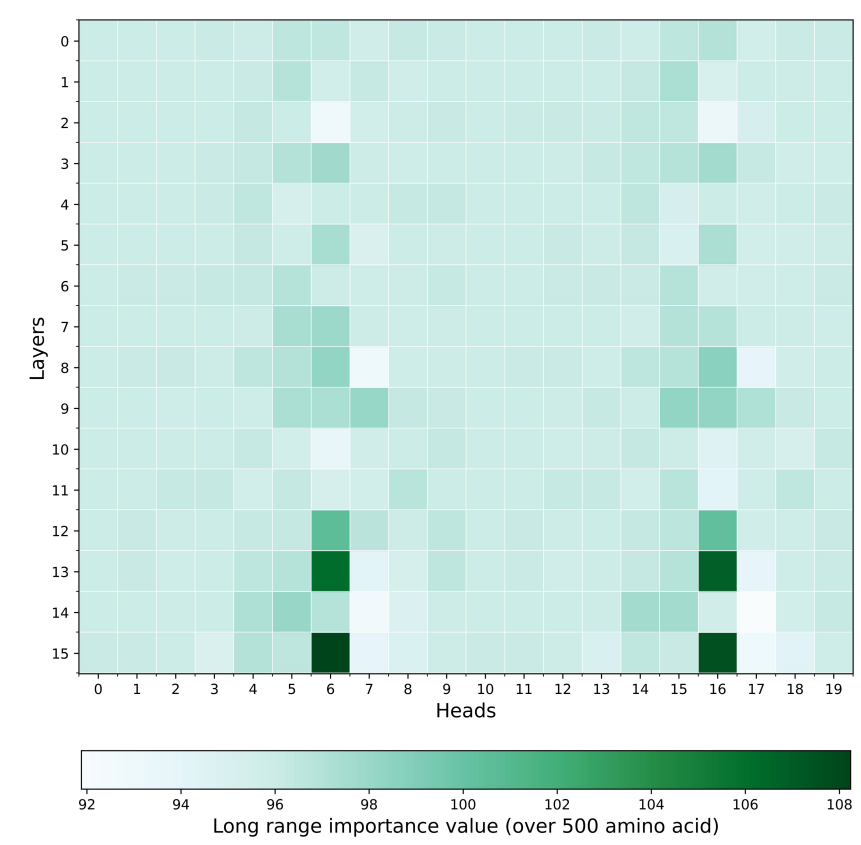

(c)

Figure 8: **Attention analysis of our models.** **(a)** Prevalence of dominant short-range (≤100 aa), medium-range (100–500 aa), and long-range (>500 aa) sectors across viral genomes. The chart represents 1,000 randomly selected viral genomes (≤13,000 aa in length), showing the proportion of genomes in which each interaction range category exhibited the highest mean attention score. **(b)** Distribution of normalized attention scores across interaction distance categories in viral proteomes. Stacked bar charts represent the distribution of attention scores for each interaction range category across 1,000 viral genomes. **(c)** Fine-grained analysis of long-range attention patterns in the LV-3C model. Darker (green) blocks indicate attention heads with a predominant long range attention. The visualization demonstrates how attention mechanisms capture biologically significant inter-protein interactions spanning substantial genomic distances within viral proteomes. Distribution variance indicates the relative focus of attention heads on specific interaction distances.

The significant divergence in embedding spaces between context-independent (other models) and the context-aware LV-3C and LV-5B models, as evidenced by the distinct distributions of pairwise Euclidean distances between protein embeddings, underscores the fundamental difference in information captured by these approaches. The wider embedding space of our LV-3C model, with its greater interquartile range and broader distribution of distances, suggests a richer representation that incorporates both sequence-specific and context-dependent information.

To assess generalization across viral diversity, we evaluated model performance specifically on RNA viruses, which represent the most abundant and challenging subgroup in our dataset (Supplementary Material S5). Despite their higher mutation rates and structural complexity, our models consistently outperformed baselines (Supplementary Figure S6), suggesting robust performance across viral groups.

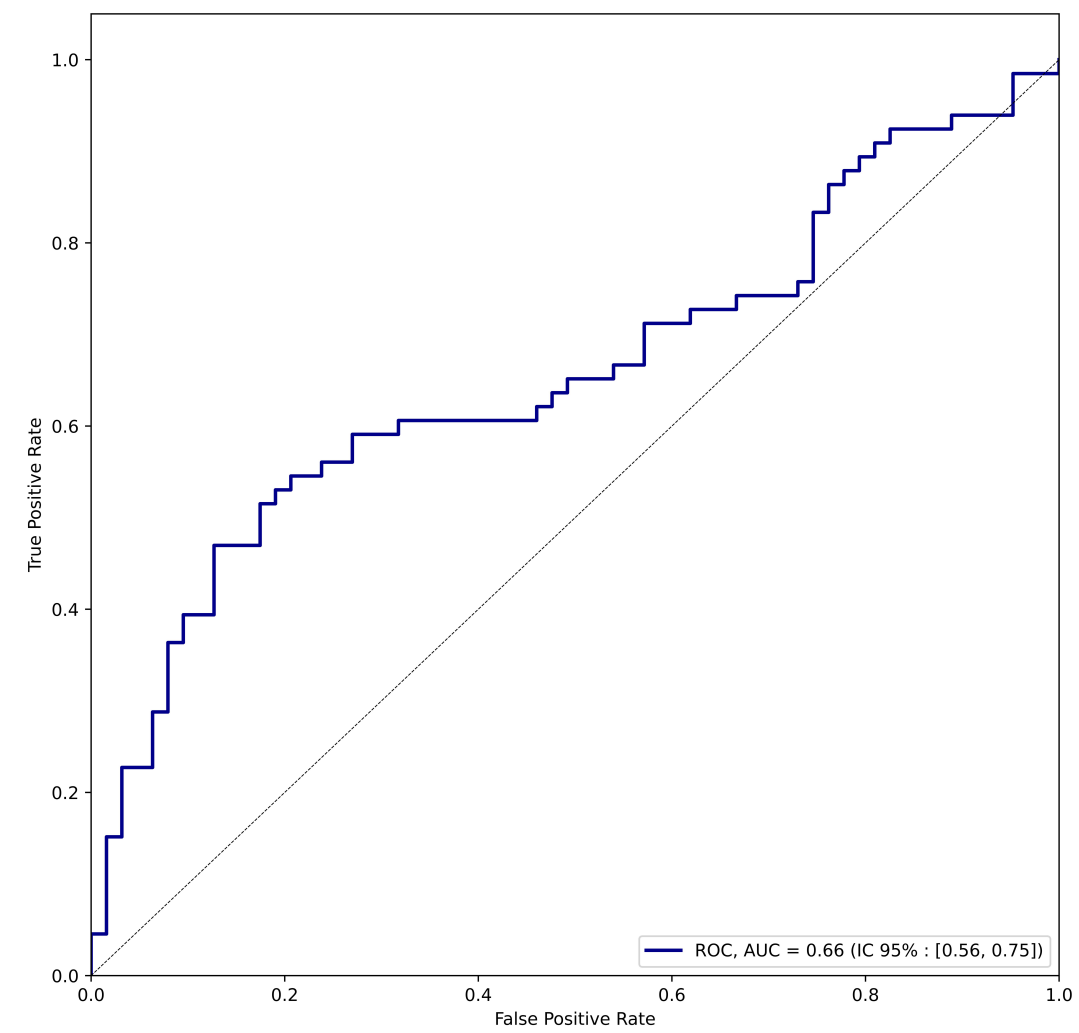

Figure 9: **Receiver Operating Characteristic (ROC) curve of the binary classification task.** ROC curve for distinguishing STRING-supported protein pairs from non-reported protein pairs using attention-derived pair scores. The curve shows LV-3C ability to distinguish experimentally validated interactions (STRING confidence $\geq$ 0.500) from non-interacting protein pairs across five viral genomes (146 positive interactions). The AUC of 0.66, with a 95% bootstrapped confidence interval of [0.56, 0.75], indicates statistically significant predictive performance.

To assess sensitivity to our 90% deduplication threshold, we additionally clustered at 65% identity, yielding 246,737 sequences—indicating that 71% of sequences retained at 90% clustering have no other sequence sharing >65% identity. This analysis suggests that the majority of our evaluation data consists of substantially divergent sequences, not near-duplicates. The impact of sequence similarity also varies by task: perplexity is most sensitive to train/test similarity, while taxonomy and species classification are less affected since these tasks require learning species-level patterns, making within-species similarity a desired signal rather than a confounder. Critically, all models were evaluated under identical conditions, ensuring valid relative comparisons.

To validate that our embeddings capture functional information beyond taxonomy, we evaluated performance using Pfam protein domain annotations (Supplementary Figure S5). While silhouette scores are low, reflecting that genomic context, not domain identity, is the dominant organizing principle—classification performance remains strong, with LV-3C and LV-5B outperforming baselines. This confirms that domain information is encoded within our embeddings alongside the contextual signals that distinguish proteins with identical domains in different genomic environments.

Despite these advances, computational overhead remains a practical consideration. Our selection of a maximum of 50 protein–protein interactions for the sparse attention mechanism strikes an effective balance for viral datasets while maintaining computational efficiency. This parameter can be readily increased if GPU resources are available. We intentionally constrained our implementation to run on a single GPU, as we believe this represents a widely accessible computational environment for most research groups. While we utilized an NVIDIA A6000 for this study, the rapid advancement of more affordable GPUs (sub-$5,000) with comparable memory capacities makes our approach increasingly accessible. For processing significantly larger genomes—particularly from eukaryotic organisms—additional optimizations may be beneficial. Additionally, our semi-supervised approach to inferring putative interactions could benefit from larger ground-truth databases or complementary evidence from proteomic or structural studies to further refine the biological relevance of the learned attention patterns.

Although our training data includes human pathogen sequences, our models pose substantially lower biosecurity risk compared to generative genomic models such as Evo [35]. Both LV-3C and LV-5B are designed to produce embeddings for downstream analysis rather than to generate novel sequences. The masked language modeling objective predicts individual positions given surrounding context, rather than generating complete sequences autoregressively. These distinctions substantially limit the utility of our models for generating functional novel proteins or genomes.

Looking forward, our framework offers considerable promise for extension to larger viral genomes, bacteriophages, and potentially even eukaryotic genomes with appropriate scaling. The methodology could be particularly valuable for analyzing metagenomic data, where contextual information might help resolve ambiguities in protein function

assignment. Furthermore, the correlation between embedding similarity and taxonomic distance suggests potential applications in phylogenetic analysis and evolutionary studies.

6 Conclusion

Our study proposes a novel framework for viral protein language modeling by extending transformer architectures to process entire viral genomes. By integrating a biologically informed sparse attention mechanism, our approach efficiently captures putative long-range inter-protein interactions that are missed by conventional single-protein models. This design not only reduces perplexity across extended sequences (up to 61,000 aa) but also generates context-aware embeddings that accurately recapitulate known co-evolutionary relationships—for instance, among PolA, RNR, and helicase.

Our results demonstrate that large-scale learning improves embedding quality. The enhanced clustering of protein and genome embeddings, along with robust taxonomic classification, underscores the broader applicability of our method to phylogenetic analysis and metagenomic studies. Our work provides a solid foundation for working with viral genomes and paves the way for more refined analyses of protein–protein interactions, ultimately deepening our understanding of viral biology and evolution. Looking ahead, our framework offers considerable promise for extension to larger genomes, and potentially even eukaryotic genomes. By refining the biological specificity of sparse attention patterns and incorporating additional layers of experimental validation, our approach can continue to evolve toward more precise and comprehensive genomic analyses. Ultimately, this work not only deepens our understanding of viral genome organization and evolution but also provides a scalable framework for protein modeling at genomic scales, bridging the gap between computational biology and experimental validation.

7 Acknowledgments

This work is supported by the National Science Foundation grants 1736030 and 2025567. Support from the University of Delaware Center for Bioinformatics and Computational Biology (CBCB) Core Facility, the University of Delaware Sequencing and Genotyping Center, and use of the BIOMIX compute cluster was made possible through funding from Delaware INBRE (NIGMS P20GM103446, NIH S10OD028725), and the State of Delaware.

References

[1] Xueliang Liu. Deep Recurrent Neural Network for Protein Function Prediction from Sequence. January 2017. arXiv:1701.08318 [q-bio].

[2] Abel Chandra, Laura Tünnermann, Tommy Löfstedt, and Regina Gratz. Transformer-based deep learning for predicting protein properties in the life sciences. *eLife*, 12:e82819, 2023.

[3] Zeming Lin, Halil Akin, Roshan Rao, Brian Hie, Zhongkai Zhu, Wenting Lu, Allan dos Santos Costa, Maryam Fazel-Zarandi, Tom Sercu, Sal Candido, and Alexander Rives. Language models of protein sequences at the scale of evolution enable accurate structure prediction. July 2022. Pages: 2022.07.20.500902 Section: New Results.

[4] Ahmed Elnaggar, Michael Heinzinger, Christian Dallago, Ghalia Rihawi, Yu Wang, Llion Jones, Tom Gibbs, Tamas Feher, Christoph Angerer, Martin Steinegger, Debsindhu Bhowmik, and Burkhard Rost. ProtTrans: Towards Cracking the Language of Life's Code Through Self-Supervised Deep Learning and High Performance Computing, May 2021. arXiv:2007.06225 [cs].

[5] Erik Nijkamp, Jeffrey Ruffolo, Eli N. Weinstein, Nikhil Naik, and Ali Madani. ProGen2: Exploring the Boundaries of Protein Language Models, June 2022. arXiv:2206.13517 [cs].

[6] Claire D. McWhite and Mona Singh. Vector-clustering Multiple Sequence Alignment: Aligning into the twilight zone of protein sequence similarity with protein language models, October 2022.

[7] Benjamin Giovanni Iovino and Yuzhen Ye. Protein embedding based alignment. *BMC Bioinformatics*, 25(1):85, February 2024.

[8] Zeming Lin, Halil Akin, Roshan Rao, Brian Hie, Zhongkai Zhu, Wenting Lu, Nikita Smetanin, Robert Verkuil, Ori Kabeli, Yaniv Shmueli, Allan dos Santos Costa, Maryam Fazel-Zarandi, Tom Sercu, Salvatore Candido, and Alexander Rives. Evolutionary-scale prediction of atomic level protein structure with a language model, October 2022. Pages: 2022.07.20.500902 Section: New Results.

[9] Christoph Feinauer, Hendrik Szurmant, Martin Weigt, and Andrea Pagnani. Inter-protein sequence co-evolution predicts known physical interactions in bacterial ribosomes and the trp operon. *PLoS One*, 11(2):e0149166, February 2016.

[10] Elias T Friman, Ilya M Flyamer, Davide Marenduzzo, Shelagh Boyle, and Wendy A Bickmore. Ultra-long-range interactions between active regulatory elements. *Genome Res.*, 33(8):1269–1283, August 2023.

[11] Yiling Jiang, Kuijie Tong, Roubin Yao, Yuanze Zhou, Hanwen Lin, Liubing Du, Yunyun Jin, Liu Cao, Jingquan Tan, Xing-Ding Zhang, Deyin Guo, Ji-An Pan, and Xiaoxue Peng. Genome-wide analysis of protein–protein interactions and involvement of viral proteins in SARS-CoV-2 replication. *Cell & Bioscience*, 11(1):140, July 2021.

[12] Xindi Wang, Mahsa Salmani, Parsa Omidi, Xiangyu Ren, Mehdi Rezagholizadeh, and Armaghan Eshaghi. Beyond the Limits: A Survey of Techniques to Extend the Context Length in Large Language Models, May 2024. arXiv:2402.02244 [cs].

[13] Ashish Vaswani, Noam Shazeer, Niki Parmar, Jakob Uszkoreit, Llion Jones, Aidan N. Gomez, Lukasz Kaiser, and Illia Polosukhin. Attention Is All You Need, August 2023. arXiv:1706.03762 [cs].

[14] J Rodney Brister, Danso Ako-Adjei, Yiming Bao, and Olga Blinkova. Ncbi viral genomes resource. *Nucleic acids research*, 43(D1):D571–D577, 2015.

[15] Jianlin Su, Yu Lu, Shengfeng Pan, Ahmed Murtadha, Bo Wen, and Yunfeng Liu. RoFormer: Enhanced Transformer with Rotary Position Embedding, November 2023. arXiv:2104.09864 [cs].

[16] Ofir Press, Noah A. Smith, and Mike Lewis. Train Short, Test Long: Attention with Linear Biases Enables Input Length Extrapolation, April 2022. arXiv:2108.12409 [cs].

[17] Shouyuan Chen, Sherman Wong, Liangjian Chen, and Yuandong Tian. Extending Context Window of Large Language Models via Positional Interpolation, June 2023. arXiv:2306.15595 [cs].

[18] Tri Dao, Daniel Y. Fu, Stefano Ermon, Atri Rudra, and Christopher Ré. FlashAttention: Fast and Memory-Efficient Exact Attention with IO-Awareness, June 2022. arXiv:2205.14135 [cs].

[19] Philippe Tillet, H. T. Kung, and David Cox. Triton: an intermediate language and compiler for tiled neural network computations. In *Proceedings of the 3rd ACM SIGPLAN International Workshop on Machine Learning and Programming Languages*, pages 10–19, Phoenix AZ USA, June 2019. ACM.

[20] Iz Beltagy, Matthew E. Peters, and Arman Cohan. Longformer: The Long-Document Transformer, December 2020. arXiv:2004.05150 [cs].

[21] Yukang Chen, Shengju Qian, Haotian Tang, Xin Lai, Zhijian Liu, Song Han, and Jiaya Jia. LongLoRA: Efficient Fine-tuning of Long-Context Large Language Models, March 2024. arXiv:2309.12307 [cs].

[22] Manzil Zaheer, Guru Guruganesh, Avinava Dubey, Joshua Ainslie, Chris Alberti, Santiago Ontanon, Philip Pham, Anirudh Ravula, Qifan Wang, Li Yang, and Amr Ahmed. Big Bird: Transformers for Longer Sequences, January 2021. arXiv:2007.14062 [cs].

[23] Aurko Roy, Mohammad Saffar, Ashish Vaswani, and David Grangier. Efficient Content-Based Sparse Attention with Routing Transformers, October 2020. arXiv:2003.05997 [cs].

[24] Nadav Brandes, Dan Ofer, Yam Peleg, Nadav Rappoport, and Michal Linial. ProteinBERT: a universal deep-learning model of protein sequence and function. *Bioinformatics*, 38(8):2102–2110, April 2022.

[25] Baris E Suzek, Yuqi Wang, Hongzhan Huang, Peter B McGarvey, Cathy H Wu, and UniProt Consortium. UniRef clusters: a comprehensive and scalable alternative for improving sequence similarity searches. *Bioinformatics*, 31(6):926–932, March 2015.

[26] Cody Martin, Anthony Gitter, and Karthik Anantharaman. Protein Set Transformer: A protein-based genome language model to power high diversity viromics, July 2024. Pages: 2024.07.26.605391 Section: New Results.

[27] Yunha Hwang, Andre Cornman, Elizabeth Kellogg, Sergey Ovchinnikov, and Peter Girguis. Genomic language model predicts protein co-regulation and function. 2024.

[28] Alexander Rives, Joshua Meier, Tom Sercu, Siddharth Goyal, Zeming Lin, Jason Liu, Demi Guo, Myle Ott, C. Lawrence Zitnick, Jerry Ma, and Rob Fergus. Biological structure and function emerge from scaling unsupervised learning to 250 million protein sequences. *Proc Natl Acad Sci U S A*, 118(15):e2016239118, April 2021.

[29] Rachel A. Keown, Jacob T. Dums, Phillip J. Brumm, Joyanne MacDonald, David A. Mead, Barbra D. Ferrell, Ryan M. Moore, Amelia O. Harrison, Shawn W. Polson, and K. Eric Wommack. Novel viral dna polymerases from metagenomes suggest genomic sources of strand-displacing biochemical phenotypes. *Frontiers in Microbiology*, Volume 13 - 2022, 2022.

[30] Eugene V. Koonin, Valerian V. Dolja, and Mart Krupovic. The logic of virus evolution. *Cell Host & Microbe*, 30(7):917–929, 2022.

[31] Daniel J Nasko, Jessica Chopyk, Eric G Sakowski, Barbra D Ferrell, Shawn W Polson, and K Eric Wommack. Family a DNA polymerase phylogeny uncovers diversity and replication gene organization in the virioplankton. *Front. Microbiol.*, 9:3053, December 2018.

[32] torch.sparse_bsr_tensor — PyTorch 2.6 documentation.

[33] Damian Szklarczyk, Rebecca Kirsch, Mikaela Koutrouli, Katerina Nastou, Farrokh Mehryary, Radja Hachilif, Annika L. Gable, Tao Fang, Nadezhda T. Doncheva, Sampo Pyysalo, Peer Bork, Lars J. Jensen, and Christian von Mering. The STRING database in 2023: protein-protein association networks and functional enrichment analyses for any sequenced genome of interest. *Nucleic Acids Res*, 51(D1):D638–D646, January 2023.

[34] Milot Mirdita, Martin Steinegger, and Johannes Söding. MMseqs2 desktop and local web server app for fast, interactive sequence searches. *Bioinformatics*, 35(16):2856–2858, August 2019.

[35] Eric Nguyen, Michael Poli, Matthew G. Durrant, Armin W. Thomas, Brian Kang, Jeremy Sullivan, Madelena Y. Ng, Ashley Lewis, Aman Patel, Aaron Lou, Stefano Ermon, Stephen A. Baccus, Tina Hernandez-Boussard, Christopher Ré, Patrick D. Hsu, and Brian L. Hie. Sequence modeling and design from molecular to genome scale with Evo, February 2024. Pages: 2024.02.27.582234 Section: New Results.

[36] William L Harrigan, Barbra D Ferrell, K Eric Wommack, et al. Improvements in viral gene annotation using large language models and soft alignments. *BMC Bioinformatics*, 25:165, 2024.

[37] Rajan Sawhney et al. Fine-tuning protein language models unlocks the potential of underrepresented viral proteomes. *PeerJ*, 13:e19919, 2025.

Supplementary Material

S1 NCBI Virus Database

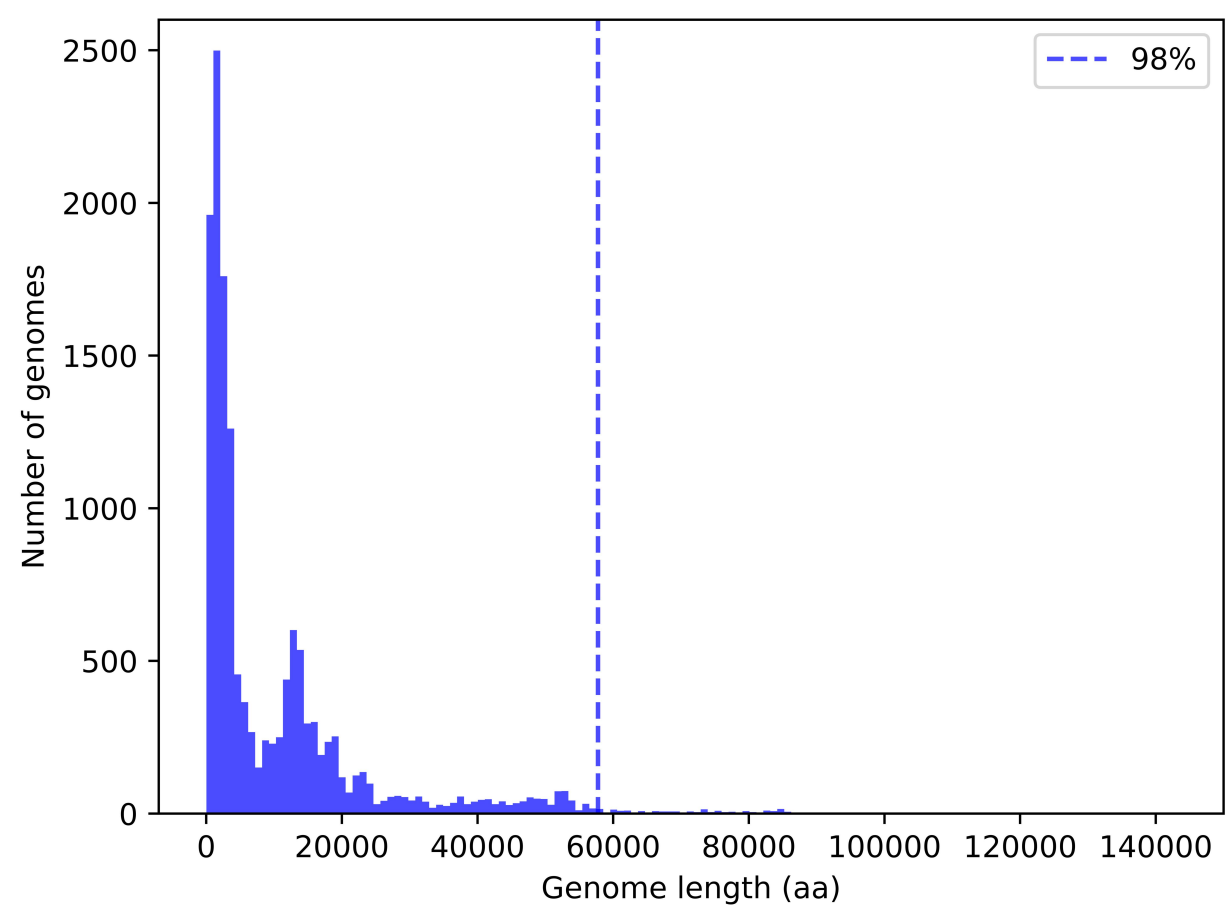

Supplementary Figure S1: **Viral genome length distributions in the NCBI Virus Database.** This histogram shows that a context length of 61k amino acids allows us to process over 98% of the 14,436 complete genomes of the database.

S2 Development of the PPI Inference Model (Stage 1)

To extend ESM-2's context window for PPI inference, we evaluated two positional encoding strategies (RoPE and ALiBi) and three sparse attention mechanisms (Longformer, Shifted Sparse Attention, and BigBird).

The evaluation of positional encoding strategies showed that ALiBi exhibited superior performance in handling extended sequences. Although RoPE demonstrated robust performance within its original training context, its effectiveness deteriorated significantly beyond 4,000 aa. In contrast, ALiBi maintained consistent perplexity (1.99) across sequence lengths extending up to 13,000 aa (Supplementary Figure S2).

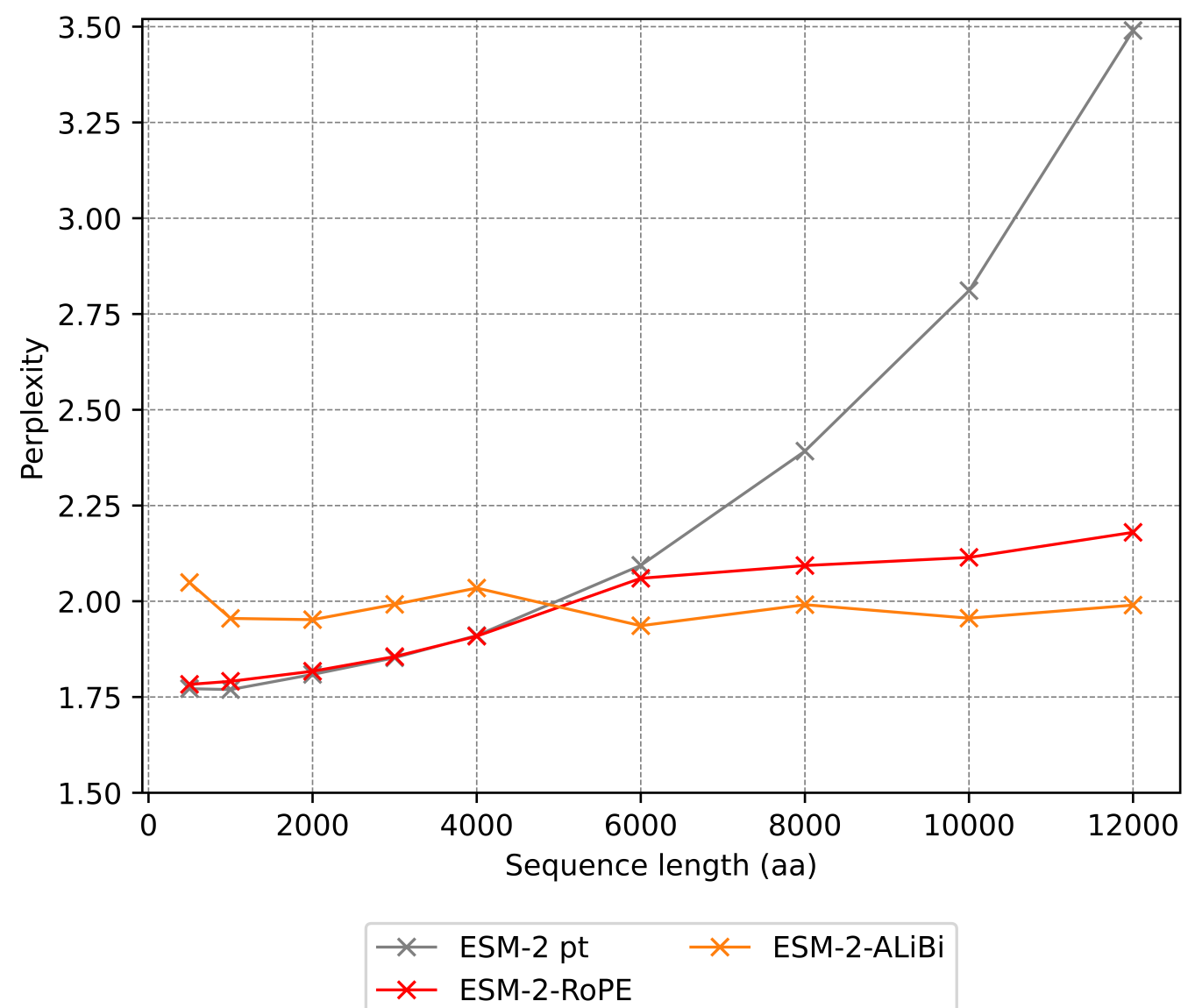

Supplementary Figure S2: **Perplexity evaluation of positional encoding methods for fine-tuning.** Performance metrics for ESM-2-pt and fine-tuned variants using Sliding Window (SW) attention mechanism with RoPE and ALiBi positional encoding. Perplexity values represent mean across sequences ranging from 500 to 13,000 amino acids.

Among the sparse attention mechanisms tested, Longformer provided the optimal balance between performance (perplexity: 1.99, silhouette score: 0.88) and computational efficiency (training time: 32s/step or 46 hours) (Supplementary Table S1). Based on these findings, we adopted ALiBi positional encoding combined with Longformer attention for ESM-2-PPI.

	ESM-2-pt	Longformer	Longformer + full	S^2-Attn	S^2-Attn + full	BigBird
Training context size	1,024	4,000	4,000	4,000	4,000	4,000
Training time (s/step)		32	32	36	36	126
Perplexity	3.17	2.18	**1.99**	2.22	2.05	2.02
Silhouette	0.65	0.85	**0.88**	0.86	0.85	0.84

Supplementary Table S1: **Performance metrics of ESM-2-pt and fine-tuned versions using different sparse attention patterns.** Longformer refers to the Sliding Window Attention pattern from [20], S^2-Attn to the Shifted Sparse Attention from [21] and BigBird to the eponymous attention pattern from [22]. '+full' means normalization and embedding layers are trainable during low-rank fine-tuning. Metrics are calculated on genome fragments ranging between 10,000 and 13,000 amino acids.

To validate that ESM-2-PPI captures biologically meaningful interactions, we analyzed genomic fragments from 83 viral genomes containing complete PolA, RNR, and HEL proteins. These genomes were identified by searching the

NCBI Virus database using BLAST and soft alignment [36, 37] against reference sequences for each protein, retaining only genomes where all three were detected with high confidence (e-value $< 10^{-3}$, coverage $> 80\%$). Supplementary Figure S3 shows that these known interacting pairs consistently rank among the highest-scoring interactions, particularly after excluding genomically adjacent pairs. A Wilcoxon–Mann–Whitney test confirmed a highly significant difference between the distributions ($p = 3.31 \times 10^{-22}$).

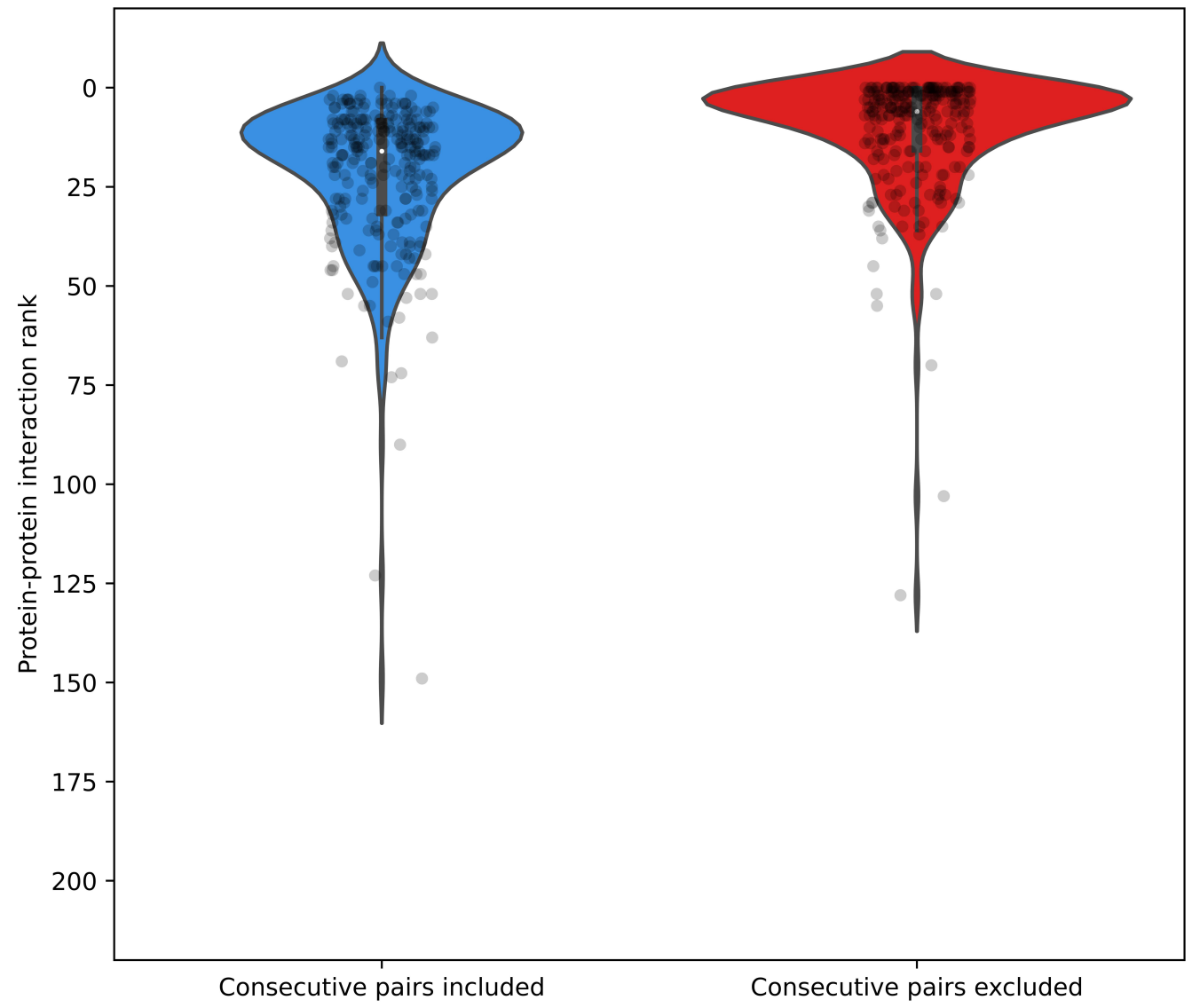

Supplementary Figure S3: **Distribution of interaction pair rankings of DNA polymerase I (PolA), ribonucleotide reductase (RNR), and helicase (HEL) across 83 viral genomes.** Violin plots represent the ranking positions of PolA-HEL, PolA-RNR, and RNR-HEL pairs (total n = 249). Left panel: rankings among all protein pairs within fragments. Right panel: rankings after excluding genomically adjacent protein pairs.

To select an appropriate interaction threshold, we evaluated different values of k (the number of top-ranked non-adjacent pairs to include). Supplementary Figure S4 and Supplementary Table S2 show that $k = 50$ retains 97.6% of known interacting pairs while remaining computationally tractable.

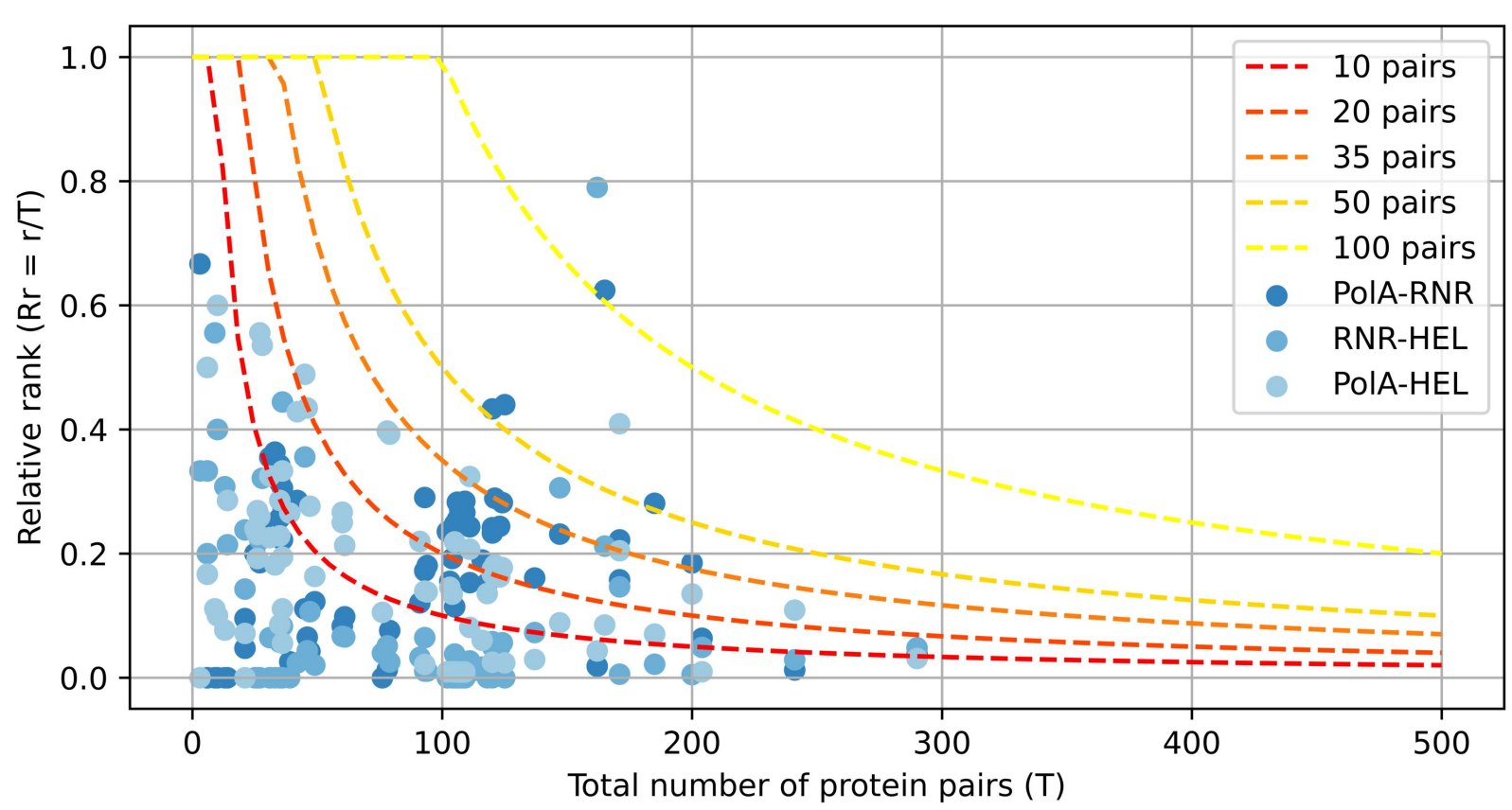

Supplementary Figure S4: **Selection of interaction threshold.** PolA-HEL, PolA-RNR, and RNR-HEL pairs shown as a function of total genomic pairs and relative genomic rank. Dashed curves denote selection thresholds; pairs below each curve are retained for attention calculations.

Threshold (number of selected pairs)	10	20	35	50	100
% studied pairs selected	64.0%	80.6%	94.4%	97.6%	99.2%

Supplementary Table S2: **Percentage of known interacting pairs** (PolA-RNR-HEL) captured at different selection thresholds.

S3 Using embeddings to predict taxonomy

To assess the taxonomic discriminability of the learned embeddings, we conducted a supervised classification analysis. For each protein or genome in the dataset, a 1,280-dimensional embedding vector was generated and paired with its corresponding taxonomic label. The dataset, comprising 100,000 proteins and 5,000 genomes, was randomly partitioned into training (80%) and validation (20%) subsets. To ensure a robust evaluation, we varied the number of taxonomic species from 5 to 100, as described in Section 3.9. A single-layer neural network with an intermediate hidden dimension of 2,560 was trained for 1,000 epochs using a learning rate of 1e-3. Model performance was evaluated using 5-fold cross-validation, and standard classification metrics were reported.

S4 Validation Using Protein Domain Annotations

To assess whether our embeddings capture functional information beyond taxonomic signals, we evaluated clustering and classification performance using Pfam protein family annotations. Pfam families group proteins by conserved domain structure and function, providing an orthogonal validation axis to taxonomy-based evaluations.

Supplementary Figure S5 presents silhouette scores and classification F1-scores using Pfam families as labels. Notably, silhouette scores are negative across all models (Supplementary Figure S5a), indicating that proteins from the same Pfam family do not form tight clusters in embedding space. This result is expected and reflects a key design principle of our approach: our embeddings are trained to encode genomic context, not solely domain identity. A helicase in a bacteriophage and a helicase in an RNA virus belong to the same Pfam family but occupy fundamentally different genomic contexts, and our models are designed to distinguish them. Embeddings that clustered purely by Pfam family would simply recapitulate single-protein models like ESM-2, rather than capturing the genomic relationships that are the focus of this work.

Despite this, classification performance remains strong (Supplementary Figure S5b), with LV-3C and LV-5B consistently outperforming ESM-2-pt and PST across all numbers of Pfam families evaluated. This indicates that Pfam-relevant information is encoded within the embeddings—it is simply not the dominant organizing signal. A linear classifier can extract domain identity even when genomic context is the primary structuring principle of the embedding space.

Taken together, these results demonstrate that our context-aware embeddings capture functional domain information at least as effectively as single-protein baselines, while additionally encoding genomic context that distinguishes proteins with identical domains but different genomic environments.

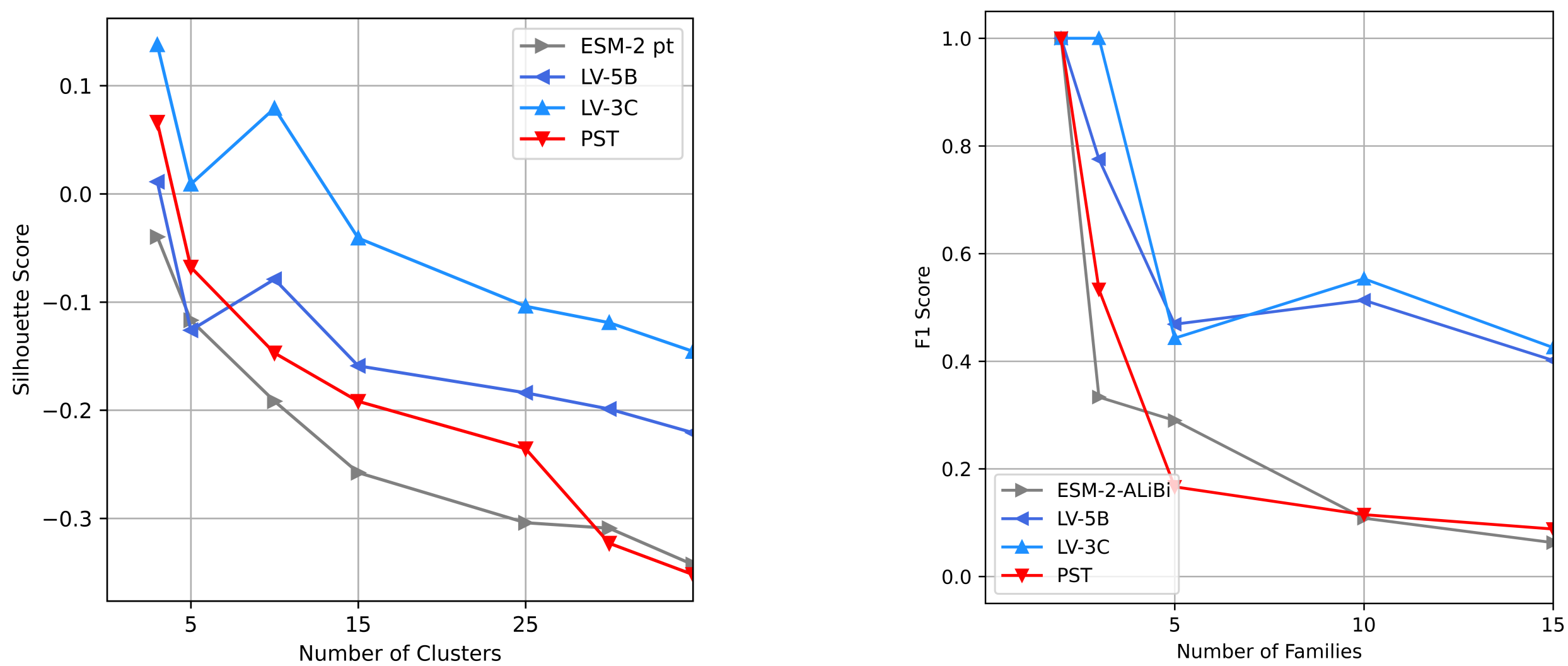

(a) Silhouette Score

(b) Classification

Supplementary Figure S5: **Evaluation of embeddings using Pfam domain annotations. (a)** Silhouette scores using Pfam families as cluster labels. Negative scores across all models reflect that genomic context—not domain identity alone—is the primary organizing principle of the embedding space. **(b)** F1-scores for Pfam family classification. Despite low clustering coherence, LV-3C and LV-5B achieve strong classification performance, indicating that domain information is encoded but not dominant.

S5 Model Performance Across Viral Subgroups

Viral genomes vary substantially in size, structure, and evolutionary dynamics. To assess whether our models generalize across this diversity, we evaluated performance on RNA viruses—the most challenging and abundant subgroup in our dataset (approximately 70% of training genomes).

RNA viruses present unique modeling challenges: their high mutation rates and rapid evolution generate greater sequence diversity, while their broad host ranges and varied genome organizations (segmented, ambisense, positive/negative-sense) introduce additional complexity. If our models perform well on this demanding subgroup, we would expect comparable or better performance on structurally simpler dsDNA and ssDNA viruses.

Supplementary Figure S6 compares model performance on RNA viruses (solid lines) versus all viruses (lighter lines). As expected, performance on RNA viruses is slightly lower than on the full dataset; the latter being lifted by the inclusion of less complex viral groups. Critically, however, LV-3C and LV-5B consistently outperform both ESM-2-pt and PST baselines on RNA viruses across all evaluation metrics. These results demonstrate that our models capture biologically meaningful signals even within the most challenging viral subgroup, suggesting robust generalization across viral diversity.

S6 Controls for the Interaction-Inference Step

To test whether the interaction-inference step itself improves the model, we ran a controlled comparison in which the only parameter that varied was which long-range links each model received. The random conditions use three independent draws whose results we average. Additionally, because the inferred links tend to fall at particular genomic distances, we also included a distance-matched control that draws random links at distances similar to those of the inferred pairs, so that any difference cannot be attributed to genomic distance alone. Together with a model that receives no long-range links and connects only neighboring proteins, this gives eight models.

- **Inferred** (1 model). The 50 top-ranked pairs from the interaction-inference model (ESM-2-PPI).
- **Random** (3 models). 50 pairs drawn uniformly at random, averaged over three independent draws.

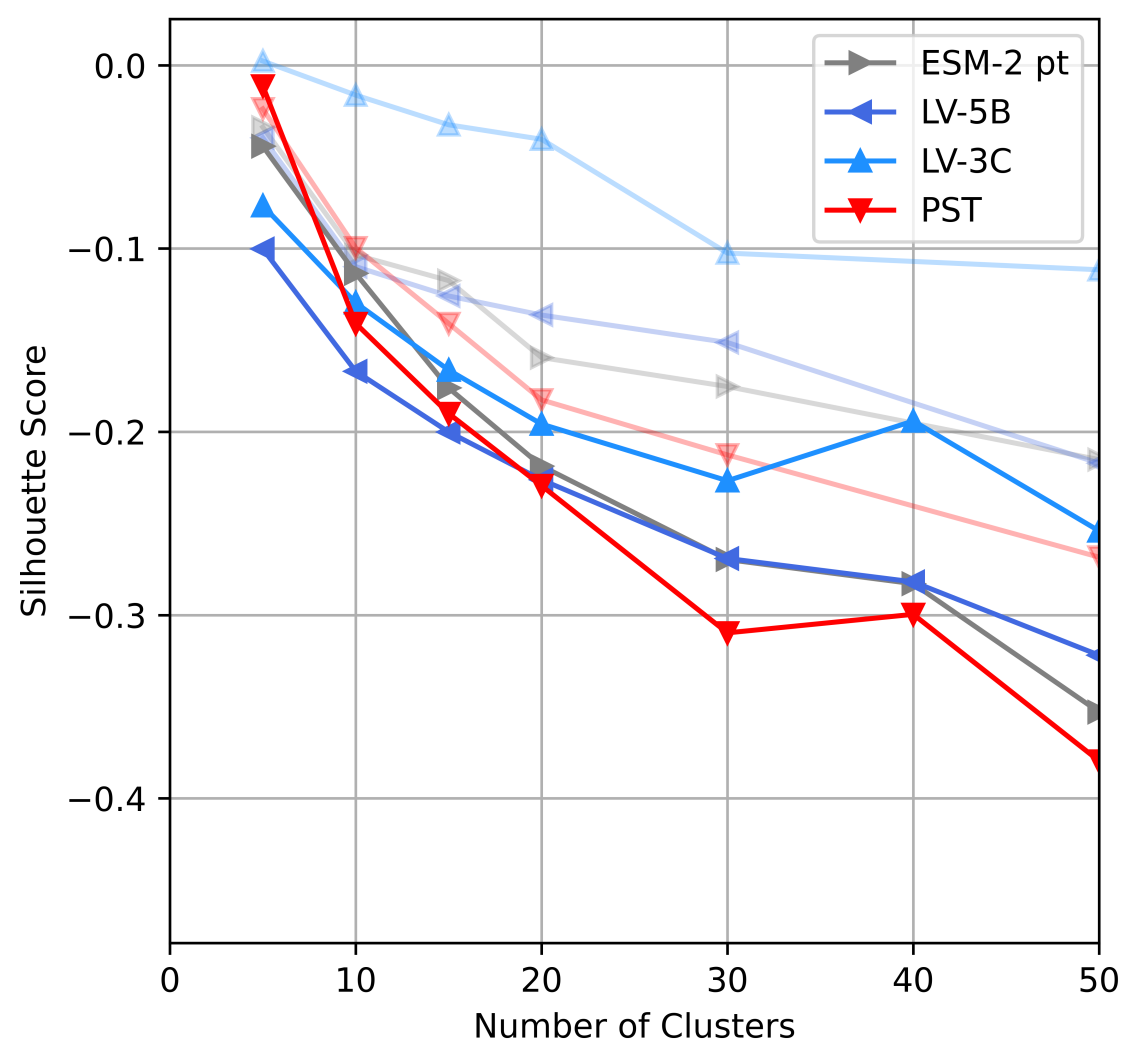

(a) Silhouette Score

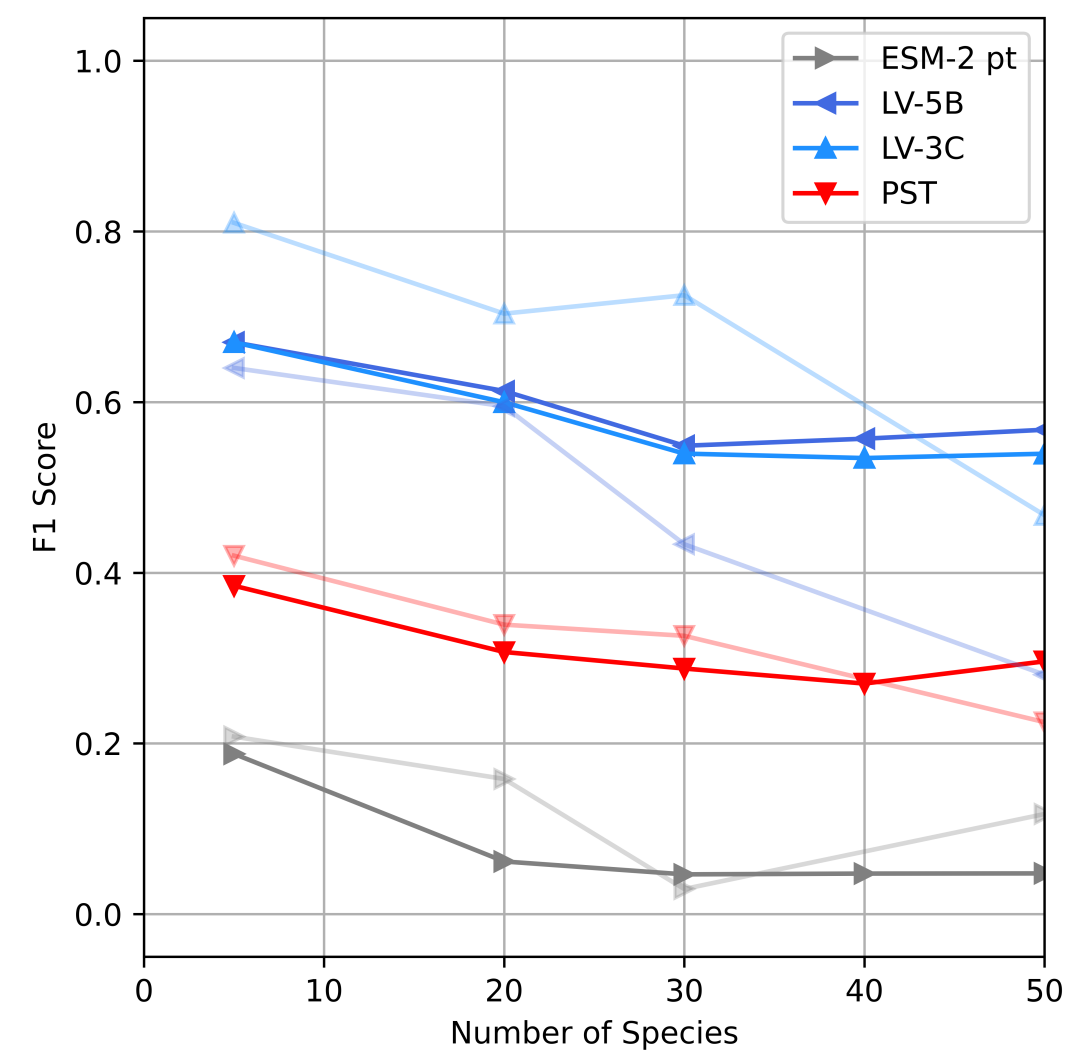

(b) Classification

Supplementary Figure S6: **Model performance on RNA viruses compared to all viruses. (a)** Silhouette scores and **(b)** F1-scores for taxonomic species classification, comparing LV-3C, LV-5B, PST, and ESM-2-pt. Solid lines: RNA viruses only; lighter lines: all viruses. Despite the greater complexity of RNA viruses, our models maintain consistent improvements over baselines.

- **Distance-matched** (3 models). 50 random pairs drawn to match the genomic-distance range of the inferred pairs, averaged over three draws.
- **No-link** (1 model). Only neighboring proteins are connected.

We used the ESM-2-35M-parameter model so that all eight could be trained from scratch under identical conditions. All models share the same architecture, initial weights, training data and order, masking, and optimizer, so any difference in their results can come only from which pairs are connected.

Table S3 reports held-out perplexity on 600 genomes. Adding 50 random links leaves perplexity essentially unchanged relative to having no long-range links (16.124 versus 16.120), whereas adding the same number of inferred links lowers it (lower is better). The distance-matched control performs like the other controls, so the advantage reflects which proteins are paired rather than how far apart they occur.

Model	Held-out perplexity
No long-range links	16.120
50 random links (average of 3)	16.124
50 distance-matched links (average of 3)	16.119
50 inferred links	**16.064**

Supplementary Table S3: **Held-out perplexity for the interaction-inference controls.** Lower is better. Only the inferred links lower perplexity relative to having no long-range links.

The genome-wide change is modest (approximately 0.4%), but this figure represents an average across all positions in the genome. Because the 50 links are relevant only to the minority of positions that occur within an inferred PPI link, the majority of positions are unaffected. Averaging this small number of positions against the large number of unaltered ones minimizes the magnitude of the effect. The effect is nonetheless consistent across genomes as the inferred model achieves lower perplexity than the random average on 554 of 600 genomes (92%), and it outperforms the lowest perplexity attained by any of the three random models on a per-genome basis for 78% of genomes.

The improvement in perplexity is also concentrated among the pairs assigned the highest scores by the inference model. We ranked positions according to the reduction in per-position loss conferred by the inferred links relative to the control,

and examined the distribution of the most-improved positions. In Table S4, the baseline rate denotes the fraction of the genome occupied by the proteins in the retained pairs—the value expected by chance—and the top-100 enrichment quantifies how much more frequently the 100 most-improved positions coincide with those pairs than this baseline predicts. The enrichment increases with the confidence of the pairs considered, ranging from approximately 1.4-fold when all 50 pairs are pooled to approximately 11-fold for the highest-scoring pairs. The improvement therefore scales with the model's inferred PPI score rather than emerging at an arbitrary threshold.

Edges kept (by confidence)	Baseline rate	Top-100 enrichment
top 2%	4.1%	11.1×
top 5%	9.0%	7.2×
top 10%	13.8%	5.2×
top 20%	25.6%	3.1×
top 50%	50.4%	1.7×
all inferred pairs	71.6%	1.4×

Supplementary Table S4: **Enrichment of improved positions on high-scoring inferred pairs.** The most-improved positions fall disproportionately on the proteins the inference model ranks most highly.

With every other setting held identical, 50 randomly chosen links give no measurable improvement over having no long-range links, whereas 50 links chosen by the inference model lower held-out perplexity. The advantage is consistent across genomes and concentrates on the pairs the model scores highest, which are the same pairs an independent, separately validated model ranks as most likely to interact.

Manuscript With Tracked Changes

Click here to access/download;Manuscript;final_pdf_tracked_changes_5_8_26

Extending Protein Language Models to a Viral Genomic Scale Using Biologically Induced Sparse Attention

Thibaut Dejean
Department of Information and Computer Sciences
University of Hawaii
Honolulu, HI
Barbra D. Ferrell
Department of Computer & Information Sciences
Delaware Biotechnology Institute
University of Delaware, Newark, DE

Zachary D. Schreiber
Department of Computer & Information Sciences
Delaware Biotechnology Institute
University of Delaware, Newark, DE

William Harrigan
Hawai'i Institute of Marine Biology
University of Hawai'i
Kaneohe, HI

Rajan Sawhney
Department of Information and Computer Sciences
University of Hawaii
Honolulu, HI

K. Eric Wommack
Department of Plant & Soil Sciences
Delaware Biotechnology Institute
University of Delaware, Newark, DE

Shawn W. Polson
Department of Computer & Information Sciences
Delaware Biotechnology Institute
University of Delaware, Newark, DE

Mahdi Belcaid*
Department of Information and Computer Sciences
University of Hawaii
Honolulu, HI
mahdi@hawaii.edu
*Corresponding author

Abstract

The transformer architecture in deep learning has revolutionized protein sequence analysis. Recent advancements in protein language models have paved the way for significant progress across various domains, including protein function and structure prediction, multiple sequence alignments and mutation effect prediction. A protein language model is commonly trained on individual proteins, ignoring the interdependencies between sequences within a genome. However, biological understanding reveals that protein–protein interactions span entire genomic regions, underscoring the limitations of focusing solely on individual proteins. To address these limitations, we propose a novel approach that extends the context size of transformer models across the entire viral genome. By training on large genomic fragments, our method captures *putative* long-range dependencies consistent with inter-protein relationships and encodes protein sequences with integrated information from distant proteins within the same genome, offering benefits across downstream tasks. Viruses, with their densely packed genomes, minimal intergenic regions, and protein annotation challenges, are ideal candidates for genome-wide learning. We introduce a long-context protein language model, trained on entire viral genomes, leveraging a biologically informed sparse attention mechanism in which inter-protein links are *inferred computationally* and used as sparsity priors. Our semi-supervised

approach supports long sequences of up to 61,000 amino acids (aa). Our evaluations show improved ~~perplexity~~ prediction of masked amino acids and improved downstream discrimination relative to single-protein models and long-context baselines, with additional validation that our inferred links correlate with independently curated interaction resources.

1 Introduction

Deep learning has transformed the analysis of genomic data~~, with the field evolving from recurrent neural networks (RNNs) to~~ , driven by transformer models [1, 2]. This ~~advancement has spawned~~ shift produced powerful protein language models such as ESM-2, ProtTrans, and ProGen~~, which excel in capturing the complex relationships between amino acids (aa) within proteins~~ [3, 4, 5], which capture complex relationships between amino acids (aa) within proteins and yield high-dimensional embeddings that are effective across tasks including protein function annotation [6], multiple sequence alignment [7], and structure prediction [8]. ~~These models create rich, high-dimensional embeddings—mathematical representations of proteins—that have proven to be remarkably effective for various challenging tasks, including annotation of protein functions, multiple sequence alignment, and structure prediction.~~

However, ~~these advances come with an important limitation: by~~ by focusing on individual proteins, ~~current~~ these models miss crucial interactions between distant proteins that shape genome ~~functionality~~ function. While proximity-based interactions~~,~~ such as those within operons~~,~~ ~~have been extensively studied~~ are well studied [9], growing evidence highlights ~~the significance of~~ long-range protein interactions across ~~vast~~ large genomic distances [10]~~. These distant relationships present a compelling parallel to natural language structures, where coherence and meaning often depend on connections between distant elements. This biological reality is particularly evident in evolutionary processes—when~~ ; in evolutionary terms, when one protein changes, its ~~partner proteins~~ partners often must adapt to ~~maintain~~ preserve their collective function [11]. ~~Current~~ Yet transformer-based models, constrained by limited context windows (e.g., ESM-2 is limited to 1,024 aa, or tokens), cannot ~~simultaneously process multiple long proteins. This technical barrier prevents fully understanding and modeling the~~ process multiple long proteins simultaneously, preventing them from modeling the web of protein interactions across entire genomes. ~~complex web of protein interactions across entire genomes.~~

Recent large language models (LLMs) show that extended context improves ~~a model's ability to maintain coherence and capture~~ the capture of long-range relationships in text [12]~~. We hypothesize that~~ , suggesting that analogous approaches could yield more accurate protein embeddings and ~~potentially~~ reveal ~~crucial~~ functional relationships between ~~distantly related proteins missed by current methods~~ distant proteins that current methods miss. However, extending context ~~in language models presents significant computational challenges, primarily due to the quadratic scaling of attention mechanisms with sequence length~~ is computationally demanding, primarily because attention scales quadratically with sequence length [13]. ~~While various solutions have emerged—from~~ Existing solutions—from sparse attention patterns to ~~efficient architectural modifications—these approaches are fundamentally~~ architectural modifications—are optimized for text ~~processing~~ and do not account for the ~~unique~~ principles of protein biology, ~~potentially limiting their effectiveness when applied to~~ limiting their effectiveness on multiple protein sequences.

To address these limitations, we introduce a ~~novel~~ protein language model ~~architecture capable of processing~~ that processes entire viral genomes~~. Our model achieves~~ , achieving a context window exceeding 60,000 aa on a single commodity GPU~~. This expanded capacity enables capturing both local protein features and genome-wide interactions in their native genomic context. Notably, this context length covers~~ —covering 98% of all ~~known~~ sequenced viral genomes in the NCBI ~~virus~~ Virus database [14] (Supplementary Figure S1)~~, representing a significant advancement in studying protein relationships at a genomic scale~~. Our semi-supervised ~~protein language~~ model employs sparse attention ~~mechanisms~~ guided by ~~known~~ computationally inferred protein~~—~~protein interactions~~. This innovation extends the boundaries of protein sequence modeling and provides new insights into long-range interactions shaping viral genome organization and function. Comprehensive evaluations demonstrate~~ , and our evaluations show that ~~our~~ the resulting viral genome embeddings capture biologically meaningful signals missed by current single-protein models~~, establishing a novel framework for understanding protein relationships in their native genomic context~~.

2 Related Work

2.1 Long Context LLMs

Recent advances in large language models have sparked intense research into efficiently handling long contexts. Approaches to increasing context length fall broadly into four popular categories [12] : 1) context length extrapolation, 2) hardware and algorithmic strategies, 3) prompt compression, and 4) attention matrix approximation. Context length extrapolation methods enable models to process sequences longer than those on which they were initially trained

by manipulating positional embeddings. Hardware and algorithmic strategies focus on optimizing computation and memory usage through system optimizations. Prompt compression methods reduce the sequence length by condensing the input information into more compact representations before processing. Lastly, attention matrix approximation employs computational and statistical strategies to estimate attention mechanisms without incurring the quadratic scaling costs of standard attention. We discuss these four approaches in detail below;We briefly review these approaches below since our approach builds on advances from several of them. From context length extrapolation methods (Section 2.1.1), we adopt ALiBi positional encoding for stable inference on extended sequences. From attention matrix approximation strategies (Section 2.1.4), we build on sparse attention patterns similar to Longformer, but replace fixed patterns with biologically informed sparsity derived from predicted inferred protein-protein interactions (PPI). We leverage ESM-2 both as a foundation for PPI inference and as initialization for one of our models. This section provides background on each of these areas to contextualize our contributions.

2.1.1 Context Length Extrapolation

A fundamental limitation in extending transformer models to longer sequences lies in Positional Encodings (PEs). While PEs are essential for encoding the sequential order of tokens, their effectiveness diminishes significantly when processing sequences that exceed the training data length. This constraint represents a critical bottleneck when adapting models trained on shorter sequences to handle longer inputs.

Early architectures used fixed sinusoidal positional encodings (SinPE) [13], while newer approaches such as Approaches such as rotational position embeddings (RoPE) [15] introduced trainable embeddings that apply position-based rotations to query and key representations. However, traditional PEs suffer substantial performance degradation when processing sequences that are longer than their training context. For example, models that use RoPE or SinPE exhibit significant increases in perplexity, indicating poorer predictive performance, when used to infer sequences that exceed the lengths encountered during training [16]. To overcome this limitation, context length extension strategies have evolved, enabling efficient processing of long sequences without requiring comprehensive model retraining. These solutions primarily utilize two approaches: extrapolation and interpolation. Two families of methods address this without full retraining: extrapolation and interpolation. Extrapolation methods, exemplified by Attention with Linear Biases (ALiBi) [16], introduce unlearned static biases into attention calculations, systematically adjusting attention scores between key–query pairs based on distance. In contrast, interpolation methods generalize learned PEs to longer sequences: RoPE-based interpolation linearly scales position indices to match the original context window [17], whereas more sophisticated methods such as Yet another RoPE extensioN method (YaRN) [18] employ nonuniform frequency interpolation to preserve positional information more effectively, for example by scaling RoPE position indices to the original context window [17].

2.1.2 Hardware and Algorithmic strategies

Training transformer models directly on longer sequences would eliminate the need for positional embedding extrapolation techniques. However, this approach is fundamentally constrained by the quadratic memory complexity inherent to the attention mechanism. Specifically, standard attention mechanisms require memory and computational complexity of $O(n^2d + nd^2)$ per layer, where n represents the sequence length and d denotes the hidden dimension size. This quadratic scaling imposes substantial hardware limitations: for example, a Tesla V100 GPU with 16 GB RAM can handle significantly shorter sequences compared to an NVIDIA A100 GPU with 80 GB HBM2e memory, assuming identical model architectures and batch sizes.

To overcome these scaling challenges, researchers have pursued multiple complementary approaches. Distributed computing architectures leverage Distributed computing architectures address these constraints by leveraging model parallelism, distributing the memory and computation demands of attention across multiple GPUs.

Additionally, recent Recent algorithmic innovations have substantially improved the efficiency of attention computation without altering its theoretical complexity. For example, FlashAttention [19] optimizes memory access patterns through fused kernel operations implemented in specialized computing frameworks such as Triton [20]. These practical optimizations reduce memory requirements significantly while preserving computational performance, thus enabling the processing of longer sequences within existing hardware constraints. Together, these hardware advancements, distributed computing strategies, and memory-efficient algorithms establish a robust framework for extending transformer models to process substantially longer sequences.

2.1.3 Prompt Compression

Prompt compression methods aim to reduce input dimensionality by exploiting redundancy within input sequences, ideally preserving semantic content. Techniques employed range from straightforward token-level filtering to sophisti-

cated semantic compression algorithms [21]. Recent compression methods have demonstrated compression ratios of up to 95% without substantially degrading downstream task performance [22]. However, this approach introduces a fundamental limitation in protein sequence analysis: compressed inputs cannot generate detailed per-residue embeddings for every amino acid, as compression inherently reduces sequence representation granularity. Consequently, prompt compression is less suitable for tasks requiring residue-level detail, such as protein structure prediction or mutation effect prediction.

2.1.4 Attention Matrix Approximation

An alternative approach to managing extended contexts involves approximating rather than explicitly computing the full attention matrix. This strategy includes two primary methodologies: context-agnostic and context-aware sparse matrices, each offering distinct trade-offs between computational efficiency and representational capacity.

Context-Agnostic Sparse Matrices. Context-agnostic approaches address the quadratic complexity of traditional self-attention through systematic approximation techniques [13]. These methods encompass low-rank matrix decomposition [23], sparsity-induced dimension reduction, and simplified softmax normalization [24]. Among the most successful implementations are architectures leveraging predefined sparse attention patterns, exemplified by Longformer, LongLoRA, and BigBird [25, 26, 27], each employing distinct sparsification strategies (Figure 1). Longformer implements dilated sliding-window attention, strategically positioning sparse attention windows around tokens (Figure 1a). LongLoRA extends context length by introducing shifted attention patterns during fine-tuning (Figure 1b). BigBird employs a hybrid approach combining global attention for select tokens, sliding-window attention, and random attention connections (Figure 1c). While BigBird enables whole-sequence consideration, the stochastic nature of random attention introduces variability in performance, as randomly selected attention positions lack inherent semantic or functional relevance. Although these approaches successfully extend context windows, their predetermined patterns remain independent of sequence content and semantic meaning, presenting inherent limitations. Consequently, context-agnostic designs may inadvertently omit critical interactions or excessively emphasize irrelevant sequence elements. Most notably for biological applications, these methods cannot incorporate domain-specific knowledge—such as experimentally validated protein–protein interactions or genomic relationships—into attention calculations.

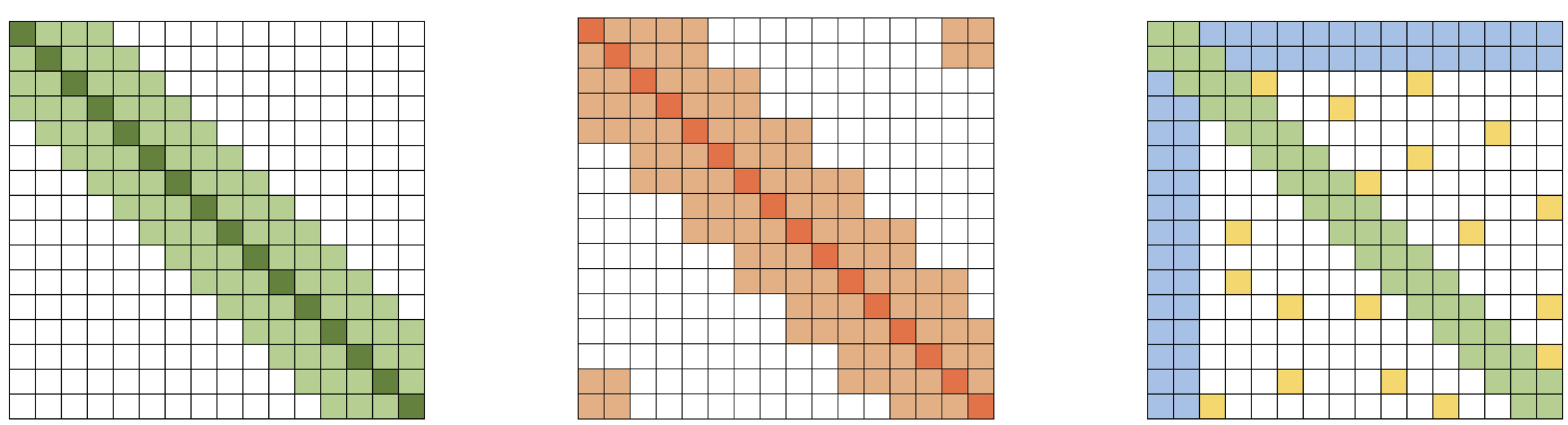

(a) Longformer (b) LongLoRA (c) BigBird

Figure 1: **Sparse attention patterns implemented in context-agnostic transformer architectures. (a)** Longformer's dilated sliding-window attention pattern, showing a diagonal band of attention with varying intensities of green. **(b)** LongLoRA's shifted attention pattern during fine-tuning, displayed as orange blocks. **(c)** BigBird's hybrid approach combining global attention (blue columns), sliding-window attention (green diagonal), and random attention connections (scattered yellow squares). These predetermined sparse attention patterns reduce computational complexity while enabling longer context windows, though they operate independently of sequence content and semantic meaning.

Context-Aware Sparse Matrices. In contrast to context-agnostic methods, context-aware compression approaches dynamically adjust their attention patterns based on input content. For example, the Routing Transformer [28] employs K-means clustering on key and query vectors to construct sparse, content-dependent attention matrices. This clustering-based routing enables efficient attention computation between distant sequence elements by restricting attention interactions to intra-cluster relationships. Consequently, memory complexity is effectively reduced from quadratic to linear scaling relative to sequence length, as the attention computation becomes bounded by cluster size rather than the total input sequence length.

However, the exclusive reliance on clustering for routing attention presents significant limitations when analyzing biological sequences. Crucially, clustering-based methods may fail to capture interactions between functionally related

but structurally and sequentially distinct elements, such as complementary protein domains that co-evolved despite limited sequence similarity. ~~Indeed, biologically critical interactions frequently occur between proteins exhibiting highly divergent sequences and distinct structural characteristics, challenging the effectiveness of purely clustering-based approaches.~~

2.2 Protein & Genome Language Modeling

2.2.1 Protein Language Models

Current popular protein language models (pLMs), including ProtBert [29], ProtTrans [4], and ProGen2 [5], utilize transformer-based architectures with context windows ranging from 512 aa (ProtTrans-XL) to 2,048 aa (ProGen2). These context limitations have largely confined pLMs to analyzing individual proteins in isolation, neglecting their broader genomic context. Among these, the ESM-2 model [3], trained on the UniRef50 database [30] via masked language modeling, has emerged as one of the most widely adopted pLMs. ESM-2 demonstrates impressive performance in protein contact prediction—attributed to its effective attention mechanism—and generates biologically meaningful embeddings useful across diverse downstream applications [6, 7, 8]. However, ESM-2, like its predecessors, focuses exclusively on single-protein sequences with a restricted context length of only 1,024 aa, thereby limiting its ability to capture inter-protein relationships within genomic contexts.

To integrate genomic context into protein representations, the Protein Set Transformer (PST) [31] introduces a graph-based framework wherein proteins, initially embedded via ESM-2, form nodes connected by edges representing genomic proximity. ~~PST propagates contextual information through a multi-head attention mechanism, incorporating learnable positional embeddings that encode protein locations within the genome. By combining local sequence features (ESM-2 embeddings) with genomic relational context (graph representation of proximal interactions), PST refines protein-level embeddings to reflect neighborhood information and generates genome-level embeddings that adaptively weigh protein importance.~~ Despite these advantages, PST relies on precomputed single-protein ESM-2 embeddings and is constrained to modeling interactions between consecutive proteins.

2.2.2 Genomic Language Models

Recognizing the inherent limitations of analyzing proteins in isolation has spurred the development of genomic language models (gLMs) [32], which augment ESM-2 embeddings by incorporating context from larger genomic regions. For instance, recent gLMs utilize 30-gene fragments in a masked prediction task, enabling the prediction of protein embeddings by considering the contextual influence of 29 neighboring proteins. This approach generates enriched representations that significantly improve performance across tasks such as protein function prediction and taxonomic classification. ~~Parallel developments in DNA sequence modeling have yielded architectures capable of processing substantially longer contexts and capturing gene-level dependencies across extensive genomic regions. Prominent examples include Evo [33], DNABert-2 [34], and GenSLM [35], each employing distinct strategies for handling extended sequence lengths. Evo implements a hybrid architecture combining Hyena layers [36] with traditional transformers, initially training on sequences of 8,000 nucleotides and subsequently scaling to 131,000 nucleotides via the StripedHyena architecture [37]. DNABert-2 leverages Attention with Linear Biases (ALiBi) positional encoding, enabling effective inference on sequences significantly longer than its initial 128 token (768 nucleotide) training context without relying on learned positional embeddings. GenSLM adopts a progressive strategy, starting with 2,048-token sequences and extending to 10,000 tokens during fine-tuning using model compression techniques and specialized hardware (Cerebras CS-2). These advances in genomic-scale language modeling represent a meaningful shift from protein-centric analyses, enabling exploration of complex interactions and long-range dependencies across extensive genetic sequences.~~ However, existing gLMs primarily utilize conventional attention mechanisms, highlighting potential opportunities for incorporating biologically informed sparse attention patterns to further enhance their capabilities.

While existing methods have significantly advanced general language modeling and biological sequence analysis, important limitations remain. Current context-extension techniques in language models typically lack biological specificity. Although extrapolation methods such as ALiBi demonstrate effectiveness in general language tasks, they inherently assume consistent structural patterns between short and extended sequences (for instance, local grammatical structures remain stable regardless of sequence length). This assumption does not hold for multi-protein sequences, where relationships between distant proteins can fundamentally differ from the local sequence patterns learned by single-protein models. Additionally, while content-dependent sparse attention mechanisms show promise for managing extended contexts, their potential for explicitly incorporating biological priors into language model architectures remains largely unexplored.

To address these limitations, we introduce a novel genome-scale protein language model architecture specifically designed for viral sequences. Our approach synthesizes recent advances in context-length extension methods with

biologically informed sparse attention mechanisms, enabling the effective processing of significantly longer amino acid sequences on commodity hardware while explicitly modeling inter-protein interactions. The following sections detail our methodology and validate its effectiveness across multiple downstream biological tasks.

3 Methods

3.1 Datasets and Preprocessing

Sources and retrieval. We used the NCBI Virus database (retrieved 2024-06), selecting only genomes annotated as "complete" (14,436 genomes) (Supplementary Materials S1, Supplementary Figure S1). The list of loci used is included in the Github page (`https://github.com/Hawaii-Bioinformatics/ViralEmbed`). For downstream classification tasks, the final dataset comprised 100,000 proteins from 5,000 genomes after de-duplication.

Protein non-redundancy. To prevent highly abundant sequences from biasing training, we applied CD-HIT clustering at 90% sequence identity, reducing the dataset by approximately 50%. Some viral proteins occur dozens or hundreds of times in the database; without deduplication, such sequences would dominate gradient updates and bias learned representations. This threshold collapses near-identical sequences while preserving biologically distinct variants.

Splits. To prevent data leakage, we partitioned the dataset at the genome level: all proteins from a given genome were assigned to the same split (train, validation, or test). Splits were stratified by taxonomic species to maintain balanced representation across partitions. The final proportions were 80% train, 10% validation, and 10% test. This design ensures that no protein from a genome in the test set could have been seen during training, either directly or through related proteins from the same genome.

Fragments. For long-context baselines, we constructed fragments by concatenating proteins in genomic order with separators, truncating or padding to target lengths as specified.

Metadata. For downstream labels we used (i) NCBI taxonomy (species/family), and (ii) Pfam domain family assignments from HMMER3 against Pfam 35.0 to create task-aligned labels used in Sections 3.8 and 3.9.

3.2 Training and Evaluation Pipeline Overview

Our approach involves three distinct stages, each employing different models (Figure 2):

Stage 1 – PPI Inference Model (ESM-2-PPI): Training protein language models on entire viral genomes is computationally demanding due to the quadratic scaling of attention mechanisms with sequence length. While existing sparse attention approaches can capture co-evolutionary signals, they typically use static or random masking patterns that neglect biological context. To overcome this, we develop a biologically informed sparse attention method that restricts attention computations exclusively to protein pairs identified as likely interaction partners. Ideally, experimental PPI databases would guide this identification; however, the limited coverage of viral genomes in these databases necessitated a computational strategy. Specifically, we fine-tune ESM-2 (650M parameters) with ALiBi positional encoding (selected after comparing against RoPE; see Supplementary Materials), LoRA adaptation, and Longformer-style sliding window attention (selected after comparing against Shifted Sparse Attention and BigBird; see Supplementary Materials). This extends the effective context window to approximately 13,000 aa, enabling analysis of multi-protein genomic fragments. This model is used exclusively for extracting attention scores to infer PPIs and is not employed for any downstream embedding or classification tasks. Section 3.3 describes the optimization of this model, and Section 4.1 presents the results.

Stage 2 – Genome-Scale Language Models (LV-3C and LV-5B): Using the PPIs identified in Stage 1 as biologically informed sparsity priors, we train two novel long-context protein language models. Both models use identical sparse attention patterns; they differ in scale and initialization:

- **LV-3C** (317M parameters; 16 layers): Trained from scratch on sequences up to 61,000 aa. Its lighter architecture prioritizes processing longer genomic context.
- **LV-5B** (756M parameters; 33 layers): Initialized from ESM-2 (650M) weights and trained on sequences up to 48,000 aa. Its larger architecture prioritizes richer pretrained representations.

This complementary design allows us to evaluate two strategies for incorporating genomic context: maximizing sequence length versus leveraging pretrained knowledge. These models represent the primary contribution of this work and are described in Sections 3.3–3.4.

Stage 3 – Baseline Models for Evaluation: To benchmark LV-3C and LV-5B, we compare against several ESM-2 variants:

- **ESM-2-pt**: The original pretrained ESM-2 model with a 1,024 aa context window.
- **ESM-2-RoPE**: ESM-2 fine-tuned with RoPE positional encoding for extended context inference.
- **ESM-2-ALiBi**: ESM-2 fine-tuned with ALiBi positional encoding for extended context inference.

These baselines isolate the contribution of our biologically informed sparse attention mechanism from general context extension strategies.

Throughout the manuscript, we use these standardized names to refer to each model variant. The following sections describe each stage in detail: Section 3.3 covers Stage 1 (PPI inference), Sections 3.4–3.5 cover Stage 2 (model training), and Sections 3.6–3.11 cover Stage 3 (evaluation).

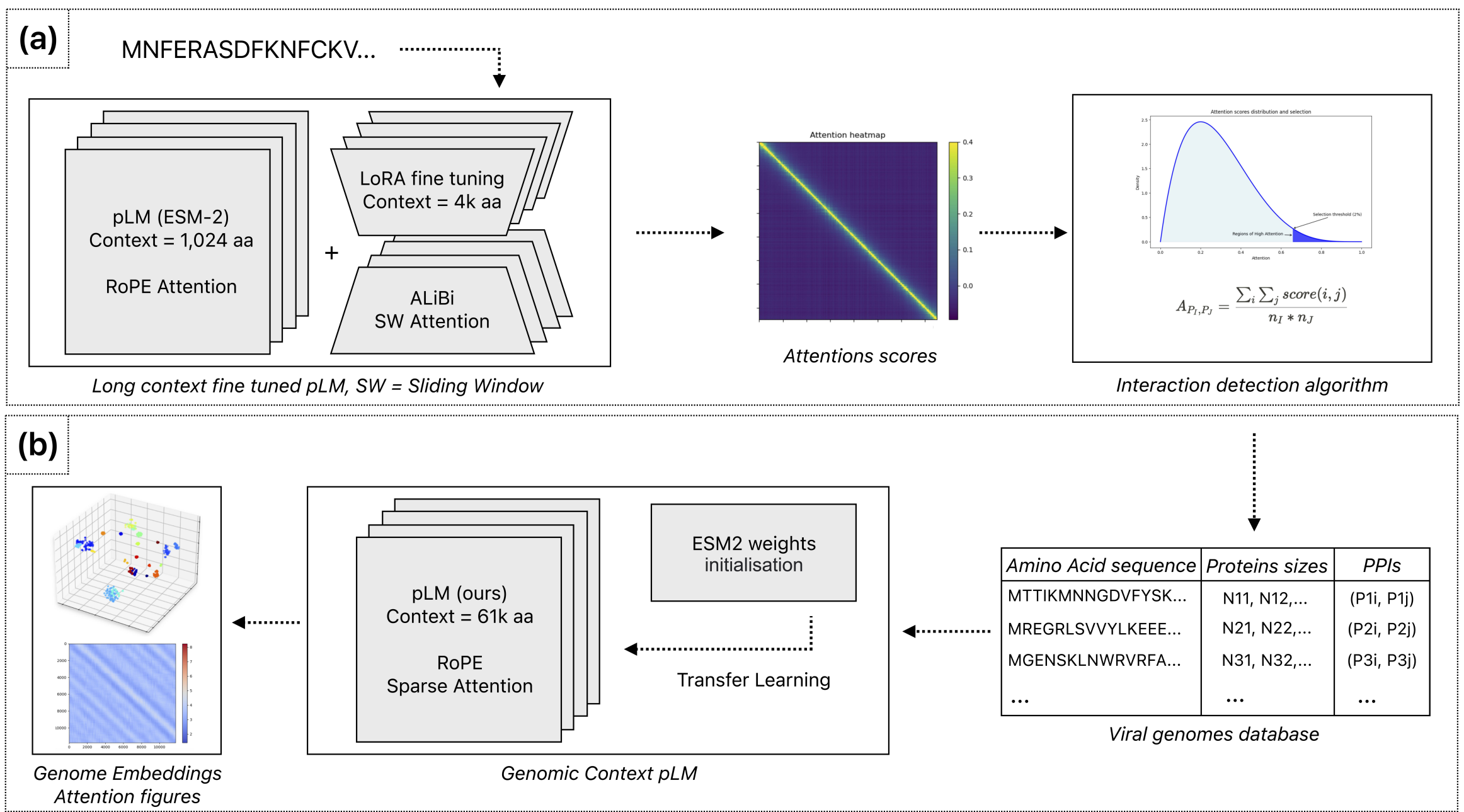

Figure 2: **Two-stage approach for training a protein language model on complete viral genomes. (a)** Identifying putative protein-protein interactions on multi-protein fragments using an extended-context ESM-2 model combining LoRA fine-tuning and ALiBi positional extrapolation. The attention scores produced by this model are subsequently used to inform the protein interaction detection algorithm. **(b)** Training of two long-context protein language models from scratch or initialized from ESM-2 weights using sparse attention patterns guided by ~~predicted~~ inferred protein interactions, enabling efficient whole-genome embedding generation.

3.3 ~~Optimizing~~ Developing the PPI Inference Model (Stage 1)

To extend ESM-2's context for PPI inference, we evaluated multiple approaches for achieving a context window of 13,000 aa (Supplementary Materials S2). Specifically, we compared two positional encoding strategies (RoPE and ALiBi) (Supplementary Figure S2) and three sparse attention mechanisms (Longformer, Shifted Sparse Attention, and BigBird) (Supplementary Table S1). Performance was benchmarked against the baseline ESM-2 650M model using both perplexity, which quantifies how well the model predicts amino acid sequences (lower is better), and silhouette scores, which measure how well protein embeddings cluster by structural similarity (higher is better).

PPIs can be inferred from attention scores between proteins in language models, as demonstrated by previous work. For instance, [38] showed that specific attention heads in ESM-1b capture distinct protein relationships, and subsequent work extended these findings to larger models including ESM-2 [3]. Our approach builds upon this concept by analyzing

attention patterns simultaneously across multiple proteins within viral genomes to predict potential interactions. Standard protein language models, such as ESM-2, have a context length limitation of 1,024 aa, restricting analysis to only 3–5 average-length proteins at once. To overcome this, we first extended ESM-2's context window to 4,000 aa using attention matrix approximation, and subsequently increased it further to 13,000 aa through context length extrapolation (ALiBi). For the initial extension, ESM-2 was fine-tuned on concatenated protein fragments from the NCBI Viral database [14] using an NVIDIA A6000 GPU with 48 GB RAM. Protein fragments were constructed by sequentially concatenating complete proteins in their genomic order until reaching the 4,000 aa threshold, padding as necessary. To enable efficient training on standard GPU hardware, we used LoRA for parameter-efficient fine-tuning, while Longformer was selected as the sparse-attention pattern based on the comparisons in Supplementary Table S1.

Interaction Threshold Selection. A key parameter in our sparse attention mechanism is k, the number of top-ranked non-adjacent protein pairs included in the attention mask for each genome. To select an appropriate value, we varied $k \in \{10, 20, 35, 50, 100\}$ and evaluated downstream performance on taxonomy and Pfam classification tasks (Supplementary Materials S2). We also tested an alternative policy that includes genomically adjacent pairs in the ranking. Supplementary Table S2 shows that $k = 50$ maximizes F1 score with modest computational cost, while including adjacency during selection provides no benefit since consecutive protein attention is already explicitly included in the sparse pattern (see Section 3.4).

For each protein fragment analyzed by the fine-tuned model, attention matrices from all encoder layers were extracted. For each layer ℓ and attention head h, we denote the token-level attention matrix as $M^{(\ell,h)} \in \mathbb{R}^{L\times L}$ and use p, q, r, s for token positions. Each matrix was normalized and symmetrized using Average Product Correction (APC), yielding $N^{(\ell,h)}$ computed as [3]:

$$APC^{(\ell,h)}_{p,q} = \frac{\sum_{s=1}^{L} M^{(\ell,h)}_{p,s} \; \sum_{r=1}^{L} M^{(\ell,h)}_{r,q}}{\sum_{r=1}^{L} \sum_{s=1}^{L} M^{(\ell,h)}_{r,s}},$$
$$\widetilde{M}^{(\ell,h)}_{p,q} = M^{(\ell,h)}_{p,q} - APC^{(\ell,h)}_{p,q},$$
$$N^{(\ell,h)}_{p,q} = \frac{1}{2}\left(\widetilde{M}^{(\ell,h)}_{p,q} + \widetilde{M}^{(\ell,h)}_{q,p}\right).$$

Here, ℓ indexes layers, h indexes attention heads, and $p, q, r, s \in \{1, \dots, L\}$ index amino-acid positions within the sequence.

From each normalized attention matrix $N^{(\ell,h)}$, we define a set $S^{(\ell,h)}$ containing the top $t_{\text{aa}}\%$ highest attention values between amino-acid pairs. We used $t_{\text{aa}} = 2$, corresponding to the top 2% of amino-acid attention values; this amino-acid-level threshold is separate from the final protein-pair threshold $k = 50$:

$$S^{(\ell,h)} = \{(p, q) \mid N^{(\ell,h)}_{p,q} > \text{Quantile}_{1-\frac{t_{\text{aa}}}{100}}(N^{(\ell,h)})\}.$$

Attention values within the set S were subsequently converted to scores via min-max normalization. These normalized scores were then aggregated to compute an attention-based interaction score A_{P_I,P_J} between protein pairs P_I and P_J, having lengths n_I and n_J, respectively:

$$A_{P_I,P_J} = \frac{\sum_{p\in P_I} \sum_{q\in P_J} \text{score}(p, q)}{n_I \times n_J} \quad \text{with} \quad (p, q) \in S^{(\ell,h)}.$$

Interaction scores were computed for all protein pairs within the genome. Preliminary analyses indicated that genomically adjacent proteins consistently yielded the highest attention scores (Supplementary Figure S3), likely reflecting genuine biological interactions. Therefore, for constructing the sparse attention matrix used in subsequent model training, only the top 50 ranked scores from non-consecutive protein pairs were included as interacting proteins, with the remaining pairs masked. This threshold of 50 interactions represents a balance between computational efficiency and interaction coverage appropriate for the viral genome sizes analyzed (Supplementary Figure S4). The threshold parameter can be adjusted based on genome sequence length and available computational resources.

To calibrate the number of protein pairs selected for sparse attention on biologically relevant data, we investigated interaction patterns among Family A DNA polymerase (PolA), ribonucleotide reductase (RNR), and helicase (HEL) (Supplementary Figures S3, S4, Supplementary Table S2). These proteins constitute a well-characterized interaction network [39] and display strong evolutionary coupling due to their coordinated roles in DNA replication [40, 41].

3.4 Sparse Attention Computation (Stage 2)

Once the PPIs have been identified and selected using the mechanism described above, it is possible to proceed with training a long-context pLM. Model training is based on a sparse attention mechanism, described below, and represented in Figure 3.

To maintain biological fidelity when computing cross-protein attention, we developed a two-stage fragmentation approach. First, we segment the viral genome precisely at protein boundaries, preserving each protein as a distinct unit. Second, we subdivide each protein into uniform blocks of fixed length B, resulting in a block-level representation of the viral genome. We then map the previously identified PPIs onto these blocks, creating fragment-level interaction pairs that guide our sparse attention model. This transformation preserves biologically meaningful relationships while enabling efficient computation across the entire viral genome.

Specifically, given two interacting proteins P_1 and P_2, we transfer these interactions to interactions between their constituent blocks:

$$(P_1, P_2) \Rightarrow (B_{1_i}, B_{2_j}), \quad i \in [1, n_1],\ j \in [1, n_2]$$

where:

- P_1, P_2 are two interacting proteins from genome G.
- $B_{1,i}$ is the i-th block of protein P_1, with n_1 total blocks.
- $B_{2,j}$ is the j-th block of protein P_2, with n_2 total blocks.

For each pair of blocks $(B_{1,i}, B_{2,j})$, we compute the block-level attention scores using the scaled dot-product attention formula:

$$Att_G(B_{1,i}, B_{2,j}) = \frac{Q_{B_{1,i}} K_{B_{2,j}}^T}{\sqrt{d_k}}$$

where:

- $B_{1,i}$, $B_{2,j}$ are the blocks from genome G being considered
- $Q_{B_{1,i}}$ is the query vector subset from genome G corresponding to block $B_{1,i}$.
- $K_{B_{2,j}}$ is the key vector subset from genome G corresponding to block $B_{2,j}$.
- d_k is the dimensionality of the key vectors.

Efficient computation of sparse attention requires specialized data structures optimized for block operations. We utilize the Block Sparse Row (BSR) tensor format [42], which stores non-zero elements in fixed-size blocks, significantly reducing both memory consumption and computational overhead. Empirical testing identified a block size $B = 32$ as optimal, balancing computational efficiency and minimal padding overhead in our viral protein datasets.

In our implementation, each query–key product $Q_{F_i}(K_{F_j})^T$ between fragment pairs $B_{1,i}$ and $B_{2,j}$ produces a 32×32 tensor, directly aligning with BSR's block structure. We subsequently apply softmax normalization followed by multiplication with the corresponding value vectors using optimized Triton [20] kernels tailored for block-sparse operations. Finally, we remove padding tokens to restore original sequence dimensions using precomputed position indices (Algorithm 1).

3.5 Model Training (Stage 2)

The training was conducted using the NCBI Virus database (retrieved on 06/2024), comprising 14,436 complete viral genomes totaling 683 million aa (Supplementary Figure S1). The database represents a comprehensive reference set of viral diversity, ensuring robust generalization across viral families. All genomes were segmented at protein boundaries and padded appropriately to meet sequence length constraints. All training was conducted using NVIDIA A6000 GPUs (48 GB RAM) with block-sparse computations optimized through the Triton library [20].

The long-context protein language models represent two main variants, designed to produce complementary types of embeddings. These two models differ in scale, with LV-3C being the lighter version (317M parameters) and LV-5B the larger one (756M parameters).

The two models also differ in their initialization and training strategies:

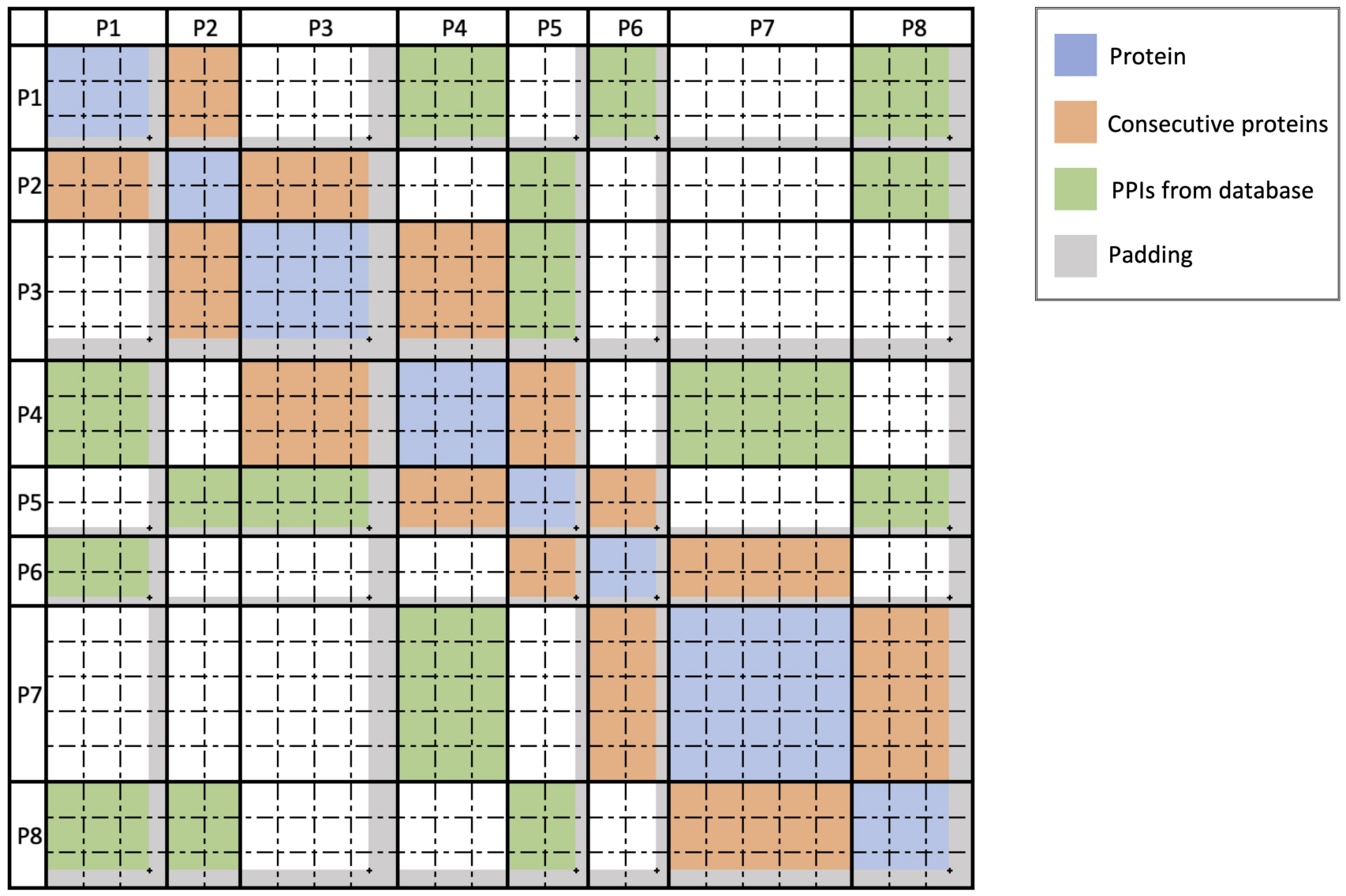

Figure 3: **Sparse attention computation architecture.** Example attention matrix demonstrating four distinct protein interaction patterns considered during sparse attention computation: intra-protein (self) attention (blue), interactions between consecutive proteins (orange), putative protein–protein interactions identified by our method (Section 3.3) and stored in a database (green), and protein pairs not identified as interacting (white), for which attention calculations are omitted. Gray regions indicate padding introduced during fragment construction.

- LV-3C: Randomly initialized and trained from scratch on genomic segments (up to 61,000 amino acids) with an initial learning rate of 3×10^{-4}, using biologically informed sparse attention for 12 epochs.
- LV-5B: Trained leveraging transfer learning from the ESM-2 model (650M parameters) on genomic segments (up to 48,000 amino acids), using the same biologically informed sparse attention with an initial learning rate of 3×10^{-5}, also for 12 epochs.

This two-model design aims to capture complementary aspects of viral genomic information. LV-3C prioritizes the modeling of long-range genomic context through a lighter architecture that enables the processing of extended sequences, whereas LV-5B prioritizes richer pretrained representations through a larger model operating on shorter segments.

3.6 Evaluation Metrics (Stage 3)

We employ two primary metrics throughout our evaluations:

Perplexity measures how well the model predicts amino acid sequences, quantifying the model's uncertainty when predicting masked tokens. It is defined as the exponentiated average cross-entropy loss:

$$\text{Perplexity} = \exp\left(-\frac{1}{N}\sum_{i=1}^{N}\log P(x_i|\text{context})\right)$$

where N is the number of masked tokens and $P(x_i|\text{context})$ is the predicted probability of the true amino acid x_i. Lower perplexity indicates better predictive performance. This metric is particularly important for assessing whether models maintain consistent prediction quality as sequence length increases.

Silhouette score evaluates embedding quality by measuring clustering coherence. For a given embedding i with cluster label a, the silhouette score is:

$$s(i) = \frac{b(i) - a(i)}{\max(a(i), b(i))}$$

where $a(i)$ is the mean distance to other points in the same cluster and $b(i)$ is the mean distance to points in the nearest neighboring cluster. Scores range from -1 to 1, where higher values indicate better-defined clusters. We use this

Algorithm 1 Algorithm for computing biologically informed sparse attention, from input tensors through block-wise operations to final output.

Entries: Queries, Keys, and Values: $Q, K, V \in \mathbb{R}^{n \times d}$
Protein interactions $P_I \in \mathbb{R}^{i \times 2}$, Protein sizes $P_S \in \mathbb{R}^N$

$Q, K, V \leftarrow$ reshape_tensor_padding(Q, K, V, P_S, B) ▷ Split genome into proteins, apply padding if necessary, then into B-size blocks: $X(bs, n_b, B, d) \leftarrow X(bs, n, d)$

$Q, K, V \leftarrow$ transpose_for_scores(Q, K, V, n_h, h_s) ▷ Transpose tensors for attention computation: $X(bs, n_h, h_s, n_b, B) \leftarrow X(bs, n_b, B, d)$

$BI \leftarrow$ chunk_proteins_padding(PI, PS) ▷ Transform protein interactions into block interactions

$Q, K \leftarrow$ rotary_embeddings(Q, K) ▷ Apply rotary positional embeddings

$Att \leftarrow$ sparse_attention_matrix(Q, K, BI) ▷ Compute attention scores and construct BSR tensor
$Att \leftarrow$ bsr_softmax(Att)
$Att \leftarrow$ bsr_dropout$(Att,$ dropout$)$
$V \leftarrow$ bsr_dense_mm(Att, V)

$V \leftarrow$ revert_padding(V) ▷ Reverse padding and reshape into final dimensions : $V(bs, n, d) \leftarrow V(bs, n_b, B, d)$

return V

metric to assess whether embeddings group biologically related sequences (e.g., by sequence similarity or taxonomic classification).

3.7 Evaluating Global Performance & Protein Embeddings Quality

We evaluated our biologically informed sparse attention models (LV-3C and LV-5B) against ESM-2 pre-trained (ESM-2-pt) and ALiBi fine-tuned variants (ESM-2-ALiBi) using perplexity and clustering silhouette scores. Perplexity assessed token prediction accuracy over extended contexts, while the silhouette scores evaluated the quality and information content of protein sequence embeddings. We assessed model performance using viral genome fragments ranging from 500 to 20,000 aa. Although our models support sequences up to 61,000 aa, we limited evaluations to 20,000 aa to accommodate the computational constraints (time and memory) associated with full attention when running on a single GPU (NVIDIA A6000 with 48GB of RAM).

Sequence-based clustering reference. To evaluate whether model embeddings preserve sequence relationships, we clustered proteins using MMseqs2 at 80% sequence identity. These clusters serve as reference labels for computing silhouette scores: high silhouette scores indicate that proteins similar in sequence are also proximal in embedding space. This provides a baseline assessment of embedding quality independent of functional or taxonomic annotations.

3.8 Evaluating Genome Embeddings Quality

We assessed the quality of genome-scale embeddings produced by our models using taxonomic clustering and species classification. To further evaluate embedding utility, we trained a single-layer classifier to identify species from genome embeddings (details provided in Supplementary Material S3).

3.9 Evaluation of Embedding Quality for Classification

We evaluated the models' contextual embeddings by assessing their performance on species clustering and classification tasks ~~(see also Supplementary Material S4)~~(see also Supplementary Material S3; Pfam/domain-family validation is described separately in Supplementary Material S4), as well as by analyzing the similarity distributions obtained from comparing pairs of embeddings.

- **Evaluation of taxonomic information preservation:** To evaluate how effectively different models preserve taxonomic information, we analyzed clustering patterns of protein embeddings derived from 100 distinct viral species.

- **Analysis of species clustering efficiency:** Next, we trained a single-layer taxonomic classifier designed to predict the species identity of individual proteins directly from their embeddings. To systematically assess classification performance, we varied the number of species classes from 5 up to 100 species. We measured classifier performance using F1-score averaged over a 5-fold cross-validation scheme.
- **Analysis of embedding variance and distribution:** To further characterize the differences among embedding spaces produced by various models, we randomly selected 5,000 proteins from over 1,000 viral genomes and computed pairwise Euclidean distances between their embeddings. We then compared the resulting distributions from embeddings generated by ESM-2 variants and our two proposed models, LV-5B and LV-3C.

3.10 Evaluating Across-Model Attention

~~To assess our models' capacities to capture PPIs within viral genomes, we analyzed~~ The following two sections evaluate the attention patterns of the trained LV-3C and LV-5B models, as distinct from the Stage 1 PPI inference used to build the sparse-attention priors: they assess whether the trained models—having been guided by these priors during training—retain and recapitulate biologically meaningful PPI signal in their final attention. To this end, we analyzed attention patterns across the entire network architecture. Attention scores from all heads in each layer were extracted to examine each model's ability to detect and represent interactions spanning various distances within the genome sequence.

We categorized attention interactions into three distinct proximity classes:

- **Short-range interactions**: Attention between amino acids positioned within 100 residues of each other, typically representing intra-protein or intra-domain relationships.
- **Medium-range interactions**: Attention between amino acids separated by 100–500 residues, generally capturing interactions between domains or adjacent proteins.
- **Long-range interactions**: Attention between amino acids separated by more than 500 residues, predominantly representing inter-protein interactions between non-adjacent proteins.

For each category, we calculated the mean attention score across multiple viral genomes, enabling comprehensive analysis of the model's attention distribution patterns.

3.11 Validation Against Experimentally Curated Interactions

To validate the biological relevance of model-derived attention patterns, we benchmarked ~~predicted protein-protein interactions~~ attention-derived protein-pair scores, interpreted as putative interaction links, against experimentally validated interactions documented in the STRING database [43]. Attention scores were extracted from all heads and layers, and protein pairs were ranked by their aggregate long-range attention score following [38]. Validation encompassed 146 annotated interactions across five distinct viral genomes. STRING interactions with confidence ≥ 0.500 served as positive examples; negative examples consisted of all other protein pairs from the same genomes not reported as interacting in STRING. We note that this negative set likely contains some true interactions not yet experimentally characterized, making our reported metrics conservative estimates.

Data and Code Availability

All source code, long-context inference scripts, and pair-ranking utilities are available at `https://github.com/Hawaii-Bioinformatics/ViralEmbed`. Example inputs are provided in `large_context/scripts/data/`, including a test viral proteome FASTA (`LR699048.fa`) and a matching protein-labels text file (`LR699048.txt`) used for pair-ranking demonstrations.

Attention tensors are generated locally using the provided inference script (see `large_context/scripts/large_prot_encoding/inference.py` instructions in the scripts README).

A worked "realistic use case" notebook is included in `large_context/scripts/data/realistic_use_case/use_case.ipynb`. To reproduce the results, download the NCBI Virus snapshot used in this study (14,436 complete genomes; retrieved 2024–06) and regenerate the attention tensors and downstream analyses using the provided scripts.

4 Results

4.1 ~~Optimizing~~ Developing the PPI Inference Model (Stage 1)

Before training our genome-scale models LV-3C and LV-5B, we first developed ESM-2-PPI, a separate auxiliary model used exclusively to derive the protein–protein interaction priors that guide the sparse attention mechanism. ESM-2-PPI extends pretrained ESM-2 (650M) to a 13,000 aa context window via LoRA fine-tuning, sparse attention, and ALiBi positional extrapolation. Because the choice of positional encoding and sparse-attention pattern directly affects the reliability of the attention scores subsequently used as priors, we evaluated two positional encoding strategies (RoPE, ALiBi) (Supplementary Figure S2) and three sparse attention mechanisms (Longformer, Shifted Sparse Attention, BigBird) (Supplementary Table S1) on held-out viral genome fragments ranging from 500 to 13,000 aa, requiring both stable perplexity across extended sequence lengths and embeddings that preserve biological relationships.~~, we optimized a PPI inference model (ESM-2-PPI) to reliably extract attention patterns from multi-protein fragments up to 13,000 aa. This model must exhibit stable perplexity across extended sequence lengths and produce embeddings that preserve biological relationships. We evaluated two positional encoding strategies (RoPE, ALiBi) (Supplementary Figure) and three sparse attention mechanisms (Longformer, Shifted Sparse Attention, BigBird) (Supplementary Table).~~ ALiBi with Longformer attention achieved the best balance of perplexity stability (1.99 across all tested lengths), clustering quality (silhouette score: 0.88), and computational efficiency.

To validate that ESM-2-PPI captures biologically meaningful interactions, we analyzed attention patterns among Family A DNA polymerase (PolA), ribonucleotide reductase (RNR), and helicase (HEL)—proteins with well-characterized co-evolutionary relationships [39, 40, 41]. These known interacting pairs consistently ranked among the top 50 non-adjacent protein pairs across 83 viral genomes (Supplementary Figures S3, S4), supporting our selection of $k = 50$ as the interaction threshold.

This validation serves only to calibrate the PPI selection threshold for ESM-2-PPI and the PolA/RNR/HEL interactions were not used during training of LV-3C or LV-5B, which learn from PPIs inferred independently across all genomes in the training set.

4.2 Evaluating Global Performance & Embeddings Quality

This section evaluates our two genome-scale protein language models LV-3C (317M parameters, trained from scratch on sequences up to 61,000 aa) and LV-5B (756M parameters, initialized from ESM-2 weights and trained on sequences up to 48,000 aa) against three ESM-2 baselines (ESM-2-pt, ESM-2-RoPE, ESM-2-ALiBi). Both LV models share the same biologically informed sparse attention mechanism and differ only in scale and initialization, allowing us to disentangle the contribution of context length from that of pretrained knowledge.

The LV-3C and LV-5B models demonstrated significantly lower perplexity (uncertainty when predicting masked tokens) compared to ESM-2 variants on sequences longer than 3,000 aa (Figure 4a), maintaining this advantage consistently across all tested lengths. In contrast, both the pre-trained ESM-2-pt (gray) and its RoPE-fine-tuned variant ESM-2-RoPE (red) experienced significant perplexity inflation when evaluated beyond their original training context windows. Fine-tuned version of ESM-2-ALiBi (orange) maintains near-constant perplexity with sequence length, but higher than our models.

For clustering quality analysis, we clustered protein sequences and fragments using MMseqs2 [44] at an 80% similarity threshold and computed silhouette scores to quantify embedding coherence. Our results revealed clear differences in clustering performance among the tested models (Figure 4b).

Both LV models provided superior embedding coherence and better discrimination of protein sequences compared to baseline approaches. LV-3C (light blue) exhibited robust clustering quality, particularly for longer sequences (silhouette score: 0.83), while LV-5B (dark blue) achieved performance comparable to ESM-2-pt and ESM-2-RoPE for shorter sequences and maintained high effectiveness at extended lengths (silhouette score: 0.67). ESM-2-pt (gray) excelled only within its original training range (1,024 aa), with embedding quality markedly declining for longer, multi-protein sequences. While ESM-2-ALiBi (orange) extended effective clustering performance up to approximately 4,000 aa, embedding quality still deteriorated as sequence lengths increased, despite the positional encoding extrapolation strategy.

4.3 Evaluating Genome Embeddings Quality

The quality of the genomic embeddings was assessed by downstream performance in a clustering and a classification task. We employ two complementary evaluations: silhouette scores assess embedding coherence, whether proteins from the same taxonomic species cluster together in latent space, while classification accuracy assesses whether embeddings encode sufficient discriminative information for species prediction. These metrics capture related but distinct properties:

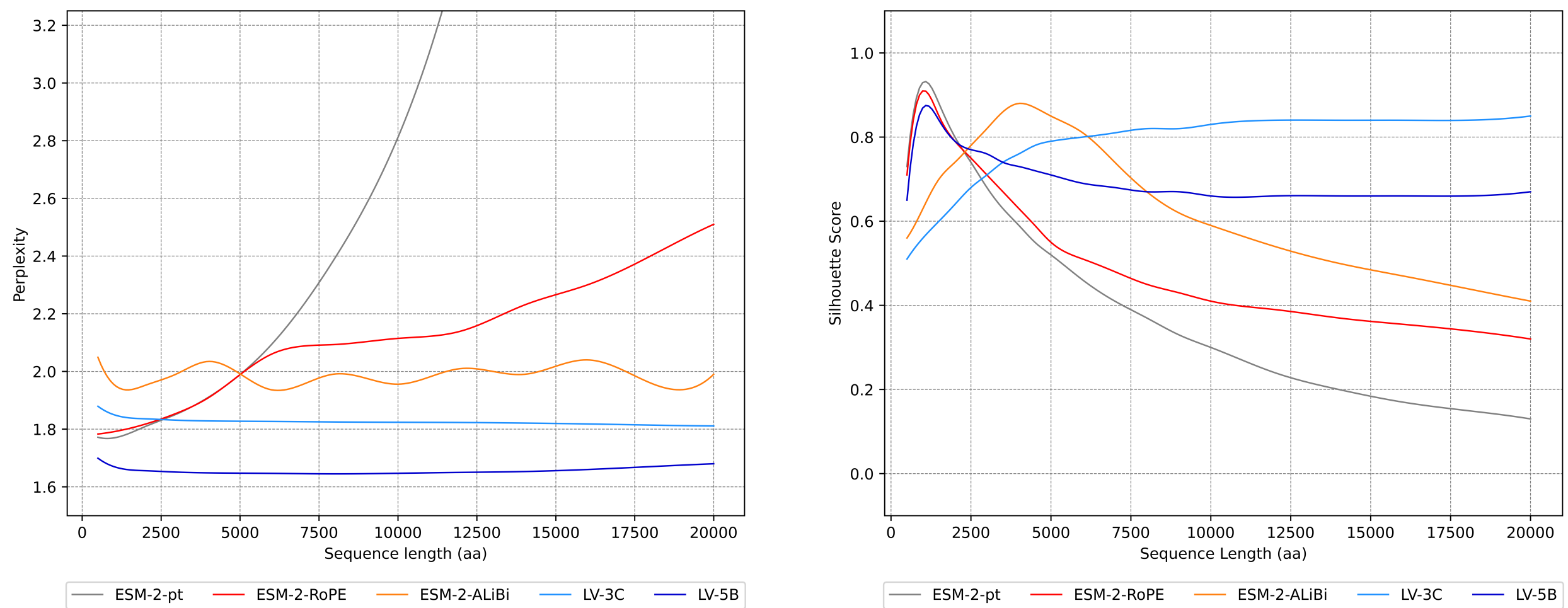

(a) Perplexity

(b) Silhouette Score

Figure 4: **Global performances of our models compared to ESM-2 variant baselines. (a)** Perplexity evaluation of ESM-2-pt, ESM-2-RoPE, ESM-2-ALiBi, and our biologically informed sparse attention models LV-3C & LV-5B. **(b)** Clustering ability evaluation using silhouette score on fragment embeddings, using sequence similarity as reference with MMSeqs2 80% amino acid identity clustering. These smoothed curves are obtained from perplexity and silhouette score measurements for sequences ranging from 500 to 20,000 amino acids.

coherent embeddings facilitate classification, but similar classification performance can arise from different embedding geometries. Silhouette scores (Figure 5a) indicated strong taxonomic discrimination across all models, with the LV-5B variant consistently outperforming others.

The performance of the species classification of genomes evaluated by F1-scores (Figure 5b) revealed a notable trend: as the number of species increased—and consequently, task complexity—embeddings generated using biologically informed sparse attention demonstrated superior classification accuracy relative to genome embeddings from fine-tuned ESM-2-ALiBi. This performance improvement at higher taxonomic resolution suggests our embeddings effectively capture nuanced genomic features, enhancing discriminative power at larger phylogenetic scales. These properties hold promising implications for phylogenetic and metagenomic applications.

4.4 Evaluation of Protein Embedding Quality for Classification

4.4.1 Analysis of Embedding Variance and Distribution

The quality of protein embeddings generated by ESM-2-ALiBi and proposed models LV-3C and LV-5B were assessed based on embedding variance and distribution of the pairwise Euclidean distances between 5,000 randomly selected protein from over 1,000 viral genomes. The analysis (Figure 6) revealed distinct variability patterns among these embedding spaces. Embeddings produced by ESM-2-ALiBi demonstrated the narrowest distribution, with the lowest interquartile range (1.16) and a relatively limited embedding space extent (range: 9.43). In contrast, embeddings from model LV-5B showed greater variability, with a broader interquartile range (1.58) and a larger embedding space (range: 15.69). Model LV-3C exhibited the most dispersed embeddings, spanning the widest embedding space (range: 16.18) and displaying significantly higher average pairwise Euclidean distances. These results indicate that incorporating genomic context into LV-3C substantially diversifies embedding representations, likely capturing biologically meaningful, genome-specific information.

Further analysis of the Euclidean distance across protein embeddings shows that context provides a stronger correlation with taxonomic distance (Figures 6, 7). Specifically, embeddings from proteins within the same species exhibit high similarity, characterized by low Euclidean distance. As the taxonomic distance between proteins increases, embedding similarity correspondingly decreases, highlighting the models' ability to capture biologically meaningful divergence across broader taxonomic categories.

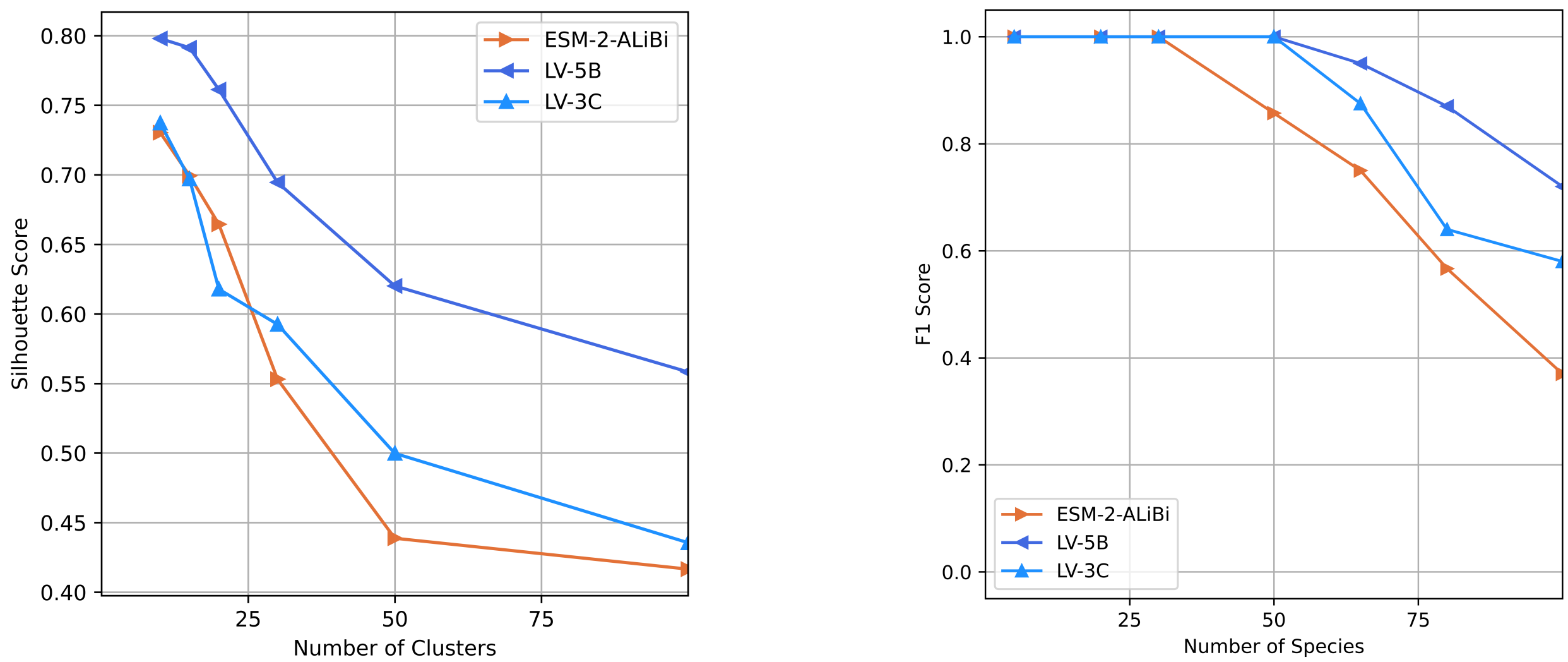

(a) Silhouette Score

(b) Classification

Figure 5: **Evaluation of full genome embeddings. (a)** Silhouette scores comparing genome embeddings generated by LV-3C, LV-5B, and ESM-2-ALiBi, using taxonomic species as cluster labels. **(b)** F1-scores for a single-layer classifier predicting species identity ~~based on~~ from genome embeddings ~~from LV-3C, LV-5B, and ESM-2-ALiBi~~ (LV-3C, LV-5B, ESM-2-ALiBi). The x-axis varies the number of viral species used as classification targets, from 5 to 100; larger values denote finer-grained taxonomic resolution.

4.4.2 Evaluation of Taxonomic Information Preservation

Given that protein embeddings inherently cluster primarily by protein type rather than species, silhouette scores based on taxonomy labels across all models were generally low (slightly negative). Despite this challenge, embeddings generated by the LV-3C model exhibited better species-specific clustering relative to those produced by ESM-2-pt and LV-5B (Figure 7a, Pfam-specific clustering in Supplementary Material S4 and Supplementary Figure S5). For comparison, we included embeddings from the Protein Set Transformer (PST), a recent genome-level protein model, as an additional baseline. We note that PST's public release (v2.1.0) does not support genome-level embeddings, and its graph neural network architecture does not produce attention scores comparable to our transformer-based approach; PST is therefore included only in protein embedding evaluations where outputs are directly comparable. PST achieved the lowest silhouette scores, indicating limited capability in capturing viral species-level taxonomic relationships.

4.4.3 Analysis of Species Clustering Efficiency

As illustrated in Figure 7b, classification performance was the lowest when utilizing context-independent embeddings generated by the pre-trained ESM-2-pt. PST embeddings improved classification outcomes slightly, affirming that PST does capture genome-level context to some degree. However, LV-3C and LV-5B embeddings enriched with extensive genomic context further enhanced classifier accuracy across all evaluated class sizes. Among the tested models, LV-3C consistently achieved superior performance, reflected in the highest F1-scores for every class configuration.

4.5 Evaluating Across-Model Attention

Our proposed models were evaluated based on mean attention scores in short-range (within 100 aa, representing intra-protein or intra-domain relationships), mid-range (within 100-500 aa, representing inter-domain or adjacent protein relationships), and long-range ($>$ 500 aa apart, representing inter-protein interactions between non-adjacent proteins) interactions. The distribution of normalized attention scores across the three ranges provides insight into the model's capacity to capture biologically relevant interactions at different genomic scales. High variance in the attention score distribution indicates preferential focus on specific interaction ranges, while more uniform distributions suggest balanced attention across all distance categories. This analysis is particularly valuable for assessing whether the

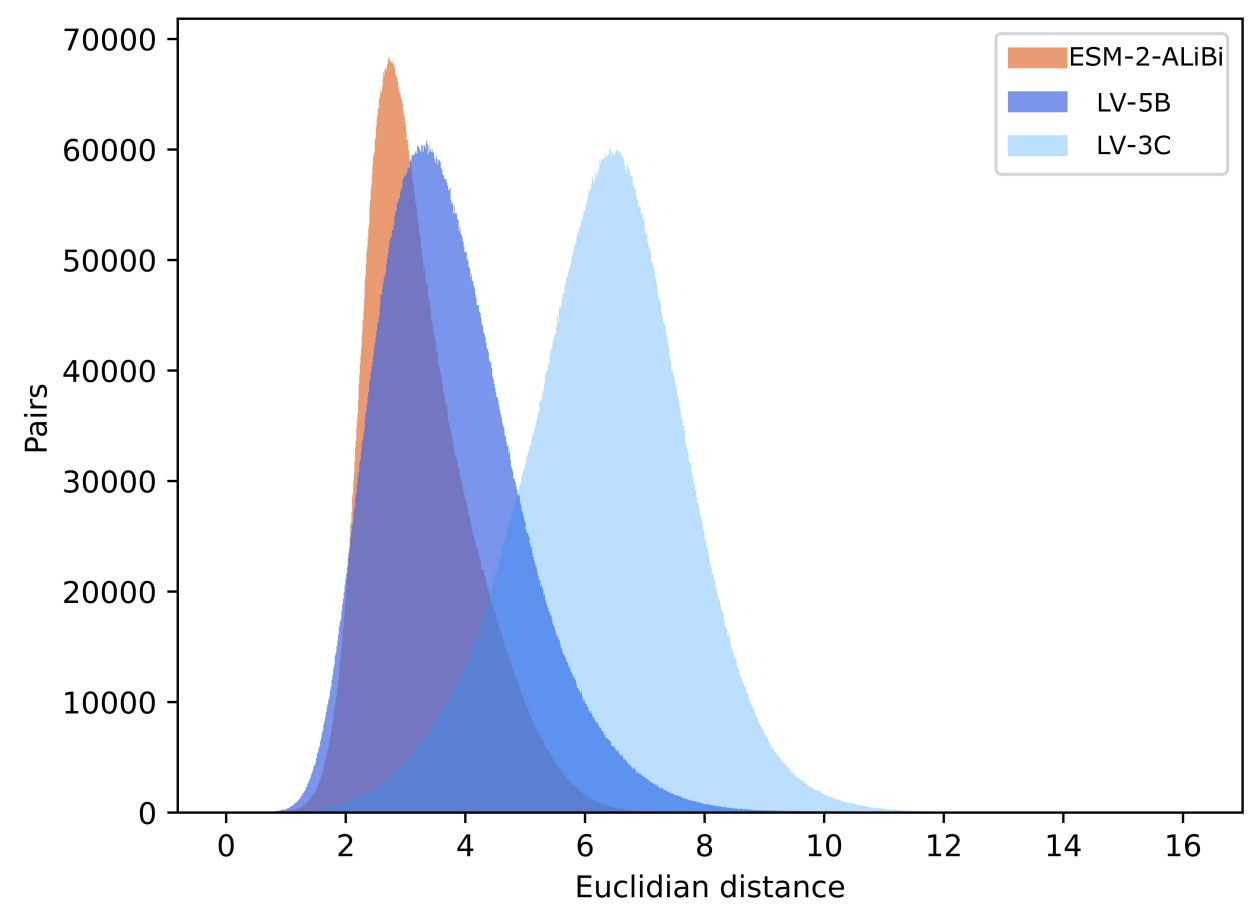

Figure 6: **Protein embeddings pairwise Euclidean distance.** Each curve summarizes ≈12.5 million pair comparisons: lower distances (left tail) indicate within-species similarity, while greater distances reflect increasing taxonomic separation. ESM-2-ALiBi yields the most compact manifold, LV-5B broadens the range, and LV-3C further widens and right-shifts the distribution, demonstrating its larger dynamic range for distinguishing distantly related proteins without sacrificing tight clustering of closely related sequences.

model effectively captures the long-range dependencies that conventional protein language models typically miss due to context limitations.

The attention profiles (short-, medium-, and long-distance) differ substantially among the four models evaluated (Figure 8a). The pre-trained ESM-2-pt model exhibits a highly localized attention mechanism, concentrating almost exclusively on short-range interactions (100%), typically within individual proteins or immediate neighboring residues. This pattern aligns with ESM-2's training paradigm, which focused on single-protein contexts and thus developed representations primarily capturing local residue relationships.

In contrast, the ESM-2-ALiBi fine-tuned model, which employs a sliding-window (LongFormer) attention approach, demonstrates a modest expansion in attention scope. While it identifies a small fraction of medium-distance interactions (3.12%), it still predominantly focuses on short-distance interactions (96.88%). These findings suggest that although the sliding-window methodology enables detection of certain medium-range relationships, the model largely remains constrained to interactions between proximal amino acids.

Our models, LV-3C and LV-5B, exhibit markedly different attention distributions. Both models display balanced average attention scores across all distance categories (short, medium, and long), indicating a comprehensive approach to protein sequence modeling that equally integrates local and global contextual information. However, important distinctions between the two models emerge:

- Model LV-3C most frequently attributes maximal attention to long-distance interactions. This behavior highlights its enhanced capability to capture meaningful relationships between amino acids widely separated in sequence space, potentially spanning different proteins within the viral genome.
- Model LV-5B, while maintaining generally balanced average scores, frequently assigns highest attention importance to both short- and long-distance interactions. This indicates a flexible modeling strategy capable of emphasizing both local structural information and global protein–protein relationships as needed. Notably, LV-5B's attention distribution retains traces of its ESM-2-pt initialization, suggesting it effectively combines characteristics of the pre-trained model with adaptations necessary for the expanded genomic context.

To further investigate inter-protein interactions, we calculated attention scores and identified attention heads specifically enriched for long-range information, following the methodology described in Section 3.11.

In a binary classification framework, using a STRING interaction confidence threshold of 0.500, our LV-3C model achieved an average F1 score of 0.74 and accuracy of 0.88. The classifier demonstrated an Area Under the Curve (AUC) of 0.66 (Figure 9, with a bootstrapped 95% confidence interval of [0.56, 0.75]), indicating statistically significant predictive performance. The difference between F1 (0.74) and AUC (0.66) reflects that F1 is measured at an optimized

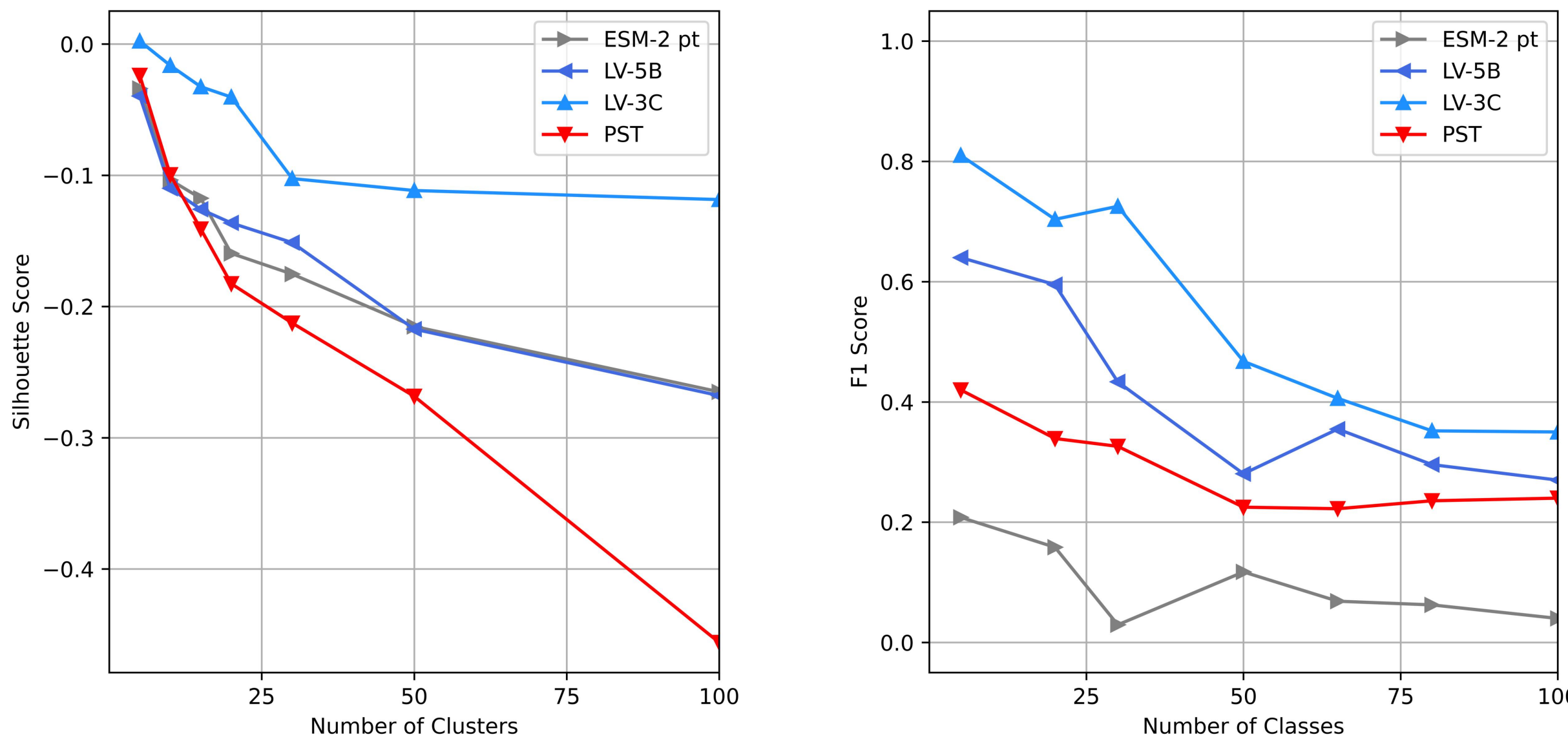

(a) Silhouette Score

(b) Classification

Figure 7: **Evaluation of contextualized protein embeddings. (a)** Silhouette scores comparing protein embeddings generated by LV-3C, LV-5B, Protein Set Transformer (PST), and ESM-2-pt, using taxonomic species as cluster labels. **(b)** F1-scores for a single-layer classifier predicting species identity ~~based on genome~~ from protein embeddings ~~from LV-3C, LV-5B, PST, and ESM-2-pt~~ (LV-3C, LV-5B, PST, ESM-2-pt). The x-axis varies the number of viral species used as classification targets, from 5 (a coarse 5-way task) to 100 (a fine-grained 100-way task); larger values denote harder, more taxonomically resolved classification.

decision threshold, while AUC captures ranking performance across all thresholds—a more challenging metric given the inherent noise in attention scores and the known incompleteness of viral protein interaction databases. These results ~~demonstrate substantial~~ show significant concordance between our attention-based ~~predictions~~ interaction scores and experimentally validated interactions in the STRING database~~. The robust F1 score and high accuracy underscore our method's capacity to identify biologically meaningful protein interactions~~ , indicating that the method enriches for putative protein interactions within viral genomes. Furthermore, attention-derived scores showed significant positive correlation with STRING confidence scores (Spearman $\rho = 0.33$, $p < 0.001$), providing additional evidence for the biological relevance of the learned attention patterns.

5 Discussion

We note that attention-derived inter-protein links should be interpreted as computational priors rather than direct evidence of physical PPIs. For this reason, our evaluation strategy focused on language-model metrics, taxonomy/Pfam-aligned downstream tasks, and concordance with independent interaction resources (STRING), rather than claiming direct prediction of physical interactions.

Our findings demonstrate that extending transformer-based language models to encompass entire viral proteomes yields substantial improvements in both local amino acid prediction and broader protein function inference. By designing a biologically informed and content-aware sparse attention mechanism guided by inter-protein co-evolutionary signals, we have addressed the long-standing challenge of modeling densely packed viral genomes while maintaining computational tractability.

A central insight from our study is that higher-order genomic context—the collective presence and arrangement of multiple proteins—significantly enhances protein representation accuracy beyond what can be achieved by analyzing protein sequences in isolation [3], or using context length extension [15, 16] or context-agnostic sparse attention [25, 26, 27]. As the results show, conventional protein language models trained on individual proteins (e.g., ESM-2) focus on local sequence features and exhibit marked performance degradation when context extends beyond their trained window. In contrast, our biologically informed sparse attention approach captures both local relationships and long-range dependencies across entire viral genomes. This more comprehensive perspective reveals functional couplings between

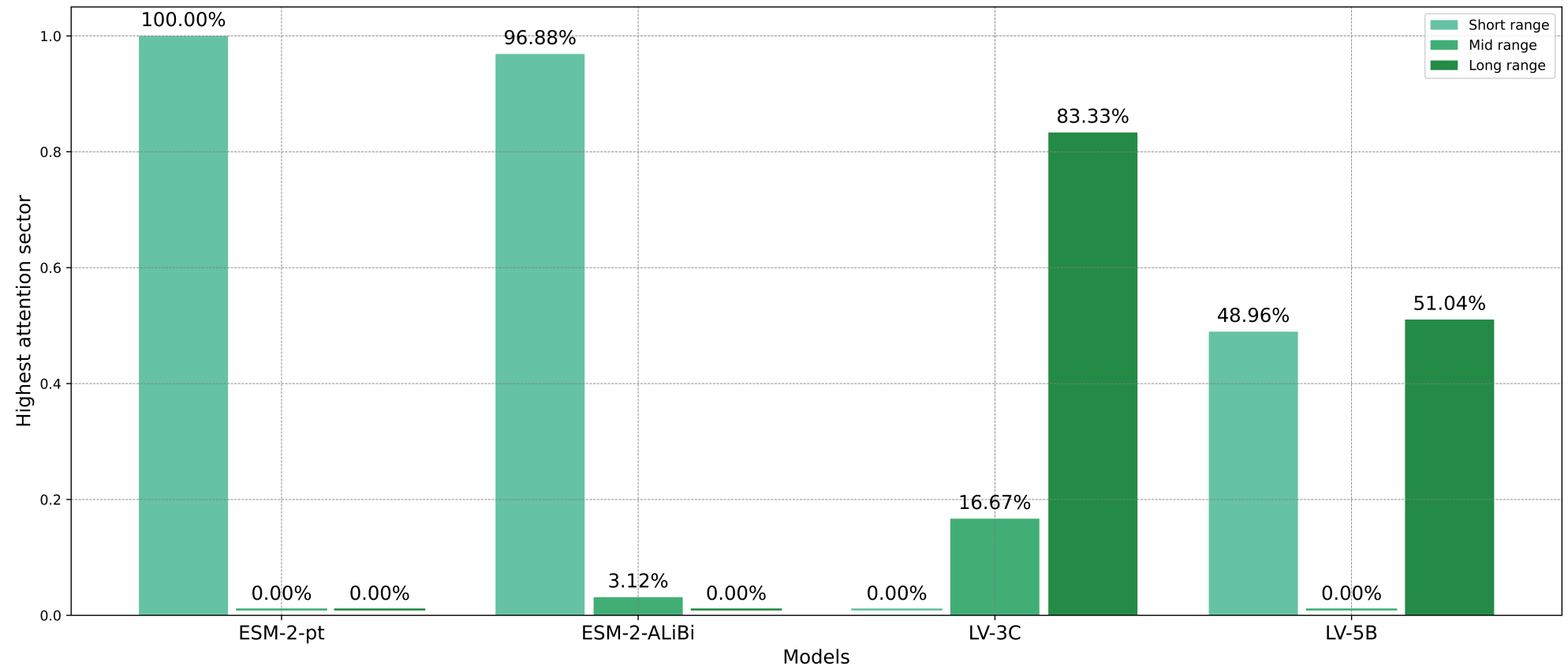

(a)

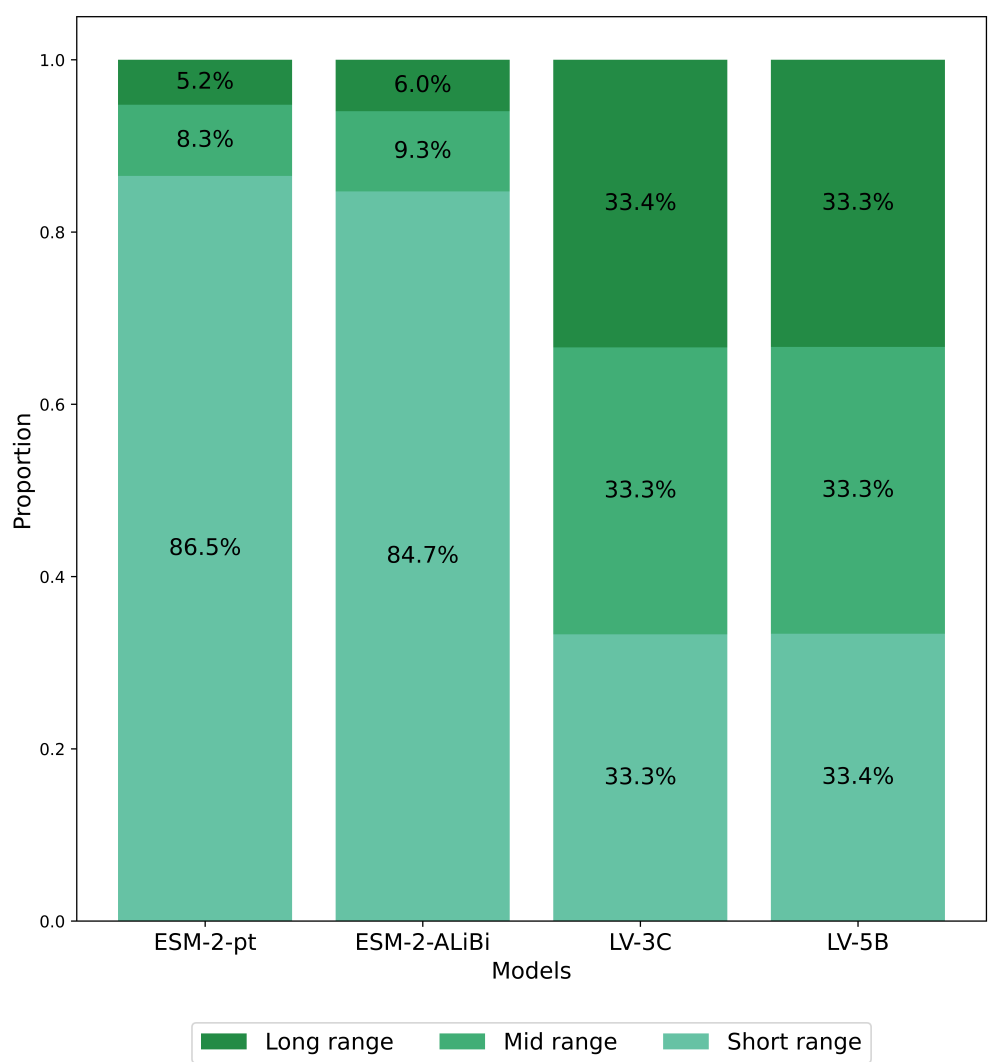

(b)

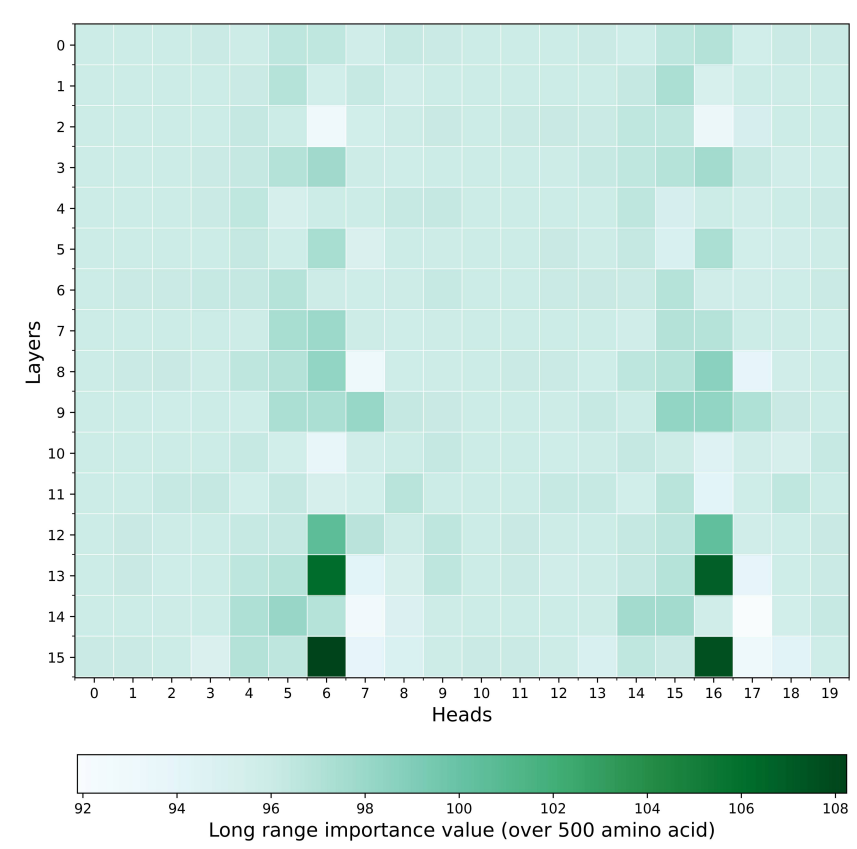

(c)

Figure 8: **Attention analysis of our models.** **(a)** Prevalence of dominant short-range (≤100 aa), medium-range (100–500 aa), and long-range (>500 aa) sectors across viral genomes. The chart represents 1,000 randomly selected viral genomes (≤13,000 aa in length), showing the proportion of genomes in which each interaction range category exhibited the highest mean attention score. **(b)** Distribution of normalized attention scores across interaction distance categories in viral proteomes. Stacked bar charts represent the distribution of attention scores for each interaction range category across 1,000 viral genomes. **(c)** Fine-grained analysis of long-range attention patterns in the LV-3C model. Darker (green) blocks indicate attention heads with a predominant long range attention. The visualization demonstrates how attention mechanisms capture biologically significant inter-protein interactions spanning substantial genomic distances within viral proteomes. Distribution variance indicates the relative focus of attention heads on specific interaction distances.

non-consecutive proteins that would otherwise remain undetected [39, 40, 41]. To confirm that this advantage arises from the inference step rather than from simply adding long-range links, we trained otherwise identical models given inferred, random, distance-matched, or no long-range links. Only the inferred links lowered held-out perplexity, and the improvement concentrated on the protein pairs the inference model scored most highly (Supplementary Section S6).

The attention patterns learned by our models ~~demonstrate remarkable alignment~~ show statistically significant concordance with experimentally validated protein–protein interactions, benchmarked against the STRING database [43]. ~~This suggests that attention mechanisms in our models effectively capture biologically meaningful co-evolutionary relationships, providing a computational approach to predict functional protein interactions that complements signal and may help enrich for putative functional interactions, complementing rather than replacing experimental techniques.~~ The balanced distribution of attention across short, medium, and long-range interactions in our LV-3C and LV-5B models, in

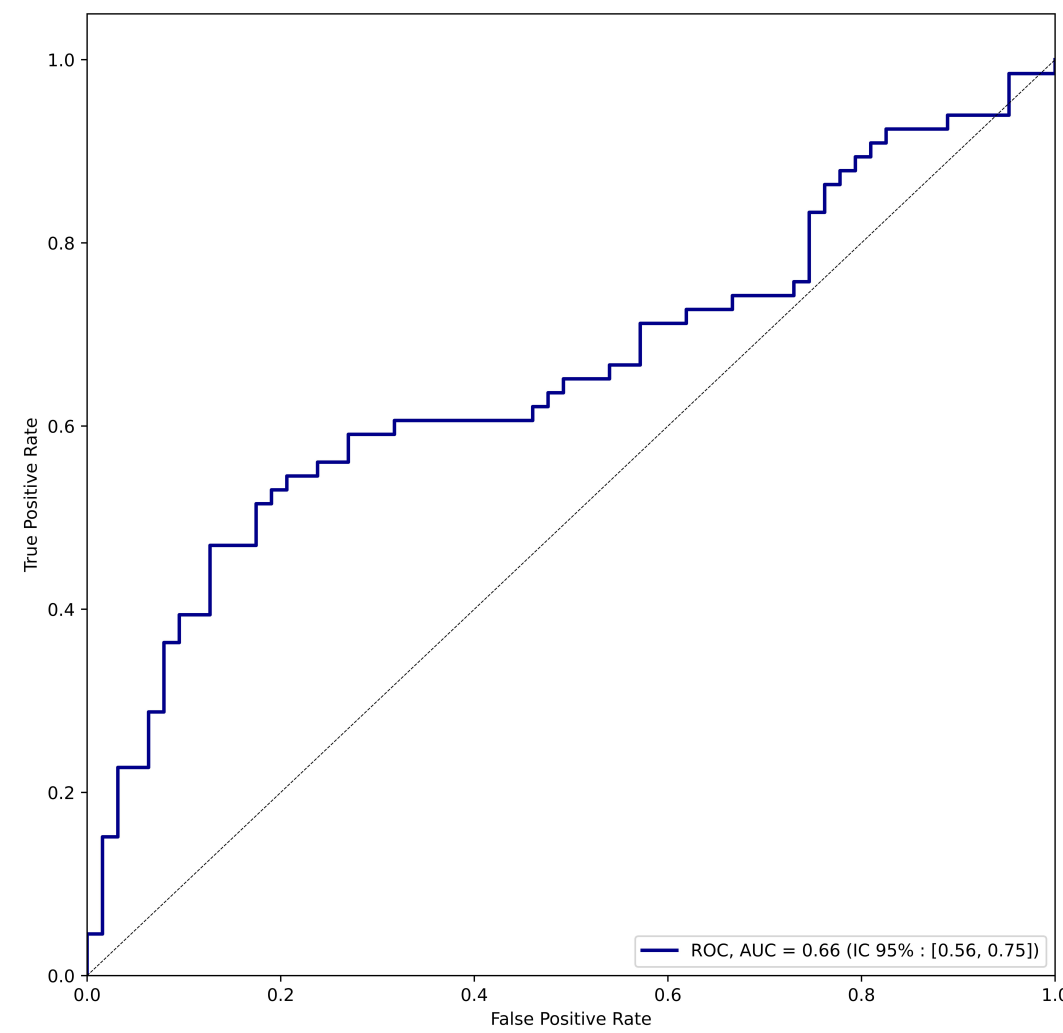

Figure 9: **Receiver Operating Characteristic (ROC) curve of the binary classification task.** ROC curve for ~~binary classification of protein–protein interactions based on attention scores~~distinguishing STRING-supported protein pairs from protein pairs inferred using attention-derived scores. The curve shows LV-3C ability to distinguish experimentally validated interactions (STRING confidence ≥ 0.500) from non-interacting protein pairs across five viral genomes (146 positive interactions). The AUC of 0.66, with a 95% bootstrapped confidence interval of [0.56, 0.75], indicates statistically significant predictive performance.

contrast to the predominantly local focus of the ESM-2 baselines, further reinforces the biological relevance of our approach.

The significant divergence in embedding spaces between context-independent (other models) and the context-aware LV-3C and LV-5B models, as evidenced by the distinct distributions of pairwise Euclidean distances between protein embeddings, underscores the fundamental difference in information captured by these approaches. The wider embedding space of our LV-3C model, with its greater interquartile range and broader distribution of distances, suggests a richer representation that incorporates both sequence-specific and context-dependent information.

To assess generalization across viral diversity, we evaluated model performance specifically on RNA viruses, which represent the most abundant and challenging subgroup in our dataset (Supplementary Material S5). Despite their higher mutation rates and structural complexity, our models consistently outperformed baselines (Supplementary Figure S6), suggesting robust performance across viral groups.

To assess sensitivity to our 90% deduplication threshold, we additionally clustered at 65% identity, yielding 246,737 sequences—indicating that 71% of sequences retained at 90% clustering have no other sequence sharing >65% identity. This analysis suggests that the majority of our evaluation data consists of substantially divergent sequences, not near-duplicates. The impact of sequence similarity also varies by task: perplexity is most sensitive to train/test similarity, while taxonomy and species classification are less affected since these tasks require learning species-level patterns, making within-species similarity a desired signal rather than a confounder. Critically, all models were evaluated under identical conditions, ensuring valid relative comparisons.

To validate that our embeddings capture functional information beyond taxonomy, we evaluated performance using Pfam protein domain annotations (Supplementary Figure S5). While silhouette scores are low, reflecting that genomic context, not domain identity, is the dominant organizing principle—classification performance remains strong, with LV-3C and LV-5B outperforming baselines. This confirms that domain information is encoded within our embeddings alongside the contextual signals that distinguish proteins with identical domains in different genomic environments.

Despite these advances, computational overhead remains a practical consideration. Our selection of a maximum of 50 protein–protein interactions for the sparse attention mechanism strikes an effective balance for viral datasets while maintaining computational efficiency. This parameter can be readily increased if GPU resources are available. We intentionally constrained our implementation to run on a single GPU, as we believe this represents a widely accessible computational environment for most research groups. While we utilized an NVIDIA A6000 for this study, the rapid advancement of more affordable GPUs (sub-$5,000) with comparable memory capacities makes

our approach increasingly accessible. For processing significantly larger genomes—particularly from eukaryotic organisms—additional optimizations may be beneficial. Additionally, our semi-supervised approach to inferring putative interactions could benefit from larger ground-truth databases or complementary evidence from proteomic or structural studies to further refine the biological relevance of the learned attention patterns.

Although our training data includes human pathogen sequences, our models pose substantially lower biosecurity risk compared to generative genomic models such as Evo [33]. Both LV-3C and LV-5B are designed to produce embeddings for downstream analysis rather than to generate novel sequences. The masked language modeling objective predicts individual positions given surrounding context, rather than generating complete sequences autoregressively. These distinctions substantially limit the utility of our models for generating functional novel proteins or genomes.

Looking forward, our framework offers considerable promise for extension to larger viral genomes, bacteriophages, and potentially even eukaryotic genomes with appropriate scaling. The methodology could be particularly valuable for analyzing metagenomic data, where contextual information might help resolve ambiguities in protein function assignment. Furthermore, the correlation between embedding similarity and taxonomic distance suggests potential applications in phylogenetic analysis and evolutionary studies.

6 Conclusion

Our study proposes a novel framework for viral protein language modeling by extending transformer architectures to process entire viral genomes. By integrating a biologically informed sparse attention mechanism, our approach efficiently captures putative long-range inter-protein interactions that are missed by conventional single-protein models. This design not only reduces perplexity across extended sequences (up to 61,000 aa) but also generates context-aware embeddings that accurately recapitulate known co-evolutionary relationships—for instance, among PolA, RNR, and helicase.

Our results demonstrate that large-scale learning improves embedding quality. The enhanced clustering of protein and genome embeddings, along with robust taxonomic classification, underscores the broader applicability of our method to phylogenetic analysis and metagenomic studies. Our work provides a solid foundation for working with viral genomes and paves the way for more refined analyses of protein–protein interactions, ultimately deepening our understanding of viral biology and evolution. Looking ahead, our framework offers considerable promise for extension to larger genomes, and potentially even eukaryotic genomes. By refining the biological specificity of sparse attention patterns and incorporating additional layers of experimental validation, our approach can continue to evolve toward more precise and comprehensive genomic analyses. Ultimately, this work not only deepens our understanding of viral genome organization and evolution but also provides a scalable framework for protein modeling at genomic scales, bridging the gap between computational biology and experimental validation.

7 Acknowledgments

This work is supported by the National Science Foundation grants 1736030 and 2025567. Support from the University of Delaware Center for Bioinformatics and Computational Biology (CBCB) Core Facility, the University of Delaware Sequencing and Genotyping Center, and use of the BIOMIX compute cluster was made possible through funding from Delaware INBRE (NIGMS P20GM103446, NIH S10OD028725), and the State of Delaware.

References

[1] Xueliang Liu. Deep Recurrent Neural Network for Protein Function Prediction from Sequence. January 2017. arXiv:1701.08318 [q-bio].

[2] Abel Chandra, Laura Tünnermann, Tommy Löfstedt, and Regina Gratz. Transformer-based deep learning for predicting protein properties in the life sciences. *eLife*, 12:e82819, 2023.

[3] Zeming Lin, Halil Akin, Roshan Rao, Brian Hie, Zhongkai Zhu, Wenting Lu, Allan dos Santos Costa, Maryam Fazel-Zarandi, Tom Sercu, Sal Candido, and Alexander Rives. Language models of protein sequences at the scale of evolution enable accurate structure prediction. July 2022. Pages: 2022.07.20.500902 Section: New Results.

[4] Ahmed Elnaggar, Michael Heinzinger, Christian Dallago, Ghalia Rihawi, Yu Wang, Llion Jones, Tom Gibbs, Tamas Feher, Christoph Angerer, Martin Steinegger, Debsindhu Bhowmik, and Burkhard Rost. ProtTrans: Towards Cracking the Language of Life's Code Through Self-Supervised Deep Learning and High Performance Computing, May 2021. arXiv:2007.06225 [cs].

[5] Erik Nijkamp, Jeffrey Ruffolo, Eli N. Weinstein, Nikhil Naik, and Ali Madani. ProGen2: Exploring the Boundaries of Protein Language Models, June 2022. arXiv:2206.13517 [cs].

[6] Claire D. McWhite and Mona Singh. Vector-clustering Multiple Sequence Alignment: Aligning into the twilight zone of protein sequence similarity with protein language models, October 2022.

[7] Benjamin Giovanni Iovino and Yuzhen Ye. Protein embedding based alignment. *BMC Bioinformatics*, 25(1):85, February 2024.

[8] Zeming Lin, Halil Akin, Roshan Rao, Brian Hie, Zhongkai Zhu, Wenting Lu, Nikita Smetanin, Robert Verkuil, Ori Kabeli, Yaniv Shmueli, Allan dos Santos Costa, Maryam Fazel-Zarandi, Tom Sercu, Salvatore Candido, and Alexander Rives. Evolutionary-scale prediction of atomic level protein structure with a language model, October 2022. Pages: 2022.07.20.500902 Section: New Results.

[9] Christoph Feinauer, Hendrik Szurmant, Martin Weigt, and Andrea Pagnani. Inter-protein sequence co-evolution predicts known physical interactions in bacterial ribosomes and the trp operon. *PLoS One*, 11(2):e0149166, February 2016.

[10] Elias T Friman, Ilya M Flyamer, Davide Marenduzzo, Shelagh Boyle, and Wendy A Bickmore. Ultra-long-range interactions between active regulatory elements. *Genome Res.*, 33(8):1269–1283, August 2023.

[11] Yiling Jiang, Kuijie Tong, Roubin Yao, Yuanze Zhou, Hanwen Lin, Liubing Du, Yunyun Jin, Liu Cao, Jingquan Tan, Xing-Ding Zhang, Deyin Guo, Ji-An Pan, and Xiaoxue Peng. Genome-wide analysis of protein–protein interactions and involvement of viral proteins in SARS-CoV-2 replication. *Cell & Bioscience*, 11(1):140, July 2021.

[12] Xindi Wang, Mahsa Salmani, Parsa Omidi, Xiangyu Ren, Mehdi Rezagholizadeh, and Armaghan Eshaghi. Beyond the Limits: A Survey of Techniques to Extend the Context Length in Large Language Models, May 2024. arXiv:2402.02244 [cs].

[13] Ashish Vaswani, Noam Shazeer, Niki Parmar, Jakob Uszkoreit, Llion Jones, Aidan N. Gomez, Lukasz Kaiser, and Illia Polosukhin. Attention Is All You Need, August 2023. arXiv:1706.03762 [cs].

[14] J Rodney Brister, Danso Ako-Adjei, Yiming Bao, and Olga Blinkova. Ncbi viral genomes resource. *Nucleic acids research*, 43(D1):D571–D577, 2015.

[15] Jianlin Su, Yu Lu, Shengfeng Pan, Ahmed Murtadha, Bo Wen, and Yunfeng Liu. RoFormer: Enhanced Transformer with Rotary Position Embedding, November 2023. arXiv:2104.09864 [cs].

[16] Ofir Press, Noah A. Smith, and Mike Lewis. Train Short, Test Long: Attention with Linear Biases Enables Input Length Extrapolation, April 2022. arXiv:2108.12409 [cs].

[17] Shouyuan Chen, Sherman Wong, Liangjian Chen, and Yuandong Tian. Extending Context Window of Large Language Models via Positional Interpolation, June 2023. arXiv:2306.15595 [cs].

[18] Bowen Peng, Jeffrey Quesnelle, Honglu Fan, and Enrico Shippole. YaRN: Efficient Context Window Extension of Large Language Models, November 2023. arXiv:2309.00071 [cs].

[19] Tri Dao, Daniel Y. Fu, Stefano Ermon, Atri Rudra, and Christopher Ré. FlashAttention: Fast and Memory-Efficient Exact Attention with IO-Awareness, June 2022. arXiv:2205.14135 [cs].

[20] Philippe Tillet, H. T. Kung, and David Cox. Triton: an intermediate language and compiler for tiled neural network computations. In *Proceedings of the 3rd ACM SIGPLAN International Workshop on Machine Learning and Programming Languages*, pages 10–19, Phoenix AZ USA, June 2019. ACM.

[21] Houda Bouamor, Juan Pino, and Kalika Bali, editors. *Proceedings of the 2023 Conference on Empirical Methods in Natural Language Processing*. Association for Computational Linguistics, Singapore, December 2023.

[22] Huiqiang Jiang, Qianhui Wu, Chin-Yew Lin, Yuqing Yang, and Lili Qiu. LLMLingua: Compressing Prompts for Accelerated Inference of Large Language Models, December 2023. arXiv:2310.05736 [cs].

[23] Genta Indra Winata, Samuel Cahyawijaya, Zhaojiang Lin, Zihan Liu, and Pascale Fung. Lightweight and efficient end-to-end speech recognition using low-rank transformer, 2020.

[24] Soroush Abbasi Koohpayegani and Hamed Pirsiavash. SimA: Simple Softmax-free Attention for Vision Transformers, March 2024. arXiv:2206.08898 [cs].

[25] Iz Beltagy, Matthew E. Peters, and Arman Cohan. Longformer: The Long-Document Transformer, December 2020. arXiv:2004.05150 [cs].

[26] Yukang Chen, Shengju Qian, Haotian Tang, Xin Lai, Zhijian Liu, Song Han, and Jiaya Jia. LongLoRA: Efficient Fine-tuning of Long-Context Large Language Models, March 2024. arXiv:2309.12307 [cs].

[27] Manzil Zaheer, Guru Guruganesh, Avinava Dubey, Joshua Ainslie, Chris Alberti, Santiago Ontanon, Philip Pham, Anirudh Ravula, Qifan Wang, Li Yang, and Amr Ahmed. Big Bird: Transformers for Longer Sequences, January 2021. arXiv:2007.14062 [cs].

[28] Aurko Roy, Mohammad Saffar, Ashish Vaswani, and David Grangier. Efficient Content-Based Sparse Attention with Routing Transformers, October 2020. arXiv:2003.05997 [cs].

[29] Nadav Brandes, Dan Ofer, Yam Peleg, Nadav Rappoport, and Michal Linial. ProteinBERT: a universal deep-learning model of protein sequence and function. *Bioinformatics*, 38(8):2102–2110, April 2022.

[30] Baris E Suzek, Yuqi Wang, Hongzhan Huang, Peter B McGarvey, Cathy H Wu, and UniProt Consortium. UniRef clusters: a comprehensive and scalable alternative for improving sequence similarity searches. *Bioinformatics*, 31(6):926–932, March 2015.

[31] Cody Martin, Anthony Gitter, and Karthik Anantharaman. Protein Set Transformer: A protein-based genome language model to power high diversity viromics, July 2024. Pages: 2024.07.26.605391 Section: New Results.

[32] Yunha Hwang, Andre Cornman, Elizabeth Kellogg, Sergey Ovchinnikov, and Peter Girguis. Genomic language model predicts protein co-regulation and function. 2024.

[33] Eric Nguyen, Michael Poli, Matthew G. Durrant, Armin W. Thomas, Brian Kang, Jeremy Sullivan, Madelena Y. Ng, Ashley Lewis, Aman Patel, Aaron Lou, Stefano Ermon, Stephen A. Baccus, Tina Hernandez-Boussard, Christopher Ré, Patrick D. Hsu, and Brian L. Hie. Sequence modeling and design from molecular to genome scale with Evo, February 2024. Pages: 2024.02.27.582234 Section: New Results.

[34] Zhihan Zhou, Yanrong Ji, Weijian Li, Pratik Dutta, Ramana Davuluri, and Han Liu. DNABERT-2: Efficient Foundation Model and Benchmark For Multi-Species Genome, March 2024. arXiv:2306.15006 [q-bio].

[35] Maxim Zvyagin, Alexander Brace, Kyle Hippe, Yuntian Deng, Bin Zhang, Cindy Orozco Bohorquez, Austin Clyde, Bharat Kale, Danilo Perez-Rivera, Heng Ma, Carla M. Mann, Michael Irvin, J. Gregory Pauloski, Logan Ward, Valerie Hayot, Murali Emani, Sam Foreman, Zhen Xie, Diangen Lin, Maulik Shukla, Weili Nie, Josh Romero, Christian Dallago, Arash Vahdat, Chaowei Xiao, Thomas Gibbs, Ian Foster, James J. Davis, Michael E. Papka, Thomas Brettin, Rick Stevens, Anima Anandkumar, Venkatram Vishwanath, and Arvind Ramanathan. GenSLMs: Genome-scale language models reveal SARS-CoV-2 evolutionary dynamics, October 2022. Pages: 2022.10.10.511571 Section: New Results.

[36] Michael Poli, Stefano Massaroli, Eric Nguyen, Daniel Y. Fu, Tri Dao, Stephen Baccus, Yoshua Bengio, Stefano Ermon, and Christopher Ré. Hyena Hierarchy: Towards Larger Convolutional Language Models, April 2023. arXiv:2302.10866 [cs].

[37] Michael Poli, Jue Wang, Stefano Massaroli, Jeffrey Quesnelle, Ryan Carlow, Eric Nguyen, and Armin Thomas. Stripedhyena: Moving beyond transformers with hybrid signal processing models. 12 2023.

[38] Alexander Rives, Joshua Meier, Tom Sercu, Siddharth Goyal, Zeming Lin, Jason Liu, Demi Guo, Myle Ott, C. Lawrence Zitnick, Jerry Ma, and Rob Fergus. Biological structure and function emerge from scaling unsupervised learning to 250 million protein sequences. *Proc Natl Acad Sci U S A*, 118(15):e2016239118, April 2021.

[39] Rachel A. Keown, Jacob T. Dums, Phillip J. Brumm, Joyanne MacDonald, David A. Mead, Barbra D. Ferrell, Ryan M. Moore, Amelia O. Harrison, Shawn W. Polson, and K. Eric Wommack. Novel viral dna polymerases from metagenomes suggest genomic sources of strand-displacing biochemical phenotypes. *Frontiers in Microbiology*, Volume 13 - 2022, 2022.

[40] Eugene V. Koonin, Valerian V. Dolja, and Mart Krupovic. The logic of virus evolution. *Cell Host & Microbe*, 30(7):917–929, 2022.

[41] Daniel J Nasko, Jessica Chopyk, Eric G Sakowski, Barbra D Ferrell, Shawn W Polson, and K Eric Wommack. Family a DNA polymerase phylogeny uncovers diversity and replication gene organization in the virioplankton. *Front. Microbiol.*, 9:3053, December 2018.

[42] torch.sparse_bsr_tensor — PyTorch 2.6 documentation.

[43] Damian Szklarczyk, Rebecca Kirsch, Mikaela Koutrouli, Katerina Nastou, Farrokh Mehryary, Radja Hachilif, Annika L. Gable, Tao Fang, Nadezhda T. Doncheva, Sampo Pyysalo, Peer Bork, Lars J. Jensen, and Christian von Mering. The STRING database in 2023: protein-protein association networks and functional enrichment analyses for any sequenced genome of interest. *Nucleic Acids Res*, 51(D1):D638–D646, January 2023.

[44] Milot Mirdita, Martin Steinegger, and Johannes Söding. MMseqs2 desktop and local web server app for fast, interactive sequence searches. *Bioinformatics*, 35(16):2856–2858, August 2019.

[45] William L Harrigan, Barbra D Ferrell, K Eric Wommack, et al. Improvements in viral gene annotation using large language models and soft alignments. *BMC Bioinformatics*, 25:165, 2024.

[46] Rajan Sawhney et al. Fine-tuning protein language models unlocks the potential of underrepresented viral proteomes. *PeerJ*, 13:e19919, 2025.

Supplementary Material

S1 NCBI Virus Database

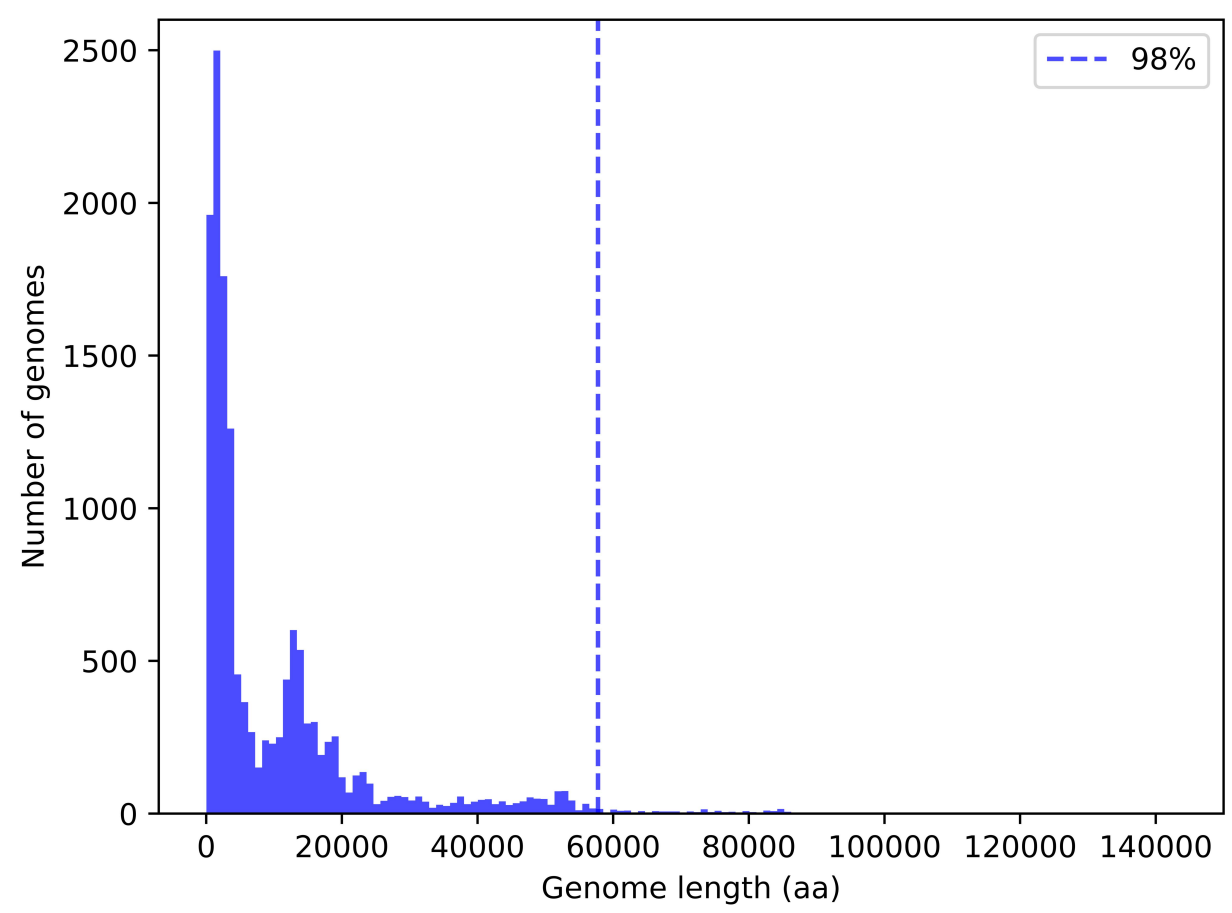

Supplementary Figure S1: **Viral genome length distributions in the NCBI Virus Database.** This histogram shows that a context length of 61k amino acids allows us to process over 98% of the 14,436 complete genomes of the database.

S2 ~~Optimization~~ Development of the PPI Inference Model (Stage 1)

To extend ESM-2's context window for PPI inference, we evaluated two positional encoding strategies (RoPE and ALiBi) and three sparse attention mechanisms (Longformer, Shifted Sparse Attention, and BigBird).

The evaluation of positional encoding strategies showed that ALiBi exhibited superior performance in handling extended sequences. Although RoPE demonstrated robust performance within its original training context, its effectiveness deteriorated significantly beyond 4,000 aa. In contrast, ALiBi maintained consistent perplexity (1.99) across sequence lengths extending up to 13,000 aa (Supplementary Figure S2).

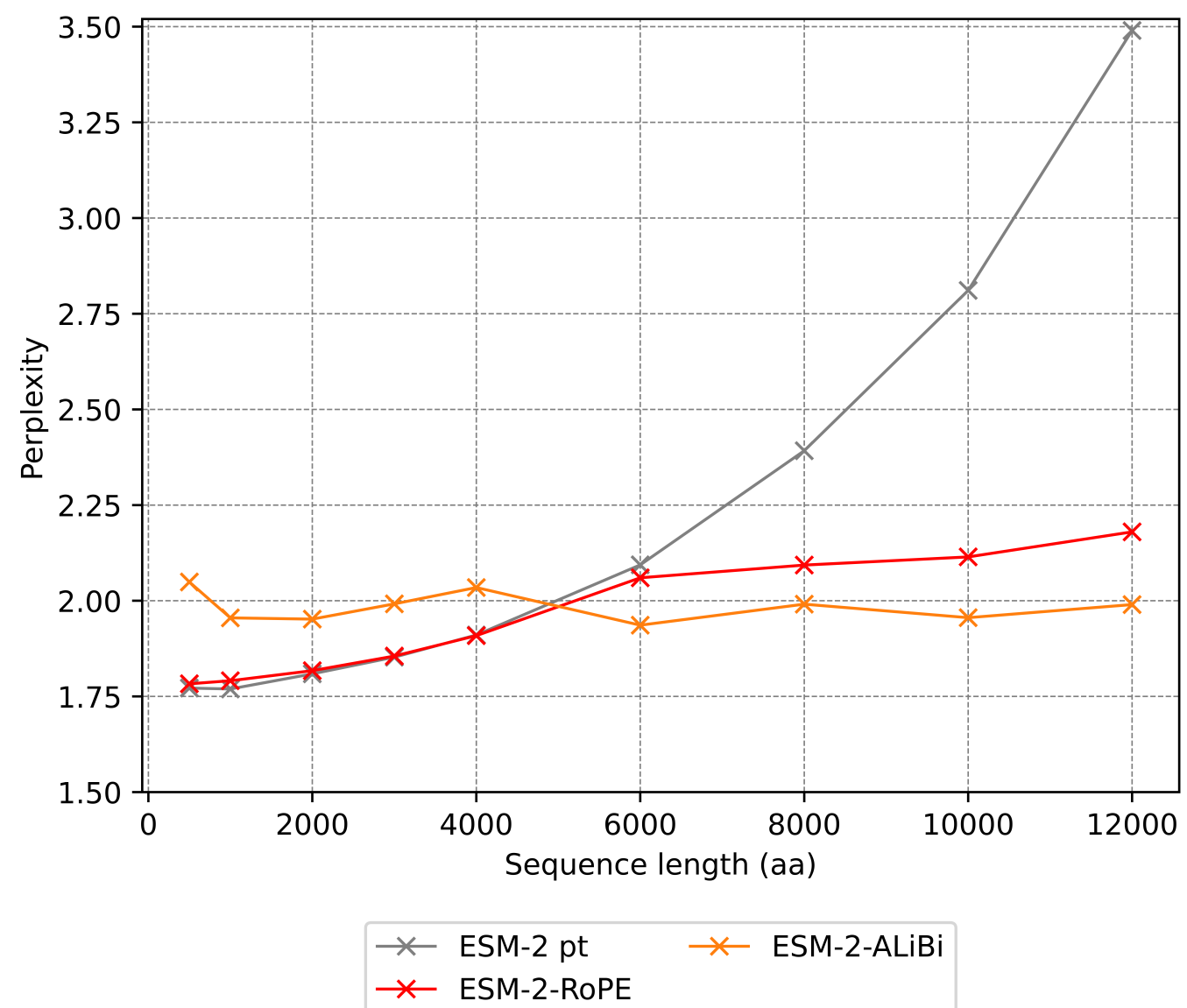

Supplementary Figure S2: **Perplexity evaluation of positional encoding methods for fine-tuning.** Performance metrics for ESM-2-pt and fine-tuned variants using Sliding Window (SW) attention mechanism with RoPE and ALiBi positional encoding. Perplexity values represent mean across sequences ranging from 500 to 13,000 amino acids.

Among the sparse attention mechanisms tested, Longformer provided the optimal balance between performance (perplexity: 1.99, silhouette score: 0.88) and computational efficiency (training time: 32s/step or 46 hours) (Supplementary Table S1). Based on these findings, we adopted ALiBi positional encoding combined with Longformer attention for ESM-2-PPI.

	ESM-2-pt	Longformer	Longformer + full	S^2-Attn	S^2-Attn + full	BigBird
Training context size	1,024	4,000	4,000	4,000	4,000	4,000
Training time (s/step)		32	32	36	36	126
Perplexity	3.17	2.18	**1.99**	2.22	2.05	2.02
Silhouette	0.65	0.85	**0.88**	0.86	0.85	0.84

Supplementary Table S1: **Performance metrics of ESM-2-pt and fine-tuned versions using different sparse attention patterns.** Longformer refers to the Sliding Window Attention pattern from [25], S^2-Attn to the Shifted Sparse Attention from [26] and BigBird to the eponymous attention pattern from [27]. '+full' means normalization and embedding layers are trainable during low-rank fine-tuning. Metrics are calculated on genome fragments ranging between 10,000 and 13,000 amino acids.

To validate that ESM-2-PPI captures biologically meaningful interactions, we analyzed genomic fragments from 83 viral genomes containing complete PolA, RNR, and HEL proteins. These genomes were identified by searching the

NCBI Virus database using BLAST and soft alignment [45, 46] against reference sequences for each protein, retaining only genomes where all three were detected with high confidence (e-value $< 10^{-3}$, coverage $> 80\%$). Supplementary Figure S3 shows that these known interacting pairs consistently rank among the highest-scoring interactions, particularly after excluding genomically adjacent pairs. A Wilcoxon–Mann–Whitney test confirmed a highly significant difference between the distributions ($p = 3.31 \times 10^{-22}$).

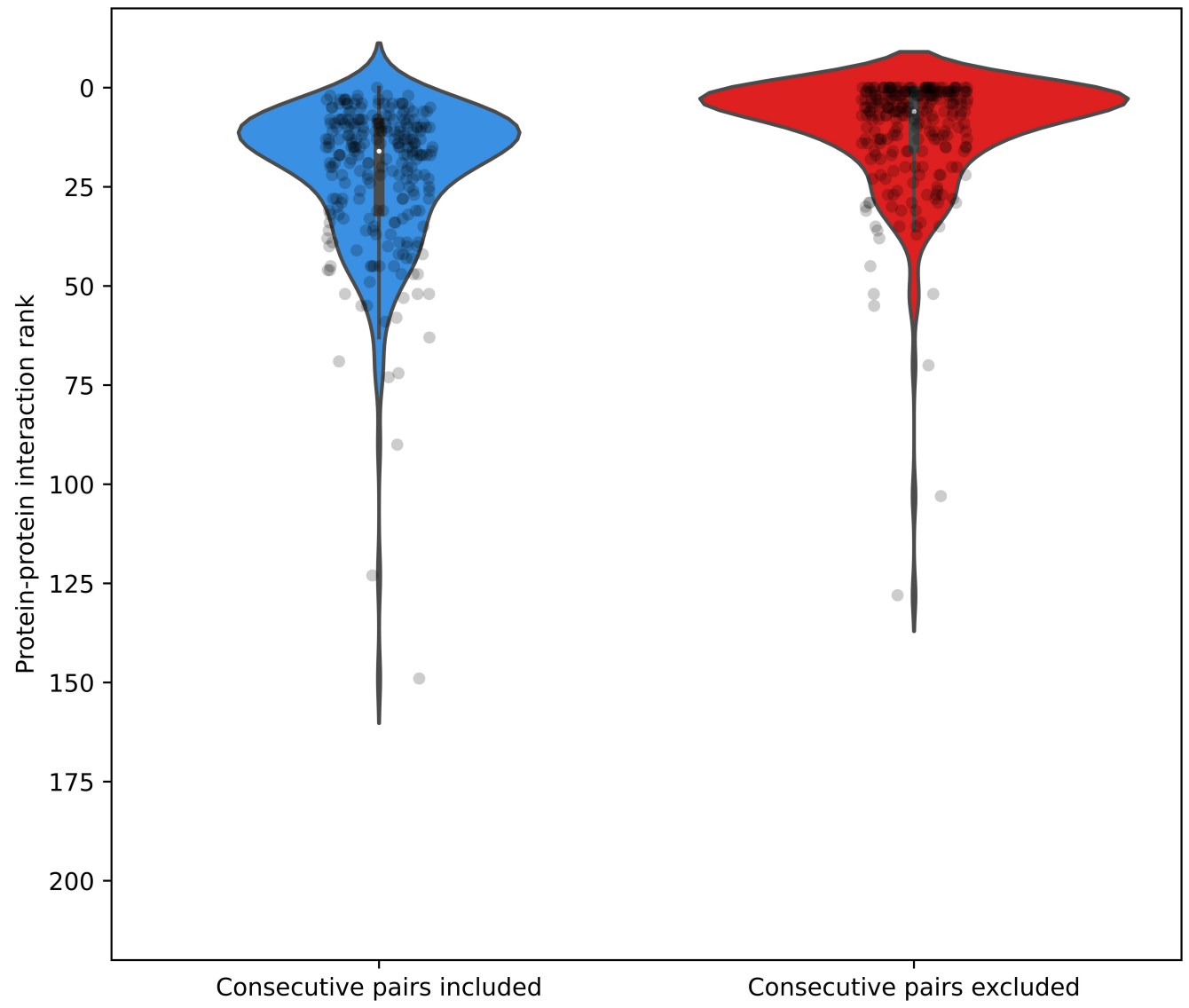

Supplementary Figure S3: **Distribution of interaction pair rankings of DNA polymerase I (PolA), ribonucleotide reductase (RNR), and helicase (HEL) across 83 viral genomes.** Violin plots represent the ranking positions of PolA-HEL, PolA-RNR, and RNR-HEL pairs (total n = 249). Left panel: rankings among all protein pairs within fragments. Right panel: rankings after excluding genomically adjacent protein pairs.

To select an appropriate interaction threshold, we evaluated different values of k (the number of top-ranked non-adjacent pairs to include). Supplementary Figure S4 and Supplementary Table S2 show that $k = 50$ retains 97.6% of known interacting pairs while remaining computationally tractable.

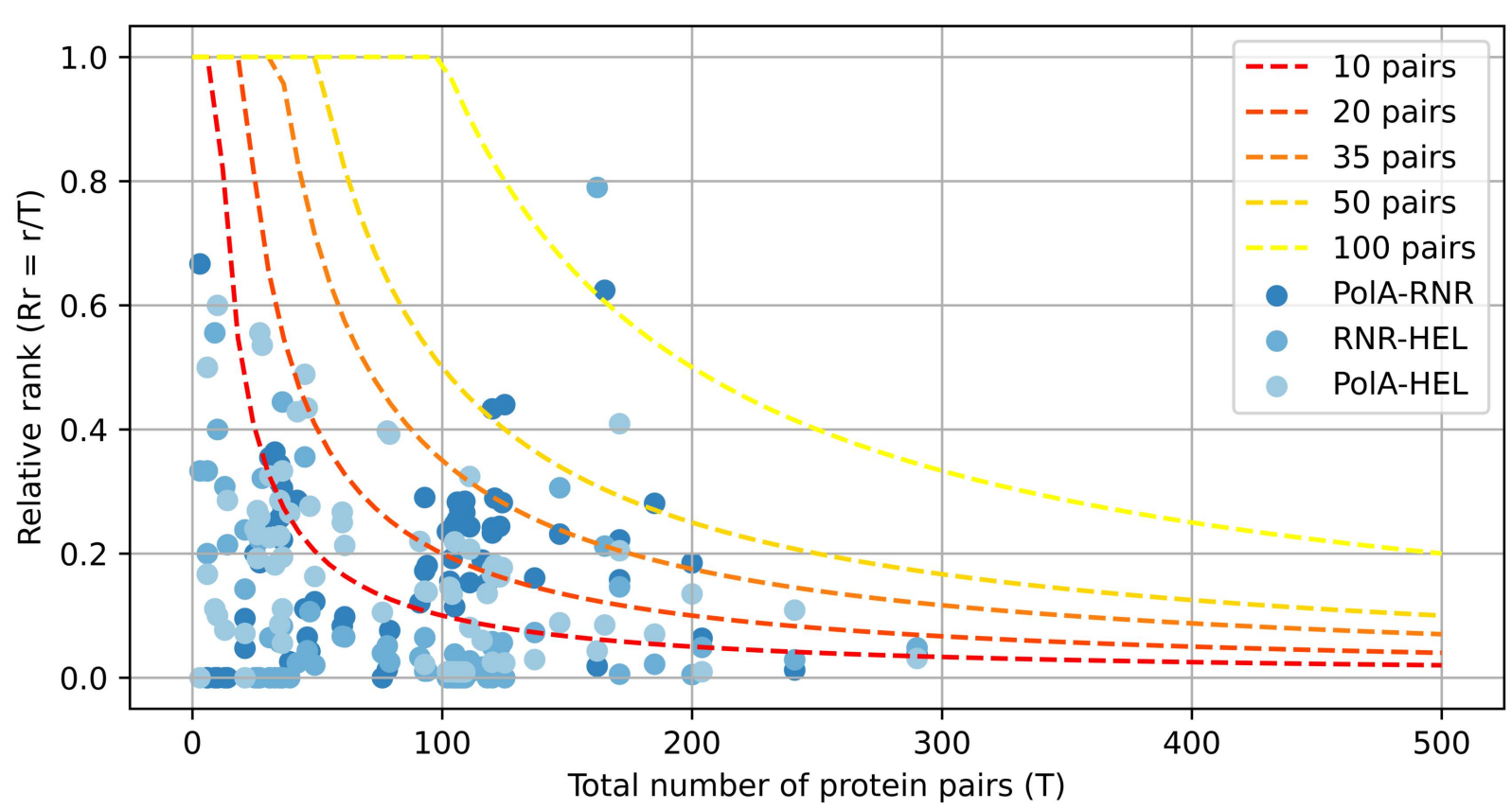

Supplementary Figure S4: **Selection of interaction threshold.** PolA-HEL, PolA-RNR, and RNR-HEL pairs shown as a function of total genomic pairs and relative genomic rank. Dashed curves denote selection thresholds; pairs below each curve are retained for attention calculations.

Threshold (number of selected pairs)	10	20	35	50	100
% studied pairs selected	64.0%	80.6%	94.4%	97.6%	99.2%

Supplementary Table S2: **Percentage of known interacting pairs** (PolA-RNR-HEL) captured at different selection thresholds.

S3 Using embeddings to predict taxonomy

To assess the taxonomic discriminability of the learned embeddings, we conducted a supervised classification analysis. For each protein or genome in the dataset, a 1,280-dimensional embedding vector was generated and paired with its corresponding taxonomic label. The dataset, comprising 100,000 proteins and 5,000 genomes, was randomly partitioned into training (80%) and validation (20%) subsets. To ensure a robust evaluation, we varied the number of taxonomic species from 5 to 100, as described in Section 3.9. A single-layer neural network with an intermediate hidden dimension of 2,560 was trained for 1,000 epochs using a learning rate of 1e-3. Model performance was evaluated using 5-fold cross-validation, and standard classification metrics were reported.

S4 Validation Using Protein Domain Annotations

To assess whether our embeddings capture functional information beyond taxonomic signals, we evaluated clustering and classification performance using Pfam protein family annotations. Pfam families group proteins by conserved domain structure and function, providing an orthogonal validation axis to taxonomy-based evaluations.

Supplementary Figure S5 presents silhouette scores and classification F1-scores using Pfam families as labels. Notably, silhouette scores are negative across all models (Supplementary Figure S5a), indicating that proteins from the same Pfam family do not form tight clusters in embedding space. This result is expected and reflects a key design principle of our approach: our embeddings are trained to encode genomic context, not solely domain identity. A helicase in a bacteriophage and a helicase in an RNA virus belong to the same Pfam family but occupy fundamentally different genomic contexts, and our models are designed to distinguish them. Embeddings that clustered purely by Pfam family would simply recapitulate single-protein models like ESM-2, rather than capturing the genomic relationships that are the focus of this work.

Despite this, classification performance remains strong (Supplementary Figure S5b), with LV-3C and LV-5B consistently outperforming ESM-2-pt and PST across all numbers of Pfam families evaluated. This indicates that Pfam-relevant information is encoded within the embeddings—it is simply not the dominant organizing signal. A linear classifier can extract domain identity even when genomic context is the primary structuring principle of the embedding space.

Taken together, these results demonstrate that our context-aware embeddings capture functional domain information at least as effectively as single-protein baselines, while additionally encoding genomic context that distinguishes proteins with identical domains but different genomic environments.

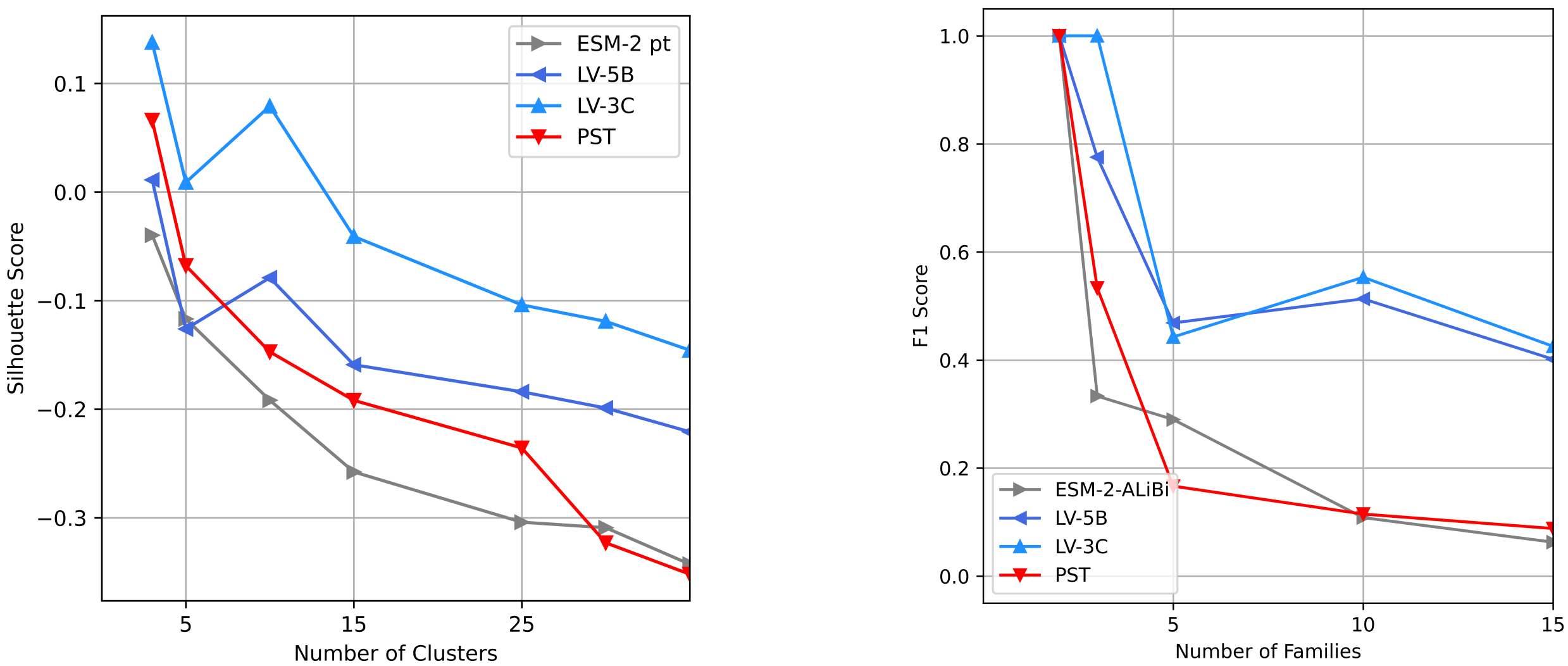

(a) Silhouette Score (b) Classification

Supplementary Figure S5: **Evaluation of embeddings using Pfam domain annotations. (a)** Silhouette scores using Pfam families as cluster labels. Negative scores across all models reflect that genomic context—not domain identity alone—is the primary organizing principle of the embedding space. **(b)** F1-scores for Pfam family classification. Despite low clustering coherence, LV-3C and LV-5B achieve strong classification performance, indicating that domain information is encoded but not dominant.

S5 Model Performance Across Viral Subgroups

Viral genomes vary substantially in size, structure, and evolutionary dynamics. To assess whether our models generalize across this diversity, we evaluated performance on RNA viruses—the most challenging and abundant subgroup in our dataset (approximately 70% of training genomes).

RNA viruses present unique modeling challenges: their high mutation rates and rapid evolution generate greater sequence diversity, while their broad host ranges and varied genome organizations (segmented, ambisense, positive/negative-sense) introduce additional complexity. If our models perform well on this demanding subgroup, we would expect comparable or better performance on structurally simpler dsDNA and ssDNA viruses.

Supplementary Figure S6 compares model performance on RNA viruses (solid lines) versus all viruses (lighter lines). As expected, performance on RNA viruses is slightly lower than on the full dataset; the latter being lifted by the inclusion of less complex viral groups. Critically, however, LV-3C and LV-5B consistently outperform both ESM-2-pt and PST baselines on RNA viruses across all evaluation metrics. These results demonstrate that our models capture biologically meaningful signals even within the most challenging viral subgroup, suggesting robust generalization across viral diversity.

S6 Controls for the Interaction-Inference Step

To test whether the interaction-inference step itself improves the model, we ran a controlled comparison in which the only parameter that varied was which long-range links each model received. The random conditions use three independent draws whose results we average. Additionally, because the inferred links tend to fall at particular genomic distances, we also included a distance-matched control that draws random links at distances similar to those of the inferred pairs, so that any difference cannot be attributed to genomic distance alone. Together with a model that receives no long-range links and connects only neighboring proteins, this gives eight models.

- **Inferred** (1 model). The 50 top-ranked pairs from the interaction-inference model (ESM-2-PPI).
- **Random** (3 models). 50 pairs drawn uniformly at random, averaged over three independent draws.

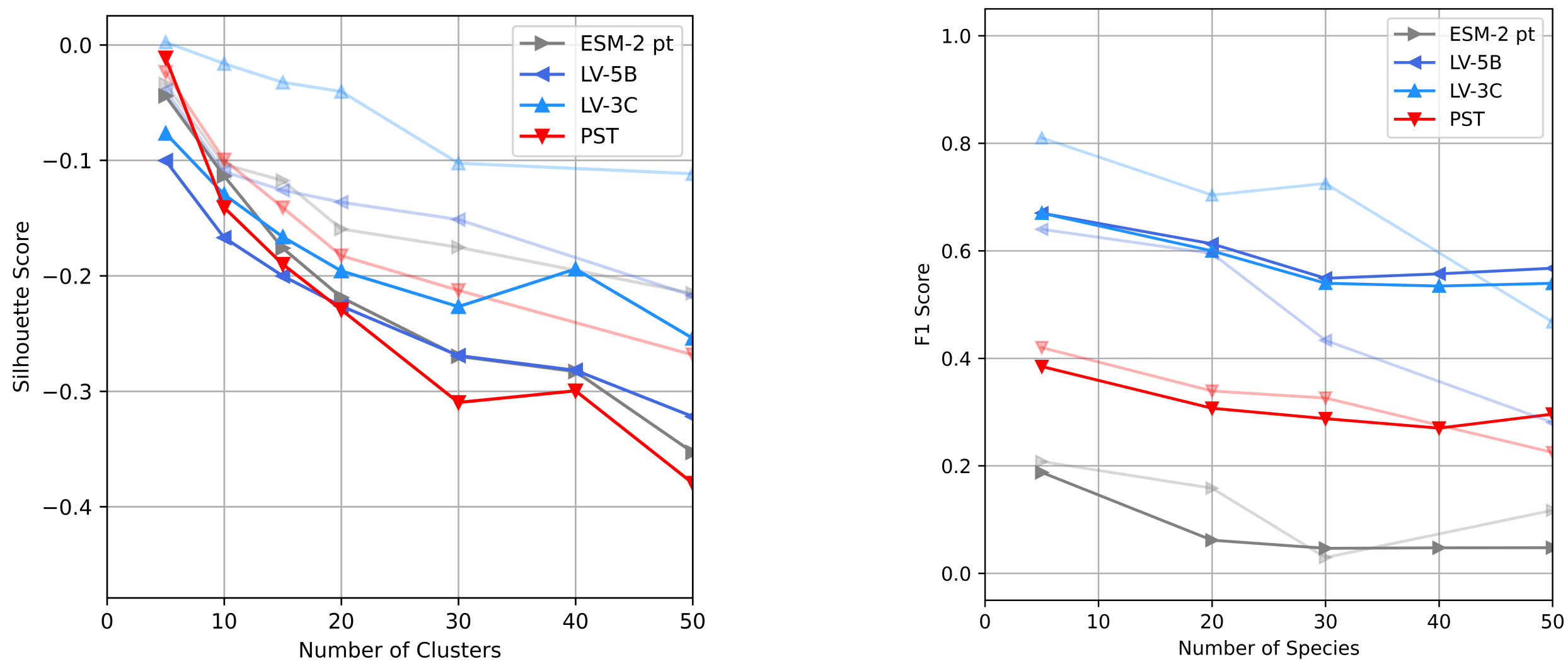

(a) Silhouette Score (b) Classification

Supplementary Figure S6: **Model performance on RNA viruses compared to all viruses. (a)** Silhouette scores and **(b)** F1-scores for taxonomic species classification, comparing LV-3C, LV-5B, PST, and ESM-2-pt. Solid lines: RNA viruses only; lighter lines: all viruses. Despite the greater complexity of RNA viruses, our models maintain consistent improvements over baselines.

- **Distance-matched** (3 models). 50 random pairs drawn to match the genomic-distance range of the inferred pairs, averaged over three draws.
- **No-link** (1 model). Only neighboring proteins are connected.

We used the ESM-2-35M-parameter model so that all eight could be trained from scratch under identical conditions. All models share the same architecture, initial weights, training data and order, masking, and optimizer, so any difference in their results can come only from which pairs are connected.

Table S3 reports held-out perplexity on 600 genomes. Adding 50 random links leaves perplexity essentially unchanged relative to having no long-range links (16.124 versus 16.120), whereas adding the same number of inferred links lowers it (lower is better). The distance-matched control performs like the other controls, so the advantage reflects which proteins are paired rather than how far apart they occur.

Model	Held-out perplexity
No long-range links	16.120
50 random links (average of 3)	16.124
50 distance-matched links (average of 3)	16.119
50 inferred links	**16.064**

Supplementary Table S3: **Held-out perplexity for the interaction-inference controls.** Lower is better. Only the inferred links lower perplexity relative to having no long-range links.

The genome-wide change is modest (approximately 0.4%), but this figure represents an average across all positions in the genome. Because the 50 links are relevant only to the minority of positions that occur within an inferred PPI link, the majority of positions are unaffected. Averaging this small number of positions against the large number of unaltered ones minimizes the magnitude of the effect. The effect is nonetheless consistent across genomes as the inferred model achieves lower perplexity than the random average on 554 of 600 genomes (92%), and it outperforms the lowest perplexity attained by any of the three random models on a per-genome basis for 78% of genomes.

The improvement in perplexity is also concentrated among the pairs assigned the highest scores by the inference model. We ranked positions according to the reduction in per-position loss conferred by the inferred links relative to the control,

and examined the distribution of the most-improved positions. In Table S4, the baseline rate denotes the fraction of the genome occupied by the proteins in the retained pairs—the value expected by chance—and the top-100 enrichment quantifies how much more frequently the 100 most-improved positions coincide with those pairs than this baseline predicts. The enrichment increases with the confidence of the pairs considered, ranging from approximately 1.4-fold when all 50 pairs are pooled to approximately 11-fold for the highest-scoring pairs. The improvement therefore scales with the model's inferred PPI score rather than emerging at an arbitrary threshold.

Edges kept (by confidence)	Baseline rate	Top-100 enrichment
top 2%	4.1%	11.1×
top 5%	9.0%	7.2×
top 10%	13.8%	5.2×
top 20%	25.6%	3.1×
top 50%	50.4%	1.7×
all inferred pairs	71.6%	1.4×

Supplementary Table S4: **Enrichment of improved positions on high-scoring inferred pairs.** The most-improved positions fall disproportionately on the proteins the inference model ranks most highly.

With every other setting held identical, 50 randomly chosen links give no measurable improvement over having no long-range links, whereas 50 links chosen by the inference model lower held-out perplexity. The advantage is consistent across genomes and concentrates on the pairs the model scores highest, which are the same pairs an independent, separately validated model ranks as most likely to interact.

Manuscript Click here to access/download;Manuscript;manuscript (3).pdf

Extending Protein Language Models to a Viral Genomic Scale Using Biologically Induced Sparse Attention

Thibaut Dejean
ORCID: 0009-0001-2394-6283
Department of Information and Computer Sciences
University of Hawaii
Honolulu, HI

Barbra D. Ferrell
ORCID: 0000-0003-3057-3478
Department of Computer & Information Sciences
Delaware Biotechnology Institute
University of Delaware, Newark, DE

William Harrigan
ORCID: 0000-0003-2935-6863
Hawai'i Institute of Marine Biology
University of Hawai'i
Kaneohe, HI

Zachary D. Schreiber
ORCID: 0000-0002-6271-2754
Department of Computer & Information Sciences
Delaware Biotechnology Institute
University of Delaware, Newark, DE

Rajan Sawhney
ORCID: 0009-0007-0060-759X
Department of Information and Computer Sciences
University of Hawaii
Honolulu, HI

K. Eric Wommack
ORCID: 0000-0001-5797-7128
Department of Plant & Soil Sciences
Delaware Biotechnology Institute
University of Delaware, Newark, DE

Shawn W. Polson
ORCID: 0000-0002-3398-6932
Department of Computer & Information Sciences
Delaware Biotechnology Institute
University of Delaware, Newark, DE

Mahdi Belcaid*
ORCID: 0009-0004-5185-0643
Department of Information and Computer Sciences
University of Hawaii
Honolulu, HI
mahdi@hawaii.edu
*Corresponding author

Abstract

The transformer architecture in deep learning has revolutionized protein sequence analysis. Recent advancements in protein language models have paved the way for significant progress across various domains, including protein function and structure prediction, multiple sequence alignments and mutation effect prediction. A protein language model is commonly trained on individual proteins, ignoring the interdependencies between sequences within a genome. However, biological understanding reveals that protein–protein interactions span entire genomic regions, underscoring the limitations of focusing solely on individual proteins. To address these limitations, we propose a novel approach that extends the context size of transformer models across the entire viral genome. By training on large genomic fragments, our method captures *putative* long-range dependencies consistent with

inter-protein relationships and encodes protein sequences with integrated information from distant proteins within the same genome, offering benefits across downstream tasks. Viruses, with their densely packed genomes, minimal intergenic regions, and protein annotation challenges, are ideal candidates for genome-wide learning. We introduce a long-context protein language model, trained on entire viral genomes, leveraging a biologically informed sparse attention mechanism in which inter-protein links are *inferred computationally* and used as sparsity priors. Our semi-supervised approach supports long sequences of up to 61,000 amino acids (aa). Our evaluations show improved prediction of masked amino acids and improved downstream discrimination relative to single-protein models and long-context baselines, with additional validation that our inferred links correlate with independently curated interaction resources.

1 Introduction

Deep learning has transformed the analysis of genomic data, driven by transformer models [1, 2]. This shift produced powerful protein language models such as ESM-2, ProtTrans, and ProGen [3, 4, 5], which capture complex relationships between amino acids (aa) within proteins and yield high-dimensional embeddings that are effective across tasks including protein function annotation [6], multiple sequence alignment [7], and structure prediction [8].

However, by focusing on individual proteins, these models miss crucial interactions between distant proteins that shape genome function. While proximity-based interactions such as those within operons are well studied [9], growing evidence highlights long-range protein interactions across large genomic distances [10]; in evolutionary terms, when one protein changes, its partners often must adapt to preserve their collective function [11]. Yet transformer-based models, constrained by limited context windows (e.g., ESM-2 is limited to 1,024 aa, or tokens), cannot process multiple long proteins simultaneously, preventing them from modeling the web of protein interactions across entire genomes.

Recent large language models (LLMs) show that extended context improves the capture of long-range relationships in text [12], suggesting that analogous approaches could yield more accurate protein embeddings and reveal functional relationships between distant proteins that current methods miss. However, extending context is computationally demanding, primarily because attention scales quadratically with sequence length [13]. Existing solutions—from sparse attention patterns to architectural modifications—are optimized for text and do not account for the principles of protein biology, limiting their effectiveness on multiple protein sequences.

To address these limitations, we introduce a protein language model that processes entire viral genomes, achieving a context window exceeding 60,000 aa on a single commodity GPU —covering 98% of all sequenced viral genomes in the NCBI Virus database [14] (Supplementary Figure S1). Our semi-supervised model employs sparse attention guided by computationally inferred protein–protein interactions, and our evaluations show that the resulting viral genome embeddings capture biologically meaningful signals missed by current single-protein models.

2 Related Work

2.1 Long Context LLMs

Recent advances in large language models have sparked intense research into efficiently handling long contexts. Approaches to increasing context length fall broadly into four popular categories [12] : 1) context length extrapolation, 2) hardware and algorithmic strategies, 3) prompt compression, and 4) attention matrix approximation. Context length extrapolation methods enable models to process sequences longer than those on which they were initially trained by manipulating positional embeddings. Hardware and algorithmic strategies focus on optimizing computation and memory usage through system optimizations. Prompt compression methods reduce the sequence length by condensing the input information into more compact representations before processing. Lastly, attention matrix approximation employs computational and statistical strategies to estimate attention mechanisms without incurring the quadratic scaling costs of standard attention. We briefly review these approaches below since our approach builds on advances from several of them. From context length extrapolation methods (Section 2.1.1), we adopt ALiBi positional encoding for stable inference on extended sequences. From attention matrix approximation strategies (Section 2.1.4), we build on sparse attention patterns similar to Longformer, but replace fixed patterns with biologically informed sparsity derived from inferred protein-protein interactions (PPI). We leverage ESM-2 both as a foundation for PPI inference and as initialization for one of our models. This section provides background on each of these areas to contextualize our contributions.

2.1.1 Context Length Extrapolation

A fundamental limitation in extending transformer models to longer sequences lies in Positional Encodings (PEs). While PEs are essential for encoding the sequential order of tokens, their effectiveness diminishes significantly when processing sequences that exceed the training data length. This constraint represents a critical bottleneck when adapting models trained on shorter sequences to handle longer inputs.

Approaches such as rotational position embeddings (RoPE) [15] introduced trainable embeddings that apply position-based rotations to query and key representations. However, traditional PEs suffer substantial performance degradation when processing sequences that are longer than their training context. Two families of methods address this without full retraining: extrapolation and interpolation. Extrapolation methods, exemplified by Attention with Linear Biases (ALiBi) [16], introduce unlearned static biases into attention calculations, systematically adjusting attention scores between key–query pairs based on distance. In contrast, interpolation methods generalize learned PEs to longer sequences, for example by scaling RoPE position indices to the original context window [17].

2.1.2 Hardware and Algorithmic strategies

Training transformer models directly on longer sequences would eliminate the need for positional embedding extrapolation techniques. However, this approach is fundamentally constrained by the quadratic memory complexity inherent to the attention mechanism. Specifically, standard attention mechanisms require memory and computational complexity of $O(n^2 d + nd^2)$ per layer, where n represents the sequence length and d denotes the hidden dimension size.

Distributed computing architectures address these constraints by leveraging model parallelism, distributing the memory and computation demands of attention across multiple GPUs.

Recent algorithmic innovations have substantially improved the efficiency of attention computation without altering its theoretical complexity. For example, FlashAttention [18] optimizes memory access patterns through fused kernel operations implemented in specialized computing frameworks such as Triton [19]. These practical optimizations reduce memory requirements significantly while preserving computational performance.

2.1.3 Prompt Compression

Prompt compression methods aim to reduce input dimensionality by exploiting redundancy within input sequences, ideally preserving semantic content. However, this approach introduces a fundamental limitation in protein sequence analysis: compressed inputs cannot generate detailed per-residue embeddings for every amino acid, as compression inherently reduces sequence representation granularity. Consequently, prompt compression is less suitable for tasks requiring residue-level detail, such as protein structure prediction or mutation effect prediction.

2.1.4 Attention Matrix Approximation

An alternative approach to managing extended contexts involves approximating rather than explicitly computing the full attention matrix. This strategy includes two primary methodologies: context-agnostic and context-aware sparse matrices, each offering distinct trade-offs between computational efficiency and representational capacity.

Context-Agnostic Sparse Matrices. Context-agnostic approaches address the quadratic complexity of traditional self-attention through systematic approximation techniques [13]. Among the most successful implementations are architectures leveraging predefined sparse attention patterns, exemplified by Longformer, LongLoRA, and BigBird [20, 21, 22], each employing distinct sparsification strategies (Figure 1). Longformer implements dilated sliding-window attention, strategically positioning sparse attention windows around tokens (Figure 1a). LongLoRA extends context length by introducing shifted attention patterns during fine-tuning (Figure 1b). BigBird employs a hybrid approach combining global attention for select tokens, sliding-window attention, and random attention connections (Figure 1c). While BigBird enables whole-sequence consideration, the stochastic nature of random attention introduces variability in performance, as randomly selected attention positions lack inherent semantic or functional relevance. Although these approaches successfully extend context windows, their predetermined patterns remain independent of sequence content and semantic meaning, presenting inherent limitations. Most notably for biological applications, these methods cannot incorporate domain-specific knowledge—such as experimentally validated protein–protein interactions or genomic relationships—into attention calculations.

Context-Aware Sparse Matrices. In contrast to context-agnostic methods, context-aware compression approaches dynamically adjust their attention patterns based on input content. For example, the Routing Transformer [23] employs K-means clustering on key and query vectors to construct sparse, content-dependent attention matrices.

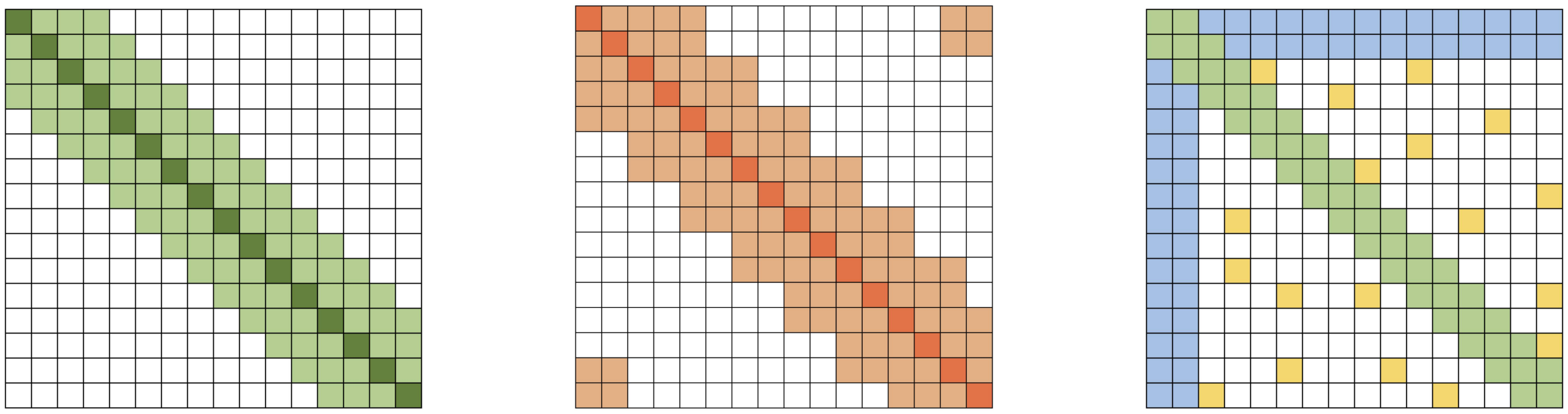

(a) Longformer (b) LongLoRA (c) BigBird

Figure 1: **Sparse attention patterns implemented in context-agnostic transformer architectures. (a)** Longformer's dilated sliding-window attention pattern, showing a diagonal band of attention with varying intensities of green. **(b)** LongLoRA's shifted attention pattern during fine-tuning, displayed as orange blocks. **(c)** BigBird's hybrid approach combining global attention (blue columns), sliding-window attention (green diagonal), and random attention connections (scattered yellow squares). These predetermined sparse attention patterns reduce computational complexity while enabling longer context windows, though they operate independently of sequence content and semantic meaning.

However, the exclusive reliance on clustering for routing attention presents significant limitations when analyzing biological sequences. Crucially, clustering-based methods may fail to capture interactions between functionally related but structurally and sequentially distinct elements, such as complementary protein domains that co-evolved despite limited sequence similarity.

2.2 Protein & Genome Language Modeling

2.2.1 Protein Language Models

Current popular protein language models (pLMs), including ProtBert [24], ProtTrans [4], and ProGen2 [5], utilize transformer-based architectures with context windows ranging from 512 aa (ProtTrans-XL) to 2,048 aa (ProGen2). These context limitations have largely confined pLMs to analyzing individual proteins in isolation, neglecting their broader genomic context. Among these, the ESM-2 model [3], trained on the UniRef50 database [25] via masked language modeling, has emerged as one of the most widely adopted pLMs. ESM-2 demonstrates impressive performance in protein contact prediction—attributed to its effective attention mechanism—and generates biologically meaningful embeddings useful across diverse downstream applications [6, 7, 8]. However, ESM-2, like its predecessors, focuses exclusively on single-protein sequences with a restricted context length of only 1,024 aa, thereby limiting its ability to capture inter-protein relationships within genomic contexts.

To integrate genomic context into protein representations, the Protein Set Transformer (PST) [26] introduces a graph-based framework wherein proteins, initially embedded via ESM-2, form nodes connected by edges representing genomic proximity. Despite these advantages, PST relies on precomputed single-protein ESM-2 embeddings and is constrained to modeling interactions between consecutive proteins.

2.2.2 Genomic Language Models

Recognizing the inherent limitations of analyzing proteins in isolation has spurred the development of genomic language models (gLMs) [27], which augment ESM-2 embeddings by incorporating context from larger genomic regions. For instance, recent gLMs utilize 30-gene fragments in a masked prediction task, enabling the prediction of protein embeddings by considering the contextual influence of 29 neighboring proteins. This approach generates enriched representations that significantly improve performance across tasks such as protein function prediction and taxonomic classification. However, existing gLMs primarily utilize conventional attention mechanisms, highlighting potential opportunities for incorporating biologically informed sparse attention patterns to further enhance their capabilities.

While existing methods have significantly advanced general language modeling and biological sequence analysis, important limitations remain. Current context-extension techniques in language models typically lack biological specificity. Although extrapolation methods such as ALiBi demonstrate effectiveness in general language tasks, they inherently assume consistent structural patterns between short and extended sequences (for instance, local grammatical

structures remain stable regardless of sequence length). This assumption does not hold for multi-protein sequences, where relationships between distant proteins can fundamentally differ from the local sequence patterns learned by single-protein models. Additionally, while content-dependent sparse attention mechanisms show promise for managing extended contexts, their potential for explicitly incorporating biological priors into language model architectures remains largely unexplored.

To address these limitations, we introduce a novel genome-scale protein language model architecture specifically designed for viral sequences. Our approach synthesizes recent advances in context-length extension methods with biologically informed sparse attention mechanisms, enabling the effective processing of significantly longer amino acid sequences on commodity hardware while explicitly modeling inter-protein interactions. The following sections detail our methodology and validate its effectiveness across multiple downstream biological tasks.

3 Methods

3.1 Datasets and Preprocessing

Sources and retrieval. We used the NCBI Virus database (retrieved 2024-06), selecting only genomes annotated as "complete" (14,436 genomes) (Supplementary Materials S1, Supplementary Figure S1). The list of loci used is included in the Github repository (`https://github.com/Hawaii-Bioinformatics/ViralEmbed`). For downstream classification tasks, the final dataset comprised 100,000 proteins from 5,000 genomes after de-duplication.

Protein non-redundancy. To prevent highly abundant sequences from biasing training, we applied CD-HIT clustering at 90% sequence identity, reducing the dataset by approximately 50%. Some viral proteins occur dozens or hundreds of times in the database; without deduplication, such sequences would dominate gradient updates and bias learned representations. This threshold collapses near-identical sequences while preserving biologically distinct variants.

Splits. To prevent data leakage, we partitioned the dataset at the genome level: all proteins from a given genome were assigned to the same split (train, validation, or test). Splits were stratified by taxonomic species to maintain balanced representation across partitions. The final proportions were 80% train, 10% validation, and 10% test. This design ensures that no protein from a genome in the test set could have been seen during training, either directly or through related proteins from the same genome.

Fragments. For long-context baselines, we constructed fragments by concatenating proteins in genomic order with separators, truncating or padding to target lengths as specified.

Metadata. For downstream labels we used (i) NCBI taxonomy (species/family), and (ii) Pfam domain family assignments from HMMER3 against Pfam 35.0 to create task-aligned labels used in Sections 3.8 and 3.9.

3.2 Training and Evaluation Pipeline Overview

Our approach involves three distinct stages, each employing different models (Figure 2):

Stage 1 – PPI Inference Model (ESM-2-PPI): Training protein language models on entire viral genomes is computationally demanding due to the quadratic scaling of attention mechanisms with sequence length. While existing sparse attention approaches can capture co-evolutionary signals, they typically use static or random masking patterns that neglect biological context. To overcome this, we develop a biologically informed sparse attention method that restricts attention computations exclusively to protein pairs identified as likely interaction partners. Ideally, experimental PPI databases would guide this identification; however, the limited coverage of viral genomes in these databases necessitated a computational strategy. Specifically, we fine-tune ESM-2 (650M parameters) with ALiBi positional encoding (selected after comparing against RoPE; see Supplementary Materials), LoRA adaptation, and Longformer-style sliding window attention (selected after comparing against Shifted Sparse Attention and BigBird; see Supplementary Materials). This extends the effective context window to approximately 13,000 aa, enabling analysis of multi-protein genomic fragments. This model is used exclusively for extracting attention scores to infer PPIs and is not employed for any downstream embedding or classification tasks. Section 3.3 describes the optimization of this model, and Section 4.1 presents the results.

Stage 2 – Genome-Scale Language Models (LV-3C and LV-5B): Using the PPIs identified in Stage 1 as biologically informed sparsity priors, we train two novel long-context protein language models. Both models use identical sparse attention patterns; they differ in scale and initialization:

- **LV-3C** (317M parameters; 16 layers): Trained from scratch on sequences up to 61,000 aa. Its lighter architecture prioritizes processing longer genomic context.

- **LV-5B** (756M parameters; 33 layers): Initialized from ESM-2 (650M) weights and trained on sequences up to 48,000 aa. Its larger architecture prioritizes richer pretrained representations.

This complementary design allows us to evaluate two strategies for incorporating genomic context: maximizing sequence length versus leveraging pretrained knowledge. These models represent the primary contribution of this work and are described in Sections 3.3–3.4.

Stage 3 – Baseline Models for Evaluation: To benchmark LV-3C and LV-5B, we compare against several ESM-2 variants:

- **ESM-2-pt**: The original pretrained ESM-2 model with a 1,024 aa context window.
- **ESM-2-RoPE**: ESM-2 fine-tuned with RoPE positional encoding for extended context inference.
- **ESM-2-ALiBi**: ESM-2 fine-tuned with ALiBi positional encoding for extended context inference.

These baselines isolate the contribution of our biologically informed sparse attention mechanism from general context extension strategies.

Throughout the manuscript, we use these standardized names to refer to each model variant. The following sections describe each stage in detail: Section 3.3 covers Stage 1 (PPI inference), Sections 3.4–3.5 cover Stage 2 (model training), and Sections 3.6–3.11 cover Stage 3 (evaluation).

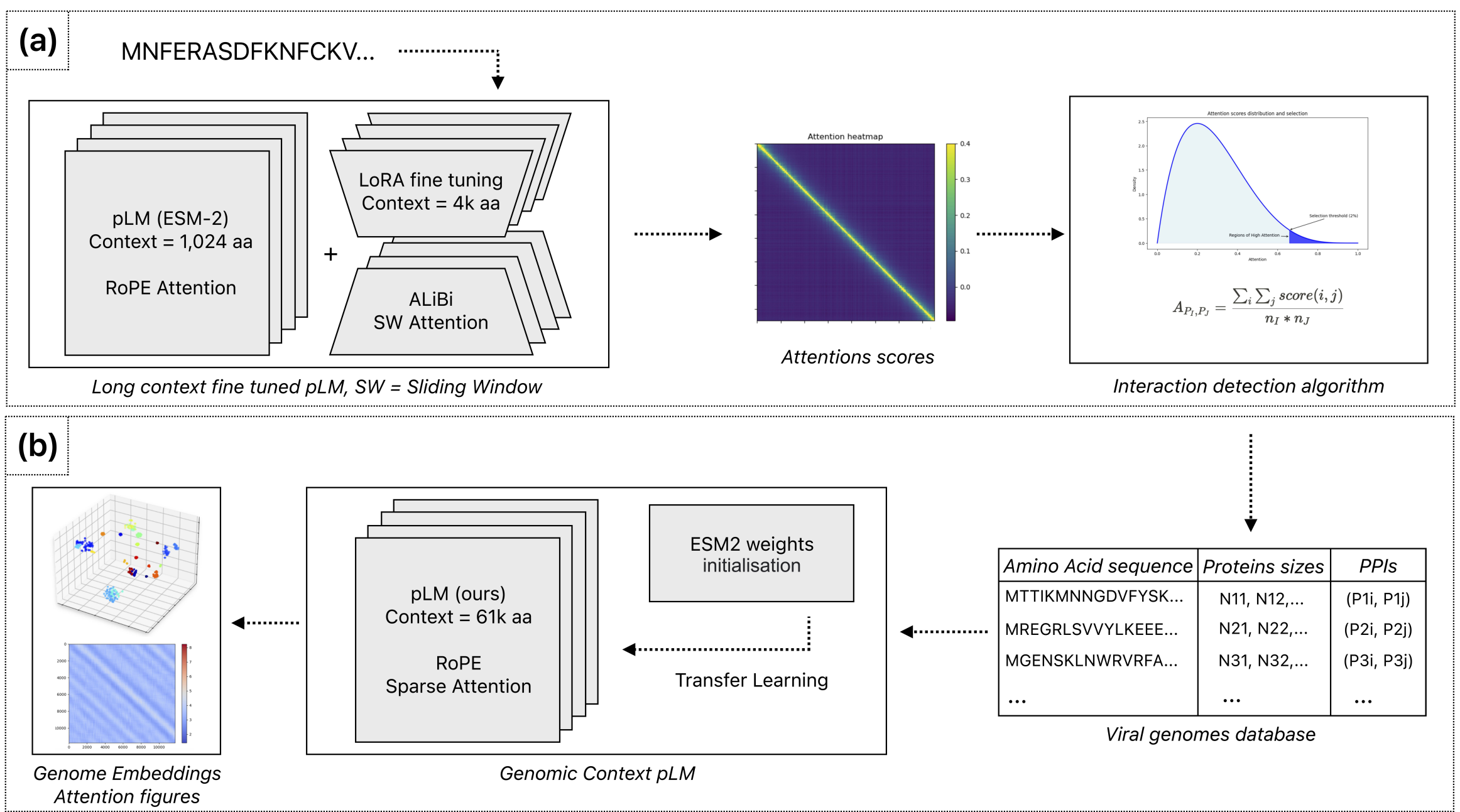

Figure 2: **Two-stage approach for training a protein language model on complete viral genomes.** **(a)** Identifying putative protein-protein interactions on multi-protein fragments using an extended-context ESM-2 model combining LoRA fine-tuning and ALiBi positional extrapolation. The attention scores produced by this model are subsequently used to inform the protein interaction detection algorithm. **(b)** Training of two long-context protein language models from scratch or initialized from ESM-2 weights using sparse attention patterns guided by inferred protein interactions, enabling efficient whole-genome embedding generation.

3.3 Developing the PPI Inference Model (Stage 1)

To extend ESM-2's context for PPI inference, we evaluated multiple approaches for achieving a context window of 13,000 aa (Supplementary Materials S2). Specifically, we compared two positional encoding strategies (RoPE and ALiBi) (Supplementary Figure S2) and three sparse attention mechanisms (Longformer, Shifted Sparse Attention, and

BigBird) (Supplementary Table S1). Performance was benchmarked against the baseline ESM-2 650M model using both perplexity, which quantifies how well the model predicts amino acid sequences (lower is better), and silhouette scores, which measure how well protein embeddings cluster by structural similarity (higher is better).

PPIs can be inferred from attention scores between proteins in language models, as demonstrated by previous work. For instance, [28] showed that specific attention heads in ESM-1b capture distinct protein relationships, and subsequent work extended these findings to larger models including ESM-2 [3]. Our approach builds upon this concept by analyzing attention patterns simultaneously across multiple proteins within viral genomes to rank putative interaction links. Standard protein language models, such as ESM-2, have a context length limitation of 1,024 aa, restricting analysis to only 3–5 average-length proteins at once. To overcome this, we first extended ESM-2's context window to 4,000 aa using attention matrix approximation, and subsequently increased it further to 13,000 aa through context length extrapolation (ALiBi). For the initial extension, ESM-2 was fine-tuned on concatenated protein fragments from the NCBI Viral database [14] using an NVIDIA A6000 GPU with 48 GB RAM. Protein fragments were constructed by sequentially concatenating complete proteins in their genomic order until reaching the 4,000 aa threshold, padding as necessary. To enable efficient training on standard GPU hardware, we used LoRA for parameter-efficient fine-tuning, while Longformer was selected as the sparse-attention pattern based on the comparisons in Supplementary Table S1.

Interaction Threshold Selection. A key parameter in our sparse attention mechanism is k, the number of top-ranked non-adjacent protein pairs included in the attention mask for each genome. To select an appropriate value, we varied $k \in \{10, 20, 35, 50, 100\}$ and evaluated downstream performance on taxonomy and Pfam classification tasks (Supplementary Materials S2). We also tested an alternative policy that includes genomically adjacent pairs in the ranking. Supplementary Table S2 shows that $k = 50$ maximizes F1 score with modest computational cost, while including adjacency during selection provides no benefit since consecutive protein attention is already explicitly included in the sparse pattern (see Section 3.4).

For each protein fragment analyzed by the fine-tuned model, attention matrices from all encoder layers were extracted. For each layer ℓ and attention head h, we denote the token-level attention matrix as $M^{(\ell,h)} \in \mathbb{R}^{L\times L}$ and use p, q, r, s for token positions. Each matrix was normalized and symmetrized using Average Product Correction (APC), yielding $N^{(\ell,h)}$ computed as [3]:

$$
\begin{aligned}
APC^{(\ell,h)}_{p,q} &= \frac{\sum_{s=1}^{L} M^{(\ell,h)}_{p,s} \, \sum_{r=1}^{L} M^{(\ell,h)}_{r,q}}{\sum_{r=1}^{L}\sum_{s=1}^{L} M^{(\ell,h)}_{r,s}}, \\
\widetilde{M}^{(\ell,h)}_{p,q} &= M^{(\ell,h)}_{p,q} - APC^{(\ell,h)}_{p,q}, \\
N^{(\ell,h)}_{p,q} &= \frac{1}{2}\left(\widetilde{M}^{(\ell,h)}_{p,q} + \widetilde{M}^{(\ell,h)}_{q,p}\right).
\end{aligned}
$$

Here, ℓ indexes layers, h indexes attention heads, and $p, q, r, s \in \{1, \ldots, L\}$ index amino-acid positions within the sequence.

From each normalized attention matrix $N^{(\ell,h)}$, we define a set $S^{(\ell,h)}$ containing the top $t_{\mathrm{aa}}\%$ highest attention values between amino-acid pairs. We used $t_{\mathrm{aa}} = 2$, corresponding to the top 2% of amino-acid attention values; this amino-acid-level threshold is separate from the final protein-pair threshold $k = 50$:

$$
S^{(\ell,h)} = \{(p, q) \mid N^{(\ell,h)}_{p,q} > \mathrm{Quantile}_{1-\frac{t_{\mathrm{aa}}}{100}}(N^{(\ell,h)})\}.
$$

Attention values within the set S were subsequently converted to scores via min-max normalization. These normalized scores were then aggregated to compute an attention-based interaction score A_{P_I,P_J} between protein pairs P_I and P_J, having lengths n_I and n_J, respectively:

$$
A_{P_I,P_J} = \frac{\sum_{p\in P_I}\sum_{q\in P_J} \mathrm{score}(p, q)}{n_I \times n_J} \quad \text{with} \quad (p, q) \in S^{(\ell,h)}.
$$

Interaction scores were computed for all protein pairs within the genome. Preliminary analyses indicated that genomically adjacent proteins consistently yielded the highest attention scores (Supplementary Figure S3), likely reflecting genuine biological interactions. Therefore, for constructing the sparse attention matrix used in subsequent model training, only the top 50 ranked scores from non-consecutive protein pairs were included as interacting proteins, with the remaining pairs masked. This threshold of 50 interactions represents a balance between computational efficiency

and interaction coverage appropriate for the viral genome sizes analyzed (Supplementary Figure S4). The threshold parameter can be adjusted based on genome sequence length and available computational resources.

To calibrate the number of protein pairs selected for sparse attention on biologically relevant data, we investigated interaction patterns among Family A DNA polymerase (PolA), ribonucleotide reductase (RNR), and helicase (HEL) (Supplementary Figures S3, S4, Supplementary Table S2). These proteins constitute a well-characterized interaction network [29] and display strong evolutionary coupling due to their coordinated roles in DNA replication [30, 31].

3.4 Sparse Attention Computation (Stage 2)

Once the PPIs have been identified and selected using the mechanism described above, it is possible to proceed with training a long-context pLM. Model training is based on a sparse attention mechanism, described below, and represented in Figure 3.

To maintain biological fidelity when computing cross-protein attention, we developed a two-stage fragmentation approach. First, we segment the viral genome precisely at protein boundaries, preserving each protein as a distinct unit. Second, we subdivide each protein into uniform blocks of fixed length B, resulting in a block-level representation of the viral genome. We then map the previously identified PPIs onto these blocks, creating fragment-level interaction pairs that guide our sparse attention model. This transformation preserves biologically meaningful relationships while enabling efficient computation across the entire viral genome.

Specifically, given two interacting proteins P_1 and P_2, we transfer these interactions to interactions between their constituent blocks:

$$(P_1, P_2) \Rightarrow (B_{1_i}, B_{2_j}), \quad i \in [1, n_1],\ j \in [1, n_2]$$

where:

- P_1, P_2 are two interacting proteins from genome G.
- $B_{1,i}$ is the i-th block of protein P_1, with n_1 total blocks.
- $B_{2,j}$ is the j-th block of protein P_2, with n_2 total blocks.

For each pair of blocks $(B_{1,i}, B_{2,j})$, we compute the block-level attention scores using the scaled dot-product attention formula:

$$Att_G(B_{1,i}, B_{2,j}) = \frac{Q_{B_{1,i}} K^T_{B_{2,j}}}{\sqrt{d_k}}$$

where:

- $B_{1,i}$, $B_{2,j}$ are the blocks from genome G being considered
- $Q_{B_{1,i}}$ is the query vector subset from genome G corresponding to block $B_{1,i}$.
- $K_{B_{2,j}}$ is the key vector subset from genome G corresponding to block $B_{2,j}$.
- d_k is the dimensionality of the key vectors.

Efficient computation of sparse attention requires specialized data structures optimized for block operations. We utilize the Block Sparse Row (BSR) tensor format [32], which stores non-zero elements in fixed-size blocks, significantly reducing both memory consumption and computational overhead. Empirical testing identified a block size $B = 32$ as optimal, balancing computational efficiency and minimal padding overhead in our viral protein datasets.

In our implementation, each query–key product $Q_{F_i}(K_{F_j})^T$ between fragment pairs $B_{1,i}$ and $B_{2,j}$ produces a 32×32 tensor, directly aligning with BSR's block structure. We subsequently apply softmax normalization followed by multiplication with the corresponding value vectors using optimized Triton [19] kernels tailored for block-sparse operations. Finally, we remove padding tokens to restore original sequence dimensions using precomputed position indices (Algorithm 1).

3.5 Model Training (Stage 2)

The training was conducted using the NCBI Virus database (retrieved on 06/2024), comprising 14,436 complete viral genomes totaling 683 million aa (Supplementary Figure S1). The database represents a comprehensive reference set of viral diversity, ensuring robust generalization across viral families. All genomes were segmented at protein boundaries

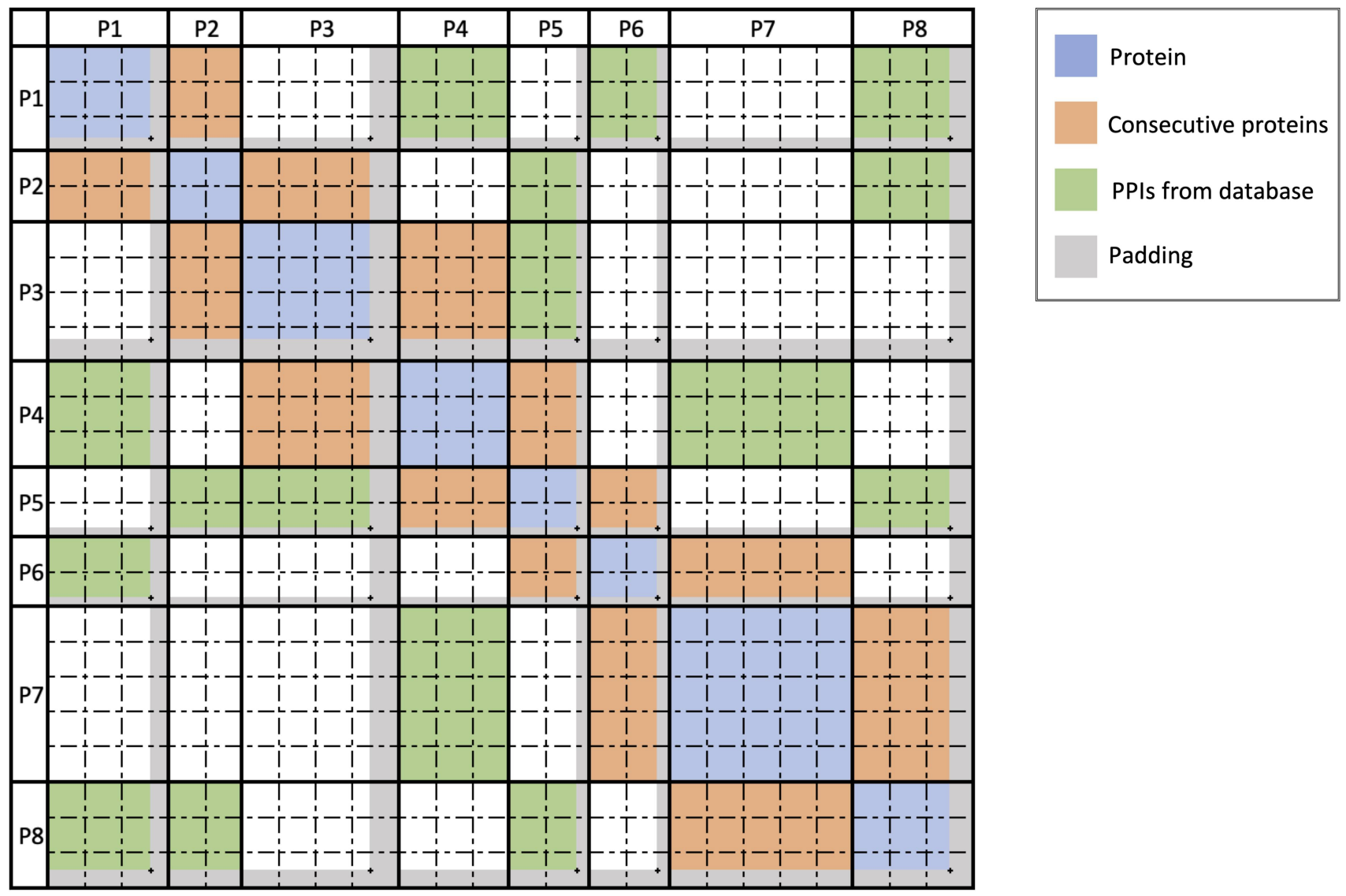

Figure 3: **Sparse attention computation architecture.** Example attention matrix demonstrating four distinct protein interaction patterns considered during sparse attention computation: intra-protein (self) attention (blue), interactions between consecutive proteins (orange), putative protein–protein interactions identified by our method (Section 3.3) and stored in a database (green), and protein pairs not identified as interacting (white), for which attention calculations are omitted. Gray regions indicate padding introduced during fragment construction.

and padded appropriately to meet sequence length constraints. All training was conducted using NVIDIA A6000 GPUs (48 GB RAM) with block-sparse computations optimized through the Triton library [19].

The long-context protein language models represent two main variants, designed to produce complementary types of embeddings. These two models differ in scale, with LV-3C being the lighter version (317M parameters) and LV-5B the larger one (756M parameters).

The two models also differ in their initialization and training strategies:

- LV-3C: Randomly initialized and trained from scratch on genomic segments (up to 61,000 amino acids) with an initial learning rate of 3×10^{-4}, using biologically informed sparse attention for 12 epochs.
- LV-5B: Trained leveraging transfer learning from the ESM-2 model (650M parameters) on genomic segments (up to 48,000 amino acids), using the same biologically informed sparse attention with an initial learning rate of 3×10^{-5}, also for 12 epochs.

This two-model design aims to capture complementary aspects of viral genomic information. LV-3C prioritizes the modeling of long-range genomic context through a lighter architecture that enables the processing of extended sequences, whereas LV-5B prioritizes richer pretrained representations through a larger model operating on shorter segments.

3.6 Evaluation Metrics (Stage 3)

We employ two primary metrics throughout our evaluations:

Perplexity measures how well the model predicts amino acid sequences, quantifying the model's uncertainty when predicting masked tokens. It is defined as the exponentiated average cross-entropy loss:

$$\text{Perplexity} = \exp\left(-\frac{1}{N}\sum_{i=1}^{N}\log P(x_i|\text{context})\right)$$

where N is the number of masked tokens and $P(x_i|\text{context})$ is the predicted probability of the true amino acid x_i. Lower perplexity indicates better predictive performance. This metric is particularly important for assessing whether models maintain consistent prediction quality as sequence length increases.

Algorithm 1 Algorithm for computing biologically informed sparse attention, from input tensors through block-wise operations to final output.

Entries: Queries, Keys, and Values: $Q, K, V \in \mathbb{R}^{n\times d}$
Protein interactions $P_I \in \mathbb{R}^{i\times 2}$, Protein sizes $P_S \in \mathbb{R}^N$

$Q, K, V \leftarrow$ reshape_tensor_padding(Q, K, V, P_S, B) ▷ Split genome into proteins, apply padding if necessary, then into B-size blocks: $X(bs, n_b, B, d) \leftarrow X(bs, n, d)$

$Q, K, V \leftarrow$ transpose_for_scores(Q, K, V, n_h, h_s) ▷ Transpose tensors for attention computation: $X(bs, n_h, h_s, n_b, B) \leftarrow X(bs, n_b, B, d)$

$BI \leftarrow$ chunk_proteins_padding(PI, PS) ▷ Transform protein interactions into block interactions

$Q, K \leftarrow$ rotary_embeddings(Q, K) ▷ Apply rotary positional embeddings

$Att \leftarrow$ sparse_attention_matrix(Q, K, BI) ▷ Compute attention scores and construct BSR tensor
$Att \leftarrow$ bsr_softmax(Att)
$Att \leftarrow$ bsr_dropout$(Att,$ dropout$)$
$V \leftarrow$ bsr_dense_mm(Att, V)

$V \leftarrow$ revert_padding(V) ▷ Reverse padding and reshape into final dimensions : $V(bs, n, d) \leftarrow V(bs, n_b, B, d)$

return V

Silhouette score evaluates embedding quality by measuring clustering coherence. For a given embedding i with cluster label a, the silhouette score is:

$$s(i) = \frac{b(i) - a(i)}{\max(a(i), b(i))}$$

where $a(i)$ is the mean distance to other points in the same cluster and $b(i)$ is the mean distance to points in the nearest neighboring cluster. Scores range from -1 to 1, where higher values indicate better-defined clusters. We use this metric to assess whether embeddings group biologically related sequences (e.g., by sequence similarity or taxonomic classification).

3.7 Evaluating Global Performance & Protein Embeddings Quality

We evaluated our biologically informed sparse attention models (LV-3C and LV-5B) against ESM-2 pre-trained (ESM-2-pt) and ALiBi fine-tuned variant (ESM-2-ALiBi) using perplexity and clustering silhouette scores. Perplexity assessed token prediction accuracy over extended contexts, while the silhouette scores evaluated the quality and information content of protein sequence embeddings. We assessed model performance using viral genome fragments ranging from 500 to 20,000 aa. Although our models support sequences up to 61,000 aa, we limited evaluations to 20,000 aa to accommodate the computational constraints (time and memory) associated with full attention when running on a single GPU (NVIDIA A6000 with 48 GB of RAM).

Sequence-based clustering reference. To evaluate whether model embeddings preserve sequence relationships, we clustered proteins using MMseqs2 at 80% sequence identity. These clusters serve as reference labels for computing silhouette scores: high silhouette scores indicate that proteins similar in sequence are also proximal in embedding space. This provides a baseline assessment of embedding quality independent of functional or taxonomic annotations.

3.8 Evaluating Genome Embeddings Quality

We assessed the quality of genome-scale embeddings produced by our models using taxonomic clustering and species classification. To further evaluate embedding utility, we trained a single-layer classifier to identify species from genome embeddings (details provided in Supplementary Material S3).

3.9 Evaluation of Embedding Quality for Classification

We evaluated the models' contextual embeddings by assessing their performance on species clustering and classification tasks (see also Supplementary Material S3; Pfam/domain-family validation is described separately in Supplementary Material S4), as well as by analyzing the similarity distributions obtained from comparing pairs of embeddings.

- **Evaluation of taxonomic information preservation:** To evaluate how effectively different models preserve taxonomic information, we analyzed clustering patterns of protein embeddings derived from 100 distinct viral species.
- **Analysis of species clustering efficiency:** Next, we trained a single-layer taxonomic classifier designed to predict the species identity of individual proteins directly from their embeddings. To systematically assess classification performance, we varied the number of species classes from 5 up to 100 species. We measured classifier performance using F1-score averaged over a 5-fold cross-validation scheme.
- **Analysis of embedding variance and distribution:** To further characterize the differences among embedding spaces produced by various models, we randomly selected 5,000 proteins from over 1,000 viral genomes and computed pairwise Euclidean distances between their embeddings. We then compared the resulting distributions from embeddings generated by ESM-2 variants and our two proposed models, LV-5B and LV-3C.

3.10 Evaluating Across-Model Attention

The following two sections evaluate the attention patterns of the trained LV-3C and LV-5B models, as distinct from the Stage 1 PPI inference used to build the sparse-attention priors: they assess whether the trained models—having been guided by these priors during training—retain and recapitulate biologically meaningful PPI signal in their final attention. To this end, we analyzed attention patterns across the entire network architecture. Attention scores from all heads in each layer were extracted to examine each model's ability to detect and represent interactions spanning various distances within the genome sequence.

We categorized attention interactions into three distinct proximity classes:

- **Short-range interactions**: Attention between amino acids positioned within 100 residues of each other, typically representing intra-protein or intra-domain relationships.
- **Medium-range interactions**: Attention between amino acids separated by 100–500 residues, generally capturing interactions between domains or adjacent proteins.
- **Long-range interactions**: Attention between amino acids separated by more than 500 residues, predominantly representing inter-protein interactions between non-adjacent proteins.

For each category, we calculated the mean attention score across multiple viral genomes, enabling comprehensive analysis of the model's attention distribution patterns.

3.11 Validation Against Experimentally Curated Interactions

To validate the biological relevance of model-derived attention patterns, we benchmarked attention-derived protein-pair scores, interpreted as putative interaction links, against experimentally validated interactions documented in the STRING database [33]. Attention scores were extracted from all heads and layers, and protein pairs were ranked by their aggregate long-range attention score following [28]. Validation encompassed 146 annotated interactions across five distinct viral genomes. STRING interactions with confidence ≥ 0.500 served as positive examples; negative examples consisted of all other protein pairs from the same genomes not reported as interacting in STRING. We note that this negative set likely contains some true interactions not yet experimentally characterized, making our reported metrics conservative estimates.

4 Results

4.1 Developing the PPI Inference Model (Stage 1)

Before training our genome-scale models LV-3C and LV-5B, we first developed ESM-2-PPI, a separate auxiliary model used exclusively to derive the protein–protein interaction priors that guide the sparse attention mechanism. ESM-2-PPI extends pretrained ESM-2 (650M) to a 13,000 aa context window via LoRA fine-tuning, sparse attention, and ALiBi positional extrapolation. Because the choice of positional encoding and sparse-attention pattern directly affects the

reliability of the attention scores subsequently used as priors, we evaluated two positional encoding strategies (RoPE, ALiBi) (Supplementary Figure S2) and three sparse attention mechanisms (Longformer, Shifted Sparse Attention, BigBird) (Supplementary Table S1) on held-out viral genome fragments ranging from 500 to 13,000 aa, requiring both stable perplexity across extended sequence lengths and embeddings that preserve biological relationships. ALiBi with Longformer attention achieved the best balance of perplexity stability (1.99 across all tested lengths), clustering quality (silhouette score: 0.88), and computational efficiency.

To validate that ESM-2-PPI captures biologically meaningful interactions, we analyzed attention patterns among Family A DNA polymerase (PolA), ribonucleotide reductase (RNR), and helicase (HEL)—proteins with well-characterized co-evolutionary relationships [29, 30, 31]. These known interacting pairs consistently ranked among the top 50 non-adjacent protein pairs across 83 viral genomes (Supplementary Figures S3, S4), supporting our selection of $k = 50$ as the interaction threshold.

This validation serves only to calibrate the PPI selection threshold for ESM-2-PPI and the PolA/RNR/HEL interactions were not used during training of LV-3C or LV-5B, which learn from PPIs inferred independently across all genomes in the training set.

4.2 Evaluating Global Performance & Embeddings Quality

This section evaluates our two genome-scale protein language models LV-3C (317M parameters, trained from scratch on sequences up to 61,000 aa) and LV-5B (756M parameters, initialized from ESM-2 weights and trained on sequences up to 48,000 aa) against three ESM-2 baselines (ESM-2-pt, ESM-2-RoPE, ESM-2-ALiBi). Both LV models share the same biologically informed sparse attention mechanism and differ only in scale and initialization, allowing us to disentangle the contribution of context length from that of pretrained knowledge.

The LV-3C and LV-5B models demonstrated significantly lower perplexity (uncertainty when predicting masked tokens) compared to ESM-2 variants on sequences longer than 3,000 aa (Figure 4a), maintaining this advantage consistently across all tested lengths. In contrast, both the pre-trained ESM-2-pt (gray) and its RoPE-fine-tuned variant ESM-2-RoPE (red) experienced significant perplexity inflation when evaluated beyond their original training context windows. Fine-tuned version of ESM-2-ALiBi (orange) maintains near-constant perplexity with sequence length, but higher than our models.

For clustering quality analysis, we clustered protein sequences and fragments using MMseqs2 [34] at an 80% similarity threshold and computed silhouette scores to quantify embedding coherence. Our results revealed clear differences in clustering performance among the tested models (Figure 4b).

Both LV models provided superior embedding coherence and better discrimination of protein sequences compared to baseline approaches. LV-3C (light blue) exhibited robust clustering quality, particularly for longer sequences (silhouette score: 0.83), while LV-5B (dark blue) achieved performance comparable to ESM-2-pt and ESM-2-RoPE for shorter sequences and maintained high effectiveness at extended lengths (silhouette score: 0.67). ESM-2-pt (gray) excelled only within its original training range (1,024 aa), with embedding quality markedly declining for longer, multi-protein sequences. While ESM-2-ALiBi (orange) extended effective clustering performance up to approximately 4,000 aa, embedding quality still deteriorated as sequence lengths increased, despite the positional encoding extrapolation strategy.

4.3 Evaluating Genome Embeddings Quality

The quality of the genomic embeddings was assessed by downstream performance in a clustering and a classification task. We employ two complementary evaluations: silhouette scores assess embedding coherence, whether proteins from the same taxonomic species cluster together in latent space, while classification accuracy assesses whether embeddings encode sufficient discriminative information for species prediction. These metrics capture related but distinct properties: coherent embeddings facilitate classification, but similar classification performance can arise from different embedding geometries. Silhouette scores (Figure 5a) indicated strong taxonomic discrimination across all models, with the LV-5B variant consistently outperforming others.

The performance of the species classification of genomes evaluated by F1-scores (Figure 5b) revealed a notable trend: as the number of species increased—and consequently, task complexity—embeddings generated using biologically informed sparse attention demonstrated superior classification accuracy relative to genome embeddings from fine-tuned ESM-2-ALiBi. This performance improvement at higher taxonomic resolution suggests our embeddings effectively capture nuanced genomic features, enhancing discriminative power at larger phylogenetic scales. These properties hold promising implications for phylogenetic and metagenomic applications.

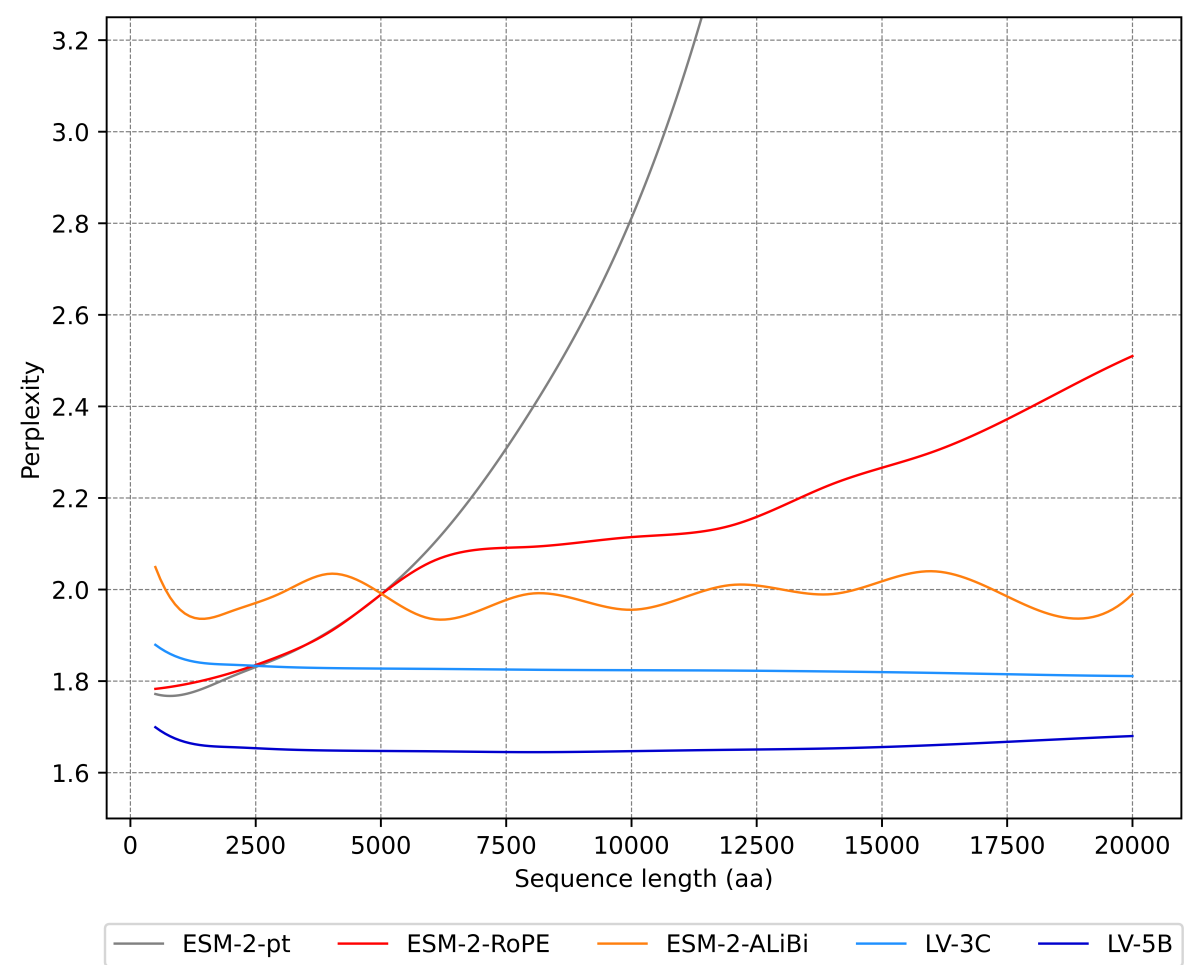

(a) Perplexity

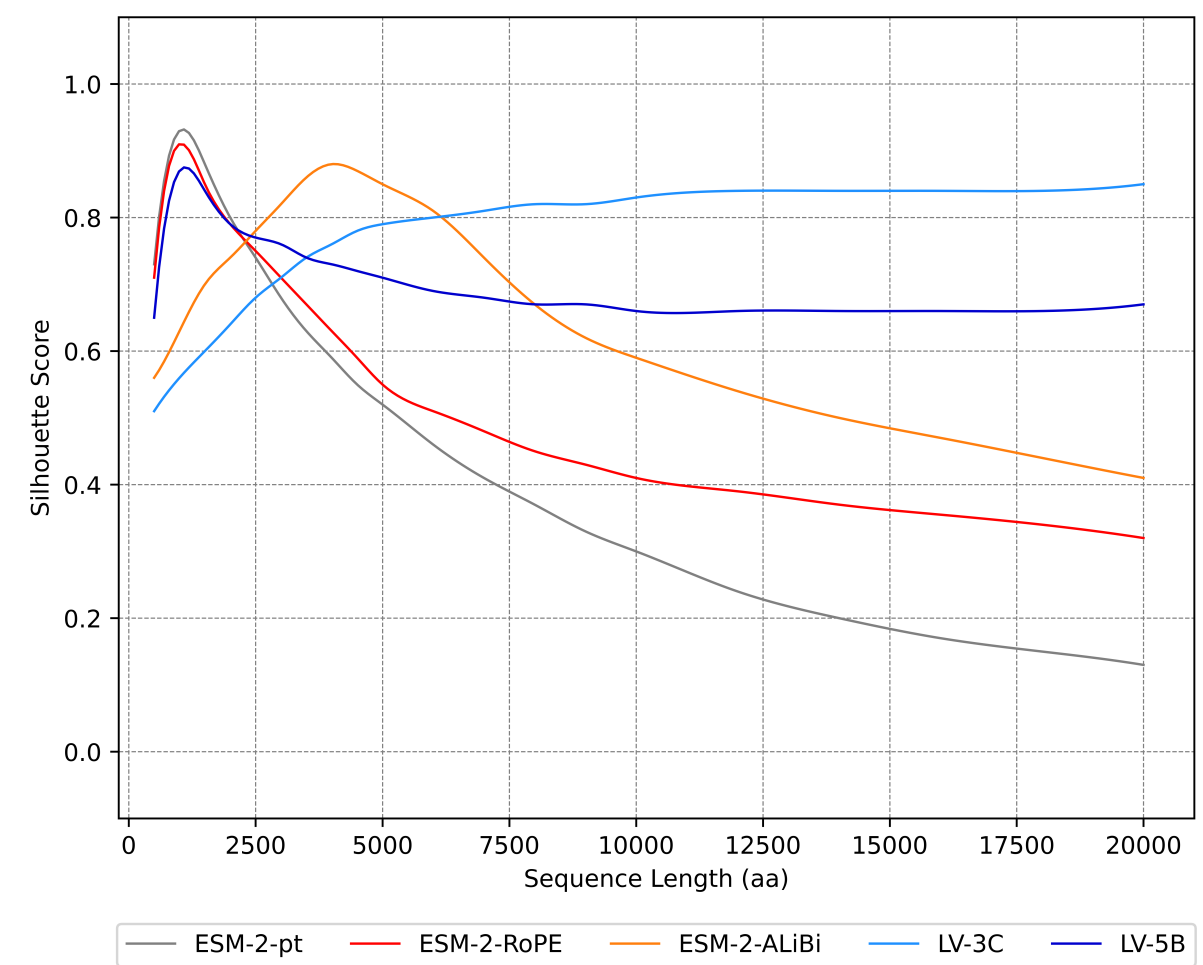

(b) Silhouette Score

Figure 4: **Global performances of our models compared to ESM-2 variant baselines. (a)** Perplexity evaluation of ESM-2-pt, ESM-2-RoPE, ESM-2-ALiBi, and our biologically informed sparse attention models LV-3C & LV-5B. **(b)** Clustering ability evaluation using silhouette score on fragment embeddings, using sequence similarity as reference with MMSeqs2 80% amino acid identity clustering. These smoothed curves are obtained from perplexity and silhouette score measurements for sequences ranging from 500 to 20,000 amino acids.

4.4 Evaluation of Protein Embedding Quality for Classification

4.4.1 Analysis of Embedding Variance and Distribution

The quality of protein embeddings generated by ESM-2-ALiBi and proposed models LV-3C and LV-5B were assessed based on embedding variance and distribution of the pairwise Euclidean distances between 5,000 randomly selected protein from over 1,000 viral genomes. The analysis (Figure 6) revealed distinct variability patterns among these embedding spaces. Embeddings produced by ESM-2-ALiBi demonstrated the narrowest distribution, with the lowest interquartile range (1.16) and a relatively limited embedding space extent (range: 9.43). In contrast, embeddings from model LV-5B showed greater variability, with a broader interquartile range (1.58) and a larger embedding space (range: 15.69). Model LV-3C exhibited the most dispersed embeddings, spanning the widest embedding space (range: 16.18) and displaying significantly higher average pairwise Euclidean distances. These results indicate that incorporating genomic context into LV-3C substantially diversifies embedding representations, likely capturing biologically meaningful, genome-specific information.

Further analysis of the Euclidean distance across protein embeddings shows that context provides a stronger correlation with taxonomic distance (Figures 6, 7). Specifically, embeddings from proteins within the same species exhibit high similarity, characterized by low Euclidean distance. As the taxonomic distance between proteins increases, embedding similarity correspondingly decreases, highlighting the models' ability to capture biologically meaningful divergence across broader taxonomic categories.

4.4.2 Evaluation of Taxonomic Information Preservation

Given that protein embeddings inherently cluster primarily by protein type rather than species, silhouette scores based on taxonomy labels across all models were generally low (slightly negative). Despite this challenge, embeddings generated by the LV-3C model exhibited better species-specific clustering relative to those produced by ESM-2-pt and LV-5B (Figure 7a, Pfam-specific clustering in Supplementary Material S4 and Supplementary Figure S5). For comparison, we included embeddings from the Protein Set Transformer (PST), a recent genome-level protein model, as an additional baseline. We note that PST's public release (v2.1.0) does not support genome-level embeddings, and its graph neural network architecture does not produce attention scores comparable to our transformer-based approach; PST is therefore included only in protein embedding evaluations where outputs are directly comparable. PST achieved the lowest silhouette scores, indicating limited capability in capturing viral species-level taxonomic relationships.

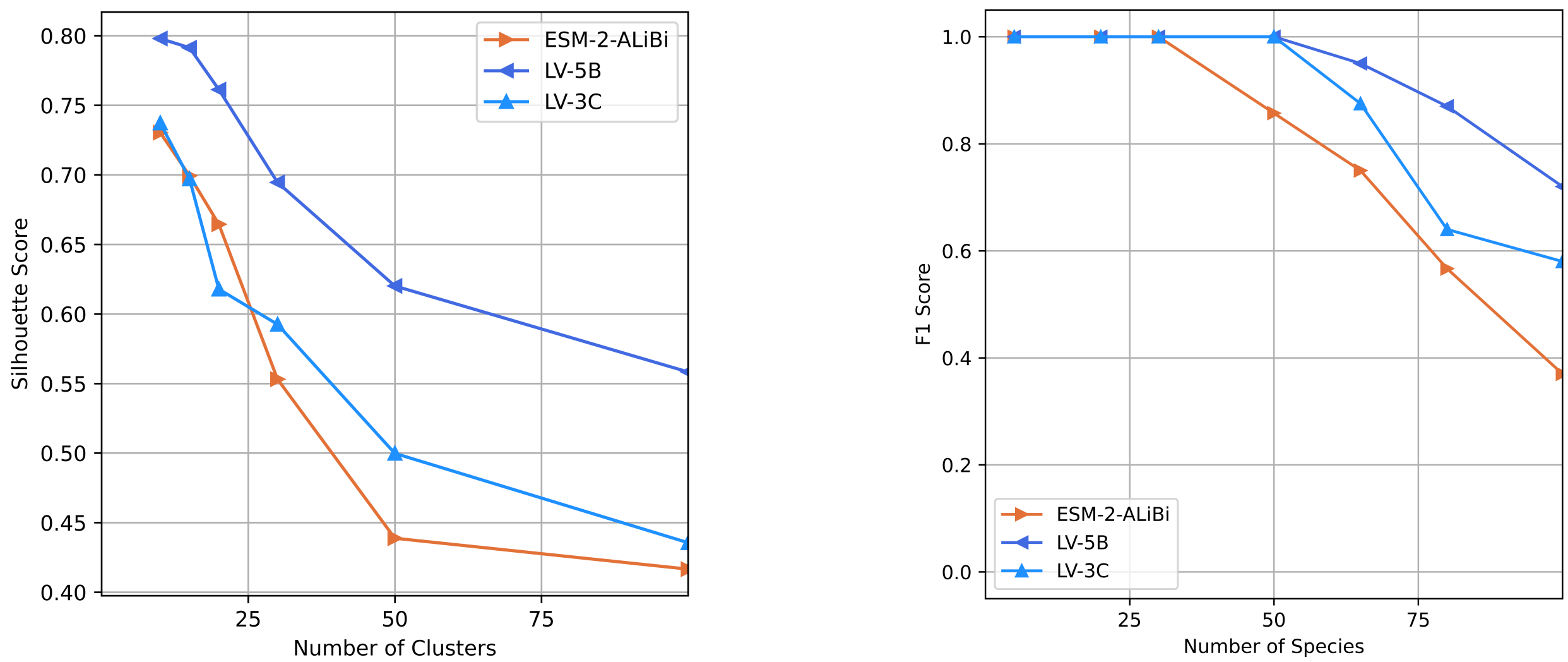

(a) Silhouette Score

(b) Classification

Figure 5: **Evaluation of full genome embeddings. (a)** Silhouette scores comparing genome embeddings generated by LV-3C, LV-5B, and ESM-2-ALiBi, using taxonomic species as cluster labels. **(b)** F1-scores for a single-layer classifier predicting species identity from genome embeddings (LV-3C, LV-5B, ESM-2-ALiBi). The x-axis varies the number of viral species used as classification targets, from 5 to 100; larger values denote finer-grained taxonomic resolution.

4.4.3 Analysis of Species Clustering Efficiency

As illustrated in Figure 7b, classification performance was the lowest when utilizing context-independent embeddings generated by the pre-trained ESM-2-pt. PST embeddings improved classification outcomes slightly, affirming that PST does capture genome-level context to some degree. However, LV-3C and LV-5B embeddings enriched with extensive genomic context further enhanced classifier accuracy across all evaluated class sizes. Among the tested models, LV-3C consistently achieved superior performance, reflected in the highest F1-scores for every class configuration.

4.5 Evaluating Across-Model Attention

Our proposed models were evaluated based on mean attention scores in short-range (within 100 aa, representing intra-protein or intra-domain relationships), mid-range (within 100-500 aa, representing inter-domain or adjacent protein relationships), and long-range ($>$ 500 aa apart, representing inter-protein interactions between non-adjacent proteins) interactions. The distribution of normalized attention scores across the three ranges provides insight into the model's capacity to capture biologically relevant interactions at different genomic scales. High variance in the attention score distribution indicates preferential focus on specific interaction ranges, while more uniform distributions suggest balanced attention across all distance categories. This analysis is particularly valuable for assessing whether the model effectively captures the long-range dependencies that conventional protein language models typically miss due to context limitations.

The attention profiles (short-, medium-, and long-distance) differ substantially among the four models evaluated (Figure 8a). The pre-trained ESM-2-pt model exhibits a highly localized attention mechanism, concentrating almost exclusively on short-range interactions (100%), typically within individual proteins or immediate neighboring residues. This pattern aligns with ESM-2's training paradigm, which focused on single-protein contexts and thus developed representations primarily capturing local residue relationships.

In contrast, the ESM-2-ALiBi fine-tuned model, which employs a sliding-window (LongFormer) attention approach, demonstrates a modest expansion in attention scope. While it identifies a small fraction of medium-distance interactions (3.12%), it still predominantly focuses on short-distance interactions (96.88%). These findings suggest that although the sliding-window methodology enables detection of certain medium-range relationships, the model largely remains constrained to interactions between proximal amino acids.

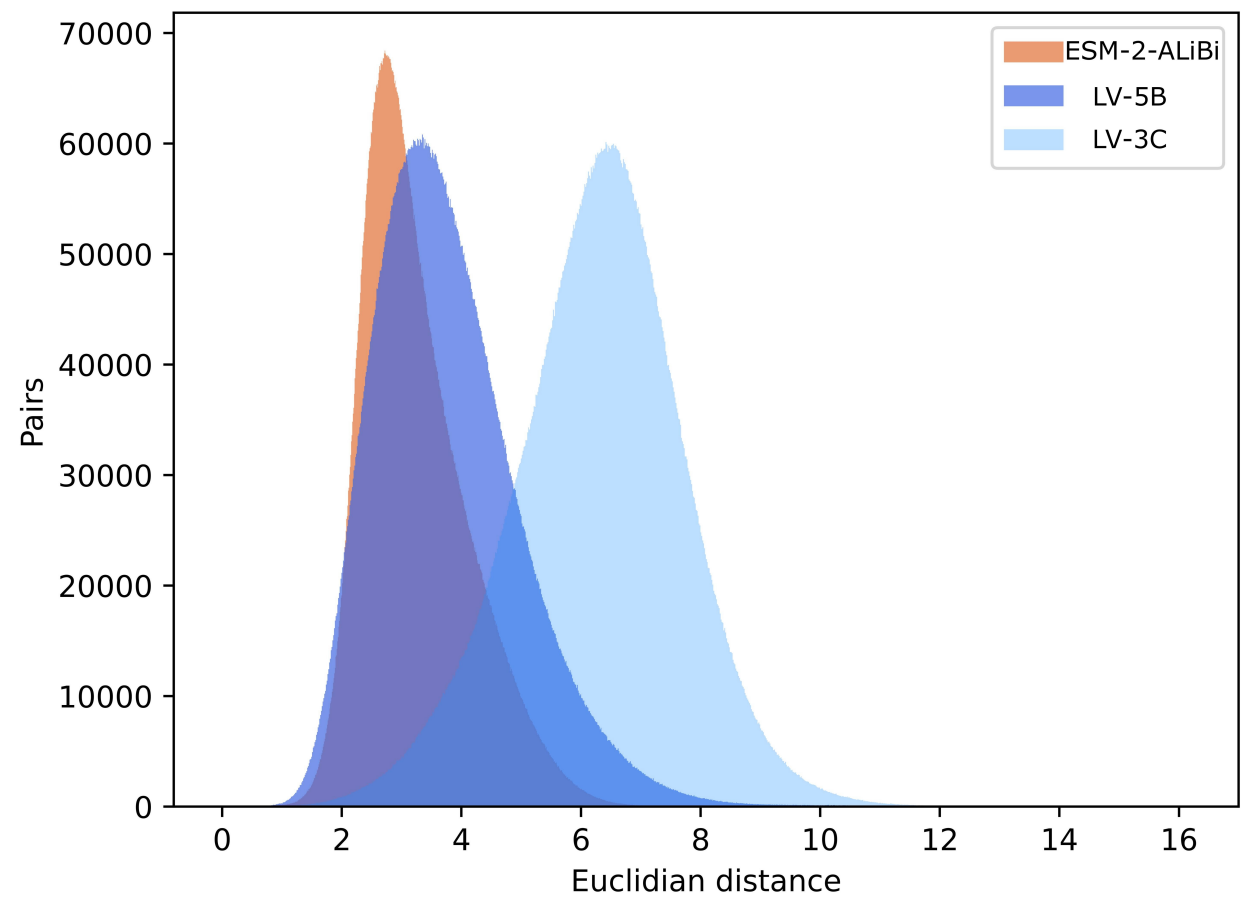

Figure 6: **Protein embeddings pairwise Euclidean distance.** Each curve summarizes ≈12.5 million pair comparisons: lower distances (left tail) indicate within-species similarity, while greater distances reflect increasing taxonomic separation. ESM-2-ALiBi yields the most compact manifold, LV-5B broadens the range, and LV-3C further widens and right-shifts the distribution, demonstrating its larger dynamic range for distinguishing distantly related proteins without sacrificing tight clustering of closely related sequences.

Our models, LV-3C and LV-5B, exhibit markedly different attention distributions. Both models display balanced average attention scores across all distance categories (short, medium, and long), indicating a comprehensive approach to protein sequence modeling that equally integrates local and global contextual information. However, important distinctions between the two models emerge:

- Model LV-3C most frequently attributes maximal attention to long-distance interactions. This behavior highlights its enhanced capability to capture meaningful relationships between amino acids widely separated in sequence space, potentially spanning different proteins within the viral genome.
- Model LV-5B, while maintaining generally balanced average scores, frequently assigns highest attention importance to both short- and long-distance interactions. This indicates a flexible modeling strategy capable of emphasizing both local structural information and global protein–protein relationships as needed. Notably, LV-5B's attention distribution retains traces of its ESM-2-pt initialization, suggesting it effectively combines characteristics of the pre-trained model with adaptations necessary for the expanded genomic context.

To further investigate inter-protein interactions, we calculated attention scores and identified attention heads specifically enriched for long-range information, following the methodology described in Section 3.11.

In a binary classification framework, using a STRING interaction confidence threshold of 0.500, our LV-3C model achieved an average F1 score of 0.74 and accuracy of 0.88. The classifier demonstrated an Area Under the Curve (AUC) of 0.66 (Figure 9, with a bootstrapped 95% confidence interval of [0.56, 0.75]), indicating statistically significant predictive performance. The difference between F1 (0.74) and AUC (0.66) reflects that F1 is measured at an optimized decision threshold, while AUC captures ranking performance across all thresholds—a more challenging metric given the inherent noise in attention scores and the known incompleteness of viral protein interaction databases. These results show significant concordance between our attention-based interaction scores and experimentally validated interactions in the STRING database, indicating that the method enriches for putative protein interactions within viral genomes. Furthermore, attention-derived scores showed significant positive correlation with STRING confidence scores (Spearman $\rho = 0.33$, $p < 0.001$), providing additional evidence for the biological relevance of the learned attention patterns.

5 Discussion

We note that attention-derived inter-protein links should be interpreted as computational priors rather than direct evidence of physical PPIs. For this reason, our evaluation strategy focused on language-model metrics, taxonomy/Pfam-

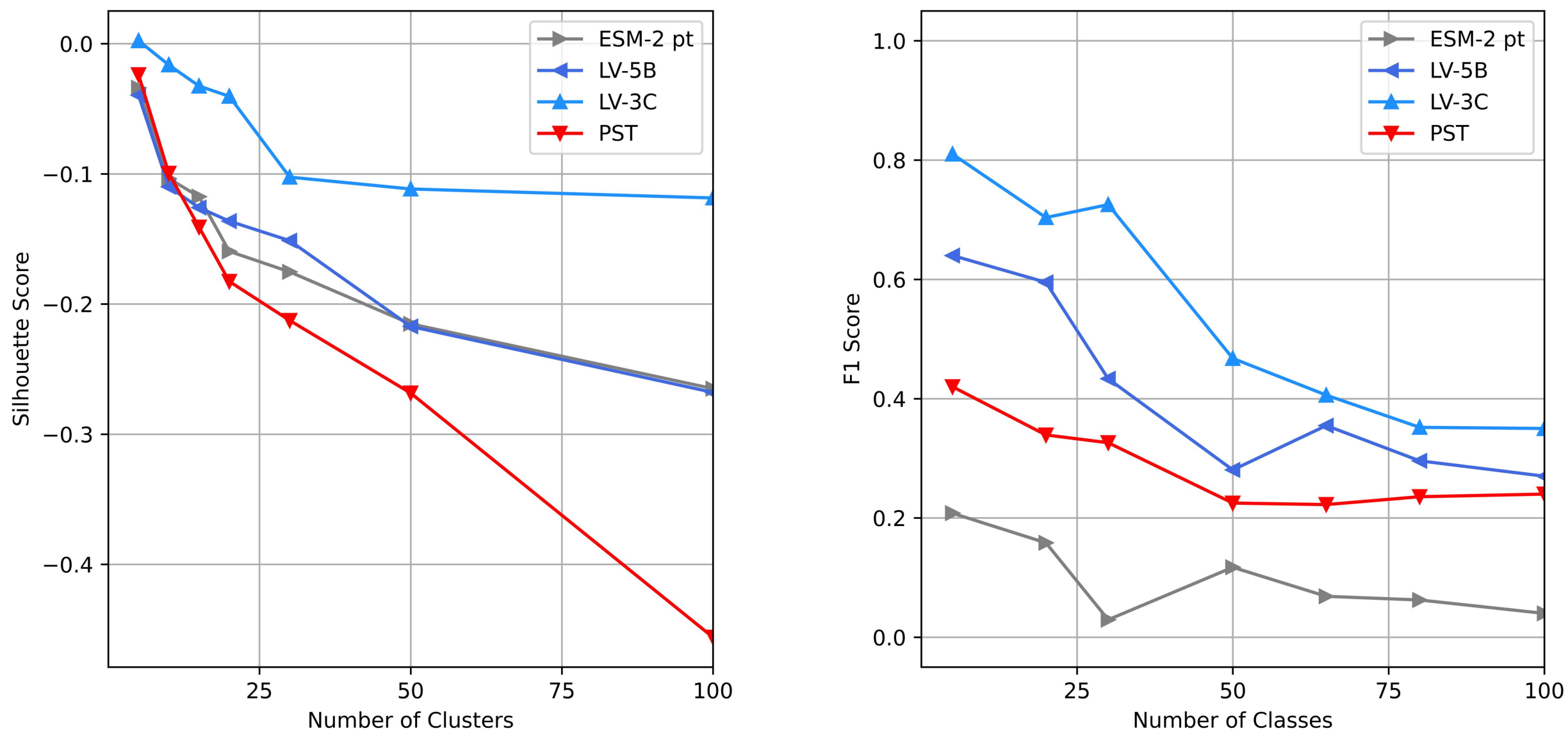

(a) Silhouette Score

(b) Classification

Figure 7: **Evaluation of contextualized protein embeddings. (a)** Silhouette scores comparing protein embeddings generated by LV-3C, LV-5B, Protein Set Transformer (PST), and ESM-2-pt, using taxonomic species as cluster labels. **(b)** F1-scores for a single-layer classifier predicting species identity from protein embeddings (LV-3C, LV-5B, PST, ESM-2-pt). The x-axis varies the number of viral species used as classification targets, from 5 (a coarse 5-way task) to 100 (a fine-grained 100-way task); larger values denote harder, more taxonomically resolved classification.

aligned downstream tasks, and concordance with independent interaction resources (STRING), rather than claiming direct prediction of physical interactions.

Our findings demonstrate that extending transformer-based language models to encompass entire viral proteomes yields substantial improvements in both local amino acid prediction and broader protein function inference. By designing a biologically informed and content-aware sparse attention mechanism guided by inter-protein co-evolutionary signals, we have addressed the long-standing challenge of modeling densely packed viral genomes while maintaining computational tractability.

A central insight from our study is that higher-order genomic context—the collective presence and arrangement of multiple proteins—significantly enhances protein representation accuracy beyond what can be achieved by analyzing protein sequences in isolation [3], or using context length extension [15, 16] or context-agnostic sparse attention [20, 21, 22]. As the results show, conventional protein language models trained on individual proteins (e.g., ESM-2) focus on local sequence features and exhibit marked performance degradation when context extends beyond their trained window. In contrast, our biologically informed sparse attention approach captures both local relationships and long-range dependencies across entire viral genomes. This more comprehensive perspective reveals functional couplings between non-consecutive proteins that would otherwise remain undetected [29, 30, 31]. To confirm that this advantage arises from the inference step rather than from simply adding long-range links, we trained otherwise identical models given inferred, random, distance-matched, or no long-range links. Only the inferred links lowered held-out perplexity, and the improvement concentrated on the protein pairs the inference model scored most highly (Supplementary Section S6).

The attention patterns learned by our models show statistically significant concordance with experimentally validated protein–protein interactions, benchmarked against the STRING database [33]. The balanced distribution of attention across short, medium, and long-range interactions in our LV-3C and LV-5B models, in contrast to the predominantly local focus of the ESM-2 baselines, further reinforces the biological relevance of our approach.

The significant divergence in embedding spaces between context-independent (other models) and the context-aware LV-3C and LV-5B models, as evidenced by the distinct distributions of pairwise Euclidean distances between protein embeddings, underscores the fundamental difference in information captured by these approaches. The wider embedding space of our LV-3C model, with its greater interquartile range and broader distribution of distances, suggests a richer representation that incorporates both sequence-specific and context-dependent information.

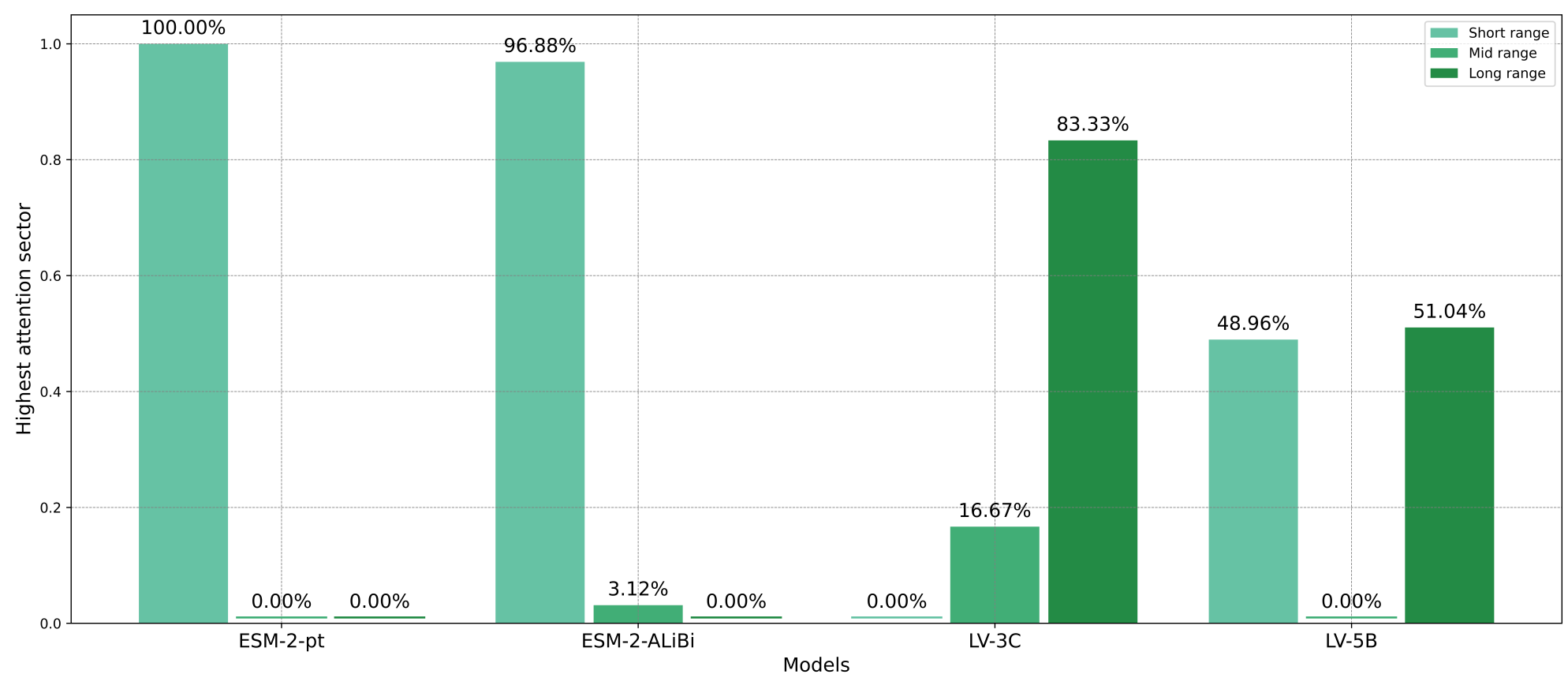

(a)

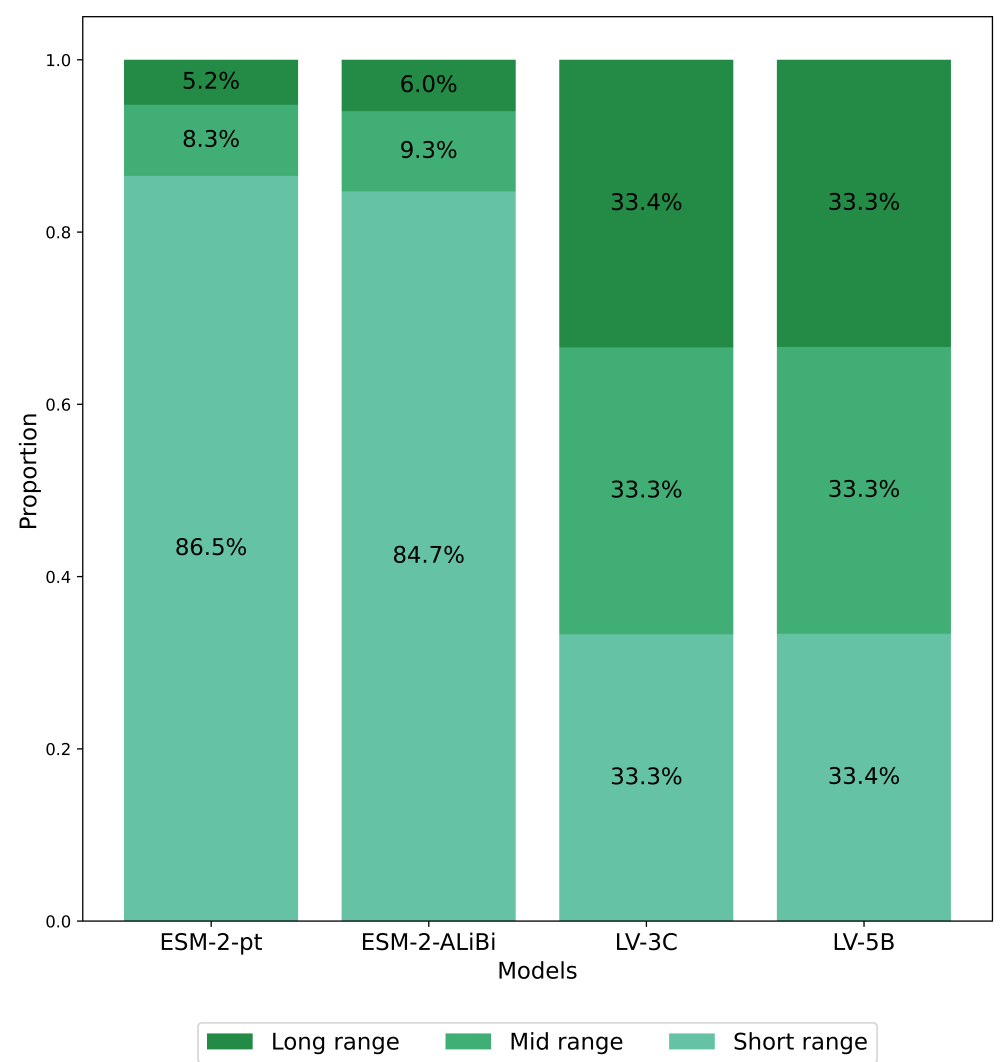

(b)

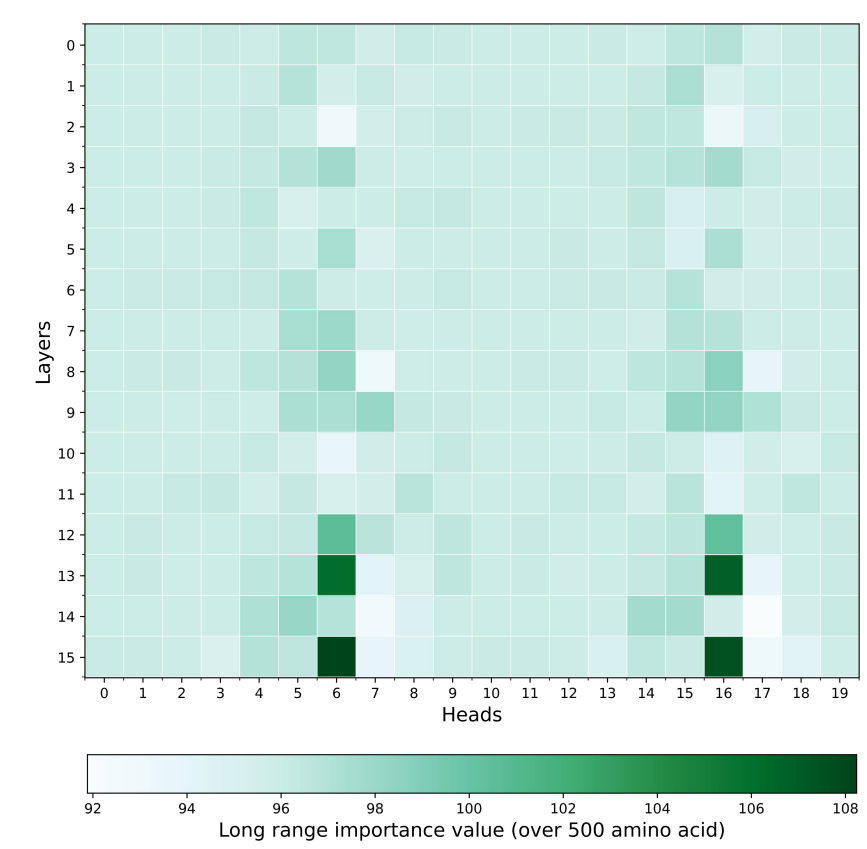

(c)

Figure 8: **Attention analysis of our models.** **(a)** Prevalence of dominant short-range (≤100 aa), medium-range (100–500 aa), and long-range (>500 aa) sectors across viral genomes. The chart represents 1,000 randomly selected viral genomes (≤13,000 aa in length), showing the proportion of genomes in which each interaction range category exhibited the highest mean attention score. **(b)** Distribution of normalized attention scores across interaction distance categories in viral proteomes. Stacked bar charts represent the distribution of attention scores for each interaction range category across 1,000 viral genomes. **(c)** Fine-grained analysis of long-range attention patterns in the LV-3C model. Darker (green) blocks indicate attention heads with a predominant long range attention. The visualization demonstrates how attention mechanisms capture biologically significant inter-protein interactions spanning substantial genomic distances within viral proteomes. Distribution variance indicates the relative focus of attention heads on specific interaction distances.

To assess generalization across viral diversity, we evaluated model performance specifically on RNA viruses, which represent the most abundant and challenging subgroup in our dataset (Supplementary Material S5). Despite their higher mutation rates and structural complexity, our models consistently outperformed baselines (Supplementary Figure S6), suggesting robust performance across viral groups.

To assess sensitivity to our 90% deduplication threshold, we additionally clustered at 65% identity, yielding 246,737 sequences—indicating that 71% of sequences retained at 90% clustering have no other sequence sharing >65% identity. This analysis suggests that the majority of our evaluation data consists of substantially divergent sequences, not near-duplicates. The impact of sequence similarity also varies by task: perplexity is most sensitive to train/test similarity, while taxonomy and species classification are less affected since these tasks require learning species-level patterns,

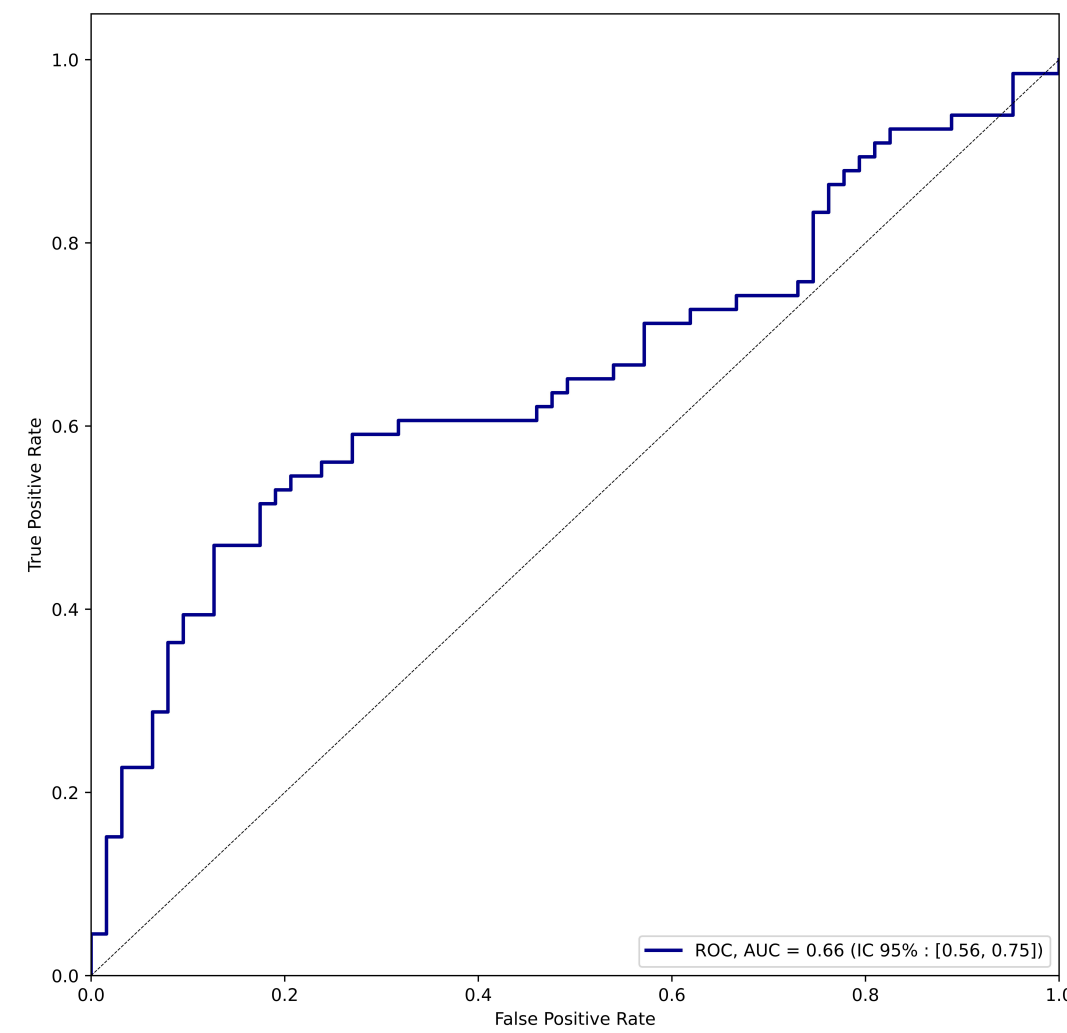

Figure 9: **Receiver Operating Characteristic (ROC) curve of the binary classification task.** ROC curve for distinguishing STRING-supported protein pairs from non-reported protein pairs using attention-derived pair scores. The curve shows LV-3C ability to distinguish experimentally validated interactions (STRING confidence ≥ 0.500) from non-interacting protein pairs across five viral genomes (146 positive interactions). The AUC of 0.66, with a 95% bootstrapped confidence interval of [0.56, 0.75], indicates statistically significant predictive performance.

making within-species similarity a desired signal rather than a confounder. Critically, all models were evaluated under identical conditions, ensuring valid relative comparisons.

To validate that our embeddings capture functional information beyond taxonomy, we evaluated performance using Pfam protein domain annotations (Supplementary Figure S5). While silhouette scores are low, reflecting that genomic context, not domain identity, is the dominant organizing principle—classification performance remains strong, with LV-3C and LV-5B outperforming baselines. This confirms that domain information is encoded within our embeddings alongside the contextual signals that distinguish proteins with identical domains in different genomic environments.

Despite these advances, computational overhead remains a practical consideration. Our selection of a maximum of 50 protein–protein interactions for the sparse attention mechanism strikes an effective balance for viral datasets while maintaining computational efficiency. This parameter can be readily increased if GPU resources are available. We intentionally constrained our implementation to run on a single GPU, as we believe this represents a widely accessible computational environment for most research groups. While we utilized an NVIDIA A6000 for this study, the rapid advancement of more affordable GPUs (sub-$5,000) with comparable memory capacities makes our approach increasingly accessible. For processing significantly larger genomes—particularly from eukaryotic organisms—additional optimizations may be beneficial. Additionally, our semi-supervised approach to inferring putative interactions could benefit from larger ground-truth databases or complementary evidence from proteomic or structural studies to further refine the biological relevance of the learned attention patterns.

Although our training data includes human pathogen sequences, our models pose substantially lower biosecurity risk compared to generative genomic models such as Evo [35]. Both LV-3C and LV-5B are designed to produce embeddings for downstream analysis rather than to generate novel sequences. The masked language modeling objective predicts individual positions given surrounding context, rather than generating complete sequences autoregressively. These distinctions substantially limit the utility of our models for generating functional novel proteins or genomes.

Looking forward, our framework offers considerable promise for extension to larger viral genomes, bacteriophages, and potentially even eukaryotic genomes with appropriate scaling. The methodology could be particularly valuable for analyzing metagenomic data, where contextual information might help resolve ambiguities in protein function assignment. Furthermore, the correlation between embedding similarity and taxonomic distance suggests potential applications in phylogenetic analysis and evolutionary studies.

6 Conclusion

Our study proposes a novel framework for viral protein language modeling by extending transformer architectures to process entire viral genomes. By integrating a biologically informed sparse attention mechanism, our approach efficiently captures putative long-range inter-protein interactions that are missed by conventional single-protein models. This design not only reduces perplexity across extended sequences (up to 61,000 aa) but also generates context-aware embeddings that accurately recapitulate known co-evolutionary relationships—for instance, among PolA, RNR, and helicase.

Our results demonstrate that large-scale learning improves embedding quality. The enhanced clustering of protein and genome embeddings, along with robust taxonomic classification, underscores the broader applicability of our method to phylogenetic analysis and metagenomic studies. Our work provides a solid foundation for working with viral genomes and paves the way for more refined analyses of protein–protein interactions, ultimately deepening our understanding of viral biology and evolution. Looking ahead, our framework offers considerable promise for extension to larger genomes, and potentially even eukaryotic genomes. By refining the biological specificity of sparse attention patterns and incorporating additional layers of experimental validation, our approach can continue to evolve toward more precise and comprehensive genomic analyses. Ultimately, this work not only deepens our understanding of viral genome organization and evolution but also provides a scalable framework for protein modeling at genomic scales, bridging the gap between computational biology and experimental validation.

Availability of source code and requirements

- Project name: ViralEmbed
- Project home page: `https://github.com/Hawaii-Bioinformatics/ViralEmbed`
- Operating system(s): Linux and macOS
- Programming language: Python ($\geq$ 3.9)
- Other requirements: PyTorch ($\geq$ 1.8), fair-esm, transformers, Biopython; a CUDA-capable GPU with $\geq$ 16 GB VRAM is recommended for processing long genomes
- License: MIT
- RRID: SCR_027596

Data availability

Machine learning annotations have been deposited in the DOME registry [36]. All additional supporting data are available in the *GigaScience* repository, GigaDB [37].

List of abbreviations

aa: amino acid(s); ALiBi: Attention with Linear Biases; APC: Average Product Correction; AUC: Area Under the Curve; BSR: Block Sparse Row; CD-HIT: Cluster Database at High Identity with Tolerance; CRediT: Contributor Roles Taxonomy; DOME: Data, Optimization, Model, Evaluation; ESM: Evolutionary Scale Modeling; gLM: genomic language model; GPU: Graphics Processing Unit; HEL: helicase; LLM: large language model; LoRA: Low-Rank Adaptation; MMseqs2: Many-against-Many sequence searching 2; NCBI: National Center for Biotechnology Information; PE: positional encoding; Pfam: Protein family database; pLM: protein language model; PolA: Family A DNA polymerase; PPI: protein–protein interaction; PST: Protein Set Transformer; RNR: ribonucleotide reductase; RoPE: Rotary Position Embedding; ROC: Receiver Operating Characteristic; RRID: Research Resource Identifier; STRING: Search Tool for the Retrieval of Interacting Genes/Proteins; VRAM: Video Random Access Memory.

Competing interests

The authors declare that they have no competing interests.

Authors' contributions

TD: Conceptualization, Methodology, Software, Formal analysis, Investigation, Visualization, Writing (original draft). BDF: Conceptualization, Formal analysis, Writing (review and editing). WH: Validation. ZDS: Data curation, Formal analysis, Investigation. RS: Data curation. KEW: Conceptualization, Writing (review and editing). SWP: Conceptualization, Writing (review and editing). MB: Conceptualization, Methodology, Software, Formal analysis, Investigation, Supervision, Project administration, Funding acquisition, Writing (original draft), Writing (review and editing). All authors read and approved the final manuscript.

Acknowledgments

This work is supported by the National Science Foundation grants 1736030 and 2025567. Support from the University of Delaware Center for Bioinformatics and Computational Biology (CBCB) Core Facility, the University of Delaware Sequencing and Genotyping Center, and use of the BIOMIX compute cluster was made possible through funding from Delaware INBRE (NIGMS P20GM103446, NIH S10OD028725), and the State of Delaware.

References

[1] Xueliang Liu. Deep Recurrent Neural Network for Protein Function Prediction from Sequence. January 2017. doi: 10.48550/arXiv.1701.08318. URL `http://arxiv.org/abs/1701.08318`. arXiv:1701.08318 [q-bio].

[2] Abel Chandra, Laura Tünnermann, Tommy Löfstedt, and Regina Gratz. Transformer-based deep learning for predicting protein properties in the life sciences. *eLife*, 12:e82819, 2023. ISSN 2050-084X. doi: 10.7554/eLife.82819. URL `https://www.ncbi.nlm.nih.gov/pmc/articles/PMC9848389/`.

[3] Zeming Lin, Halil Akin, Roshan Rao, Brian Hie, Zhongkai Zhu, Wenting Lu, Allan dos Santos Costa, Maryam Fazel-Zarandi, Tom Sercu, Sal Candido, and Alexander Rives. Language models of protein sequences at the scale of evolution enable accurate structure prediction. July 2022. doi: 10.1101/2022.07.20.500902. URL `https://www.biorxiv.org/content/10.1101/2022.07.20.500902v1`. Pages: 2022.07.20.500902 Section: New Results.

[4] Ahmed Elnaggar, Michael Heinzinger, Christian Dallago, Ghalia Rehawi, Yu Wang, Llion Jones, Tom Gibbs, Tamas Feher, Christoph Angerer, Martin Steinegger, et al. Prottrans: toward understanding the language of life through self-supervised learning. *IEEE transactions on pattern analysis and machine intelligence*, 44(10): 7112–7127, 2021. doi: 10.1109/TPAMI.2021.3095381.

[5] Erik Nijkamp, Jeffrey A. Ruffolo, Eli N. Weinstein, Nikhil Naik, and Ali Madani. ProGen2: Exploring the boundaries of protein language models. *Cell Systems*, 14(11):968–978, 2023. doi: 10.1016/j.cels.2023.10.002.

[6] Claire D. McWhite and Mona Singh. Vector-clustering Multiple Sequence Alignment: Aligning into the twilight zone of protein sequence similarity with protein language models, October 2022. URL `http://biorxiv.org/lookup/doi/10.1101/2022.10.21.513099`.

[7] Benjamin Giovanni Iovino and Yuzhen Ye. Protein embedding based alignment. *BMC Bioinformatics*, 25(1):85, February 2024. ISSN 1471-2105. doi: 10.1186/s12859-024-05699-5.

[8] Zeming Lin, Halil Akin, Roshan Rao, Brian Hie, Zhongkai Zhu, Wenting Lu, Nikita Smetanin, Robert Verkuil, Ori Kabeli, Yaniv Shmueli, Allan dos Santos Costa, Maryam Fazel-Zarandi, Tom Sercu, Salvatore Candido, and Alexander Rives. Evolutionary-scale prediction of atomic level protein structure with a language model, October 2022. URL `https://www.biorxiv.org/content/10.1101/2022.07.20.500902v2`. Pages: 2022.07.20.500902 Section: New Results.

[9] Christoph Feinauer, Hendrik Szurmant, Martin Weigt, and Andrea Pagnani. Inter-protein sequence co-evolution predicts known physical interactions in bacterial ribosomes and the trp operon. *PLoS One*, 11(2):e0149166, February 2016. doi: 10.1371/journal.pone.0149166.

[10] Elias T Friman, Ilya M Flyamer, Davide Marenduzzo, Shelagh Boyle, and Wendy A Bickmore. Ultra-long-range interactions between active regulatory elements. *Genome Res.*, 33(8):1269–1283, August 2023. doi: 10.1101/gr.277567.122.

[11] Yiling Jiang, Kuijie Tong, Roubin Yao, Yuanze Zhou, Hanwen Lin, Liubing Du, Yunyun Jin, Liu Cao, Jingquan Tan, Xing-Ding Zhang, Deyin Guo, Ji-An Pan, and Xiaoxue Peng. Genome-wide analysis of protein–protein interactions and involvement of viral proteins in SARS-CoV-2 replication. *Cell & Bioscience*, 11(1):140, July 2021. ISSN 2045-3701. doi: 10.1186/s13578-021-00644-y. URL `https://doi.org/10.1186/s13578-021-00644-y`.

[12] Xindi Wang, Mahsa Salmani, Parsa Omidi, Xiangyu Ren, Mehdi Rezagholizadeh, and Armaghan Eshaghi. Beyond the Limits: A Survey of Techniques to Extend the Context Length in Large Language Models, May 2024. URL `http://arxiv.org/abs/2402.02244`. arXiv:2402.02244 [cs].

[13] Ashish Vaswani, Noam Shazeer, Niki Parmar, Jakob Uszkoreit, Llion Jones, Aidan N. Gomez, Łukasz Kaiser, and Illia Polosukhin. Attention is all you need. *Advances in Neural Information Processing Systems*, 30, 2017.

[14] J Rodney Brister, Danso Ako-Adjei, Yiming Bao, and Olga Blinkova. Ncbi viral genomes resource. *Nucleic acids research*, 43(D1):D571–D577, 2015. doi: 10.1093/nar/gku1207.

[15] Jianlin Su, Murtadha Ahmed, Yu Lu, Shengfeng Pan, Wen Bo, and Yunfeng Liu. RoFormer: Enhanced transformer with rotary position embedding. *Neurocomputing*, 568:127063, 2024. doi: 10.1016/j.neucom.2023.127063.

[16] Ofir Press, Noah A. Smith, and Mike Lewis. Train short, test long: Attention with linear biases enables input length extrapolation. In *International Conference on Learning Representations (ICLR)*, 2022.

[17] Shouyuan Chen, Sherman Wong, Liangjian Chen, and Yuandong Tian. Extending Context Window of Large Language Models via Positional Interpolation, June 2023. URL `http://arxiv.org/abs/2306.15595`. arXiv:2306.15595 [cs].

[18] Tri Dao, Dan Fu, Stefano Ermon, Atri Rudra, and Christopher Ré. FlashAttention: Fast and memory-efficient exact attention with IO-awareness. *Advances in Neural Information Processing Systems*, 35:16344–16359, 2022.

[19] Philippe Tillet, H. T. Kung, and David Cox. Triton: an intermediate language and compiler for tiled neural network computations. In *Proceedings of the 3rd ACM SIGPLAN International Workshop on Machine Learning and Programming Languages*, pages 10–19, Phoenix AZ USA, June 2019. ACM. ISBN 978-1-4503-6719-6. doi: 10.1145/3315508.3329973. URL `https://dl.acm.org/doi/10.1145/3315508.3329973`.

[20] Iz Beltagy, Matthew E. Peters, and Arman Cohan. Longformer: The Long-Document Transformer, December 2020. URL `http://arxiv.org/abs/2004.05150`. arXiv:2004.05150 [cs].

[21] Yukang Chen, Shengju Qian, Haotian Tang, Xin Lai, Zhijian Liu, Song Han, and Jiaya Jia. LongLoRA: Efficient fine-tuning of long-context large language models. In *International Conference on Learning Representations (ICLR)*, 2024.

[22] Manzil Zaheer, Guru Guruganesh, Kumar Avinava Dubey, Joshua Ainslie, Chris Alberti, Santiago Ontanon, Philip Pham, Anirudh Ravula, Qifan Wang, Li Yang, et al. Big Bird: Transformers for longer sequences. *Advances in Neural Information Processing Systems*, 33:17283–17297, 2020.

[23] Aurko Roy, Mohammad Saffar, Ashish Vaswani, and David Grangier. Efficient content-based sparse attention with routing transformers. *Transactions of the Association for Computational Linguistics*, 9:53–68, 2021. doi: 10.1162/tacl_a_00353.

[24] Nadav Brandes, Dan Ofer, Yam Peleg, Nadav Rappoport, and Michal Linial. ProteinBERT: a universal deep-learning model of protein sequence and function. *Bioinformatics*, 38(8):2102–2110, April 2022. ISSN 1367-4811. doi: 10.1093/bioinformatics/btac020.

[25] Baris E Suzek, Yuqi Wang, Hongzhan Huang, Peter B McGarvey, Cathy H Wu, and UniProt Consortium. UniRef clusters: a comprehensive and scalable alternative for improving sequence similarity searches. *Bioinformatics*, 31 (6):926–932, March 2015. doi: 10.1093/bioinformatics/btu739.

[26] Cody Martin, Anthony Gitter, and Karthik Anantharaman. Protein Set Transformer: A protein-based genome language model to power high diversity viromics, July 2024. URL `https://www.biorxiv.org/content/10.1101/2024.07.26.605391v1`. Pages: 2024.07.26.605391 Section: New Results.

[27] Yunha Hwang, Andre Cornman, Elizabeth Kellogg, Sergey Ovchinnikov, and Peter Girguis. Genomic language model predicts protein co-regulation and function. *Nature Communications*, 15(1):2880, 2024. doi: 10.1038/s41467-024-46947-9.

[28] Alexander Rives, Joshua Meier, Tom Sercu, Siddharth Goyal, Zeming Lin, Jason Liu, Demi Guo, Myle Ott, C. Lawrence Zitnick, Jerry Ma, and Rob Fergus. Biological structure and function emerge from scaling unsupervised learning to 250 million protein sequences. *Proc Natl Acad Sci U S A*, 118(15):e2016239118, April 2021. ISSN 1091-6490. doi: 10.1073/pnas.2016239118.

[29] Rachel A. Keown, Jacob T. Dums, Phillip J. Brumm, Joyanne MacDonald, David A. Mead, Barbra D. Ferrell, Ryan M. Moore, Amelia O. Harrison, Shawn W. Polson, and K. Eric Wommack. Novel viral dna polymerases from metagenomes suggest genomic sources of strand-displacing biochemical phenotypes. *Frontiers in Microbiology*, Volume 13 - 2022, 2022. ISSN 1664-302X. doi: 10.3389/fmicb.2022.858366. URL `https://www.frontiersin.org/journals/microbiology/articles/10.3389/fmicb.2022.858366`.

[30] Eugene V. Koonin, Valerian V. Dolja, and Mart Krupovic. The logic of virus evolution. *Cell Host & Microbe*, 30(7):917–929, 2022. ISSN 1931-3128. doi: https://doi.org/10.1016/j.chom.2022.06.008. URL https://www.sciencedirect.com/science/article/pii/S1931312822003134.

[31] Daniel J Nasko, Jessica Chopyk, Eric G Sakowski, Barbra D Ferrell, Shawn W Polson, and K Eric Wommack. Family a DNA polymerase phylogeny uncovers diversity and replication gene organization in the virioplankton. *Front. Microbiol.*, 9:3053, December 2018. doi: 10.3389/fmicb.2018.03053.

[32] torch.sparse_bsr_tensor — PyTorch 2.6 documentation. URL https://pytorch.org/docs/stable/generated/torch.sparse_bsr_tensor.html#torch-sparse-bsr-tensor.

[33] Damian Szklarczyk, Rebecca Kirsch, Mikaela Koutrouli, Katerina Nastou, Farrokh Mehryary, Radja Hachilif, Annika L. Gable, Tao Fang, Nadezhda T. Doncheva, Sampo Pyysalo, Peer Bork, Lars J. Jensen, and Christian von Mering. The STRING database in 2023: protein-protein association networks and functional enrichment analyses for any sequenced genome of interest. *Nucleic Acids Res*, 51(D1):D638–D646, January 2023. ISSN 1362-4962. doi: 10.1093/nar/gkac1000.

[34] Milot Mirdita, Martin Steinegger, and Johannes Söding. MMseqs2 desktop and local web server app for fast, interactive sequence searches. *Bioinformatics*, 35(16):2856–2858, August 2019. ISSN 1367-4803. doi: 10.1093/bioinformatics/bty1057. URL https://doi.org/10.1093/bioinformatics/bty1057.

[35] Eric Nguyen, Michael Poli, Matthew G. Durrant, Armin W. Thomas, Brian Kang, Jeremy Sullivan, Madelena Y. Ng, Ashley Lewis, Aman Patel, Aaron Lou, Stefano Ermon, Stephen A. Baccus, Tina Hernandez-Boussard, Christopher Ré, Patrick D. Hsu, and Brian L. Hie. Sequence modeling and design from molecular to genome scale with Evo, February 2024. URL https://www.biorxiv.org/content/10.1101/2024.02.27.582234v1. Pages: 2024.02.27.582234 Section: New Results.

[36] Thibaut Dejean, Barbra D. Ferrell, William Harrigan, Zachary D. Schreiber, Rajan Sawhney, K. Eric Wommack, Shawn W. Polson, and Mahdi Belcaid. Extending protein language models to a viral genomic scale using biologically induced sparse attention. [dome-ml annotations]. DOME-ML Registry, 2026. https://registry.dome-ml.org/review/q0k9zcy8vm.

[37] Thibaut Dejean, Barbra D. Ferrell, William Harrigan, Zachary D. Schreiber, Rajan Sawhney, K. Eric Wommack, Shawn W. Polson, and Mahdi Belcaid. Supporting data for "extending protein language models to a viral genomic scale using biologically induced sparse attention". GigaScience Database, 2026. URL https://doi.org/10.5524/102826.

[38] William L Harrigan, Barbra D Ferrell, K Eric Wommack, et al. Improvements in viral gene annotation using large language models and soft alignments. *BMC Bioinformatics*, 25:165, 2024. doi: 10.1186/s12859-024-05779-6.

[39] Rajan Sawhney et al. Fine-tuning protein language models unlocks the potential of underrepresented viral proteomes. *PeerJ*, 13:e19919, 2025. doi: 10.7717/peerj.19919.

Supplementary Material

S1 NCBI Virus Database

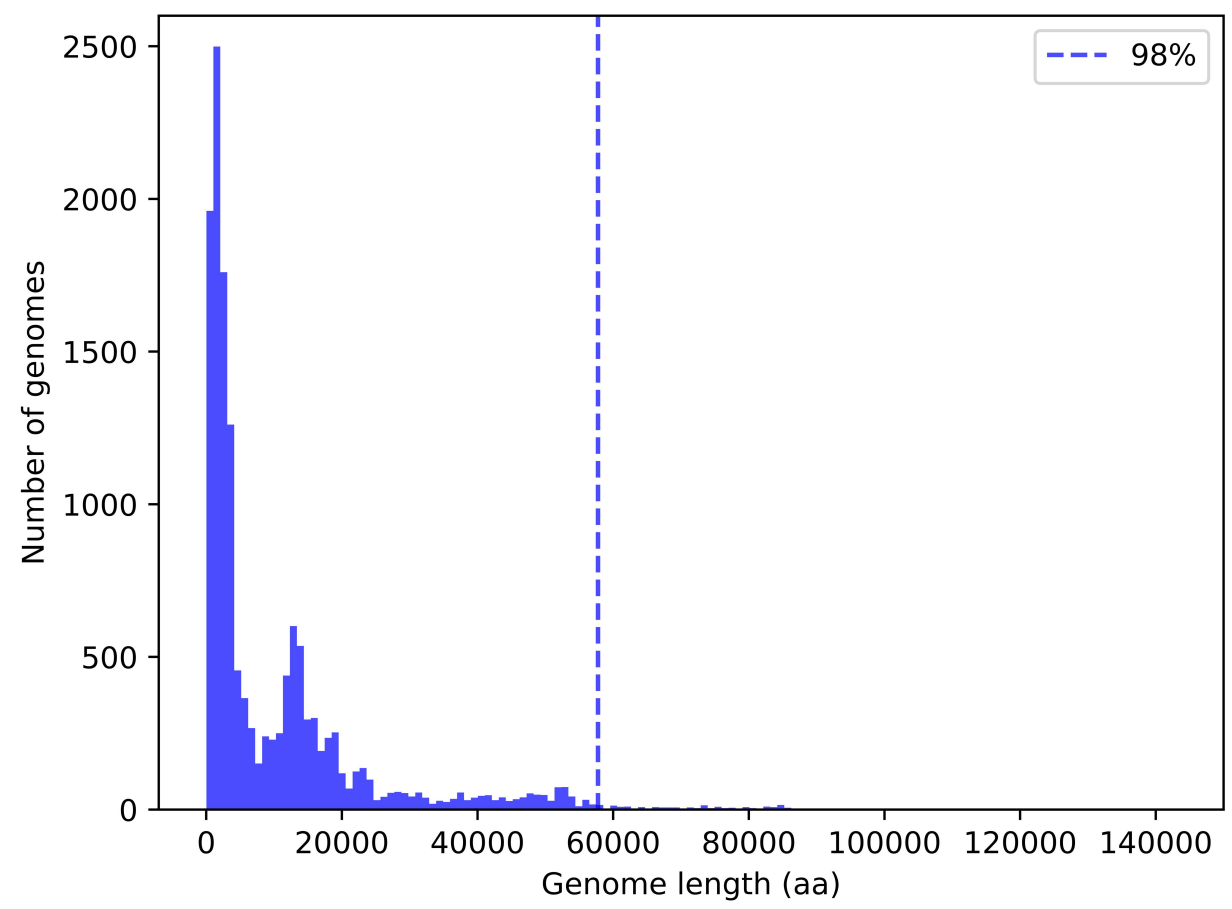

Supplementary Figure S1: **Viral genome length distributions in the NCBI Virus Database.** This histogram shows that a context length of 61k amino acids allows us to process over 98% of the 14,436 complete genomes of the database.

S2 Development of the PPI Inference Model (Stage 1)

To extend ESM-2's context window for PPI inference, we evaluated two positional encoding strategies (RoPE and ALiBi) and three sparse attention mechanisms (Longformer, Shifted Sparse Attention, and BigBird).

The evaluation of positional encoding strategies showed that ALiBi exhibited superior performance in handling extended sequences. Although RoPE demonstrated robust performance within its original training context, its effectiveness deteriorated significantly beyond 4,000 aa. In contrast, ALiBi maintained consistent perplexity (1.99) across sequence lengths extending up to 13,000 aa (Supplementary Figure S2).

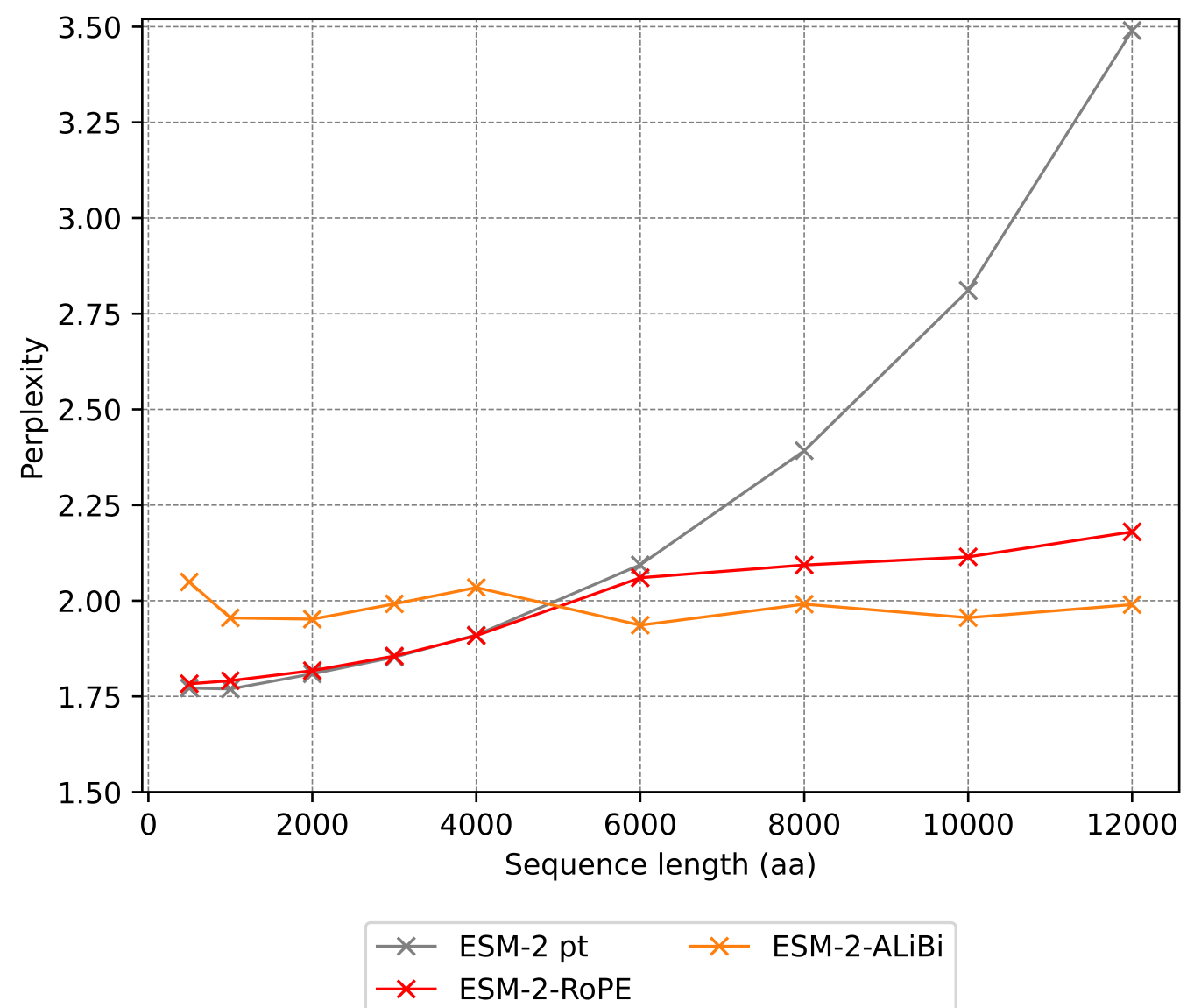

Supplementary Figure S2: **Perplexity evaluation of positional encoding methods for fine-tuning.** Performance metrics for ESM-2-pt and fine-tuned variants using Sliding Window (SW) attention mechanism with RoPE and ALiBi positional encoding. Perplexity values represent mean across sequences ranging from 500 to 13,000 amino acids.

Among the sparse attention mechanisms tested, Longformer provided the optimal balance between performance (perplexity: 1.99, silhouette score: 0.88) and computational efficiency (training time: 32s/step or 46 hours) (Supplementary Table S1). Based on these findings, we adopted ALiBi positional encoding combined with Longformer attention for ESM-2-PPI.

	ESM-2-pt	Longformer	Longformer + full	S^2-Attn	S^2-Attn + full	BigBird
Training context size	1,024	4,000	4,000	4,000	4,000	4,000
Training time (s/step)		32	32	36	36	126
Perplexity	3.17	2.18	**1.99**	2.22	2.05	2.02
Silhouette	0.65	0.85	**0.88**	0.86	0.85	0.84

Supplementary Table S1: **Performance metrics of ESM-2-pt and fine-tuned versions using different sparse attention patterns.** Longformer refers to the Sliding Window Attention pattern from [20], S^2-Attn to the Shifted Sparse Attention from [21] and BigBird to the eponymous attention pattern from [22]. '+full' means normalization and embedding layers are trainable during low-rank fine-tuning. Metrics are calculated on genome fragments ranging between 10,000 and 13,000 amino acids.

To validate that ESM-2-PPI captures biologically meaningful interactions, we analyzed genomic fragments from 83 viral genomes containing complete PolA, RNR, and HEL proteins. These genomes were identified by searching the

NCBI Virus database using BLAST and soft alignment [38, 39] against reference sequences for each protein, retaining only genomes where all three were detected with high confidence (e-value $< 10^{-3}$, coverage $> 80\%$). Supplementary Figure S3 shows that these known interacting pairs consistently rank among the highest-scoring interactions, particularly after excluding genomically adjacent pairs. A Wilcoxon–Mann–Whitney test confirmed a highly significant difference between the distributions ($p = 3.31 \times 10^{-22}$).

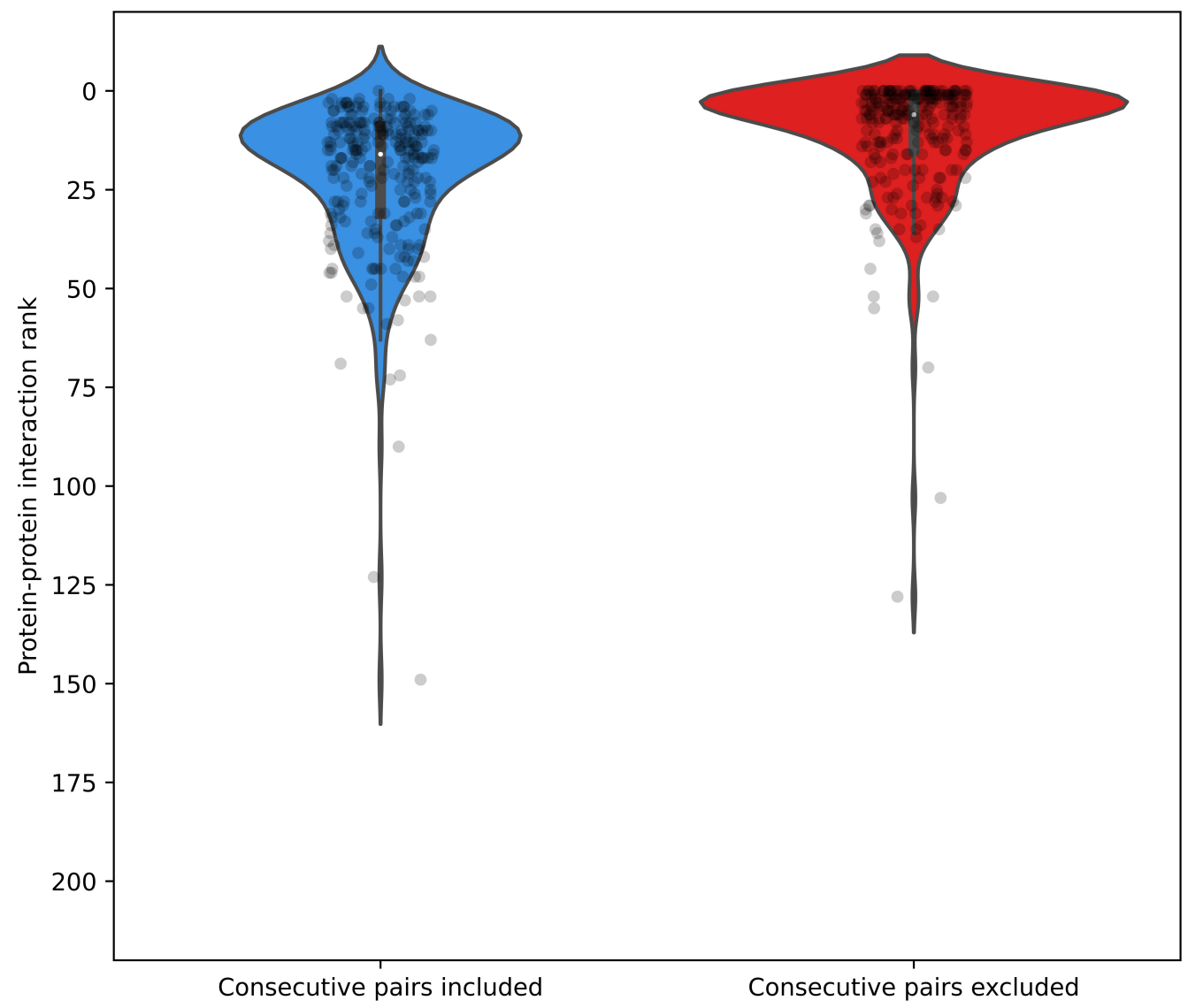

Supplementary Figure S3: **Distribution of interaction pair rankings of DNA polymerase I (PolA), ribonucleotide reductase (RNR), and helicase (HEL) across 83 viral genomes.** Violin plots represent the ranking positions of PolA-HEL, PolA-RNR, and RNR-HEL pairs (total n = 249). Left panel: rankings among all protein pairs within fragments. Right panel: rankings after excluding genomically adjacent protein pairs.

To select an appropriate interaction threshold, we evaluated different values of k (the number of top-ranked non-adjacent pairs to include). Supplementary Figure S4 and Supplementary Table S2 show that $k = 50$ retains 97.6% of known interacting pairs while remaining computationally tractable.

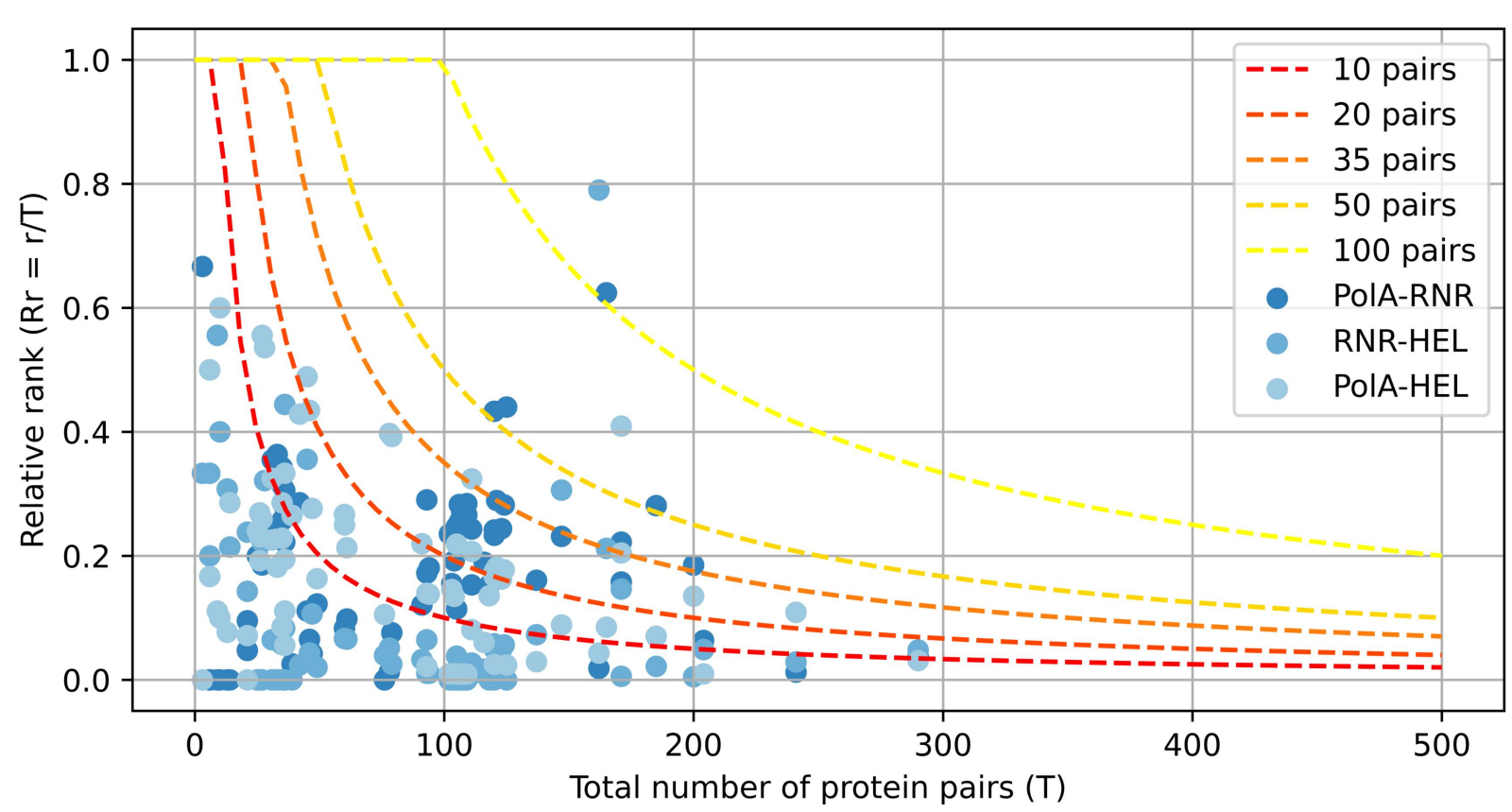

Supplementary Figure S4: **Selection of interaction threshold.** PolA-HEL, PolA-RNR, and RNR-HEL pairs shown as a function of total genomic pairs and relative genomic rank. Dashed curves denote selection thresholds; pairs below each curve are retained for attention calculations.

Threshold (number of selected pairs)	10	20	35	50	100
% studied pairs selected	64.0%	80.6%	94.4%	97.6%	99.2%

Supplementary Table S2: **Percentage of known interacting pairs** (PolA-RNR-HEL) captured at different selection thresholds.

S3 Using embeddings to predict taxonomy

To assess the taxonomic discriminability of the learned embeddings, we conducted a supervised classification analysis. For each protein or genome in the dataset, a 1,280-dimensional embedding vector was generated and paired with its corresponding taxonomic label. The dataset, comprising 100,000 proteins and 5,000 genomes, was randomly partitioned into training (80%) and validation (20%) subsets. To ensure a robust evaluation, we varied the number of taxonomic species from 5 to 100, as described in Section 3.9. A single-layer neural network with an intermediate hidden dimension of 2,560 was trained for 1,000 epochs using a learning rate of 1e-3. Model performance was evaluated using 5-fold cross-validation, and standard classification metrics were reported.

S4 Validation Using Protein Domain Annotations

To assess whether our embeddings capture functional information beyond taxonomic signals, we evaluated clustering and classification performance using Pfam protein family annotations. Pfam families group proteins by conserved domain structure and function, providing an orthogonal validation axis to taxonomy-based evaluations.

Supplementary Figure S5 presents silhouette scores and classification F1-scores using Pfam families as labels. Notably, silhouette scores are negative across all models (Supplementary Figure S5a), indicating that proteins from the same Pfam family do not form tight clusters in embedding space. This result is expected and reflects a key design principle of our approach: our embeddings are trained to encode genomic context, not solely domain identity. A helicase in a bacteriophage and a helicase in an RNA virus belong to the same Pfam family but occupy fundamentally different genomic contexts, and our models are designed to distinguish them. Embeddings that clustered purely by Pfam family would simply recapitulate single-protein models like ESM-2, rather than capturing the genomic relationships that are the focus of this work.

Despite this, classification performance remains strong (Supplementary Figure S5b), with LV-3C and LV-5B consistently outperforming ESM-2-pt and PST across all numbers of Pfam families evaluated. This indicates that Pfam-relevant information is encoded within the embeddings—it is simply not the dominant organizing signal. A linear classifier can extract domain identity even when genomic context is the primary structuring principle of the embedding space.

Taken together, these results demonstrate that our context-aware embeddings capture functional domain information at least as effectively as single-protein baselines, while additionally encoding genomic context that distinguishes proteins with identical domains but different genomic environments.

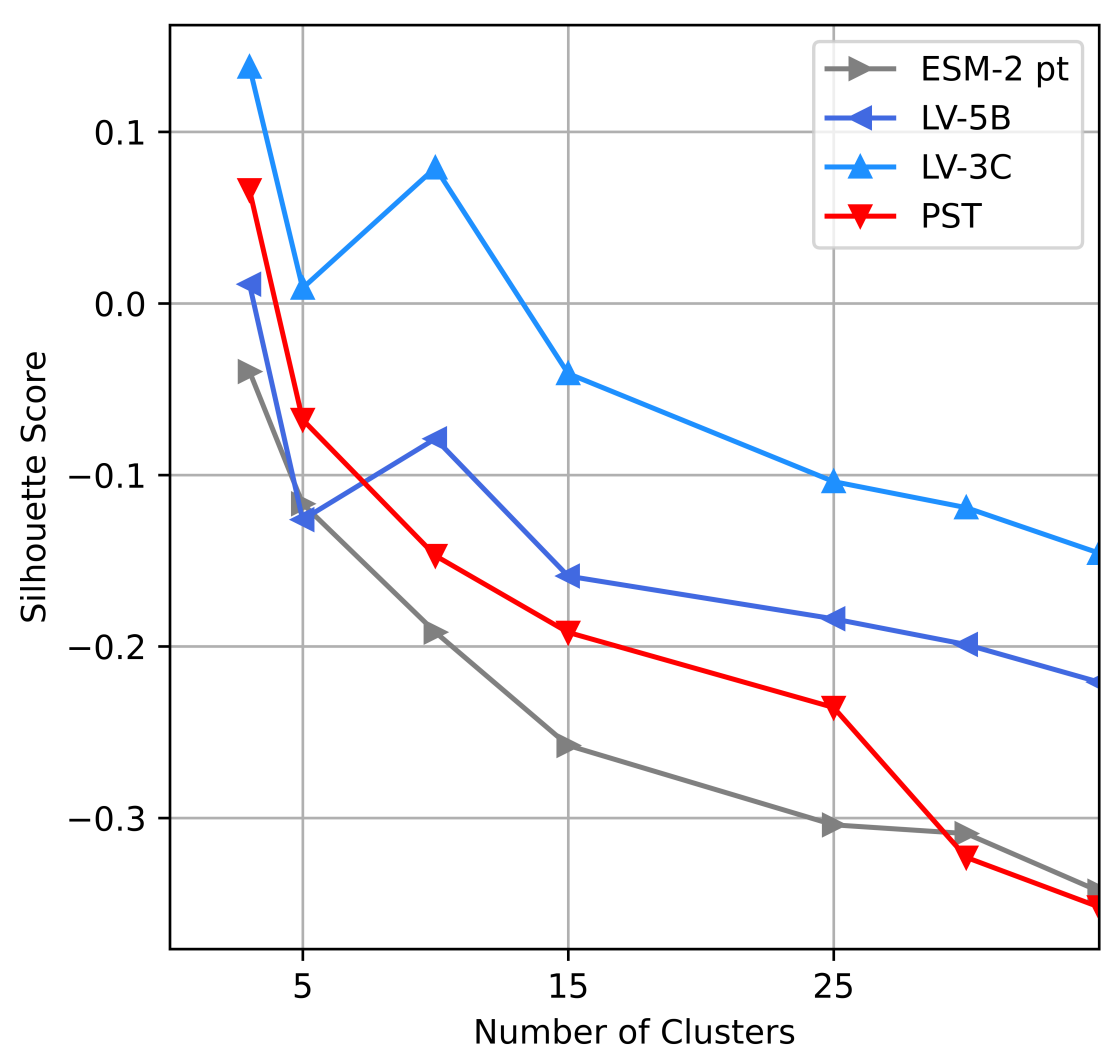

(a) Silhouette Score

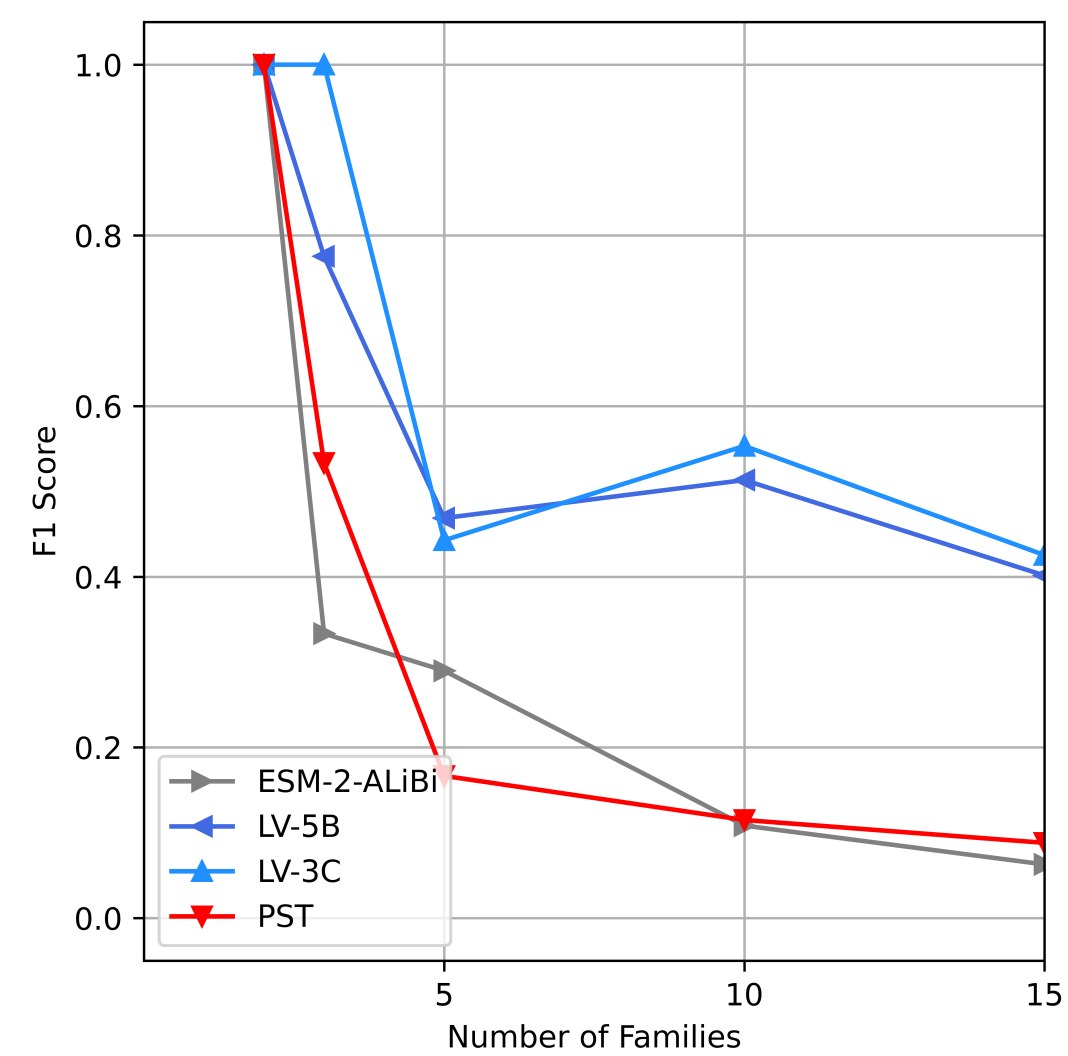

(b) Classification

Supplementary Figure S5: **Evaluation of embeddings using Pfam domain annotations.** **(a)** Silhouette scores using Pfam families as cluster labels. Negative scores across all models reflect that genomic context—not domain identity alone—is the primary organizing principle of the embedding space. **(b)** F1-scores for Pfam family classification. Despite low clustering coherence, LV-3C and LV-5B achieve strong classification performance, indicating that domain information is encoded but not dominant.

S5 Model Performance Across Viral Subgroups

Viral genomes vary substantially in size, structure, and evolutionary dynamics. To assess whether our models generalize across this diversity, we evaluated performance on RNA viruses—the most challenging and abundant subgroup in our dataset (approximately 70% of training genomes).

RNA viruses present unique modeling challenges: their high mutation rates and rapid evolution generate greater sequence diversity, while their broad host ranges and varied genome organizations (segmented, ambisense, positive/negative-sense) introduce additional complexity. If our models perform well on this demanding subgroup, we would expect comparable or better performance on structurally simpler dsDNA and ssDNA viruses.

Supplementary Figure S6 compares model performance on RNA viruses (solid lines) versus all viruses (lighter lines). As expected, performance on RNA viruses is slightly lower than on the full dataset; the latter being lifted by the inclusion of less complex viral groups. Critically, however, LV-3C and LV-5B consistently outperform both ESM-2-pt and PST baselines on RNA viruses across all evaluation metrics. These results demonstrate that our models capture biologically meaningful signals even within the most challenging viral subgroup, suggesting robust generalization across viral diversity.

S6 Controls for the Interaction-Inference Step

To test whether the interaction-inference step itself improves the model, we ran a controlled comparison in which the only parameter that varied was which long-range links each model received. The random conditions use three independent draws whose results we average. Additionally, because the inferred links tend to fall at particular genomic distances, we also included a distance-matched control that draws random links at distances similar to those of the inferred pairs, so that any difference cannot be attributed to genomic distance alone. Together with a model that receives no long-range links and connects only neighboring proteins, this gives eight models.

- **Inferred** (1 model). The 50 top-ranked pairs from the interaction-inference model (ESM-2-PPI).
- **Random** (3 models). 50 pairs drawn uniformly at random, averaged over three independent draws.

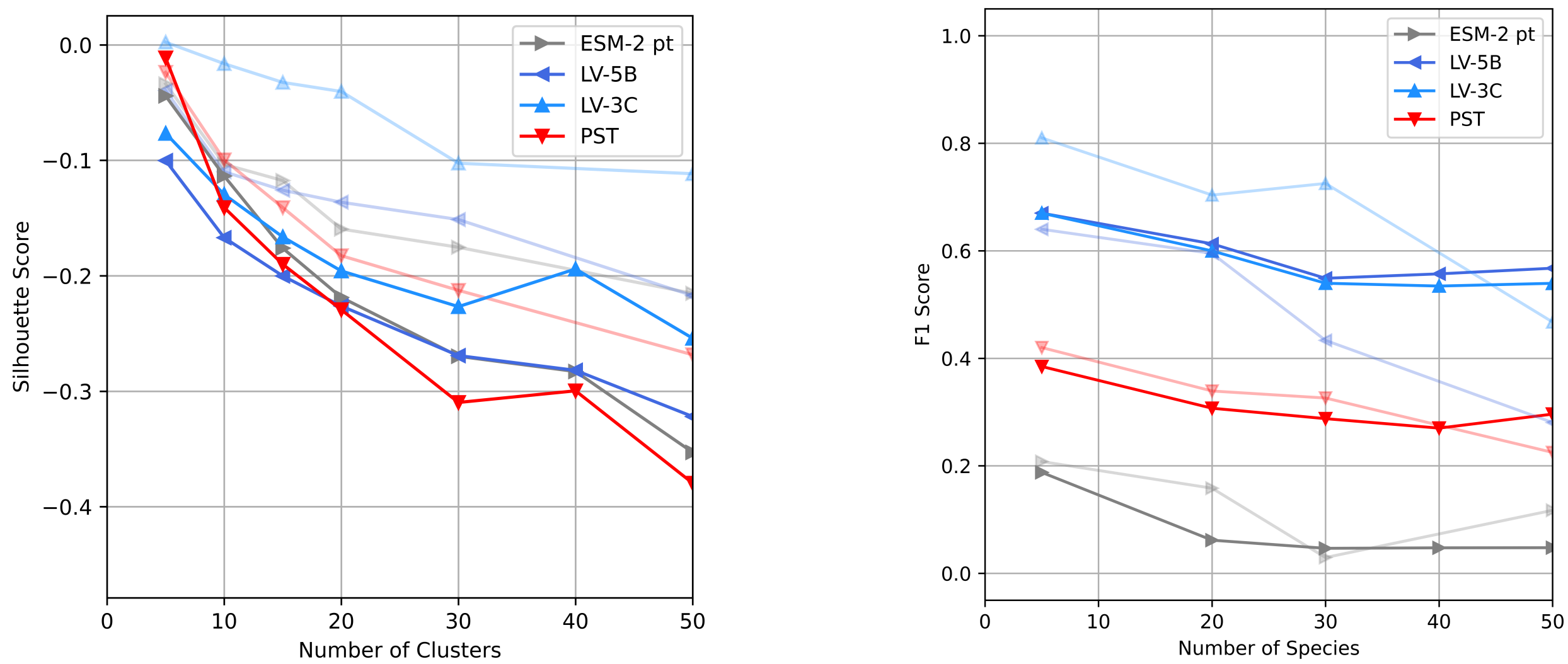

(a) Silhouette Score

(b) Classification

Supplementary Figure S6: **Model performance on RNA viruses compared to all viruses. (a)** Silhouette scores and **(b)** F1-scores for taxonomic species classification, comparing LV-3C, LV-5B, PST, and ESM-2-pt. Solid lines: RNA viruses only; lighter lines: all viruses. Despite the greater complexity of RNA viruses, our models maintain consistent improvements over baselines.

- **Distance-matched** (3 models). 50 random pairs drawn to match the genomic-distance range of the inferred pairs, averaged over three draws.
- **No-link** (1 model). Only neighboring proteins are connected.

We used the ESM-2-35M-parameter model so that all eight could be trained from scratch under identical conditions. All models share the same architecture, initial weights, training data and order, masking, and optimizer, so any difference in their results can come only from which pairs are connected.

Table S3 reports held-out perplexity on 600 genomes. Adding 50 random links leaves perplexity essentially unchanged relative to having no long-range links (16.124 versus 16.120), whereas adding the same number of inferred links lowers it (lower is better). The distance-matched control performs like the other controls, so the advantage reflects which proteins are paired rather than how far apart they occur.

Model	Held-out perplexity
No long-range links	16.120
50 random links (average of 3)	16.124
50 distance-matched links (average of 3)	16.119
50 inferred links	**16.064**

Supplementary Table S3: **Held-out perplexity for the interaction-inference controls.** Lower is better. Only the inferred links lower perplexity relative to having no long-range links.

The genome-wide change is modest (approximately 0.4%), but this figure represents an average across all positions in the genome. Because the 50 links are relevant only to the minority of positions that occur within an inferred PPI link, the majority of positions are unaffected. Averaging this small number of positions against the large number of unaltered ones minimizes the magnitude of the effect. The effect is nonetheless consistent across genomes as the inferred model achieves lower perplexity than the random average on 554 of 600 genomes (92%), and it outperforms the lowest perplexity attained by any of the three random models on a per-genome basis for 78% of genomes.

The improvement in perplexity is also concentrated among the pairs assigned the highest scores by the inference model. We ranked positions according to the reduction in per-position loss conferred by the inferred links relative to the control,

and examined the distribution of the most-improved positions. In Table S4, the baseline rate denotes the fraction of the genome occupied by the proteins in the retained pairs—the value expected by chance—and the top-100 enrichment quantifies how much more frequently the 100 most-improved positions coincide with those pairs than this baseline predicts. The enrichment increases with the confidence of the pairs considered, ranging from approximately 1.4-fold when all 50 pairs are pooled to approximately 11-fold for the highest-scoring pairs. The improvement therefore scales with the model's inferred PPI score rather than emerging at an arbitrary threshold.

Edges kept (by confidence)	Baseline rate	Top-100 enrichment
top 2%	4.1%	11.1×
top 5%	9.0%	7.2×
top 10%	13.8%	5.2×
top 20%	25.6%	3.1×
top 50%	50.4%	1.7×
all inferred pairs	71.6%	1.4×

Supplementary Table S4: **Enrichment of improved positions on high-scoring inferred pairs.** The most-improved positions fall disproportionately on the proteins the inference model ranks most highly.

With every other setting held identical, 50 randomly chosen links give no measurable improvement over having no long-range links, whereas 50 links chosen by the inference model lower held-out perplexity. The advantage is consistent across genomes and concentrates on the pairs the model scores highest, which are the same pairs an independent, separately validated model ranks as most likely to interact.

Personal Cover Click here to access/download;Personal Cover;letter_june_8.pdf

UNIVERSITY of HAWAI'I MĀNOA

College of Natural Sciences
Department of Information and Computer Sciences

June 8, 2026

To: GigaScience Editorial Office

Re: Response to Decision Letter

Dear Dr. Ma,

We are pleased to submit the revised version of our manuscript, "Extending Protein Language Models to a Viral Genomic Scale Using Biologically Induced Sparse Attention." We have addressed every point raised by the first reviewer, either by editing for clarity and resolving ambiguity in the text or by adding the analyses suggested to further solidify the results. We hope the revised manuscript meets the standards of the journal, and we look forward to your response.

Sincerely,

Mahdi Belcaid on behalf of all co-authors
Associate Professor and Associate Department Chair
Information and Computer Sciences
University of Hawaii at Mānoa
mahdi@hawaii.edu

1680 East-West Road, POST 317
Honolulu, Hawai'i 96822
Telephone: (808) 956-6049
Fax: (808) 956-3548
An Equal Opportunity/Affirmative Action Institution
