# Supplementary material for: Extending protein language models to a viral genomic scale using biologically induced sparse attention
Source: Gigascience. 2026 Jul 21;15:giag081. doi: 10.1093/gigascience/giag081 (PMC13495794; doi:10.1093/gigascience/giag081)
Supplement: Supplementary file 6 [file giag081_reviewer_1_report_original_submission.pdf]

Reviewer Report

Title: Extending Protein Language Models to a Viral Genomic Scale Using Biologically Induced Sparse Attention

Version: Original Submission **Date:** 12/11/2025

Reviewer name: Reviewer 1

Reviewer Comments to Author:

The paper by Dejean, et. al. describes a novel approach, and set of models, to extend protein language models to a much larger context window (60,000 amino acids) using a combination of approaches to extending the context window including biologically-informed attention mechanisms. The approach is interesting, and potentially very useful. The authors apply it to viral sequences, and show in a variety of ways, improvements over existing models. The manuscript could be improved in several ways detailed below:

1. The authors of the evo paper (which is referenced in the current paper), a DNA language model with a large context, thought it necessary to specifically exclude eukaryotic and thus potential human pathogen viral sequences from their training because of the potential safety issues around their generative model. It is A) not clear if any similar filtering was done in the current study (it is not mentioned, so I assume not), B) not clear if this is *as* necessary as it may be with the evo model - that is, do you consider your models to be usefully generative? At the very least some discussion of this important consideration should be mentioned in the paper.

2. The different models that are developed and used in the paper are overall confusing. This is a significant issue with the paper since it makes it very difficult to figure out what results map to which models, how the pieces fit together, and the significance of the advances. My feeling is that reducing the number of different models and forms of different models being referred to in the main text (see point 3, below).

a. If I understand (and I may not), ESM is used in some form (fine tuned, I think) to serve as the first stage that predicts protein interactions, which are then fed into a second stage which (may be) the two models labelled LV-3C and LV-5B. ESM is used in two forms (maybe more?) - which seems to be the original form and a fine-tuned form which might have an extended context window and also might be fine-tuned on the same input sequence?

b. The first part of the results section, which talks about predicting protein interactions seems to describe this fine-tuning. But it's not clear. There are two positional encoding and 3 sparse encoding strategies tested (which is fine) but it makes it really hard to figure out what is then used, and why. The motivation for this section, and the choices for the models needs to be made clear. Also, there seem to be variants of the fine-tuning that are used throughout? This makes things more confusing.

c. The difference between the two main models, LV-3C and LV-5B are not clear. 3C is trained on genomic segments using 'sparse attention informed by protein-protein interactions' (are these from the first stage?) and 5B is trained on shorter segments - but using transfer learning from ESM-2 and using 'biologically-informed sparse attention'. How is 'biologically-informed' different than 'protein-protein

interactions'? Much more clarity around the differences and motivation for each of these models is needed to be able to interpret the following results.
d. ESM 'flavors' are used to compare - as baselines - for the other models, but since it's hard to follow what these ESM flavors are exactly it makes interpretation difficult. Also, the same ESM model(s) is used for the 'first stage' to predict interactions as to compare against the final 'second stage' models? This needs to be made clear. Related, I liked the use of the PolA, RNR, and HEL to optimize the number of interactions needed. But then this same complex is used as an example in the final model. Which is confusing, and also, it's not entirely clear that it's not circular (I feel it's not, but this really needs some explanation and clarity) - that is, 'we used PolA, RNR, and HEL to optimize our stage 1, and we find that our stage 2 is really good at predicting these interactions'
e. PST is introduced as another model not trained here, but included as a comparator for only one part of the results - Fig 9, evaluation of protein embeddings. It's not clear why it needs to be included in these results, but not for others?
f. Methods 3.4 selecting an optimal fine tuning strategy: for what model or portion of the model?
3. The paper would *greatly* benefit from some revision and streamlining to make the entire process, the models used, and the methods more clear. I would suggest trying to move some of the results to a supplemental section: the discussion of interaction inference (section 4.1) or 'stage 1' and the different things tried there to be confusing - whereas I might be very interested in those results and what was being done if they weren't confusing (moving those to the supplement is one way of accomplishing this, then describing 'we optimized stage 1 (see supplement) and decided on using XXX for the final model because it showed XXX.'
4. Each in the results must have a clear motivation sentence or section right at the start: what is the hypothesis that's being examined here? What are you doing to test this hypothesis (give enough of an idea of the approach/methods to give the reader a reminder)? Then at the end, What do the results tell you and how does it suggest the next section?
5. The Related Work section is well-written and was very useful to give me good background information. However, it reads more like a mini-review paper than a focused examination of the approaches which are used in the current paper - and, importantly, it's not clear that all the methods described are important to understand for the paper. That is, which parts are *needed* for the reader to understand the approaches tested and/or used in the final work. A short section at the start of this that briefly teases that the final model includes elements from these different approaches would be helpful so the reader knows why they should be paying attention.
6. Perplexity and silhouette score are used extensively in the results section, but never described in the methods section.
7. The final paragraph of the introduction seems out of place. This should be moved to the discussion maybe? It's an odd way to end the introduction, and feels like it's more of a caveat that can be discussed after, than the most important thing we should be taking from the paper.
8. Methods: the description of Splits is not clear. It seems that genomes, and collections of proteins from the same genome were kept together in a single split so as not to contaminate the evaluation, which makes sense, but just needs to be stated in a more careful and clear way.
9. Methods: protein non-redundancy - the use of an identity filter to separate similar proteins is A) welcome, it's an important thing to do, but… B) 90% seems overly generous - that is, it makes the task

pretty easy since 89% sequence identity is still *very* similar. I would welcome a mention of this in the discussion, and some more supplemental results that show a few of the downstream task comparisons using the more stringent 50% filter.

10. Methods: metadata - I can't tell if the first paragraph only is the 'metadata' part (that seems reasonable) but the rest of the section is not about metadata and probably should be titled something on its own.

11. Methods: the section titled 'Ablation' doesn't immediately strike me as being about ablation at all? For this (and some of the other methods sections too) a short lead in of what the method describes would be good 'We needed an approach to limit the number of interactions used for the context so we …'. Also, leading with 'We varied k…' - what is k (it's there in the equation, but the reader won't know that)?

12. Figure listing is out of order it seems? Maybe because methods refer to results section - not sure.

13. Table 1 - what is S2? Also really not clear what the other labels refer to exactly either

14. Figure 5 is fairly easy to digest visually (removing the adjacent proteins improves the ranking of known interactions) but would very much benefit from a quantitative measure of how different these distributions are (likely a p-value from some test)

15. Section 4.2. The motivation for using MMseqs2 is not clear. I gather it's to provide a baseline 'trusted' clustering to compare to? Also this is not described in the methods.

16. I would suggest moving the discussion of analysis of variance and distribution of the embeddings to the first downstream analysis reported. It is useful, but really doesn't say anything about model quality or performance by itself. If you put it first (in section 4.4) it will lead in to the other measures, which are stronger (in my opinion).

17. Comparison of the embeddings to STRING is a nice addition, but not much time is spent on describing how this is done. 146 interactions (I'm assuming these are TPs) are considered. What are used as negatives? What does it mean to have an F1 score of 0.74 (which seems - pretty good) and an AUC of 0.66 (which seems marginal)? The final sentence in this section is not supported (no correlation between STRING confidence scores and attention scores was shown).

18. Figure 12 needs a figure legend.

19. Section 4.3 the authors state that 'The aim of clustering-based evaluation is to identify consistency between embeddings in latent space' - but it's not clear that this is how clustering is being used or evaluated following this? - the performance of the different models on a classification task don't directly evaluate how consistent the embeddings are (you could have very different embeddings that lead to similar performance, e.g.)

Methods

Are the methods appropriate to the aims of the study, are they well described, and are necessary controls included? Choose an item.

Conclusions

Are the conclusions adequately supported by the data shown? Choose an item.

Reporting Standards

Does the manuscript adhere to the journal's guidelines on [minimum standards of reporting?]() Choose an item.

Choose an item.

Statistics

Are you able to assess all statistics in the manuscript, including the appropriateness of statistical tests used? Choose an item.

Quality of Written English

Please indicate the quality of language in the manuscript: Choose an item.

Declaration of Competing Interests

Please complete a declaration of competing interests, considering the following questions:

- Have you in the past five years received reimbursements, fees, funding, or salary from an organisation that may in any way gain or lose financially from the publication of this manuscript, either now or in the future?
- Do you hold any stocks or shares in an organisation that may in any way gain or lose financially from the publication of this manuscript, either now or in the future?
- Do you hold or are you currently applying for any patents relating to the content of the manuscript?
- Have you received reimbursements, fees, funding, or salary from an organization that holds or has applied for patents relating to the content of the manuscript?
- Do you have any other financial competing interests?
- Do you have any non-financial competing interests in relation to this paper?

If you can answer no to all of the above, write 'I declare that I have no competing interests' below. If your reply is yes to any, please give details below.

I declare that I have no competing interests

I agree to the open peer review policy of the journal. I understand that my name will be included on my report to the authors and, if the manuscript is accepted for publication, my named report including any attachments I upload will be posted on the website along with the authors' responses. I agree for my report to be made available under an Open Access Creative Commons CC-BY license (http://creativecommons.org/licenses/by/4.0/). I understand that any comments which I do not wish to be included in my named report can be included as confidential comments to the editors, which will not be published.

Choose an item.

To further support our reviewers, we have joined with Publons, where you can gain additional credit to further highlight your hard work (see: https://publons.com/journal/530/gigascience). On publication of this paper, your review will be automatically added to Publons, you can then choose whether or not to claim your Publons credit. I understand this statement.

Yes Choose an item.
