# Supplementary material for: Extending protein language models to a viral genomic scale using biologically induced sparse attention
Source: Gigascience. 2026 Jul 21;15:giag081. doi: 10.1093/gigascience/giag081 (PMC13495794; doi:10.1093/gigascience/giag081)
Supplement: Supplementary file 7 [file giag081_reviewer_1_report_revision_1.pdf]

Reviewer Report

Title: Extending Protein Language Models to a Viral Genomic Scale Using Biologically Induced Sparse Attention

Version: Revision 1 **Date:** 5/5/2026

Reviewer name: Reviewer 1

Reviewer Comments to Author:

The authors do a reasonable job of addressing the concerns I previously raised and the manuscript is improved with respect to clarity and content. I believe that this is a valuable study with interesting results. However, one point was not addressed (shortening and clarifying the introduction) and for several others the authors response did not match the updated reorganized text. Though I was able to mostly follow the changes, the lack of clarity in the response and the authors lack of care with their responses made this challenging:

1. My original point 5 is addressed in the authors response, but not included in the paper. The introduction is still too long and has not been revised/clarified.
2. My original point 13 has been addressed but in the restructuring of the paper Table 1 has been moved to the supplement, which is fine, but just makes tracking the updates to the paper difficult.
3. Likewise - my original point 14 was about Figure 5, which was addressed but Figure 5 was moved to Supplementary Figure S3 (not indicated in the revision comments).
4. My point 15 was addressed? I think - but the response from the authors to my point includes text that is not in the paper so it's hard to evaluate.
5. My original point 18: Figure 12 has been moved to supplement, but this wasn't indicated in the response.

Since the paper was substantially reorganized I reread it to find a few issues not addressed or not present in my first review:

1. In the abstract the word 'perplexity' is used to describe the improvement in performance of the method. Though perplexity is a (somewhat) common term for AI/ML this should be explained for readers not familiar - or another word/phrase used in the abstract at least.
2. In the introduction the authors state: "Our semi-supervised protein language model employs sparse attention mechanisms guided by known protein-protein interactions." which is misleading given that a major point of the paper is to use PLMs to infer protein interactions? It seems that the model *can* use known protein-protein interactions, but that's not the main thrust of the paper.
3. As noted previously and above section 2 is great, but doesn't need to be here. This should be condensed down to *only* what is necessary to understand the motivation for choices in the paper. As it stands this significantly detracts from the paper since it is a *lot* of unnecessary content that readers must get through before they get to the content of the paper.
4. Inferring PPIs to address the attention issue is a strong idea. However, the text states that this is a 'biologically informed sparse attention method', which is not really correct. The *motivation* for this is biological information - but the way it's being applied is through further inference. It is important to

note that prediction of protein-protein interactions is not a solved problem, and - importantly - this paper does not really address the issue. The use of a single protein complex to evaluate/parameterize the approach is OK, but is insufficient to establish that it works to accurately predict protein interactions. The approach itself (using ESM to filter for interactions) is fine - I would simply suggest revising the language about what this process is accomplishing (maybe 'enriching for putative protein-protein interactions' or something similar). It would be important to see an addition to the analysis and evaluation that would be a model which included randomized protein interactions (instead of inferred) to show that the PPI inference process improves overall model performance. The baselines provided by ESM-pt, ESM-ROPE, and ESM-ALIBI are good, but don't *seem* to address the same question [it is unclear to me, which needs to be clarified as well].
5. 3.10 and 3.11 seem to be referring to a downstream task rather than the upstream task of predicting PPIs to make sparse attention matrices. However, the parallel nature of these activities is confusing and should be carefully described and delineated.
6. It is really unclear from section 4.1 what is being done and why? There are insufficient results presented in 4.1 to demonstrate that this is working (see my point 4 above). The wording in places is confusing: "we optimized a PPI inference model (ESM-2-PPI) to" implies that an existing model is being optimized, when really this is a new model based on ESM. Explaining what this is and why it's done briefly again here would be important. 3.2 stage 1 describes the ESM-2-PPI training process, and does not mention anything about about using ROPE and ALIBI models in the PPI prediction, and the reasons for using them are not clearly laid out in 4.1. "We evaluated two positional encoding.." - evaluated on what? Again - this is in the Methods somewhere, but needs to be also described here.
7. Section 4.2 highlights my prior comment (#4) about needing to motivate each results section. The section starts out, "The LV-3C and LV-5B models demonstrated significantly lower perplexity…" - where we don't know what LV-3C and LV-5B models are and how they differ (again, yes - the Methods section talks about this, but this needs to be clearly motivated and explained, briefly, in this section as well).
8. Figure 7: it's not clear what the different number of clusters means - I assume from broader (smaller number of clusters) to more specific (larger number of clusters) for taxonomy. This should be made clear in the text.

Methods

Are the methods appropriate to the aims of the study, are they well described, and are necessary controls included? Choose an item.

Conclusions

Are the conclusions adequately supported by the data shown? Choose an item.

Reporting Standards

Does the manuscript adhere to the journal's guidelines on minimum standards of reporting? Choose an item.

Choose an item.

Statistics

Are you able to assess all statistics in the manuscript, including the appropriateness of statistical tests used? Choose an item.

Quality of Written English

Please indicate the quality of language in the manuscript: Choose an item.

Declaration of Competing Interests

Please complete a declaration of competing interests, considering the following questions:

- Have you in the past five years received reimbursements, fees, funding, or salary from an organisation that may in any way gain or lose financially from the publication of this manuscript, either now or in the future?
- Do you hold any stocks or shares in an organisation that may in any way gain or lose financially from the publication of this manuscript, either now or in the future?
- Do you hold or are you currently applying for any patents relating to the content of the manuscript?
- Have you received reimbursements, fees, funding, or salary from an organization that holds or has applied for patents relating to the content of the manuscript?
- Do you have any other financial competing interests?
- Do you have any non-financial competing interests in relation to this paper?

If you can answer no to all of the above, write 'I declare that I have no competing interests' below. If your reply is yes to any, please give details below.

I declare that I have no competing interests

I agree to the open peer review policy of the journal. I understand that my name will be included on my report to the authors and, if the manuscript is accepted for publication, my named report including any attachments I upload will be posted on the website along with the authors' responses. I agree for my report to be made available under an Open Access Creative Commons CC-BY license (http://creativecommons.org/licenses/by/4.0/). I understand that any comments which I do not wish to be included in my named report can be included as confidential comments to the editors, which will not be published.

I agree to the open peer review policy of the journal

To further support our reviewers, we have joined with Publons, where you can gain additional credit to further highlight your hard work (see: https://publons.com/journal/530/gigascience). On publication of this paper, your review will be automatically added to Publons, you can then choose whether or not to claim your Publons credit. I understand this statement.

Yes Choose an item.
