# Supplementary material for: Extending protein language models to a viral genomic scale using biologically induced sparse attention
Source: Gigascience. 2026 Jul 21;15:giag081. doi: 10.1093/gigascience/giag081 (PMC13495794; doi:10.1093/gigascience/giag081)
Supplement: Supplementary file 10 [file giag081_supplemental_file.pdf]

Supplementary Material

S1 NCBI Virus Database

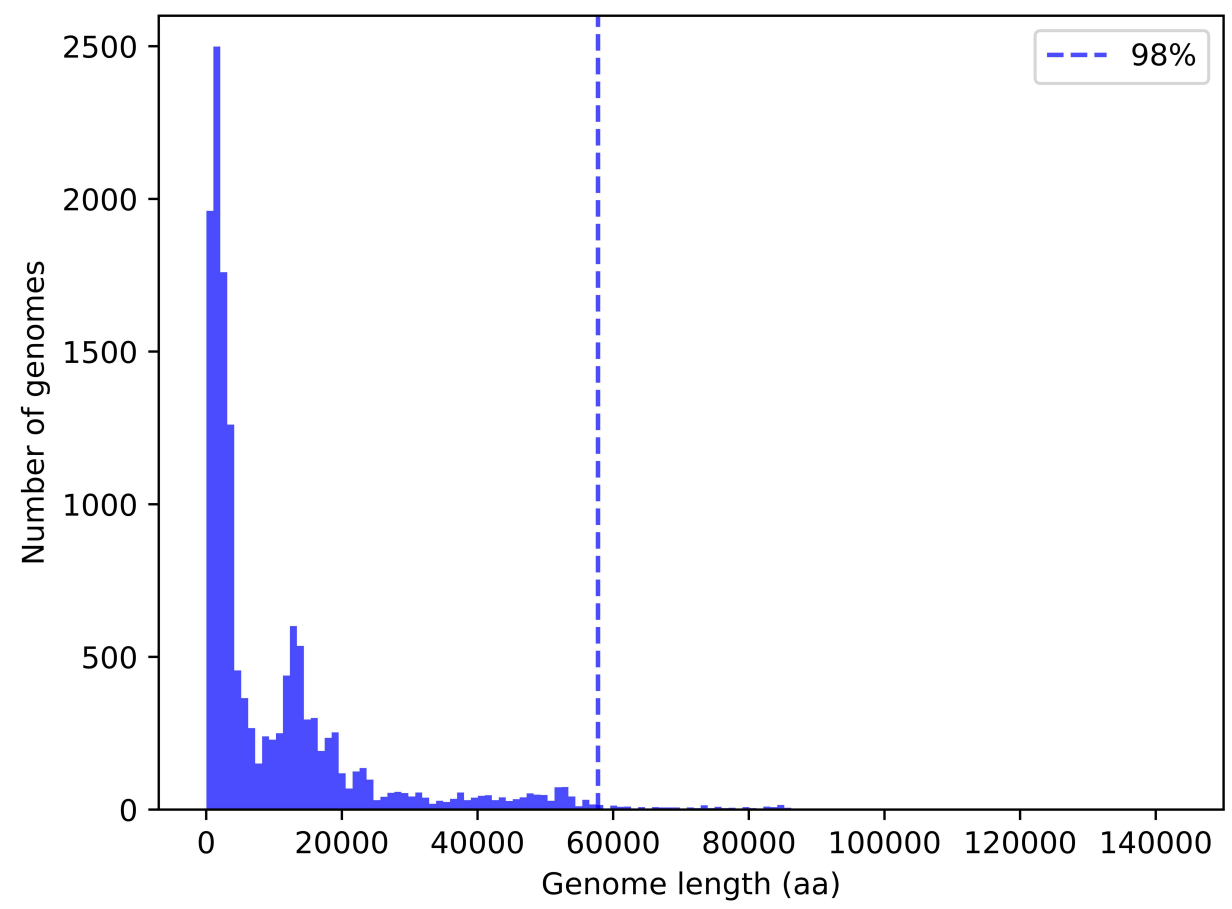

Supplementary Figure S1: **Viral genome length distributions in the NCBI Virus Database.** This histogram shows that a context length of 61k amino acids allows us to process over 98% of the 14,436 complete genomes of the database.

S2 Development of the PPI Inference Model (Stage 1)

To extend ESM-2's context window for PPI inference, we evaluated two positional encoding strategies (RoPE and ALiBi) and three sparse attention mechanisms (Longformer, Shifted Sparse Attention, and BigBird).

The evaluation of positional encoding strategies showed that ALiBi exhibited superior performance in handling extended sequences. Although RoPE demonstrated robust performance within its original training context, its effectiveness deteriorated significantly beyond 4,000 aa. In contrast, ALiBi maintained consistent perplexity (1.99) across sequence lengths extending up to 13,000 aa (Supplementary Figure S2).

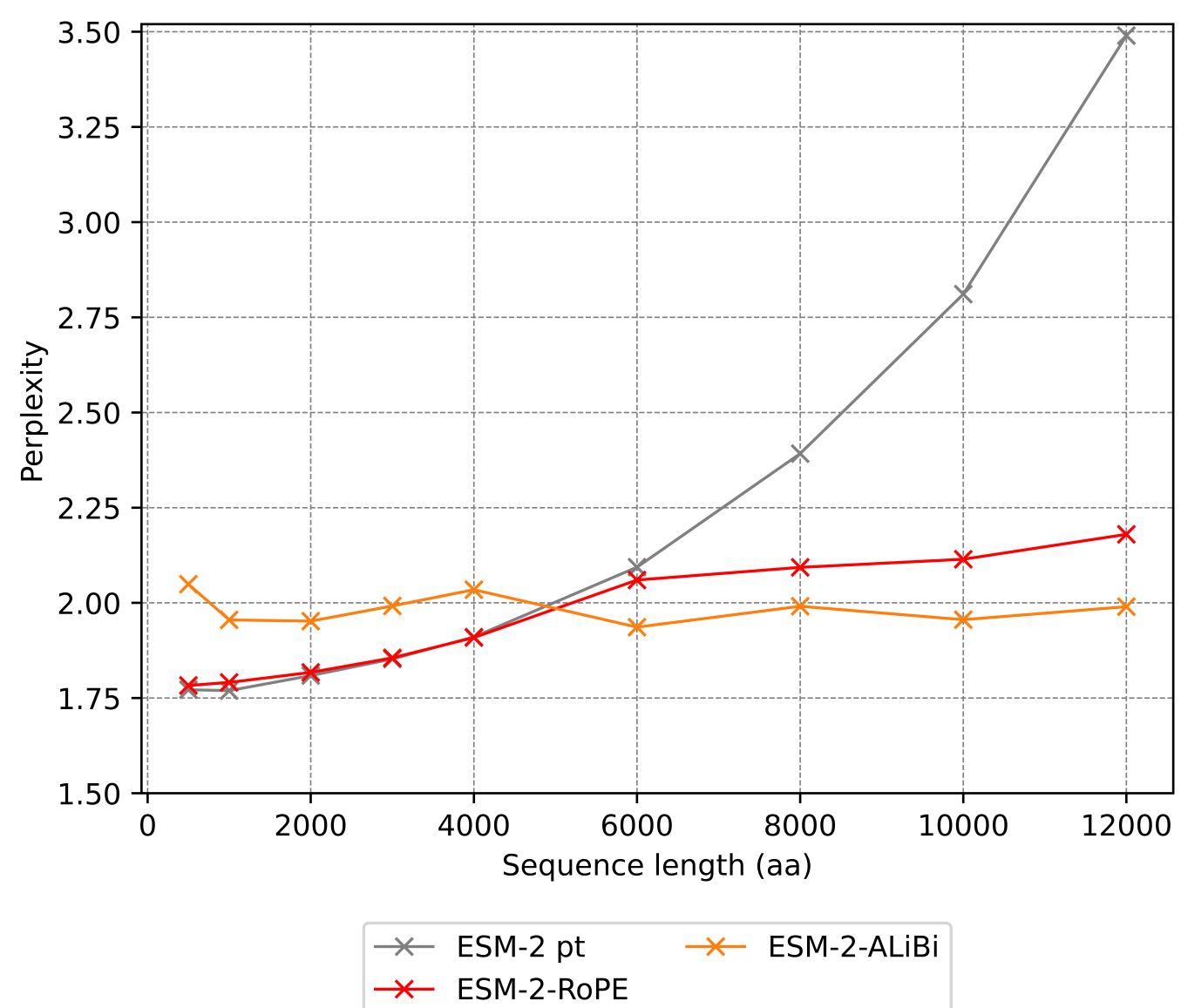

Supplementary Figure S2: **Perplexity evaluation of positional encoding methods for fine-tuning.** Performance metrics for ESM-2-pt and fine-tuned variants using Sliding Window (SW) attention mechanism with RoPE and ALiBi positional encoding. Perplexity values represent mean across sequences ranging from 500 to 13,000 amino acids.

Among the sparse attention mechanisms tested, Longformer provided the optimal balance between performance (perplexity: 1.99, silhouette score: 0.88) and computational efficiency (training time: 32s/step or 46 hours) (Supplementary Table S1). Based on these findings, we adopted ALiBi positional encoding combined with Longformer attention for ESM-2-PPI.

	ESM-2-pt	Longformer	Longformer + full	S^2-Attn	S^2-Attn + full	BigBird
Training context size	1,024	4,000	4,000	4,000	4,000	4,000
Training time (s/step)		32	32	36	36	126
Perplexity	3.17	2.18	**1.99**	2.22	2.05	2.02
Silhouette	0.65	0.85	**0.88**	0.86	0.85	0.84

Supplementary Table S1: **Performance metrics of ESM-2-pt and fine-tuned versions using different sparse attention patterns.** Longformer refers to the Sliding Window Attention pattern from [20], S^2-Attn to the Shifted Sparse Attention from [21] and BigBird to the eponymous attention pattern from [22]. '+full' means normalization and embedding layers are trainable during low-rank fine-tuning. Metrics are calculated on genome fragments ranging between 10,000 and 13,000 amino acids.

To validate that ESM-2-PPI captures biologically meaningful interactions, we analyzed genomic fragments from 83 viral genomes containing complete PolA, RNR, and HEL proteins. These genomes were identified by searching the

NCBI Virus database using BLAST and soft alignment [36, 37] against reference sequences for each protein, retaining only genomes where all three were detected with high confidence (e-value $< 10^{-3}$, coverage $> 80\%$). Supplementary Figure S3 shows that these known interacting pairs consistently rank among the highest-scoring interactions, particularly after excluding genomically adjacent pairs. A Wilcoxon–Mann–Whitney test confirmed a highly significant difference between the distributions ($p = 3.31 \times 10^{-22}$).

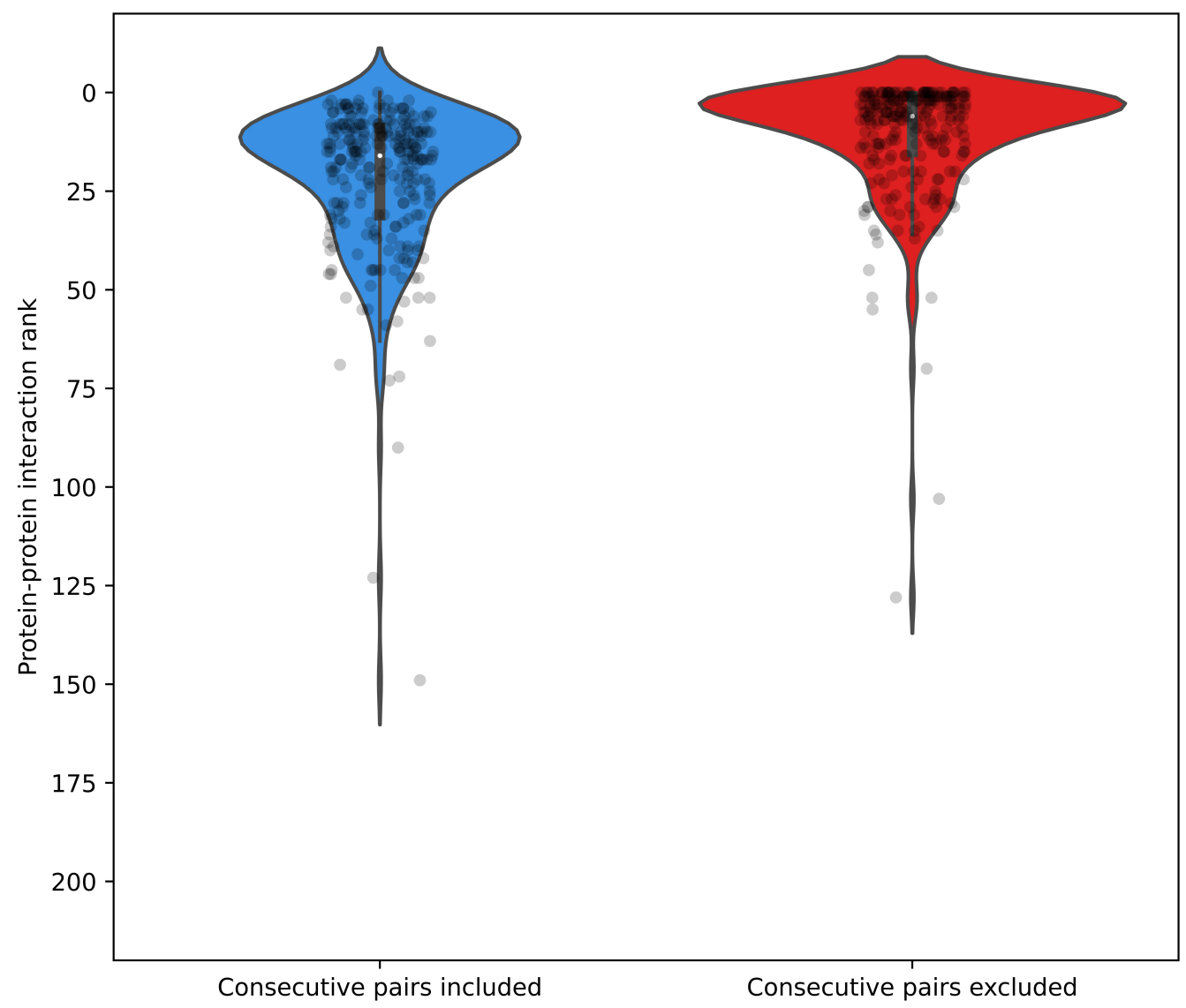

Supplementary Figure S3: **Distribution of interaction pair rankings of DNA polymerase I (PolA), ribonucleotide reductase (RNR), and helicase (HEL) across 83 viral genomes.** Violin plots represent the ranking positions of PolA-HEL, PolA-RNR, and RNR-HEL pairs (total n = 249). Left panel: rankings among all protein pairs within fragments. Right panel: rankings after excluding genomically adjacent protein pairs.

To select an appropriate interaction threshold, we evaluated different values of k (the number of top-ranked non-adjacent pairs to include). Supplementary Figure S4 and Supplementary Table S2 show that $k = 50$ retains 97.6% of known interacting pairs while remaining computationally tractable.

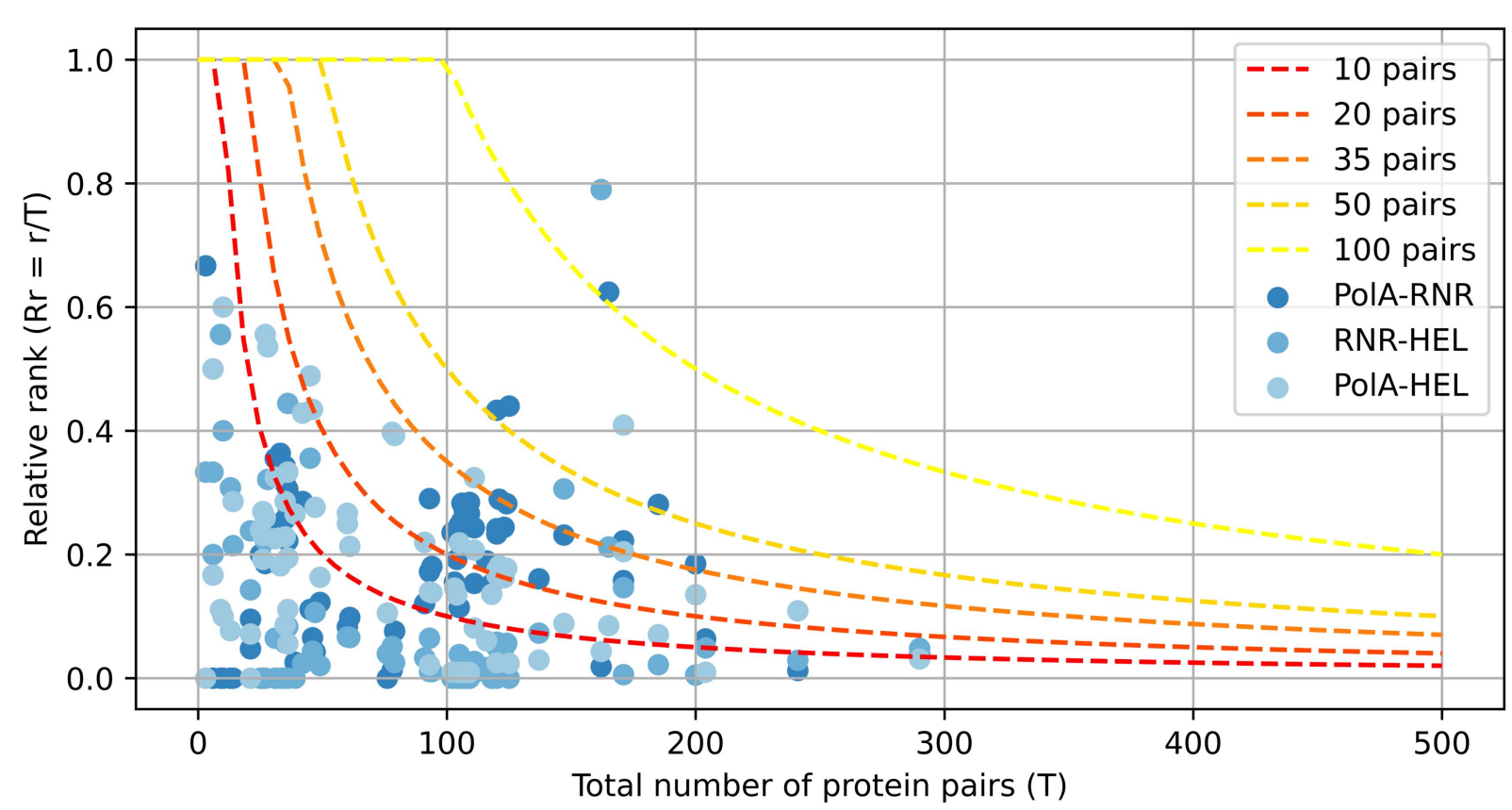

Supplementary Figure S4: **Selection of interaction threshold.** PolA-HEL, PolA-RNR, and RNR-HEL pairs shown as a function of total genomic pairs and relative genomic rank. Dashed curves denote selection thresholds; pairs below each curve are retained for attention calculations.

Threshold (number of selected pairs)	10	20	35	50	100
% studied pairs selected	64.0%	80.6%	94.4%	97.6%	99.2%

Supplementary Table S2: **Percentage of known interacting pairs** (PolA-RNR-HEL) captured at different selection thresholds.

S3 Using embeddings to predict taxonomy

To assess the taxonomic discriminability of the learned embeddings, we conducted a supervised classification analysis. For each protein or genome in the dataset, a 1,280-dimensional embedding vector was generated and paired with its corresponding taxonomic label. The dataset, comprising 100,000 proteins and 5,000 genomes, was randomly partitioned into training (80%) and validation (20%) subsets. To ensure a robust evaluation, we varied the number of taxonomic species from 5 to 100, as described in Section 3.9. A single-layer neural network with an intermediate hidden dimension of 2,560 was trained for 1,000 epochs using a learning rate of 1e-3. Model performance was evaluated using 5-fold cross-validation, and standard classification metrics were reported.

S4 Validation Using Protein Domain Annotations

To assess whether our embeddings capture functional information beyond taxonomic signals, we evaluated clustering and classification performance using Pfam protein family annotations. Pfam families group proteins by conserved domain structure and function, providing an orthogonal validation axis to taxonomy-based evaluations.

Supplementary Figure S5 presents silhouette scores and classification F1-scores using Pfam families as labels. Notably, silhouette scores are negative across all models (Supplementary Figure S5a), indicating that proteins from the same Pfam family do not form tight clusters in embedding space. This result is expected and reflects a key design principle of our approach: our embeddings are trained to encode genomic context, not solely domain identity. A helicase in a bacteriophage and a helicase in an RNA virus belong to the same Pfam family but occupy fundamentally different genomic contexts, and our models are designed to distinguish them. Embeddings that clustered purely by Pfam family would simply recapitulate single-protein models like ESM-2, rather than capturing the genomic relationships that are the focus of this work.

Despite this, classification performance remains strong (Supplementary Figure S5b), with LV-3C and LV-5B consistently outperforming ESM-2-pt and PST across all numbers of Pfam families evaluated. This indicates that Pfam-relevant information is encoded within the embeddings—it is simply not the dominant organizing signal. A linear classifier can extract domain identity even when genomic context is the primary structuring principle of the embedding space.

Taken together, these results demonstrate that our context-aware embeddings capture functional domain information at least as effectively as single-protein baselines, while additionally encoding genomic context that distinguishes proteins with identical domains but different genomic environments.

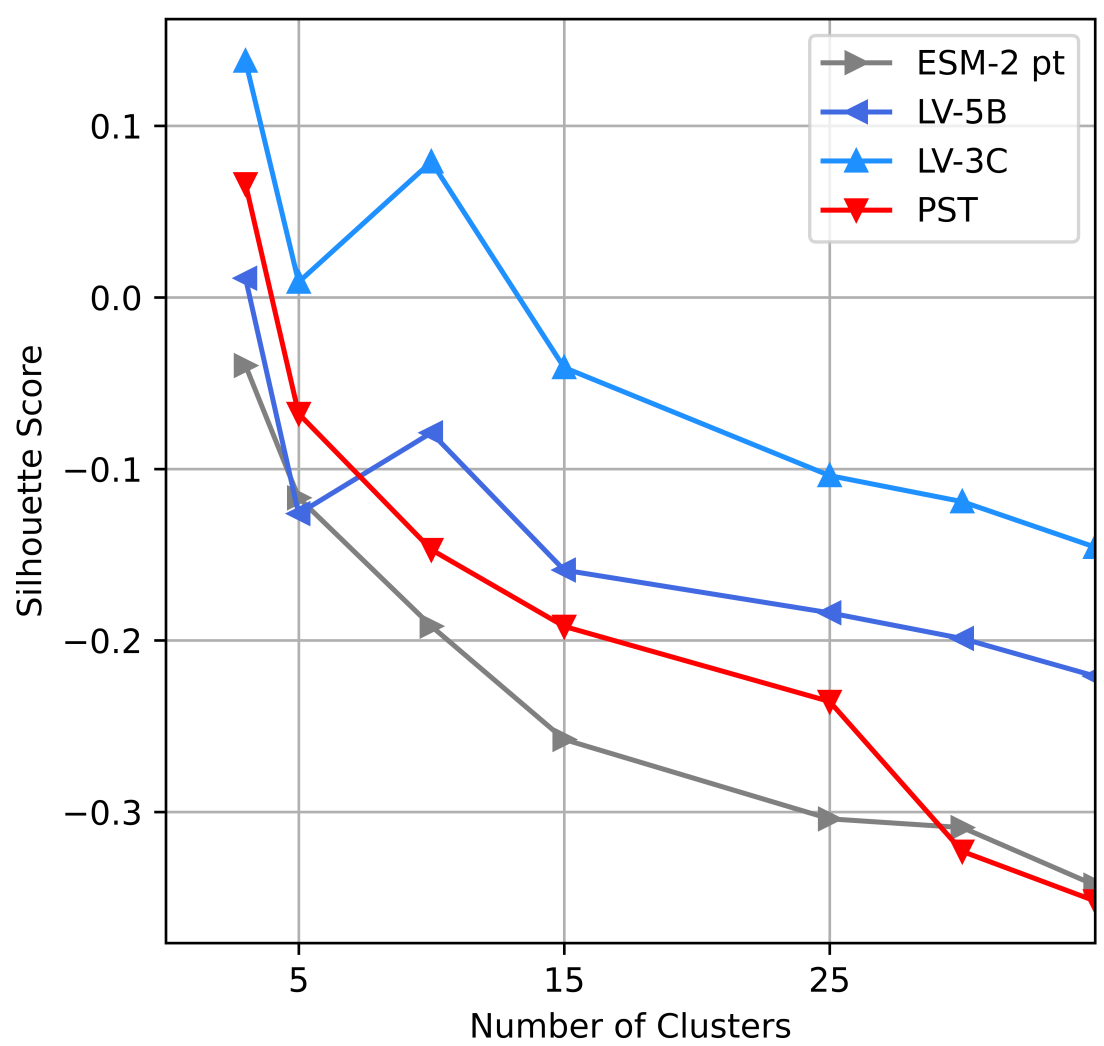

(a) Silhouette Score

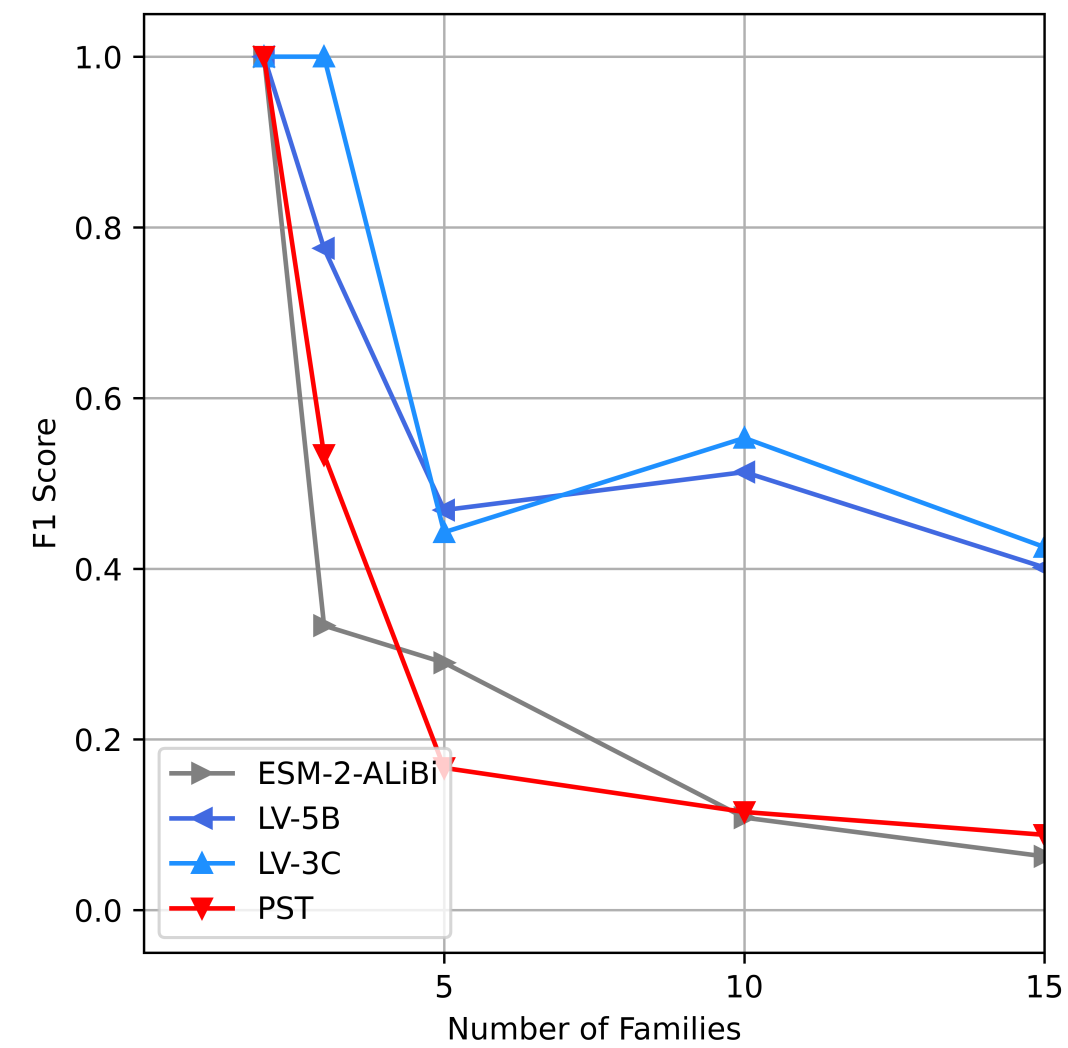

(b) Classification

Supplementary Figure S5: **Evaluation of embeddings using Pfam domain annotations. (a)** Silhouette scores using Pfam families as cluster labels. Negative scores across all models reflect that genomic context—not domain identity alone—is the primary organizing principle of the embedding space. **(b)** F1-scores for Pfam family classification. Despite low clustering coherence, LV-3C and LV-5B achieve strong classification performance, indicating that domain information is encoded but not dominant.

S5 Model Performance Across Viral Subgroups

Viral genomes vary substantially in size, structure, and evolutionary dynamics. To assess whether our models generalize across this diversity, we evaluated performance on RNA viruses—the most challenging and abundant subgroup in our dataset (approximately 70% of training genomes).

RNA viruses present unique modeling challenges: their high mutation rates and rapid evolution generate greater sequence diversity, while their broad host ranges and varied genome organizations (segmented, ambisense, positive/negative-sense) introduce additional complexity. If our models perform well on this demanding subgroup, we would expect comparable or better performance on structurally simpler dsDNA and ssDNA viruses.

Supplementary Figure S6 compares model performance on RNA viruses (solid lines) versus all viruses (lighter lines). As expected, performance on RNA viruses is slightly lower than on the full dataset; the latter being lifted by the inclusion of less complex viral groups. Critically, however, LV-3C and LV-5B consistently outperform both ESM-2-pt and PST baselines on RNA viruses across all evaluation metrics. These results demonstrate that our models capture biologically meaningful signals even within the most challenging viral subgroup, suggesting robust generalization across viral diversity.

S6 Controls for the Interaction-Inference Step

To test whether the interaction-inference step itself improves the model, we ran a controlled comparison in which the only parameter that varied was which long-range links each model received. The random conditions use three independent draws whose results we average. Additionally, because the inferred links tend to fall at particular genomic distances, we also included a distance-matched control that draws random links at distances similar to those of the inferred pairs, so that any difference cannot be attributed to genomic distance alone. Together with a model that receives no long-range links and connects only neighboring proteins, this gives eight models.

- **Inferred** (1 model). The 50 top-ranked pairs from the interaction-inference model (ESM-2-PPI).
- **Random** (3 models). 50 pairs drawn uniformly at random, averaged over three independent draws.

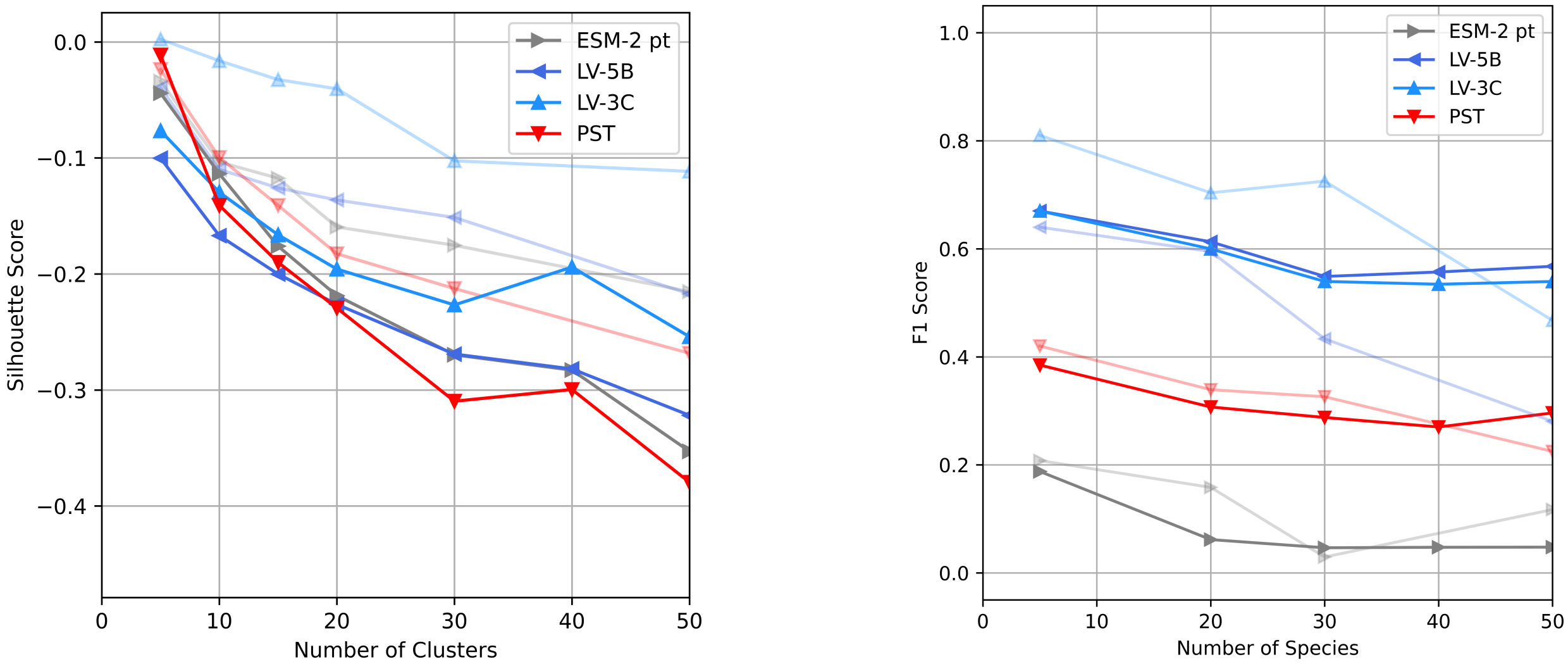

(a) Silhouette Score

(b) Classification

Supplementary Figure S6: **Model performance on RNA viruses compared to all viruses. (a)** Silhouette scores and **(b)** F1-scores for taxonomic species classification, comparing LV-3C, LV-5B, PST, and ESM-2-pt. Solid lines: RNA viruses only; lighter lines: all viruses. Despite the greater complexity of RNA viruses, our models maintain consistent improvements over baselines.

- **Distance-matched** (3 models). 50 random pairs drawn to match the genomic-distance range of the inferred pairs, averaged over three draws.
- **No-link** (1 model). Only neighboring proteins are connected.

We used the ESM-2-35M-parameter model so that all eight could be trained from scratch under identical conditions. All models share the same architecture, initial weights, training data and order, masking, and optimizer, so any difference in their results can come only from which pairs are connected.

Table S3 reports held-out perplexity on 600 genomes. Adding 50 random links leaves perplexity essentially unchanged relative to having no long-range links (16.124 versus 16.120), whereas adding the same number of inferred links lowers it (lower is better). The distance-matched control performs like the other controls, so the advantage reflects which proteins are paired rather than how far apart they occur.

Model	Held-out perplexity
No long-range links	16.120
50 random links (average of 3)	16.124
50 distance-matched links (average of 3)	16.119
50 inferred links	**16.064**

Supplementary Table S3: **Held-out perplexity for the interaction-inference controls.** Lower is better. Only the inferred links lower perplexity relative to having no long-range links.

The genome-wide change is modest (approximately 0.4%), but this figure represents an average across all positions in the genome. Because the 50 links are relevant only to the minority of positions that occur within an inferred PPI link, the majority of positions are unaffected. Averaging this small number of positions against the large number of unaltered ones minimizes the magnitude of the effect. The effect is nonetheless consistent across genomes as the inferred model achieves lower perplexity than the random average on 554 of 600 genomes (92%), and it outperforms the lowest perplexity attained by any of the three random models on a per-genome basis for 78% of genomes.

The improvement in perplexity is also concentrated among the pairs assigned the highest scores by the inference model. We ranked positions according to the reduction in per-position loss conferred by the inferred links relative to the control,

and examined the distribution of the most-improved positions. In Table S4, the baseline rate denotes the fraction of the genome occupied by the proteins in the retained pairs—the value expected by chance—and the top-100 enrichment quantifies how much more frequently the 100 most-improved positions coincide with those pairs than this baseline predicts. The enrichment increases with the confidence of the pairs considered, ranging from approximately 1.4-fold when all 50 pairs are pooled to approximately 11-fold for the highest-scoring pairs. The improvement therefore scales with the model's inferred PPI score rather than emerging at an arbitrary threshold.

Edges kept (by confidence)	Baseline rate	Top-100 enrichment
top 2%	4.1%	11.1×
top 5%	9.0%	7.2×
top 10%	13.8%	5.2×
top 20%	25.6%	3.1×
top 50%	50.4%	1.7×
all inferred pairs	71.6%	1.4×

Supplementary Table S4: **Enrichment of improved positions on high-scoring inferred pairs.** The most-improved positions fall disproportionately on the proteins the inference model ranks most highly.

With every other setting held identical, 50 randomly chosen links give no measurable improvement over having no long-range links, whereas 50 links chosen by the inference model lower held-out perplexity. The advantage is consistent across genomes and concentrates on the pairs the model scores highest, which are the same pairs an independent, separately validated model ranks as most likely to interact.
